# Quantitative Analysis of the Human Milk Whey Proteome Reveals Developing Milk and Mammary-Gland Functions across the First Year of Lactation

**DOI:** 10.3390/proteomes1020128

**Published:** 2013-09-03

**Authors:** Qiang Zhang, Judy K. Cundiff, Sarah D. Maria, Robert J. McMahon, Jessica G. Woo, Barbara S. Davidson, Ardythe L. Morrow

**Affiliations:** 1 Mead Johnson Nutrition, 2400 West Lloyd Expressway, Evansville, IN 47721, USA; E-Mails: judy.cundiff@mjn.com (J.K.C.); sarah.maria@mjn.com (S.D.M.); bob.mcmahon@mjn.com (R.J.M.); 2 Cincinnati Children’s Hospital Medical Center, Cincinnati, OH 45213, USA; E-Mails: Jessica.Woo@cchmc.org (J.G.W.); Barbara.Davidson@cchmc.org (B.S.D.); Ardythe.Morrow@cchmc.org (A.L.M.)

**Keywords:** human milk, milk proteins, immunoglobulins, complement system, cellular processes

## Abstract

In-depth understanding of the changing functions of human milk (HM) proteins and the corresponding physiological adaptions of the lactating mammary gland has been inhibited by incomplete knowledge of the HM proteome. We analyzed the HM whey proteome (*n* = 10 women with samples at 1 week and 1, 3, 6, 9 and 12 months) using a quantitative proteomic approach. One thousand three hundred and thirty three proteins were identified with 615 being quantified. Principal component analysis revealed a transition in the HM whey proteome-throughout the first year of lactation. Abundance changes in IgG, sIgA and sIgM display distinct features during the first year. Complement components and other acute-phase proteins are generally at higher levels in early lactation. Proteomic analysis further suggests that the sources of milk fatty acids (FA) shift from more direct blood influx to more *de novo* mammary synthesis over lactation. The abundances of the majority of glycoproteins decline over lactation, which is consistent with increased enzyme expression in glycoprotein degradation and decreased enzyme expression in glycoprotein synthesis. Cellular detoxification machinery may be transformed as well, thereby accommodating increased metabolic activities in late lactation. The multiple developing functions of HM proteins and the corresponding mammary adaption become more apparent from this study.

## 1. Introduction

**Figure 1:**
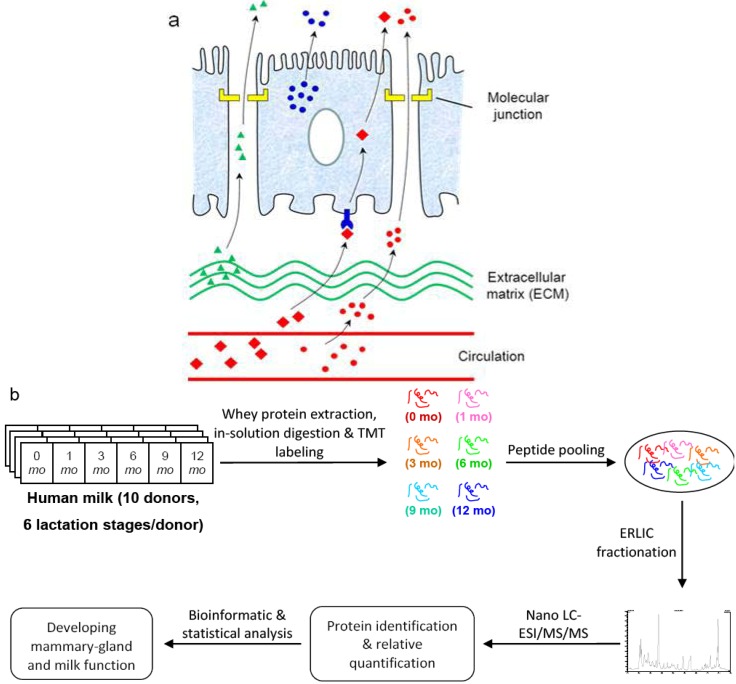
Quantitative analysis of human milk proteome during the first year of lactation. (**a**) Milk proteome comprises proteins synthesized within the mammary gland (blue circles), as well as proteins either passing through blood circulation (red circles and red diamonds) or released from mammary extracellular matrix (green triangles); (**b**) Experimental design: milk whey proteins at six different lactation stages were extracted, digested, labeled, fractionated and analyzed with mass spectrometry as described in the Experimental Section.

The majority of proteins found in human milk are secreted from mammary epithelial cells, though in addition, proteins may cross the mammary epithelial barrier into milk from either maternal blood or the extracellullar matrix (ECM) underlying the mammary epithelium (Figure 1a) [1]. Improved understanding of the lactating mammary gland may provide novel insights into the changes in milk composition and function during feeding. Considerable progress has been made in understanding the lactating mammary gland of ruminants and milk secretion in the past several decades [2]. Functional mammary genomic and transcriptome studies have been carried out for several farm animal species, including the goat mammary transcriptome [3], bovine mammary transcriptome [4,5] and cattle genome [6]. Similar studies in humans are scarce, which can be ascribed to the absence of non-invasive approaches for examining the human mammary gland during breastfeeding and the lack of cultured cell lines able to secrete milk for *in vitro* studies [7].

Proteins and other nutrient species in breast milk promote the health, growth and development of infants in many ways in addition to simple nutrition [8,9,10,11,12,13,14,15]. Despite the increased understanding of milk functionality, there is little information available regarding the development of milk function over the course of lactation where milk may contribute differently during the development from a newborn to a more mature infant. In an effort to further explore the benefits that human milk can provide, numerous studies have been carried out to investigate the composition and functionality of milk proteins [16,17,18,19,20,21,22,23,24,25]. Our initial comparison of human milk proteins between two lactational stages (1 week and 3 months postpartum) expanded the number of proteins identified and demonstrated quantitative differences between the two stages [26]. However, the two lactational time points can provide only a rudimentary depiction that is inadequate to fully understand the developing milk biology and function. For example, Goldman *et al*. has demonstrated that levels of milk sIgA are dynamic across the first year of lactation, with an initial decrease followed by an increase after 6 months [27]. Resolving such changes in protein abundance that are not unidirectional would require study of a minimum of three time points. Moreover, a deep quantitative proteome of human milk over the course of lactation is not yet available. In consequence, the developing milk function and the corresponding molecular adaptation of mammary physiology during breastfeeding remains poorly understood. 

The goal of this study is to establish a comprehensive quantitative proteome of human milk which can provide insights into the development of milk function and the accompanying transformations in the mammary gland over the entire first year of lactation. In this report, we have used a tandem mass tagging (TMT) approach to explore the changes of relative whey protein abundance in human milk produced from 1 week to 12 months after birth from a cohort of women using standardized milk collection. We further describe the significant changes in protein abundance over the course of lactation. With the establishment of quantitative protein compositions at multiple stages of lactation, we delineate transforming milk and mammary gland function in carbohydrate, lipid and glycoprotein metabolism and the immune system. Though change of protein abundance is, of course, not the only predictor of its transforming biological function, this improved understanding may encourage further exploration of the crucial dietary benefits that milk can provide to support the different stages of infant development over the course of lactation. 

## 2. Experimental

### 2.1. Chemicals

Liquid chromatography mass spectrometric- or proteomic-grade ammonium bicarbonate (NH_4_HCO_3_), acetic acid (HOAc), formic acid (FA), acetonitrile (ACN), H_2_O and trypsin were purchased from Sigma-Aldrich (St. Louis, MO, USA). Criterion Tris-HCl gradient gels, Laemmli sample buffer (4% SDS, 20% glycerol, 10% 2-mercaptoethanol, 0.004% bromophenol blue and 0.125 M TrisHCl, pH 6.8), Tris/Glycine/SDS running buffer (25 mM Tris, 192 mM glycine, 0.1% SDS, pH 8.3), Coomassie blue stain and Immun-Star Western C chemiluminescent reagent were purchased from Bio-Rad Laboratories (Hercules, CA, USA). 

### 2.2. Milk Samples

Human milk samples were obtained from ten healthy donors in Cincinnati, Ohio. Samples from day 6 to 8 after childbirth are denoted as month 0 and termed transitional milk, while additional samples collected from the same mothers at 1, 3, 6, 9 and 12 months after birth are termed mature milk. All samples were collected using a standardized protocol approved by Cincinnati Children’s Hospital Medical Center institutional review board. Samples were collected from one breast, aliquoted and frozen at −80 °C. When ready for use, milk was thawed at 4 °C, then ultra-centrifuged at 4 °C (100,000 ×*g* for 60 min) so that samples had a pellet of casein micelles on the bottom, a fat layer on the top, and delipidated whey supernatant in the middle. To obtain protein samples, the whey layer was filtered using a 10 kDa molecular-weight cut-off device (Millipore, Billerica, MA, USA) and subjected to buffer exchange with water.

### 2.3. In-Solution Tryptic Digestion, Isobaric TMT Labeling and ERLIC Fractionation

Protein concentration for filtered whey samples was determined with Dumas combustion methodology using an FP-2000 analyzer (LECO, St. Joseph, MI, USA). 100 μg of protein was removed from each sample for reduction and alkylation, followed by tryptic digestion, isobaric tagging, quenching of unreacted TMT reagents and peptide pooling according to the TMT protocol (TMT 6-plex Isobaric Label Reagent Set, Thermo-Fisher Scientific, San Jose, CA, USA) with the following modification in protein precipitation: after protein alkylation, buffer exchange with 100 mM TEAB was used as a replacement step for overnight cold-acetone precipitation. For each of the 10 donors, milk samples collected at 0, 1, 3, 6, 9 and 12 months of lactation were labeled with isobaric tags of 126, 127, 128, 129, 130 and 131 Da, respectively (Figure 1b). 

Each TMT 6-plex peptide mixture so obtained was pooled, lyophilized and resuspended in 200 μL of 90% ACN/0.1% HOAc for injection onto a 4 mm i.d. × 10 mm WAX guard column (PolyWAX LP, particle size 5 µm, pore size 1,000 Å, PolyLC Inc., Columbia, MD, USA) connected to a 2.1 mm i.d. × 200 mm WAX column (PolyWAX LP, particle size 5 µm, pore size 1,000 Å, PolyLC Inc., Columbia, MD, USA). ERLIC peptide separation was carried out via HPLC (U3000, Dionex, Sunnyvale, CA, USA) at a flow rate of 200 µL/min. A gradient was started with 100% A (98% ACN with 0.1% HOAc) for 10 min and ramped to 28% B (30% ACN with 0.1% FA) over 68 min and held for 20 min, followed by a gradient ramped to 100% B over 20 min and then held at 100% B for 10 min. UV absorption was monitored at 280 nm (Supplementary Figure S1). Approximately 40 fractions with retention times ranging from 20 to 100 min were collected at 2-min intervals. Each fraction was dried under reduced pressure, reconstituted in 20 μL of H_2_O/0.1% FA, stored at −80 °C, and thawed at 4 °C when ready for LC/MS analysis. 

### 2.4. Protein Digest Analysis by Nanocapillary Chromatography and Mass Spectrometry

Nanocapillary liquid-chromatographic electrospray-ionization tandem mass-spectrometric (LC-ESI/MS/MS) analysis was conducted on an LTQ/Orbitrap^XL^ hybrid mass spectrometer (Thermo-Fisher Scientific) coupled to a nano UltraHPLC (Eksigent, Dublin, CA, USA). For each fraction, 5 µL of tryptic digest was loaded into a 100 μm i.d. × 2.5 cm reversed-phase (RP) trap column packed with 5-μm C18 particles (IDEX, Oak Harbor, WA, USA). Peptide separations were carried out using an approximately 20-cm-long uncoated 75-μm i.d., 15-μm nanotip fused-silica column (New Objectives, Woburn, MA, USA) packed in-house with 3-μm C18 particles (Bruker-Michrom, Auburn, CA, USA). The separation was started with 96% mobile phase A (H_2_O/0.1% FA), 4% B (ACN/0.1% FA) for 2 min and then a 178 min linear gradient to 60% B, followed by a 10 min linear gradient to 80% B and held in 80% B for 10 min, with a flow rate of 300 nL/min. Full-scan mass spectra were acquired by the Orbitrap mass analyzer in the mass-to-charge ratio (*m/z*) of 380 to 1,800 and with a mass resolving power set to 60,000. Three data-dependent collision-induced-dissociation (CID) MS/MS scans of the most abundant ions from the full scan were then performed with an isolation width of *m/z* 3, a normalized collision energy setting of 35 and minimum signal threshold of 10,000 counts. In addition, three high-energy collision dissociations (HCD) were performed with a mass resolving power set to 7,500, an isolation width of 3 Da, a normalized collision energy setting of 50 and minimum signal threshold of 10,000 counts. Stepped collision energy was enabled for HCD with a normalized collision energy width of 10 in 2 steps. For both CID and HCD, each MS/MS spectrum was averaged from 3 microscans. The maximum injection time was 500 ms for parent-ion analysis and 150 ms for product-ion analysis. Target ions already selected for MS/MS with a repeat count of 2 during 30-s duration were dynamically excluded for 60 s. An AGC target value of 500,000 ions was used for full MS scans and 30,000 ions for MS/MS scans. Duplicate LC/MS/MS analyses were carried out for each of the 40 fractions for the 10 samples, resulting in a total of approximately 800 analyses. Only peptide ions with charge states of two or greater were selected for MS/MS interrogation. 

### 2.5. Protein Identification and Quantification

Peaks in MS/MS spectra are in centroid format and were converted into peak profiles using Mascot Distiller (Matrix Science, London, UK; version 2.4.3). MS/MS spectra with charges +2, +3 and +4 were analyzed using Mascot search engine (Matrix Science, London, UK; version 2.3.2). Mascot was set up to search against the human UniprotKB database (20,319 entries; version 2011_08) assuming trypsin as the digestion enzyme with a maximum of 1 missed cleavage allowed. The searches were performed with a fragment ion mass tolerance of 0.6 Da and a parent ion tolerance of 10 ppm. Iodoacetamide derivatization of cysteine and TMT 6-plex derivatization of *N*-terminus and lysine were specified in Mascot as fixed modifications. Deamidation of asparagine and glutamine, oxidation of methionine, and acetylation of the N-terminus were specified in Mascot as variable modifications. The Scaffold program (Proteome Software Inc., Portland, OR, USA; version 3.4.9) was used to validate MS/MS-based peptide and protein identifications. Peptide identifications were accepted if they could be established at greater than 95% probability as specified by the Peptide Prophet algorithm [28]. Protein identifications were accepted if they could be established at greater than 99% probability and contained at least two identified peptides by the Protein Prophet algorithm [29]. Proteins that contained similar peptides and could not be differentiated based on MS/MS analysis alone were grouped to satisfy the principles of parsimony. Technical false-discovery rate (FDR) was estimated by search against a decoy database. Relative intensities of TMT 6-plex reporter ions for a given peptide were obtained from the Mascot program. The protein ratios were calculated using the weighted average of the individual ratios of the peptides that can be assigned to that protein. Peptide identifications that can be assigned to more than one protein were removed to avoid errors in protein quantification. 

### 2.6. Data Analysis

Protein ratios were calculated by comparing protein abundance at each time point (1, 3, 6, 9 and 12 months) to month 0, for each of the10 subjects (50 total possible ratios). In each technical replicate, proteins used in quantitative analysis were those that had at least 15 of 50 (30%) possible protein ratios calculated. All statistical analyses were performed using Partek software (Partek Inc., St. Louis, MO, USA; version 6.6). A 3-way ANOVA that accounts for the data hierarchy including biological subjects, technical replicates and lactation periods was carried out to assess the statistical significance of differences in protein abundance during the first year of lactation. Proteins with *p*-values less than 0.01 were considered to have significantly altered abundance. Mean protein ratios were used to construct hierarchical heat maps of protein ratios at 1, 3, 6, 9 and 12 months of lactation relative to month 0. Relative ratios were log_2_ transformed without shifting the ratios of each protein to mean of zero or scaling to standard deviation of one. Protein ratios were clustered by distance metric of magnitude and shape using Euclidean correlation and were linked using Average Linkage method. Principal component analysis (PCA) was performed on log_2_ protein ratios for each lactation stage of each individual. 

### 2.7. Bioinformatics Analysis

Proteins identified in this work along with the set of 976 milk proteins identified previously [26] were combined for Gene Ontology (GO) analysis. GO analysis of biological processes, cellular components and molecular functions and KEGG pathway analysis for the milk proteome were carried out using the Database for Annotation, Visualization and Integrated Discovery (DAVID) software using the *Homo sapiens* genome as reference [30,31]. Both GO and KEGG pathway analyses were reported with a Benjamini (FDR corrected) *p*-value [32]. Protein interaction networks were analyzed using Search Tool for the Retrieval of Interacting Genes/Proteins (STRING; version 9.0) [33]. Tightly connected protein interactions were clustered using the MCL algorithm in STRING.

### 2.8. Immunoblotting Analysis

Cytosolic acetyl-CoA acetyltransferase (ACAT2), apolipoprotein B, (APOB), beta-1,4-galactosyltransferase 1 (B4GALT1), fatty-acid synthase (FASN), fructose-1,6-bisphosphatase 1 (FBP1), α-L-fucosidase (FUCA1), glutathione synthetase (GSS) and gamma-glutamyltranspeptidase 1 (GGT1) antibodies were purchased from Santa Cruz Biotechnology Inc., (Dallas, TX, USA). Immunoblotting was performed for a series of whey proteins extracted from 0 to 12 month milk. Whey protein samples were mixed with an equal volume of Laemmli sample buffer (Bio-Rad) and loaded onto a Criterion polyacrylamide gel. Electrophoreses were run for 50 min, transferred onto nitrocellulose membranes according to the instructions for the Trans-Blot Turbo transfer system (Bio-Rad), blocked 1 h in SuperBlock (Thermo-Pierce) at r.t., and incubated overnight at 4 °C with primary antibodies at various dilutions, followed by incubation with secondary antibodies (various dilutions) for 1 h at r.t. Bands were detected using Immun-Star Western C chemiluminescent reagent and quantified using ChemiDoc (Bio-Rad). 

## 3. Results

### 3.1. Quantitative Human Milk Proteome during the First Year of Lactation

Because of the dearth of non-invasive ways to monitor lactation within the mammary gland of healthy breastfeeding women, our proposal is to apply qualitative and quantitative milk secretome data over one year of lactation as probes for the developing cellular processes and milk functions. We approached this through TMT-labeled quantitative proteomic analysis of breast milk from 10 donors. Each of the 10 donors contributed to a set of milk samples at six different stages of lactation from 0, 1, 3, 6, 9 and 12 months. Water-soluble whey proteins were extracted through delipidation and casein precipitation, followed by *in vitro* tryptic digestion. For each donor, samples at each of the 6 different lactation stages were tagged with a unique TMT label from 126 to 131 Da (Figure 1b). 

To reduce sample complexity, strong-cation-exchange (SCX) chromatography that separates peptides based on charge has been generally used to fractionate tryptic peptides. However, the high concentration of nonvolatile salts often used in SCX can lead to the blockage of the HPLC autosampler and MS inlet, thus requiring a desalting step prior to online RPLC-MS/MS analysis. Alternatively, Electrostatic repulsion-hydrophilic interaction chromatography (ERLIC), introduced by Alpert, was recently applied to iTRAQ-based peptide fractionation [34,35]. In the ERLIC mode, separations of peptides are based on the combined effects of electrostatic repulsion and hydrophilic interaction. It has been noted that ERLIC separates peptides with a broader range of pI (isoelectric point) and GRAVY (grand average of hydropathy) values than does SCX. In this study, we further extended the use of ERLIC to fractionate TMT-labeled peptides and coupled it to RPLC-MS/MS analysis on an LTQ/Orbitrap^XL^ using CID/HCD-based fragmentation (Figure 1b). ACN and H_2_O were used in the mobile phases for ERLIC and no desalting step was required for robust online RPLC-MS/MS analysis. At the high levels of ACN used here, a portion of the tryptic digest remained in suspension rather than in solution, as has been noted by others [36,37]. The suspensions were injected anyway, as is. A potential drawback to this practice is the possibility that a particular peptide might be injected partly in solution and partly in suspension. It would then elute at two widely spaced points in the gradient or else in a continuum rather than a discrete peak. No such situations were observed. This seems to expand the range of options in ERLIC.

Duplicate LC-MS runs were carried out for the sample sets to assess the data quality of the quantitative human milk proteome. A linear correlation coefficient was calculated for each of the protein abundance ratios *n*-month/0-month (*n* = 1, 3, 6, 9 and 12) between duplicate runs. In the example of donor 8, values of the correlation coefficient are 0.96, 0.98, 0.97, 0.97 and 0.97 for the proteins’ abundance ratios 1-month/0-month, 3-month/0-month, 6-month/0-month, 9-month/0-month and 12-month/0-month, respectively (Supplementary Table S1). Similarly for donor 9, corresponding values of correlation coefficient are 0.96, 0.98, 0.97, 0.97 and 0.97. Analysis of log_2_ protein abundance ratios *n*-month/0-month between the two technical replicates (log_2_ ratio (*n*-month/0-month)) showed good linearity in protein quantification within the range of −6 to +5 log_2_ ratio (Supplementary Figure S2). Log_2_ protein abundance ratios between the two technical replicates (log_2_ ratio (rep. 1/rep. 2)) showed a log_2_ ratio within ±0.35 for lower 10% and upper 90% percentiles (Supplementary Figure S2). The above observations suggest good technical reproducibility and quantification accuracy of the mass spectrometric analysis. 

**Figure 2:**
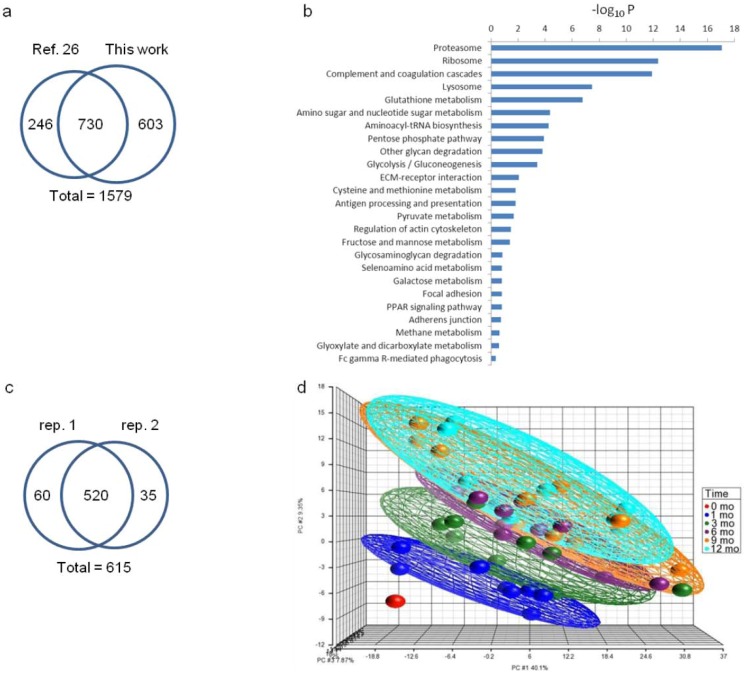
(**a**) Venn diagram comparison of proteins identified from this work and those from the previous work [26]; (**b**) KEGG pathway analysis of the 1,579 human milk proteins; (**c**) Venn diagram comparison of proteins quantified in technical replicate 1 and 2; (**d**) Principal component analysis of the quantitative human milk proteomes from all 10 donors in this study. Each donor contributed to milk at 0-*mo* (red), 1-*mo* (blue), 3-*mo* (green), 6-*mo* (purple), 9-*mo* (orange) and 12-*mo* (aqua) of lactation. Samples are grouped by ellipsoid according to lactation time. One dot in each of the 6 lactation stages represents the quantitative dataset from each of the 10 donors. The axes represent three principal components (PC#1, PC#2, PC#3) labeled with the percent of dataset variation explained by each.

With this setup, we identified 10,945 unique peptides in 15,981 unique spectra that can be assigned to 1,333 non-redundant proteins (³2 unique peptides) using the Protein Prophet algorithm with an estimated FDR < 1% (Dataset 1). We further merged the current dataset with a previous set of 976 whey proteins [26] which were identified using fractionation approaches of mixed-bed ion exchange chromatography [38] and SDS-PAGE, leading to a total of 1,579 human milk whey proteins (Figure 2a). The entire dataset of 1,579 protein entries was taken for GO annotation and KEGG pathway analysis (Figure 2b). Among the 1,333 proteins identified in this work, 615 proteins were quantified (Dataset 2) where 580 and 555 proteins meet or exceed the 30% ratio coverage threshold (Data analysis section) in technical replicates 1 and 2, respectively (Figure 2c). Milk is notoriously dominated by major proteins [26]. The relatively low quantitation percentage may be ascribed to the difficulty in tagging very low-abundance proteins in the presence of the high-abundance ones. Hierarchical clustering based on the 615 proteins at 6 stages of lactation is available as Supplementary Figure S3a.

### 3.2. Transforming Human Milk Proteome throughout the First Year of Lactation

Human mammary secretion starts with a small volume of colostrum within the first 48 h after delivery. Larger volumes of “transitional” milk are produced between approximately day 2 and 14 postnatally. By approximately 2 weeks after childbirth, secretion has been thought to stabilize, and the milk therein has traditionally been termed mature milk. In this study, abundance changes in milk proteins from transitional milk at month 0 to mature milk at 1, 3, 6, 9 and 12 months after childbirth reveal a striking yet consistent change in the milk proteome throughout the first year of lactation (Clustered heat map in Supplementary Figure S3a). Among the 615 quantified proteins, 471 proteins are considered to be expressed non-constitutively (FDR-adjusted *p* < 0.01) with respect to lactation time (Dataset 2). When compared to month 0, 141 and 320 proteins were found to be down- and up-regulated, respectively, with change greater or equal to 1.3-fold sometime during later lactation between 1 and 12 months (Venn diagram, Supplementary Figure S4). After grouping the dataset for all individuals by lactation stage, the PCA of variation in protein abundances showed distinct differences for each of the six lactation stages, with increasing separation of the fields from month 0 to each successive lactation stage from 1 to 12 months (Figure 2d). The larger degrees of separation are consistent with later lactation-stage milks that are increasingly different from the transitional milk (0 month). The observation suggests that the transformation of mature milk proteomes continues throughout the first year of lactation. PCA further revealed greater separation between milk samples at 0–1 month, 1–3 months and 3–6 months while less separation was observed for 6–9 month and 9–12 month sample*s*, suggesting that more substantial developmental changes in the milk proteome occur during the first 6 months of lactation. 

### 3.3. Abundance Changes of Proteins Involved in Fatty-Acid and Carbohydrate Metabolism

**Figure 3:**
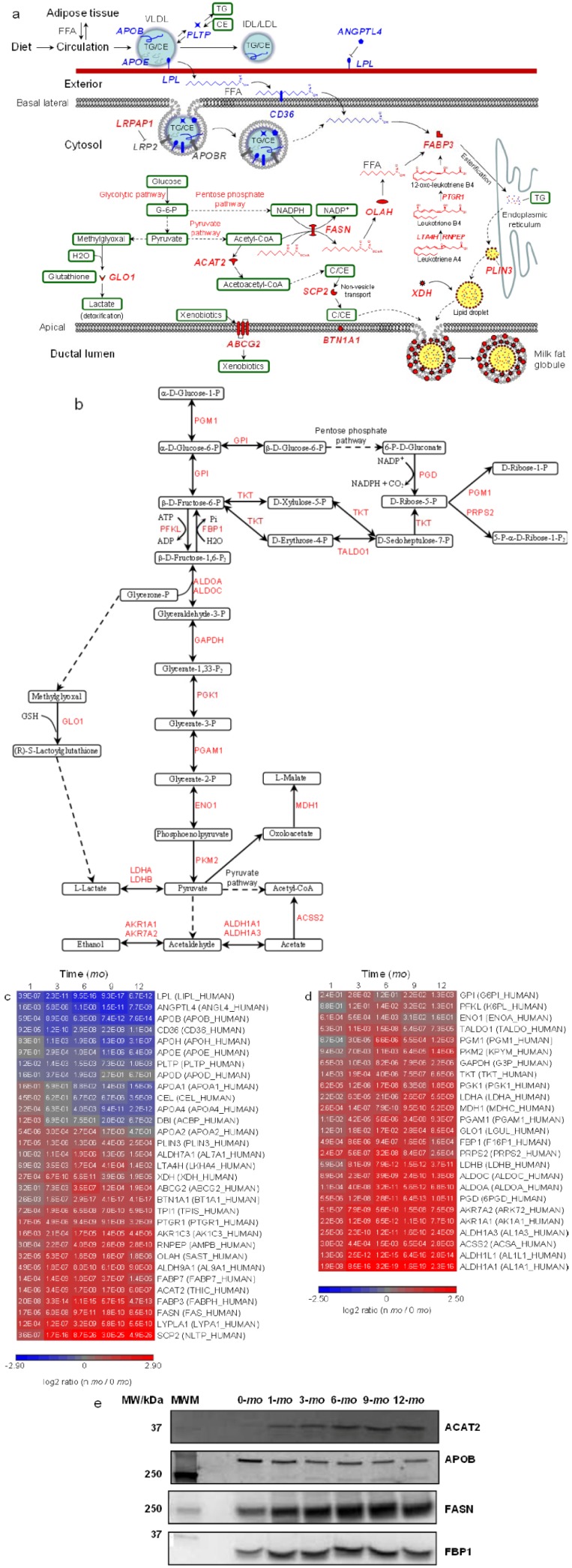
Quantitative analysis of carbohydrate and lipid metabolism. Proteins and pathways up- or down-regulated over lactation are in red and blue, respectively. Schematic representations of (**a**) extracellular and intracellular pathways in lipid transport and metabolism and (**b**) glycolysis pathway, pentose phosphate pathway and pyruvate pathway. Heat map representation of up- or down-regulated proteins involved in (**c**) lipid transport and metabolism and (**d**) glycolysis pathway, pentose phosphate pathway and pyruvate pathway. Protein abundances at n-*mo* (n = 1–12) are compared to 0-*mo* of lactation with significance *p*-values shown in the heat maps. Color legends are at log_2_ scale; (**e**) Examples of immunoblotting validation of proteins expressed in human milk at 0, 1, 3, 6, 9 and 12-*mo* of lactation. Whey proteins at each stage of lactation were pooled from 10 donors. MWM, molecular weight marker; ACAT2, cytosolic acetyl-CoA acetyltransferase; APOB, apolipoprotein B; FASN, fatty-acid synthase; FBP1, fructose-1,6-bisphosphatase 1.

Milk lipids such as triglycerides (TGs) are stored in mammary epithelial cells as cytosolic lipid droplets (LDs), where they can then be secreted into milk as milk fat globules (MFG). The fatty acid (FA) moiety of TGs in milk can be either recruited via lipolysis from blood LDs, primarily through chylomicrons (CM) and very low-density lipoproteins (VLDL) and to a lesser extent through intermediate-density lipoproteins (IDL) and low-density lipoproteins (LDL), or provided by *de novo* lipogenesis within the mammary epithelial cells [7,39]. Notably, apolipoproteins are constituent components essential for LD assembly (Figure 3a). A variety of apolipoproteins have been identified in human milk, including apolipoprotein A-I (apoA-I), A-II (apoA-II), A-IV (apoA-IV), B (apoB100), D (apoD), E (apoE) and H (apoH). Among them, apoB100 differs from other apolipoproteins in that it is required for the assembly of VLDL and it cannot be exchanged from the LDs where it is originally lipidated [40]. Lipoprotein lipase (LPL) catalyzes the lipolytic process by hydrolyzing TG molecules stored in circulating VLDL into glycerol and free fatty acids (FFA). The latter can then be transported into the cytosol of mammary epithelial cells via binding to CD36 [41]. Phospholipid transfer protein (PLTP) was thought to facilitate this process by transferring surface remnants from LDs and non-vesicle cholesteryl ester (CE) into LD [42]. The depletion of TG and enrichment of CE convert VLDL to IDL, and further to LDL. This conversion also reduces the binding affinity of apolipoprotein E (apoE) and other apolipoproteins, resulting in LDL particles with a single copy of the non-exchangeable apoB100 [43]. Angiopoietin-related protein 4 (ANGPTL4) plays an important role in regulating lipid metabolism in that it inhibits directly the lipolysis activity of LPL [44]. From early to late lactation milk samples, marked down-regulation of relative protein abundance was observed for apoB100, apoE, CD36, LPL, ANGPTL4 and PLTP (Figure 3c). The observation suggests both decreased blood-borne FFA release, via LPL-, ANGPTL4- and PLTP-regulated lipolysis of TG, and reduced cell FFA uptake, via CD36-facilitated translocation during later lactation. 

The influx of FFAs from blood LDs represent an important source for milk FA supplies, either as FFA or re-esterified TG. In parallel, FFA can be produced through *de novo* biosynthesis within mammary epithelial cells [45]. Cellular synthesis of FFA chains is catalyzed by fatty acid synthase (FASN). Thioesterase (OLAH) then hydrolyzes the thioester bond, leading to chain termination and the release of newly synthesized FFA. FABP3 plays a major role in intracellular FFA transport to the endoplasmic reticulum (ER) for synthesis of TG and eventual incorporation into LD for secretion. ACAT2 converts acetyl-CoA to acetoacetyl-CoA, which is essential for cholesterol and bile acid synthesis and steroidogenesis [46]. Sterol carrier protein 2 (SCP2) promotes cholesterol non-vesicle transport between intracellular membranes, cytosolic LDs and probably to the inner leaflet of the plasma membrane [47,48]. TG and cholesterol containing cytosolic LDs are eventually secreted in the form of MFG where BTN1A1, PLIN3 and XDH play important roles during the secretion process [49]. ACAT2, BTN1A1, FABP3, FASN, OLAH, PLIN3, SCP2 and XDH are all upregulated in late lactation compared to month 0 (Figure 3c). These findings, along with the downregulation of apoB100, apoE, CD36, LPL, ANGPTL4 and PLTP during late lactation, suggest greater FA intake from blood during early lactation as opposed to increased *de novo* mammary FA and cholesterol synthesis in late lactation. 

Proteins involved in glycolytic, pentose phosphate and pyruvate pathways are upregulated in later lactation (Figure 3b,d). G-6-P derived from the glycolytic pathway enters the pentose phosphate pathway to produce NADPH, while pyruvate, the end product of the glycolytic pathway, enters the pyruvate pathway to produce acetyl-CoA (Figure 3b). Acetyl-CoA and NADPH are required reaction intermediates during FFA synthesis. The extensive metabolomic analyses of human milk are scarce and therefore the abundance changes of acetyl-CoA and NADPH in milk over lactation remain unknown. Determination of the levels of milk metabolites would provide additional insights into the mammary production of acetyl-CoA and NADPH from carbohydrate metabolism and their roles in *de novo* mammary FFA synthesis processes. 

### 3.4. Abundance Changes of Proteins Involved in Glycoprotein Metabolism

**Figure 4:**
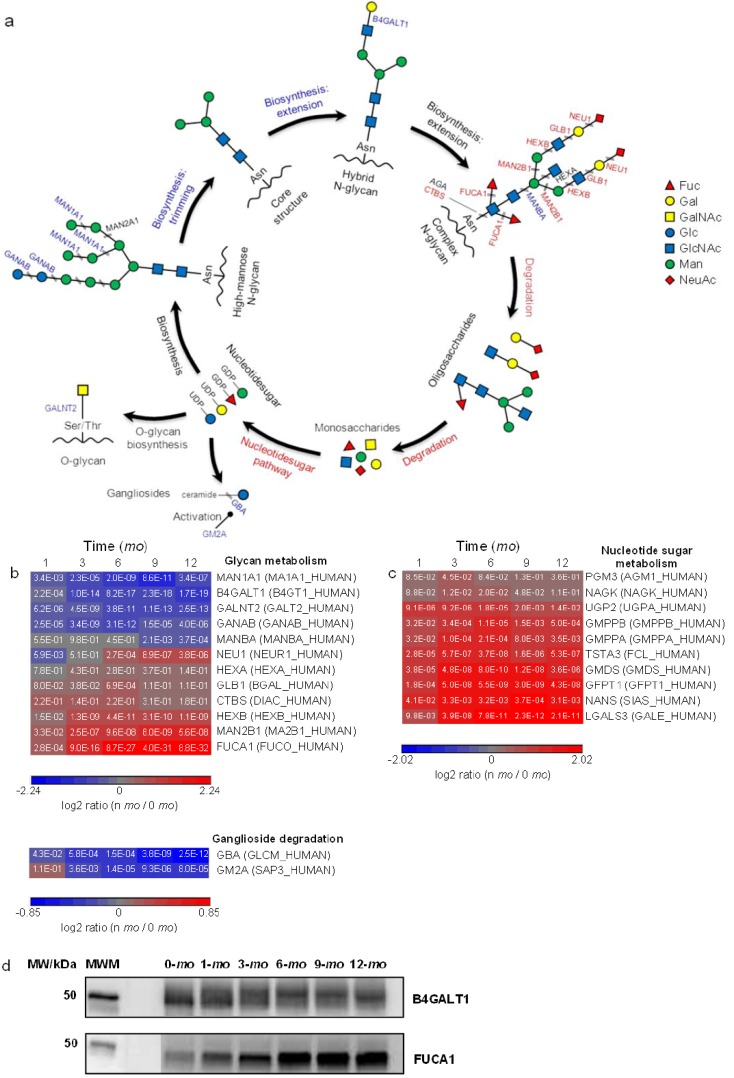
Quantitative analysis of glycoprotein metabolism. Proteins and pathways up- or down-regulated over lactation are in red and blue, respectively. (**a**) Schematic representations of glycoprotein metabolism; (**b**,**c**): heat map representation of up- or down-regulated proteins involved in glycan metabolism and nucleotide sugar metabolism, respectively. Protein abundances at n-*mo* (n = 1–12) are compared to 0-*mo* of lactation with significance *p*-values shown in the heat maps. Color legends are at log_2_ scale; (**d**) Examples of immunoblotting validation of proteins expressed in human milk at 0, 1, 3, 6, 9 and 12-*mo* of lactation. Whey proteins at each stage of lactation were pooled from 10 donors. MWM, molecular weight marker; B4GALT1, Beta-1,4-galactosyltransferase 1; FUCA1, α-l-fucosidase.

Carbohydrates are frequently bound to proteins via glycosylation, resulting in either *N*- or *O*-linked glycoproteins. *N*-glycans attach covalently to Asn that is typically part of a consensus sequence of Asn-X-Ser/Thr on the protein moiety via a glycosidic bond (GlcNAc-Asn) where X denotes a non-proline residue (Figure 4a). During the biosynthesis of *N*-linked glycoproteins, the protein-bound *N*-glycan precursors (high-mannose subtype) are first trimmed to a core glycan, then extended and modified to form hybrid and complex subtypes. All subtypes contain the same core oligosaccharide, (Man)_3_(GlcNAc)_2_ attached to Asn on the protein moiety (KEGG pathway: Glycan biosynthesis). *O*-glycans, on the other hand, bind covalently to Ser/Thr by a glycosidic bond, GalNAc-Ser/Thr, with as yet no known consensus sequence (Figure 4a). Among the proteins involved in *N*-glycan biosynthesis of trimming and extension, we identified β-1,4-galactosyltransferase 1 (B4GALT1), dolichol-phosphate mannosyltransferase subunit 3 (DPM3), neutral α-glucosidase AB (GANAB), α-mannosidase IA (MAN1A1), α-mannosidase 2 (MAN2A1) and mannosyl-glycoprotein acetylglucosaminyltransferase (MGAT1). GANAB and MAN1A1/MAN2A1 trim terminal Glc and Man, respectively, in immature high-mannose *N*-glycan precursors whereas B4GALT1 catalyzes the synthesis of the lactose motif, Gal-Glc(NAc), during *N*-glycan extension (Figure 4a). Among proteins that were classified in *O*-glycan synthesis, we identified GalNAc transferases, GALNT2, 3 and 5, which catalyze the formation of the GalNAc-Ser/Thr bond. B4GALT1, GALNT2, GANAB and MAN1A1 were quantified and all found to be downregulated in later lactation (Figure 4b).

In addition to the proteins involved in N-linked and O-linked glycoprotein synthesis, a number of proteins involved in complex *N*-glycan degradation were identified in milk. Lysosomal sialidase (NEU1), β-galactosidase (GLB1), β-hexosaminidase subunit α/β (HEXA/HEXB) and α-l-fucosidase (FUCA1) catalyze the removal of Neu/NeuNAc, β-d-Gal, GlcNAc and Fuc, respectively, from the non-core structure of *N*-glycans (Figure 4a). Lysosomal α-mannosidase (MAN2B1) and lysosomal β A mannosidase (MANBA) hydrolyze α-d-Man and β-d-Man, respectively, from the core structure (KEGG pathway: Other glycan degradation). Glycosylasparaginase (AGA) and di-*N*-acetylchitobiase (CTBS), both which cleave the Glc-Asn bond which joins *N*-glycans to proteins, were also identified. Among them, FUCA1, HEXB, MAN2B1 and NEU1 were found to be upregulated in later lactation (Figure 4b). 

Extensive breakdowns of glycans would likely require subsequent metabolic regulation. The KEGG pathway of nucleotide sugar metabolism was found to be overrepresented in milk (Figure 2b). Nucleotide sugars represent the active forms of monosaccharides that are used for glycan biosynthesis. A number of proteins involved in nucleotide sugar synthesis were quantified and found to be upregulated in late lactation (Figure 4c and Supplementary Figure S5). Collectively, we found that the proteins involved in the formation of *O*-glycan GalNAc-Ser/Thr bond, the trimming and extension of high-mannose glycans are downregulated whereas the proteins involved in the degradation of complex glycans and the synthesis of nucleotide sugars are upregulated as lactation progresses. The bidirectional nature of these changes refutes the explanation of overall increased glycoprotein turnover as, in that case, intensification of protein expression in both glycan synthesis and degradation would likely be expected. In addition to their intracellular biosynthesis, certain milk glycoproteins, such as immunoglobulins and ECM proteins, are known to be taken up from extracellular space via transepithelial transport [1,50]. Among the milk proteins identified, 445 milk proteins were classified as glycoproteins using GO and 213 of these proteins were quantified. Despite their partial extracellular origin, the majority of quantified glycoproteins were found downregulated in late lactation (Supplementary Figure S3b), an observation consistent with decreased intracellular glycoprotein biosynthesis over lactation. 

### 3.5. Abundance Changes of Proteins Involved in Detoxification Processes

Cellular toxins generated during various metabolic processes are typically deactivated prior to their excretion into blood circulation. During later lactation, cellular activities in the form of carbohydrate metabolism, *de novo* FA synthesis, and glycoprotein catabolism found in this work would likely be increased and could result in higher levels of metabolic by-products in milk. Though milk is considered adaptively optimal for nursing infants, unregulated production and/or excretion of metabolic by-products in the lactating mammary gland could lead to deleterious health outcomes for nursing infants. Enhancement of detoxification processes during later lactation would thus be a remarkable accommodation to safeguard the quality of milk against increasing metabolic activities of late lactation. 

**Figure 5:**
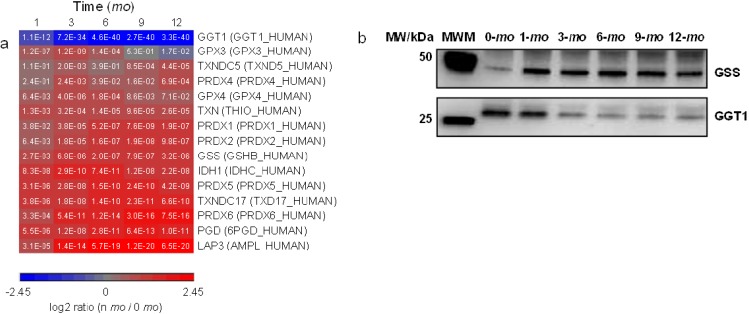
Quantitative analysis of glutathione metabolism. Proteins up- or down-regulated over lactation are in red and blue, respectively. (**a**) Heat map representation of up- or down-regulated proteins involved in glutathione pathway. Protein abundances at n-mo (n = 1–12) are compared to 0-mo of lactation with significance *p*-values shown in the heat maps. Color legends are at log2 scale; (**b**) Examples of immunoblotting validation of proteins expressed in human milk at 0, 1, 3, 6, 9 and 12-mo of lactation. Whey proteins at each stage of lactation were pooled from 10 donors. MWM, molecular weight marker; GSS, glutathione synthetase; GGT1, gamma-glutamyltranspeptidase 1.

One essential piece of machinery in the cellular detoxification mechanism is the homeostasis of redox couples, including glutathione (GSH)/glutathione disulfide (GSSG), NADPH/NADP^+^, and reduced/oxidized peroxiredoxin and thioredoxin [51]. GO analysis showed that the KEGG pathway of glutathione metabolism is significantly overrepresented in milk (Figure 2b). As lactation progresses, gamma-glutamyltranspeptidase 1 (GGT1), which initiates GSH breakdown [52], was found to be significantly downregulated whereas GSH synthetase (GSS), which catalyzes the formation of GSH, was found to be upregulated (Figure 5). The expressional changes of GGT1 and GSS suggest that GSH homeostasis increasingly favors GSH biosynthesis over the course of lactation. IDH1 and PGD convert NADP^+^ to its reduced form NADPH; peroxiredoxins (PRDX1, 2, 4, 5 and 6) eliminate peroxides generated during metabolism; and thioredoxins (TXN, TXNDC5 and 17) facilitate the reduction of other proteins by cysteine thiol-disulfide exchange. These proteins are all upregulated in late lactation. Methylglyoxal is a catechol-quinone toxin commonly formed during several cellular metabolic pathways [53]. GLO1, which catalyzes the hydroxylation/detoxification of methylglyoxal together with GSH, was also found to be upregulated in later lactation (Figure 3d). 

Exocytosis is an important path for secretion of mammary cellular contents into milk. Additionally, cellular metabolites can be excreted through protein transporters. For example, ATP-binding cassette sub-family G member 2 (ABCG2) plays an active role in eliminating xenobiotics and endogenous metabolic by-products from various tissues [54]. It has been found as well to transport a variety of small molecules from mammary gland into milk [55,56]. In this study, ABCG2 was found to be upregulated in mature milk compared to early milk (Figure 3c). Increased cellular detoxification through redox cycling and enhanced molecular secretion via the ABCG2 transporter is consistent with the observation of elevated expression of enzymes in carbohydrate metabolism, FA *de novo* synthesis and glycoprotein catabolism in later lactation. Collectively, the regulatory changes of proteins involved in cellular redox cycling and excretion may represent a means to rid both mammary tissue and milk of deleterious metabolic species during lactation. 

### 3.6. Milk Immunoglobulins

Milk contains antibody isotypes IgG, sIgA and sIgM that provide a means for mothers to confer immunity to their offspring [57]. Ig heavy chains, IGHA, IGHG and IGHM, are characteristic of IgA, IgG and IgM, respectively, and are used as measures of Ig isotypes in bottom-up proteomics. IgA and IgM in milk take the secretory form (sIgA and sIgM), which binds to a portion of the polymeric Ig receptor (PIGR) expressed in mammary epithelia [50]. To account for the secretory forms, PIGR was further taken as an indicator of sIgA and sIgM in milk [26]. For each of the Ig isotypes, it contains identical light chains, either kappa or lambda, resulting in monospecific antigen-binding sites. Ig kappa chain C (IGKC) and Ig lambda-like chain (IGLL) were taken as measures of kappa antibody and lambda antibody, respectively. 

**Figure 6:**
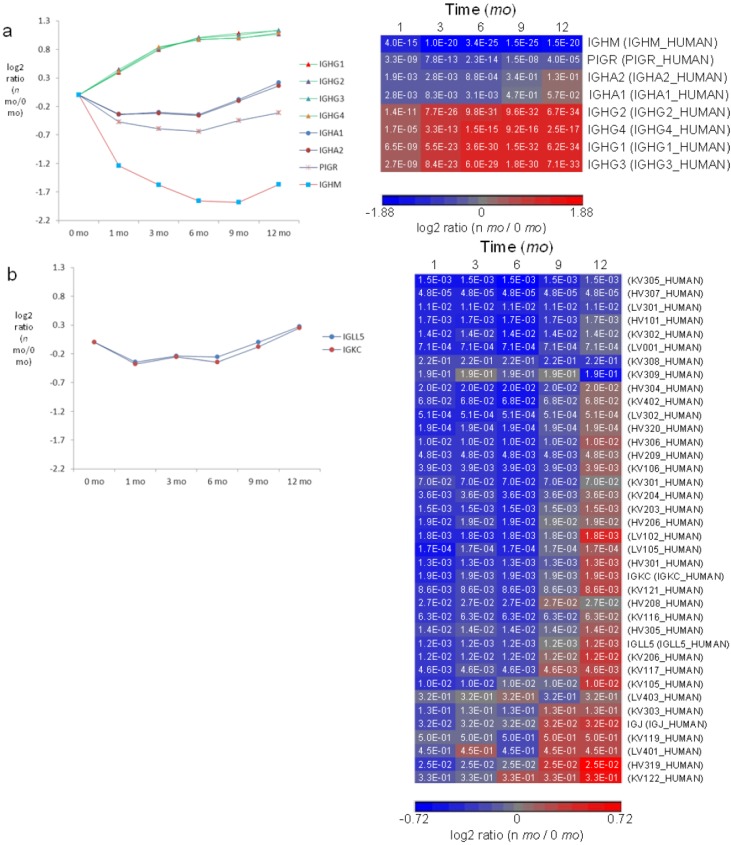
Quantitative analysis of immunoglobulins in milk. (**a**) Left panel: relative-abundance distribution of sIgM (IGHM and PIGR), sIgA (IGHA1, IGHA2 and PIGR) and IgG (IGHG1, IGHG2, IGHG3 and IGHG4). Right panel: significance *p*-values in hierarchical heat map; (**b**) Left panel: relative-abundance distribution of Ig classes of kappa and lambda. Right panel: significance *p*-values in hierarchical heat map of kappa and lambda chains and Ig variable-chain regions. HV, heavy chain variable region; KV, kappa chain variable region; LV, lambda chain variable region; IGKC, kappa chain constant region; IGLL, Ig lambda-like polypeptide; IGJ, Ig J chain. Color legends of heat maps are at log_2_ scale. Standard errors for each of IGHG1-4, IGHA1-2, IGHM, PIGR, IGLL and IGKC are available in Supplementary Figure S7.

Ig’s are traditionally thought to be present in breast milk at higher levels in early lactation compared to late lactation to provide a higher degree of protection to neonates whose defense systems are naive and immature compared to more developed infants. Unexpectedly, we found previously that sIgA_1,2_ and sIgM are upregulated in 1 week milk whereas IgG_1,2_ is upregulated in 3 month milk [26]. In this report, we quantified all four different IgGs and found that levels of IgG_1–4_ indeed increase consistently from week 1 to 12 months (Figure 6a). It is known that transplacental transport of maternal IgG leads to levels of IgG in newborns comparable to their mothers, but after birth, maternal IgG begins to be catabolized by the infant. In addition, endogenous production of IgG is delayed in the infant until about 6 months after birth. The combination of late production of endogenous IgG and catabolism of maternal IgG leads to a transient deficiency of IgG in infants during the period from birth to one year of age [58]. The increasing supply of milk IgG during the first 12 months may provide a means to complement the synchronous decrease in IgG and protect the basal propria underlying the GI epithelium from pathogenic infection during early infancy. Levels of sIgA_1,2_ (IGHA1, IGHA2 and PIGR) decrease from 0 to 1 month, remain stable from 1 to 6 months and rise again at 6 months (Figure 6a), an observation consistent with previous findings that measured sIgA_1,2_ during the first year of lactation [27]. Similarly, a modest increase in abundance was observed for sIgM from 9 to 12 months. 

While the regulatory patterns are significantly different among the isotypes of IgG, sIgA and sIgM, the relative abundance ratios among the four forms of IgG (IgG_1–4_) and between the two forms of IgA (IgA_1–2_) change proportionately over the course of lactation (Figure 6a). Similarly, the relative abundance ratios between kappa and lambda classes remain proportional over lactation (Figure 6b). In addition to the constant regions of Ig heavy chains and the lambda and kappa chains, a number of other Ig chain regions were quantified. Notably, their levels decrease in the first 6 months and increase from 6–12 months, a trend consistent with changes in sIgA (Figure 6b). Despite the unclear signaling pathway that regulates increased Ig expression in late lactation, the transition in sIgA expression at 6 months coincides with typical introduction of solid foods into the diets of infants and may represent a transforming defense mechanism provided by milk to combat pathogen exposure from increasingly diverse food sources. 

### 3.7. Both Classical and Alternative Pathways of the Complement Cascade are Present in Milk with a Higher Extent in Early Lactation

GO analysis showed that acute inflammatory response is the most significantly overrepresented biological process in milk proteins (Benjamini *p*-value = 3.8E−24). Acute-phase proteins (APPs) are a class of proteins whose expression changes in response to inflammation. The ten milk donors from this study did not present with symptoms of acute inflammation at the time of donations, yet the levels of APPs still underwent significant changes over the course of lactation. Quantitative proteomic analysis showed levels of APP proteins generally decreasing over the course of lactation, which suggests a higher degree of inflammatory response during early lactation (Supplementary Figure S6). 

**Figure 7:**
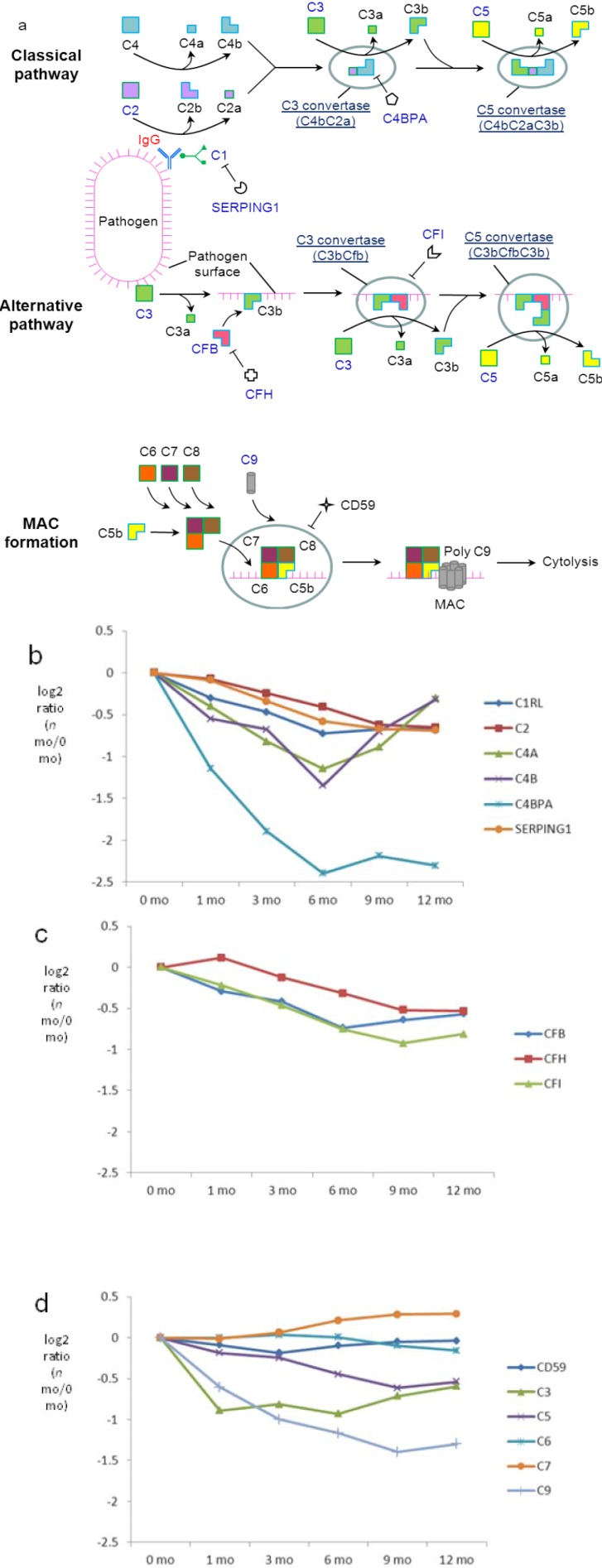
Quantitative analysis of complement components in milk. (**a**) Schematic representation of the KEGG complement components found in milk. Relative-abundance distribution of complement components; (**b**) specific to the classical pathway; (**c**) specific to the alternative pathway; (**d**) common to both. Significance *p*-values are available in Supplementary Figure S6. Standard errors for each of the proteins are available in Supplementary Figure S8.

One important class of APPs are those involved in complement activation through classical or alternative pathways (Figure 7a). In the classical pathway, the complement system works in conjunction primarily with IgG, which recognizes the surface of pathogens. In the alternative pathway, the microbial surfaces directly activate the complement system and the presence of antibodies is not required [58]. Our previous analysis suggested that complement components specific to the alternative pathway are present in human milk at higher abundances than those specific to the classical pathway [26]. Nevertheless, regulation of the milk complement system over the course of lactation has remained unclear. In the present study, we provide a more complete view of the changes in the milk complement system during the first 12 months of lactation. 

The levels of classical-pathway-specific component C1RL, a subunit of C1, and its inhibitor, SERPING1, are relatively stable with modest decreases during late lactation (Figure 7b). A similar regulatory pattern was observed for the downstream component C2, which is activated by C1 during the cascade of the classical pathway. In contrast, levels of C4a/b decrease during the first 6 months but rise again to 12 months of lactation. Changes in the protein abundance of C4a/b imply active regulation of the milk classical pathway, which would probably function throughout the course of lactation. Among various antibody classes, IgG is the strongest mediator of the classical pathway. Although it is not clear which factors regulate the increased conversion of C4a/b from C4 after 6 months, the steady increase in the abundance of IgG (Figure 6a) may provide a means to retain the presence and function of the classical pathway via increased formation of the pathogen-IgG-C1 complex (Figure 7a), which activates the cascade that eventually results in higher C4a/b abundance after 6 months. C4b is used in the classical pathway to form C3 convertase, which propagates the pathway by catalyzing conversion of C3 into C3a and C3b. Indeed, following an initial decrease in the first month, levels of C3 increase after 6 months (significance *p*-value (12 mo *versus* 6 mo) < 0.01). Interestingly, significant decreases in protein abundance were found for C4BPA, which inhibits C4. Collectively, the higher abundance of IgG, C3 and C4, as well as the lower abundance of C4BPA at later lactation may provide a mechanism to retain the function of the classical pathway in milk. 

Among proteins unique to the alternative pathway, levels of CFB, CFH and CFI are slightly downregulated (Figure 7c), suggesting a modest decrease in the extent of alternative pathway function over lactation. Several quantified proteins are common to both alternative and classical pathways, including C3, which in addition to its classical pathway functionality initiates the alternative pathway by interacting with microbial surfaces. Both classical and alternative pathways lead to the formation of C5, which plays a central role in complement cascade by recruiting C9 onto microbial surfaces to form the membrane attack complex (MAC) during cytolysis. While the levels of CD59, which prevent the binding of C5–C9 to the cell surface, remain stable over lactation, a modest but consistent decrease was observed for C5 and C9 (Figure 7d; significance *p*-values (12-mo *versus* 0 mo) 2.6E−7 and 9.9E−28, respectively, in Supplementary Figure S6). The observation suggests an overall decrease in the classical and alternative complement cascades in milk. 

In summary, the quantitative analysis of the lactation changes of complement components suggests the presence of both the classical and the alternative pathway in milk. Increasing IgG levels over lactation may contribute to retention of the functionality of the classical pathway. The alternative pathway likely plays a predominant role compared to the classical pathway and, overall, this study suggests that the contribution of anti-pathogenic complement system proteins made by healthy women to milk decreases over the course of 12 months of lactation. 

One major consideration in conducting proteomic studies of human milk is that apparently healthy donors may have unidentified sources of inflammation during feeding that are not realized. In this case, the immune factors may not truly reflect the health status of breastfeeding women. However, the consistency in the decreases of MAC-forming C9 and other APPs over lactation instead suggest that this change is physiological rather than a reflection of healthiness of the donors that were explored in the present study. While the system of the alternative pathway is relatively stable with a modest decrease over lactation, the complement cascade through the classical pathway may undergo significant changes over lactation. The significant transition in complement system at 6 months took place at the same period as sIgA, again coinciding with the life event of solid food introduction. 

## 4. Discussion

This work represents the first in-depth quantitative study of the human milk proteome during the first 12 months of lactation, based on the premise that a better understanding of human lactogenesis and infant health can be achieved in a non-invasive manner from the readouts of milk secretion. Many factors, including maternal nutrition, genetic variation, health, and environment may affect milk composition and functions therein. Our first attempt here was to delineate the changes in milk composition over time. To separate other factors from the temporal effect, we collected milk from 10 donors, each contributing to samples at multiple stages of lactation from week 1 to 12 months. The TMT labeling and the extensive sample prefractionation using ERLIC allowed us to measure the temporal changes in milk proteins in depth, with 615 proteins quantified across 6 different stages of lactation. 

Conventionally, milk produced in the first 2 weeks after childbirth is considered transitional milk as milk transitions from colostrum to milk, while milk produced after 2 weeks tends to be more stable and is generally considered mature. The current study, however, highlights that protein composition of “mature milk” is not fixed, but continues to transition throughout the first year of lactation (Figure 2d). It is worth noting further that protein expression varies between individuals as well as over time (Figure 2d). Indeed, among the 615 proteins quantified, 509 proteins showed significant changes with respect to the subject factor (FDR-adjusted *p* < 0.01, Dataset 2), highlighting the complexity of milk secretion in which multiple parameters play a role. Detailed functional analyses of the proteins with more conserved expression among subjects may provide additional insights into the evolutionary development of milk, and this topic will be discussed further.

A few transforming pathways during lactogenesis were newly identified from this work. We have highlighted lipid/carbohydrate metabolism, glycoprotein metabolism, cellular detoxification processes and immune processes in the Results section. Previous work on lipid metabolism using stable isotope tracer methodology showed that human milk FAs are derived from either blood LDs via dietary uptake or *de novo* mammary synthesis [59]. Several factors, including dietary composition, total calories, fasting/starvation status and body fat content, were found to lead to large variations in the short-term FA compositions of milk [60,61], which may be superimposed onto the long-term temporal trend. Direct measurement of milk FA content may make it difficult to assess the general trend of FA metabolism over the course of lactation. However, the distinct but consistent changes in the abundance of milk proteins involved in extracellular and intracellular FA metabolism (Figure 3), indeed, suggest that the underlying mammary cellular machinery is operating in ways that are distinct from ephemeral factors like diet. It has been well documented that higher carbohydrate intakes are associated with increased FA *de novo* synthesis [45]. Interestingly, inversed correlations were found between dietary fat intakes and *de novo* mammary FA lipogenesis [45]. These observations are consistent with our current findings of downregulation of proteins involved in dietary FA intake and upregulation in both carbohydrate metabolism and *de novo* lipogenesis over lactation (Figure 3). 

Much thought has been given to fine-tuning milk composition in both breastfeeding women and lactating farm species through diet [1]. Our findings in transitioning mammary FA metabolism suggest that maternal dietary intake may contribute more to the milk FA composition in early lactation as opposed to late lactation. Such improved understanding of human milk can be applied to guide the feeding process when fresh mother’s milk is not available. Aside from human milk, cow’s milk has received particular attention because it is a major human food and agro-economic product. Bovine fat production and FA milk profiles can be affected by stage of lactation and nutrition provided [62,63,64]. The transitioning FA metabolism identified in this work may be used to guide such efforts. 

It was previously found that the total concentration of human milk oligosaccharides (HMOs) decreased during the first 12 months of lactation with larger and more complex species prevailing in early lactation [65]. Similarly, the total concentration of human milk glycoproteins decreased during the first 28 weeks of lactation [66]. Although an enzyme’s expression may not be equal to its catalytic activity, the decreases in both total HMO abundance/complexity and glycoprotein abundance can be explained by our findings of increased protein expression in glycan degradation and decreased protein expression in glycoprotein synthesis over lactation. HMOs may act as decoys to prevent pathogen binding to intestinal epithelial cells and to enrich beneficial microbiota during infant growth [67,68]. The preference of mammary epithelial cells in releasing more complex HMOs during early lactation highlights the importance of larger HMOs during early infant development. 

We further note that the composition of milk is capable of being adjusted in response to life events. In the example of milk sIgA, levels decrease during the first 6 months of postnatal life and rise again after 6 months. Changes in the levels of milk sIgA appear to be in accordance with the life events of birth and solid food introduction, during which both the infant would benefit from greater protection against increased pathogenic exposure. The expressional changes of milk IgG provide another example of synchronous association between milk composition and fetal and postnatal life. While infants are born with high levels of circulating maternal IgG via transplacental transfer, production of the infant’s own IgG is delayed and leads to a decreased abundance in IgG throughout the first year of life. Levels of milk IgG, however, increase consistently throughout the first year of lactation, which is complementary to the need for IgG during the early life of infants when they experience a transient deficiency. IgG binds to antigens that may be found in the intestinal lumen and transfers them across the epithelial layer [50]. It is not certain whether the increased supplies of milk IgG would meet the entire need for growing infants. Given that the infant’s GI tract is relatively immature and more vulnerable to pathogenic infections, the milk IgG so transferred may protect primarily the basal propria underlying the GI epithelium from pathogenic infection during early infancy. 

The proteomes of nonlactating human mammary epithelial cells were explored previously both *in vivo* and *in vitro* [69,70]. We compared the 1,579 milk proteins to the 1,574 proteins in human mammary epithelial cells [70] (Supplementary Table S2). The two proteome sets have approximate numbers of identified proteins where only 435 proteins were found in common between the two proteome sets. We further compared the milk proteome to a set of 889 extracellular-space proteins found in the media of phorbol ester (PMA)-stimulated nonlactating human mammary epithelial cells [71]. 429 proteins were found in common between the two proteome sets. The relatively small overlaps can be, in part, ascribed to the complementarity between different proteomic setups and the complex origins of milk proteome (Figure 1a). GO analysis showed that nuclear and mitochondrial proteins are overrepresented in the mammary epithelial cells compared to the extracellular space of mammary epithelial cells and milk (Supplementary Table S2; nucleus: 35.9%, 29.8% and 21.0%, respectively, for mammary epithelial cells, extracellular space of mammary epithelial cells and milk; mitochondrion: 16.7%, 4.3% and 6.3%, respectively, for mammary epithelial cells, extracellular space of mammary epithelial cells and milk). These differences are consistent with the biological understanding that local nuclear and mitochondrial proteins are less likely secreted. Conversely, the proteome representation of Golgi apparatus is least abundant in the mammary epithelial cells (4.8%, 8.6% and 9.2%, respectively, for mammary epithelial cells, extracellular space of mammary epithelial cells and milk). Golgi is a central cellular organelle during vesicle formation and secretion. Its overrepresentation in milk and extracellular space of mammary epithelial cells is indicative of higher secretory activities during lactation and under PMA stimulation. Secretion induced by PMA seems not to affect the GO representation of ER proteins between the mammary epithelial cells and its extracellular space (6.8%, 6.9% and 8.0%, respectively, for mammary epithelial cells, extracellular space of mammary epithelial cells and milk). The modest GO enrichment of ER proteins for milk, however, is consistent with a large production of proteins and fats in ER during lactation. 

Low levels of proteins from cell debris might contribute to the milk proteome as well. Similar to the proteome of extracellular space of mammary epithelial cells, we identified in milk a number of ribosomal and proteasomal proteins that are typically thought to be nonsecreted. The GO comparison further showed a lower representation of nuclear proteins in milk than in the extracellular space of mammary epithelial cells (21.0% *versus* 29.8%). The lower occurrence of nuclear proteins in milk may be reflective to a lesser extent of cell degranulation/detachment from the lactating mammary gland as opposed to the cultured cell dishes. 

## 5. Summary and Future Perspectives

The non-invasive approach to the in-depth determination of protein contents in milk whey followed by bioinformatic analysis illustrates its large potential in advancing our understanding of milk and the lactating mammary gland. In addition to the whey fraction, milk contains fat droplets in the form of milk-fat-globule membrane (MFGM). Albeit a number of membrane-bound and endosomal proteins were identified in milk whey, more of them may present in MFGM. Many membranes proteins are involved in various para-/trans-cellular and intra-/inter-cellular processes. In-depth exploration of the temporal changes of the MFGM proteome would likely provide more insights into these biological processes during lactation. In addition to looking at other fractions of milk, systems biology that integrates proteomic, transcriptomic, metabolomic, lipidomic, glycomic and peptidomic data with bioinformatic analysis creates a system-wide view of a given biological entity. More insights and molecular mechanisms underlying many of the biological properties of milk will be revealed by combining proteomics with other -omics analysis. Milk is conventionally considered as the gold standard for the purpose of feeding infants. In addition to lactation time, many more variables such as maternal nutrition, genetic variation, age, health, environment etc., may affect milk compositions and functions therein. With the high diversity of milk, -omics studies that differentiate these factors would have high propensity to lead to fruitful outcomes separating the significance of the various aspects. A clearer understanding of milk and the lactating mammary gland would guide our efforts in improving human health and wellness beginning in early life.

## Supplementary Materials

Supplementary File 1
