# Supplementary material for: Quantitative Analysis of the Human Milk Whey Proteome Reveals Developing Milk and Mammary-Gland Functions across the First Year of Lactation
Source: Proteomes. 2013 Sep 3;1(2):128–58. doi: 10.3390/proteomes1020128 (PMC5302745; doi:10.3390/proteomes1020128)
Supplement: Supplementary File 1 [file proteomes-01-00128-s001.zip › proteomes-38507-Supplementary-final.pdf]

Supplementary Materials

Figure S1. (**A**) Typical ERLIC chromatogram of TMT-labeled human-milk tryptic peptides. Milk whey proteins were digested and pooled from transitional milk (0 *month*) and mature milk (1, 3, 6, 9 and 12 *month*) and loaded in 200 µL volumes containing 600 µg peptides. Detection: A_{280}. Gradient: See Experimental Section; (**B**) Example of peptides fractionation using ERLIC. Blue: number of identified peptides in each fraction. Red: number of peptides overlapping with previous fraction.

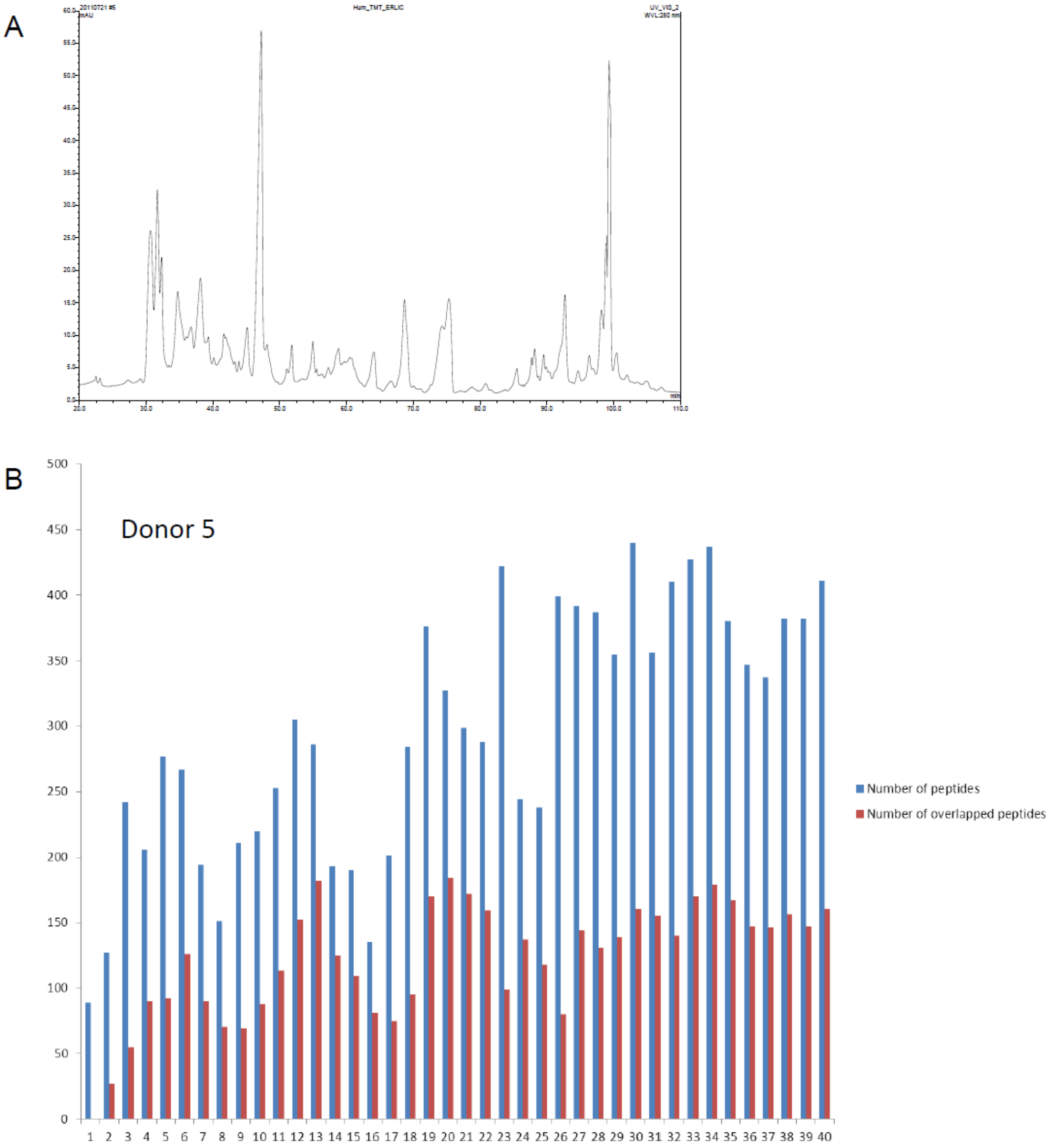

Figure S2. Accuracy in protein quantification. Left panel: scatter plot of log_2 fold changes between two technical replicates at 1, 3, 6, 9 and 12 *month* relative to 0 *month* obtained from donors 4 and 9. Ratios from x-axis were obtained from technical replicate one and y-axis from replicate two. Right panel: histograms of log_2 fold changes between two technical replicates from donor 4 and 9 at 1, 3, 6, 9 and 12 *month* relative to 0 *month*. Box plots indicate the variation of protein ratios. The vertical line indicates the median, and the whisker caps indicate the lower 10th and upper 90th percentiles.

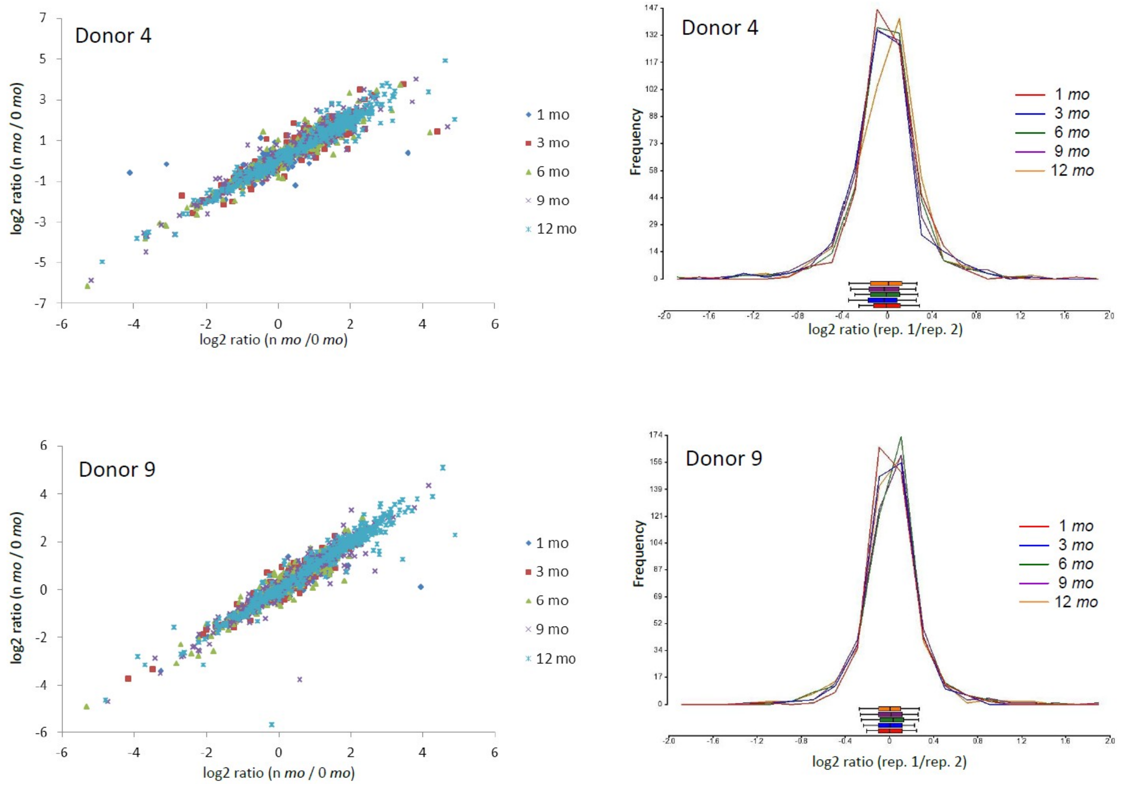

Figure S3. Hierarchical clustering analysis of (**A**) 615 milk proteins and (**B**) a subset of 213 glycoproteins (from Gene Ontology classification) quantified in this work. Protein abundances at n *month* (n = 1, 3, 6, 9 and 12) are compared to 0 *month* (1 *week*) of lactation. Color legends are at log_2 scale.

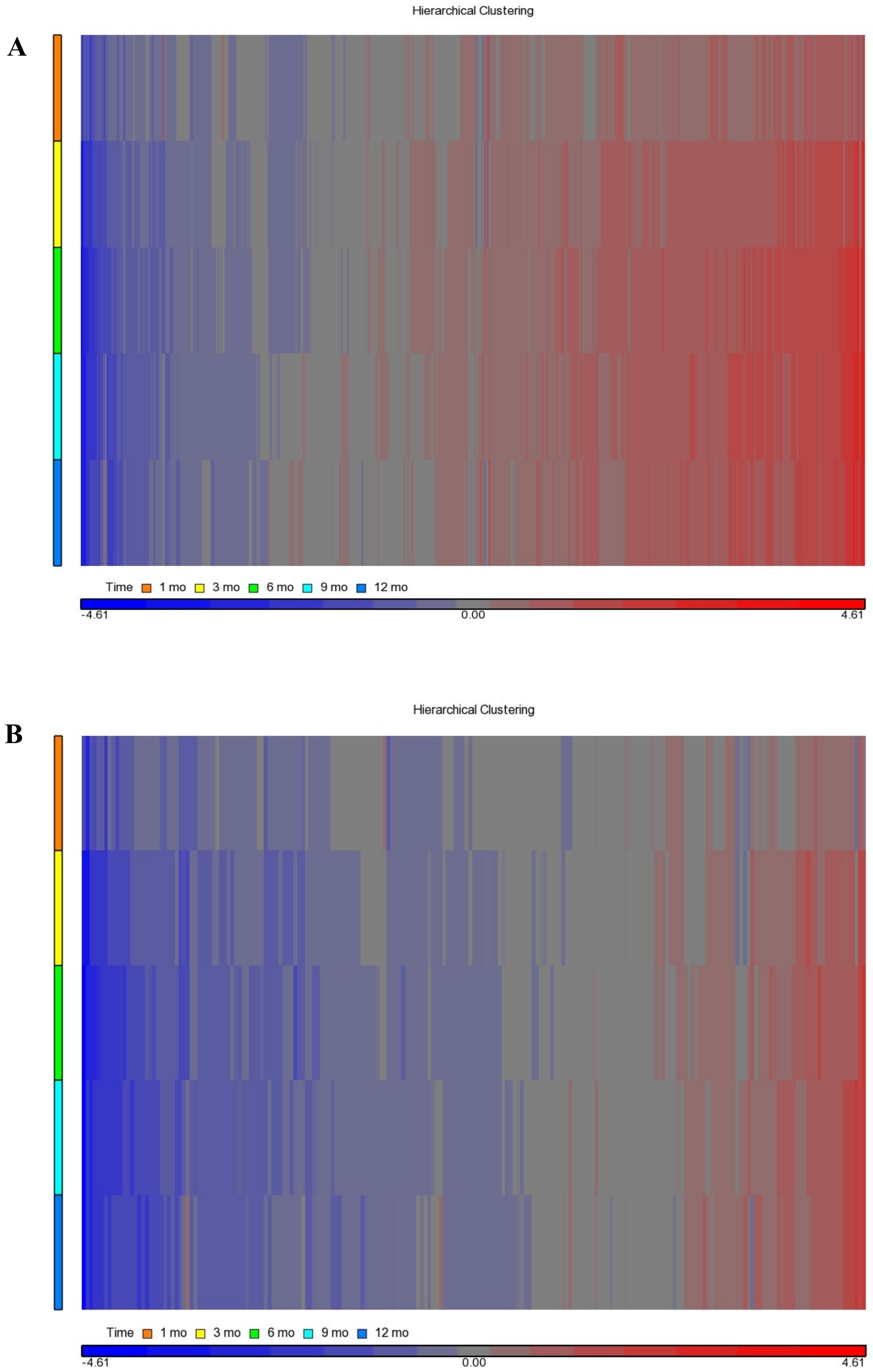

Figure S4. Venn diagram. (**A**) Protein downregulated at 1–12 *month* relative to 0 *month* with fold change greater or equal to 1.3; (**B**) Protein upregulated at 1–12 *month* relative to 0 *month* with change greater or equal to 1.3-fold.

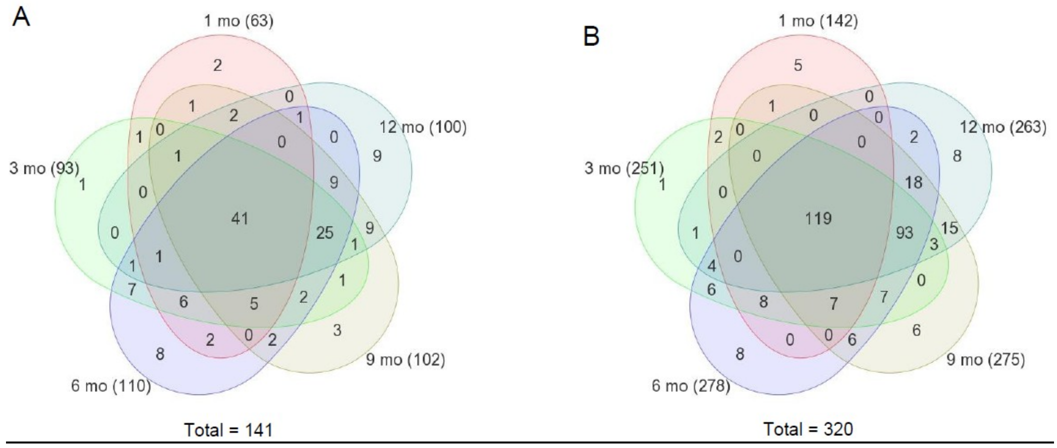

Figure S5. Schematic representation of the KEGG pathway of nucleotide sugar metabolism. Proteins upregulated over lactation are in red.

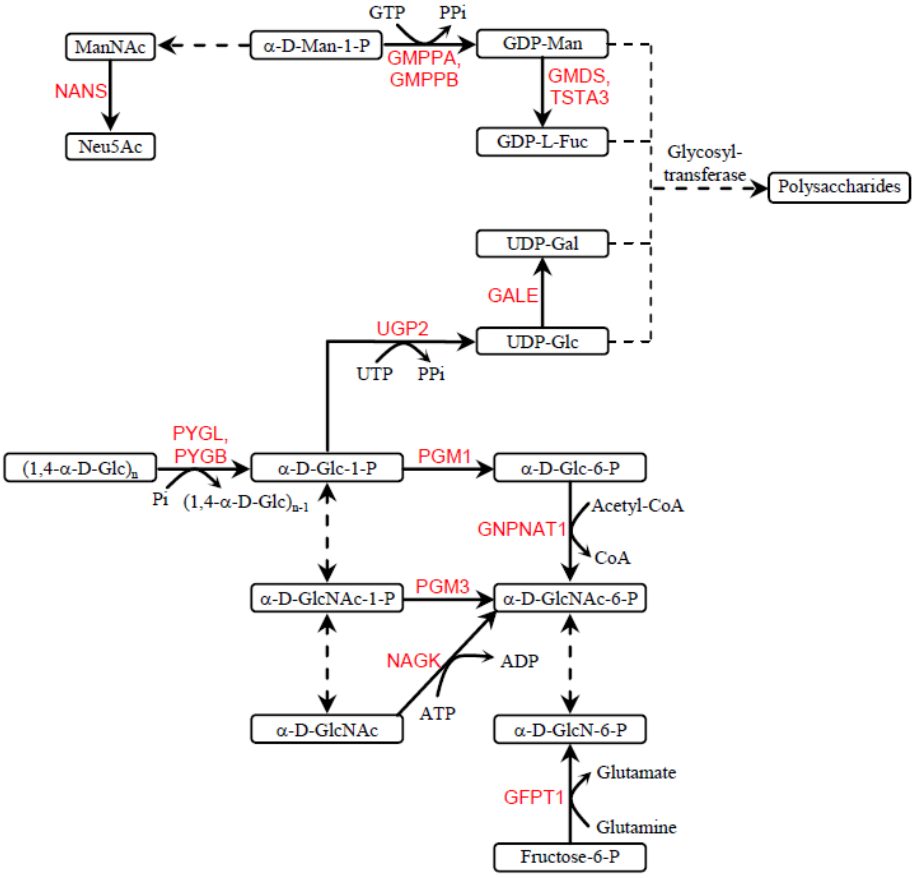

Figure S6. Hierarchical clustering of proteins involved in acute-phase response. Color legends are at $\log_2$ scale.

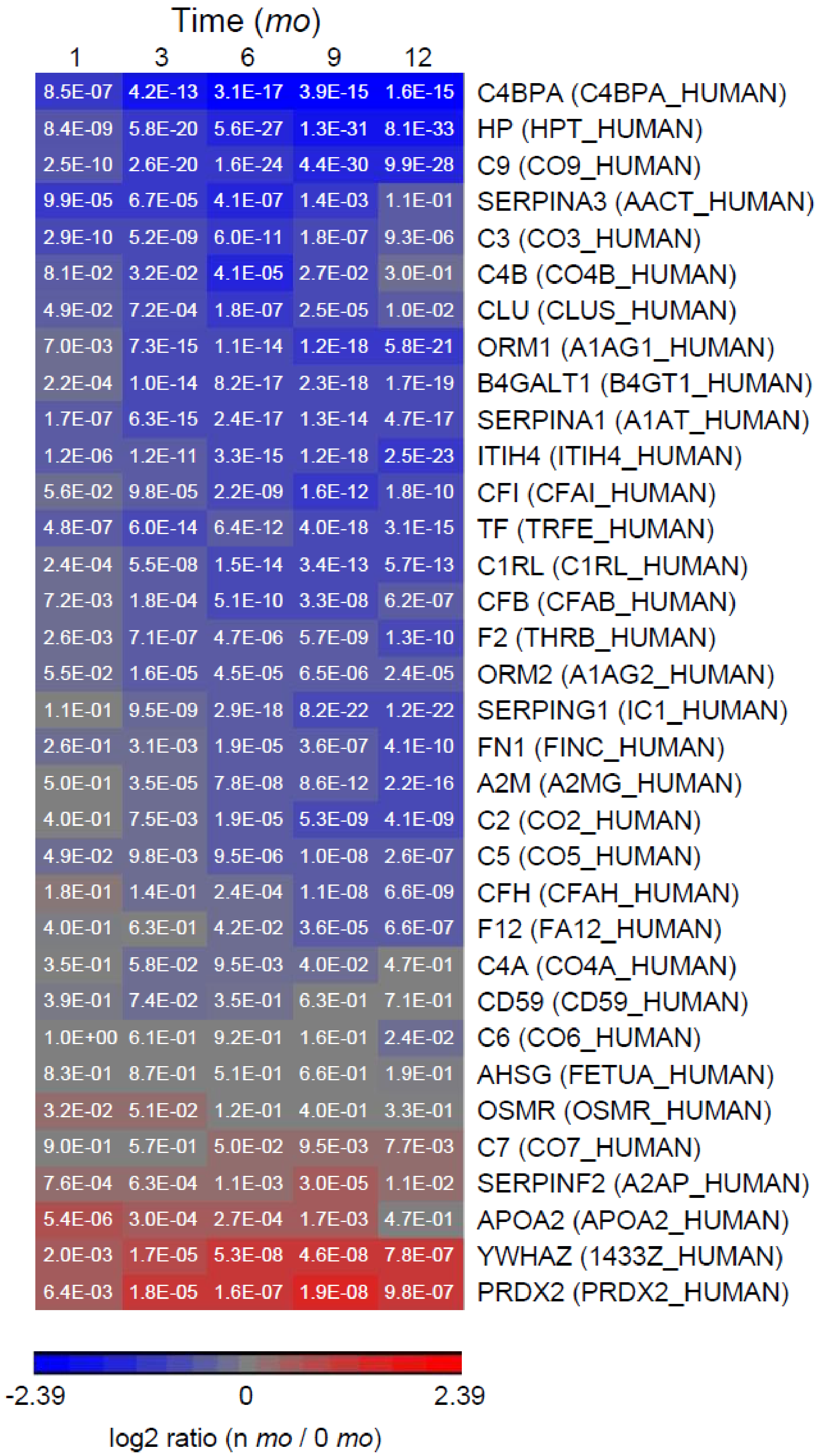

Figure S7. (**A**) Relative-abundance distributions of IgG (IGHG1, IGHG2, IGHG3 and IGHG4), sIgA (IGHA1, IGHA2 and PIGR) and IgM (IGHM) with standard error bars shown; (**B**) Relative-abundance distributions of Ig lambda (IGLL5) and kappa (IGKC) classes with standard error bars shown.

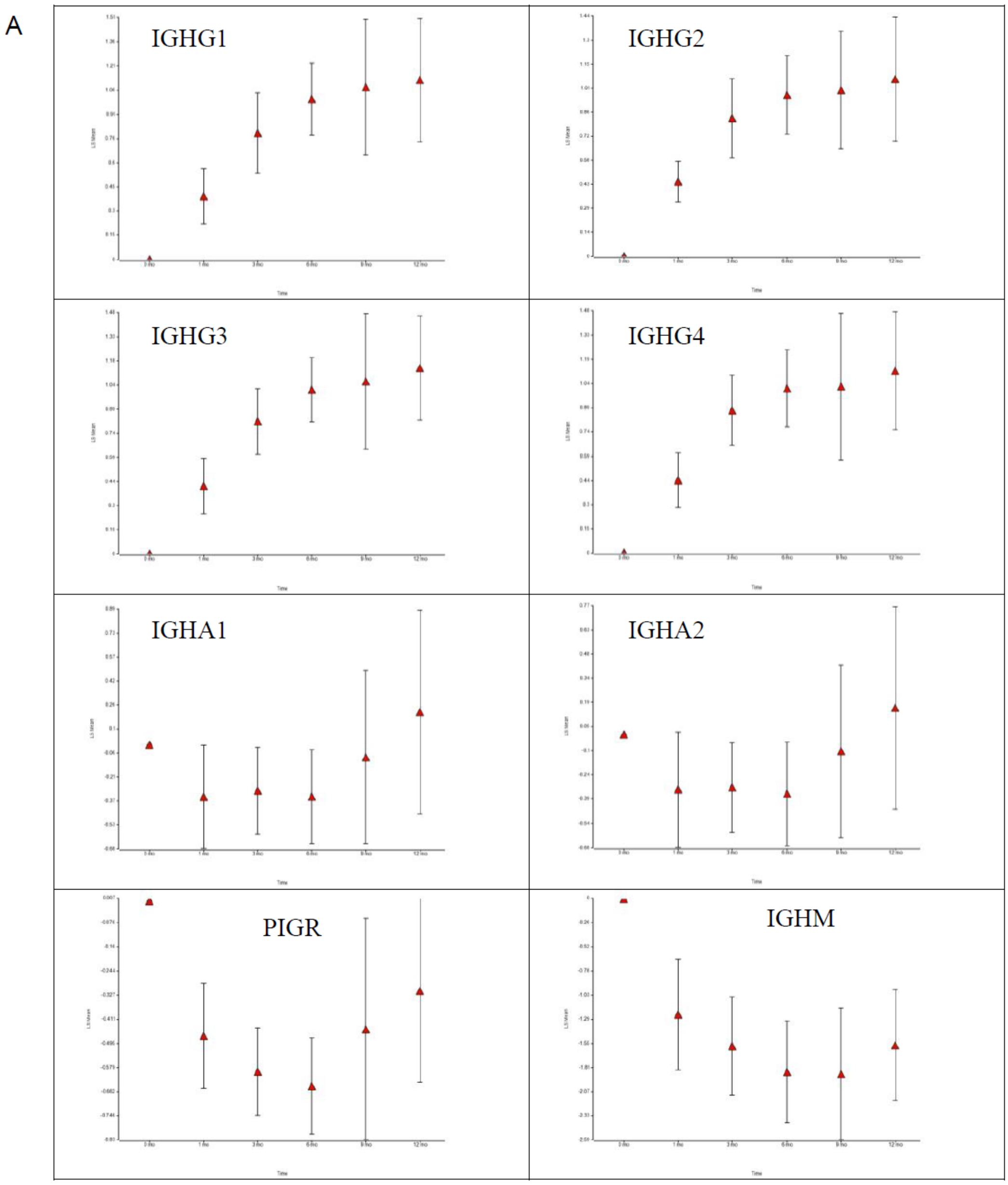

Figure S7. *Cont.*

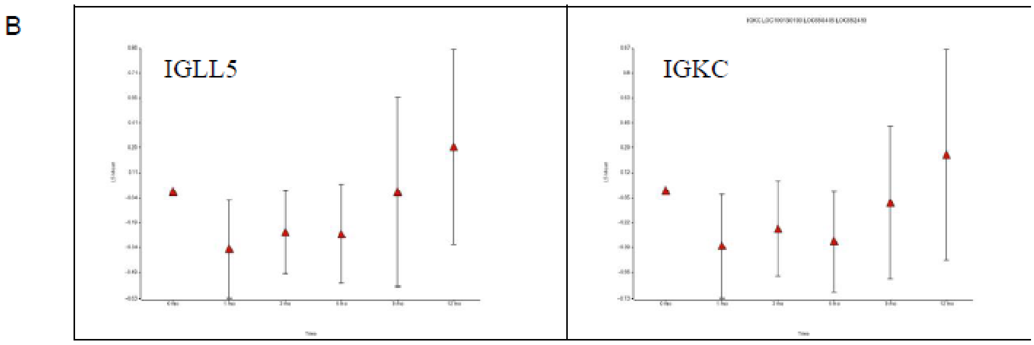

Figure S8. Relative-abundance distribution of complement components (**A**) specific to the classical pathway; (**B**) specific to the alternative pathway and (**C**) common to both with standard error bars shown.

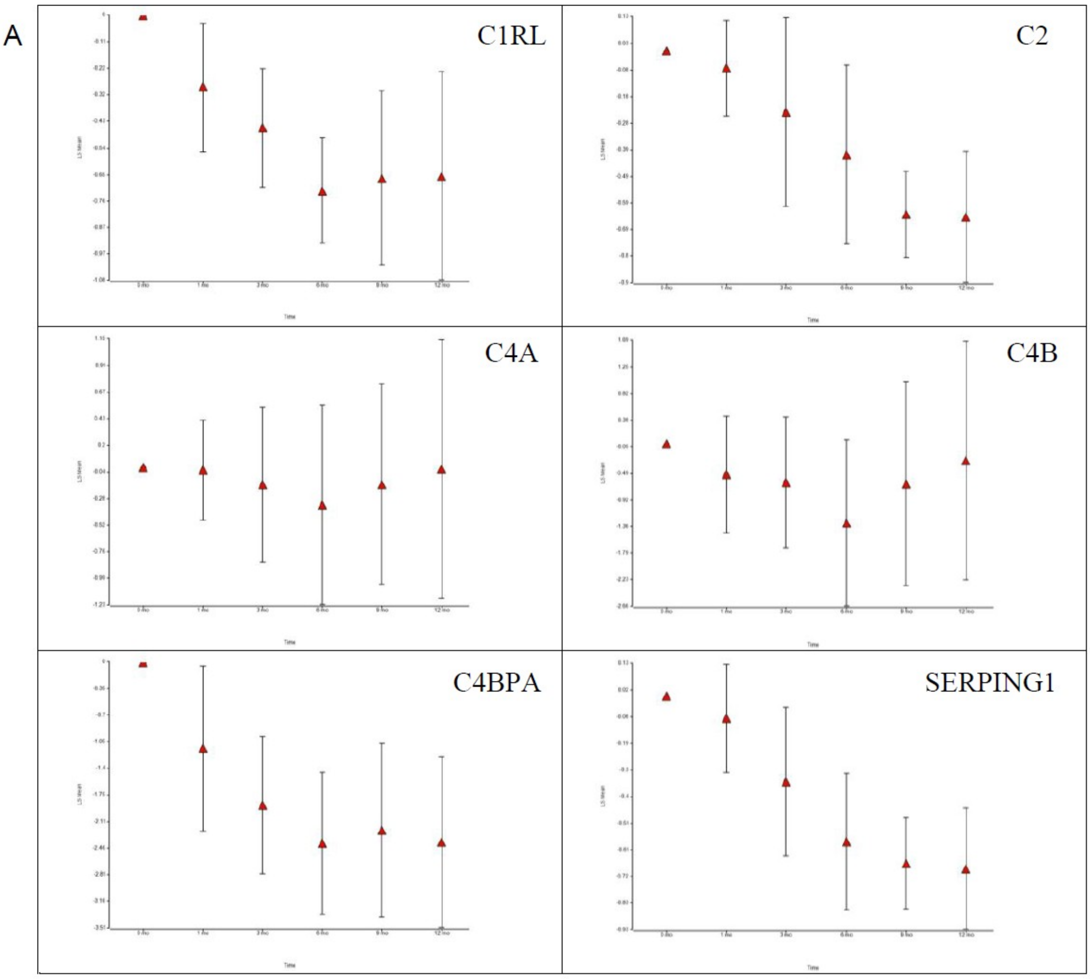

Figure S8. *Cont.*

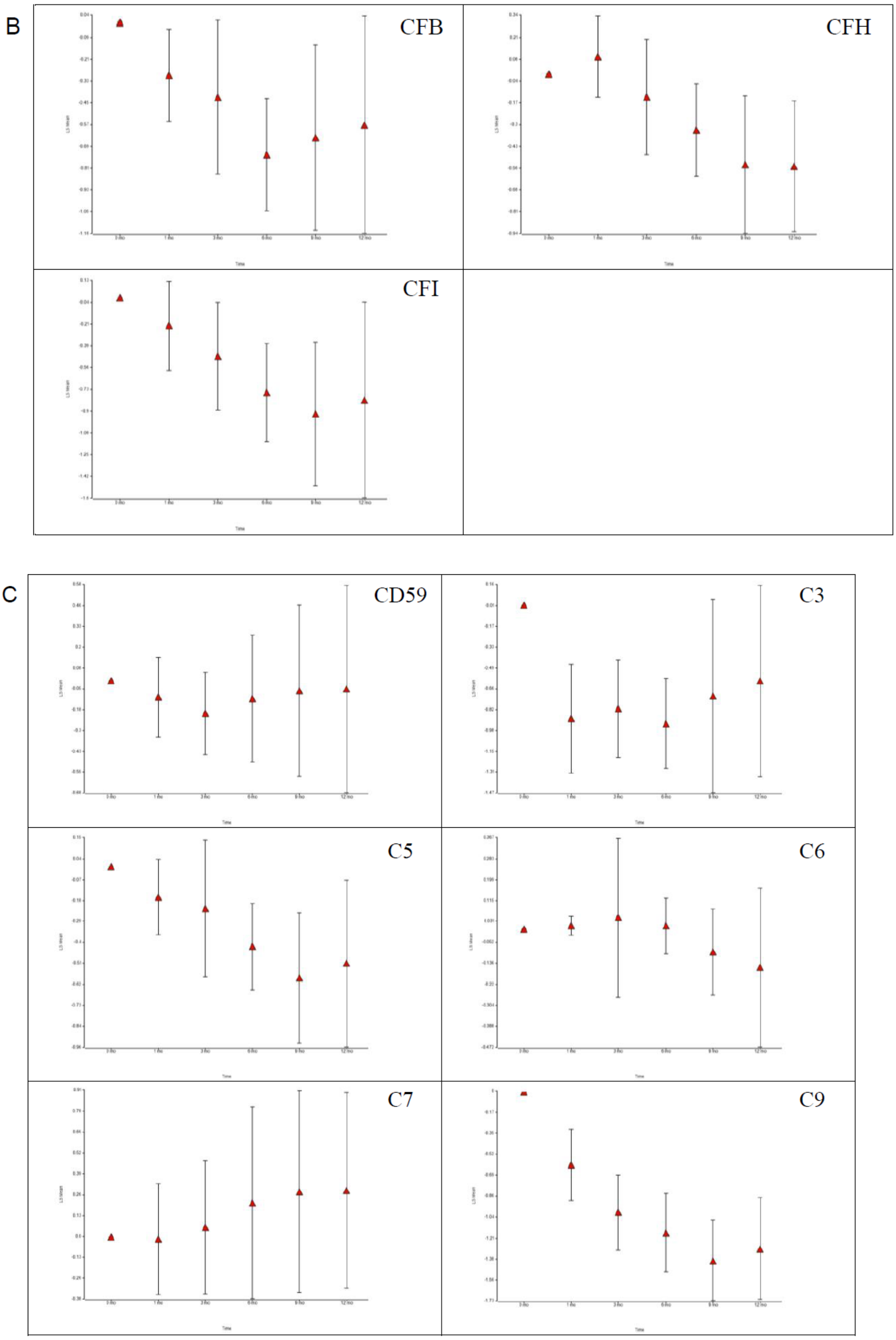

Table S1. Linear correlation coefficients for each of the protein abundance ratios n-*mo*/0-*mo* (n = 1, 3, 6, 9 and 12) between two repeated LC-MS runs.

Donor ID.	1 *mo*	3 *mo*	6 *mo*	9 *mo*	12 *mo*
1	0.87	0.90	0.95	0.96	0.95
2	0.96	0.95	0.98	0.97	0.95
3	0.94	0.95	0.92	0.97	0.91
4	0.80	0.87	0.94	0.91	0.91
5	0.70	0.94	0.95	0.96	0.97
6	0.91	0.88	0.91	0.87	0.86
7	0.94	0.96	0.96	0.94	0.95
8	0.94	0.94	0.96	0.97	0.97
9	0.93	0.96	0.96	0.96	0.96
10	0.96	0.98	0.97	0.97	0.97

Table S2. Gene ontology comparison of the proteomes of human mammary epithelial cells (Ref. 70), extracellular space proteins of human mammary epithelial cells under phorbol ester stimulation (Ref. 71) and milk (this work).

	Human mammary epithelial cell	***Extracellular space of human mammary epithelial cell***	***Milk***
Number of proteins	1,574	889	1,579
Nucleus	35.9%	29.8%	21.0%
Mitochondrion	16.7%	4.3%	6.3%
Endoplasmic reticulum	6.8%	6.9%	8.0%
Golgi apparatus	4.8%	8.6%	9.2%
