# Supplementary material for: Quantitative Analysis of the Human Milk Whey Proteome Reveals Developing Milk and Mammary-Gland Functions across the First Year of Lactation
Source: Proteomes. 2013 Sep 3;1(2):128–58. doi: 10.3390/proteomes1020128 (PMC5302745; doi:10.3390/proteomes1020128)
Supplement: Supplementary File 1 [file proteomes-01-00128-s001.zip › Supplementary dataset 1.pdf]

Supplementary Dataset 1. List of human milk whey proteins using electrostatic repulsion-hydrophilic interaction chromatography (ERLIC).

#	Proteins	Uniprot ID	Molecular Weight	Number of unique peptides	Percent of coverage
1	14-3-3 protein beta/alpha OS=Homo sapiens GN=YWHAB PE=1 SV=3	1433B_HUMAN	28 kDa	6	52%
2	14-3-3 protein epsilon OS=Homo sapiens GN=YWHAE PE=1 SV=1	1433E_HUMAN	29 kDa	20	74%
3	14-3-3 protein eta OS=Homo sapiens GN=YWHAH PE=1 SV=4	1433F_HUMAN	28 kDa	11	46%
4	14-3-3 protein gamma OS=Homo sapiens GN=YWHAG PE=1 SV=2	1433G_HUMAN	28 kDa	13	60%
5	14-3-3 protein sigma OS=Homo sapiens GN=SFN PE=1 SV=1	1433S_HUMAN	28 kDa	11	52%
6	14-3-3 protein theta OS=Homo sapiens GN=YWHAQ PE=1 SV=1	1433T_HUMAN	28 kDa	7	48%
7	14-3-3 protein zeta/delta OS=Homo sapiens GN=YWHAZ PE=1 SV=1	1433Z_HUMAN	28 kDa	15	63%
8	HLA class I histocompatibility antigen, A-3 alpha chain OS=Homo sapiens GN=HLA-A PE=1 SV=2	1A03_HUMAN	41 kDa	2	24%
9	HLA class I histocompatibility antigen, A-23 alpha chain OS=Homo sapiens GN=HLA-A PE=1 SV=1	1A23_HUMAN (+1)	41 kDa	2	20%
10	HLA class I histocompatibility antigen, A-69 alpha chain OS=Homo sapiens GN=HLA-A PE=1 SV=2	1A69_HUMAN	41 kDa	5	20%
11	HLA class I histocompatibility antigen, B-35 alpha chain OS=Homo sapiens GN=HLA-B PE=1 SV=1	1B35_HUMAN (+1)	40 kDa	4	24%
12	HLA class I histocompatibility antigen, B-49 alpha chain OS=Homo sapiens GN=HLA-B PE=2 SV=2	1B49_HUMAN (+1)	41 kDa	2	19%
13	HLA class I histocompatibility antigen, Cw-7 alpha chain OS=Homo sapiens GN=HLA-C PE=1 SV=3	1C07_HUMAN	41 kDa	5	26%
14	Serine/threonine-protein phosphatase 2A 65 kDa regulatory subunit A alpha isoform OS=Homo sapiens GN=PPP2R1A PE=1 SV=4	2AAA_HUMAN	65 kDa	17	34%
15	HLA class II histocompatibility antigen, DRB1-13 beta chain OS=Homo sapiens GN=HLA-DRB1 PE=1 SV=1	2B1D_HUMAN	30 kDa	2	9.40%
16	3-hydroxyisobutyrate dehydrogenase, mitochondrial OS=Homo sapiens GN=HIBADH PE=1 SV=2	3HIDH_HUMAN	35 kDa	4	20%
17	4F2 cell-surface antigen heavy chain OS=Homo sapiens GN=SLC3A2 PE=1 SV=3	4F2_HUMAN	68 kDa	11	20%
18	Cytosolic purine 5'-nucleotidase OS=Homo sapiens GN=NT5C2 PE=1 SV=1	5NTC_HUMAN	65 kDa	2	5.50%
19	6-phosphogluconate dehydrogenase, decarboxylating OS=Homo sapiens GN=PGD PE=1 SV=3	6PGD_HUMAN	53 kDa	20	53%
20	6-phosphogluconolactonase OS=Homo sapiens GN=PGLS PE=1 SV=2	6PGL_HUMAN	28 kDa	6	29%
21	Alpha-1-acid glycoprotein 1 OS=Homo sapiens GN=ORM1 PE=1 SV=1	A1AG1_HUMAN	24 kDa	8	41%
22	Alpha-1-acid glycoprotein 2 OS=Homo sapiens GN=ORM2 PE=1 SV=2	A1AG2_HUMAN	24 kDa	6	40%
23	Alpha-1-antitrypsin OS=Homo sapiens GN=SERPINA1 PE=1 SV=3	A1AT_HUMAN	47 kDa	29	64%
24	Alpha-1B-glycoprotein OS=Homo sapiens GN=A1BG PE=1 SV=4	A1BG_HUMAN	54 kDa	14	42%
25	Alpha-2-antiplasmin OS=Homo sapiens GN=SERPINF2 PE=1 SV=3	A2AP_HUMAN	55 kDa	15	41%
26	Leucine-rich alpha-2-glycoprotein OS=Homo sapiens GN=LRG1 PE=1 SV=2	A2GL_HUMAN	38 kDa	14	54%
27	Alpha-2-macroglobulin OS=Homo sapiens GN=A2M PE=1 SV=3	A2MG_HUMAN	163 kDa	52	48%
28	Amyloid beta A4 protein OS=Homo sapiens GN=APP PE=1 SV=3	A4_HUMAN	87 kDa	21	24%
29	Neutral amino acid transporter B(0) OS=Homo sapiens GN=SLC1A5 PE=1 SV=2	AAAT_HUMAN	57 kDa	4	8.30%
30	Acetoacetyl-CoA synthetase OS=Homo sapiens GN=AACS PE=1 SV=1	AACS_HUMAN	75 kDa	3	4.80%
31	Alpha-1-antichymotrypsin OS=Homo sapiens GN=SERPINA3 PE=1 SV=2	AACT_HUMAN	48 kDa	26	62%
32	5'-AMP-activated protein kinase subunit gamma-1 OS=Homo sapiens GN=PRKAG1 PE=1 SV=1	AAKG1_HUMAN	38 kDa	3	13%
33	Aspartate aminotransferase, cytoplasmic OS=Homo sapiens GN=GOT1 PE=1 SV=3	AATC_HUMAN	46 kDa	12	40%
34	ATP-binding cassette sub-family B member 5 OS=Homo sapiens GN=ABCB5 PE=1 SV=3	ABCB5_HUMAN	90 kDa	2	2.10%
35	ATP-binding cassette sub-family E member 1 OS=Homo sapiens GN=ABCE1 PE=1 SV=1	ABCE1_HUMAN	67 kDa	3	5.70%
36	ATP-binding cassette sub-family G member 2 OS=Homo sapiens GN=ABCG2 PE=1 SV=3	ABCG2_HUMAN	72 kDa	8	16%
37	Abhydrolase domain-containing protein 14B OS=Homo sapiens GN=ABHD14B PE=1 SV=1	ABHEB_HUMAN	22 kDa	7	41%
38	Acetyl-CoA carboxylase 2 OS=Homo sapiens GN=ACACB PE=1 SV=3	ACACB_HUMAN	277 kDa	10	4.70%

#	Proteins	Uniprot ID	Molecular Weight	Number of unique peptides	Percent of coverage
39	Acyl-CoA-binding protein OS=Homo sapiens GN=DBI PE=1 SV=2	ACBP_HUMAN	10 kDa	5	61%
40	Angiotensin-converting enzyme OS=Homo sapiens GN=ACE PE=1 SV=1	ACE_HUMAN	150 kDa	15	14%
41	Angiotensin-converting enzyme 2 OS=Homo sapiens GN=ACE2 PE=1 SV=2	ACE2_HUMAN	92 kDa	13	20%
42	ATP-citrate synthase OS=Homo sapiens GN=ACLY PE=1 SV=3	ACLY_HUMAN	121 kDa	43	46%
43	Acyl-coenzyme A thioesterase 11 OS=Homo sapiens GN=ACOT11 PE=1 SV=1	ACO11_HUMAN	68 kDa	2	3.80%
44	Cytoplasmic aconitate hydratase OS=Homo sapiens GN=ACO1 PE=1 SV=3	ACOC_HUMAN	98 kDa	29	44%
45	Acyl-coenzyme A thioesterase 1 OS=Homo sapiens GN=ACOT1 PE=1 SV=1	ACOT1_HUMAN	46 kDa	4	10%
46	Acylamino-acid-releasing enzyme OS=Homo sapiens GN=APEH PE=1 SV=4	ACPH_HUMAN	81 kDa	11	18%
47	Acetyl-coenzyme A synthetase, cytoplasmic OS=Homo sapiens GN=ACSS2 PE=1 SV=1	ACSA_HUMAN	79 kDa	10	19%
48	Long-chain-fatty-acid--CoA ligase 1 OS=Homo sapiens GN=ACSL1 PE=1 SV=1	ACSL1_HUMAN	78 kDa	4	7.30%
49	Long-chain-fatty-acid--CoA ligase 3 OS=Homo sapiens GN=ACSL3 PE=1 SV=3	ACSL3_HUMAN	80 kDa	4	7.50%
50	Long-chain-fatty-acid--CoA ligase 4 OS=Homo sapiens GN=ACSL4 PE=1 SV=2	ACSL4_HUMAN	79 kDa	3	9.30%
51	Actin, cytoplasmic 1 OS=Homo sapiens GN=ACTB PE=1 SV=1	ACTB_HUMAN	42 kDa	22	63%
52	Beta-actin-like protein 2 OS=Homo sapiens GN=ACTBL2 PE=1 SV=2	ACTBL_HUMAN	42 kDa	3	18%
53	Actin, alpha cardiac muscle 1 OS=Homo sapiens GN=ACTC1 PE=1 SV=1	ACTC_HUMAN (+1)	42 kDa	2	31%
54	Actin, cytoplasmic 2 OS=Homo sapiens GN=ACTG1 PE=1 SV=1	ACTG_HUMAN	42 kDa	2	63%
55	Alpha-actinin-1 OS=Homo sapiens GN=ACTN1 PE=1 SV=2	ACTN1_HUMAN	103 kDa	21	48%
56	Alpha-actinin-4 OS=Homo sapiens GN=ACTN4 PE=1 SV=2	ACTN4_HUMAN	105 kDa	40	50%
57	Alpha-centractin OS=Homo sapiens GN=ACTR1A PE=1 SV=1	ACTZ_HUMAN	43 kDa	2	5.90%
58	Aminoacylase-1 OS=Homo sapiens GN=ACY1 PE=1 SV=1	ACY1_HUMAN	46 kDa	2	4.90%
59	Disintegrin and metalloproteinase domain-containing protein 10 OS=Homo sapiens GN=ADAM10 PE=1 SV=1	ADA10_HUMAN	84 kDa	6	12%
60	Disintegrin and metalloproteinase domain-containing protein 15 OS=Homo sapiens GN=ADAM15 PE=1 SV=4	ADA15_HUMAN	93 kDa	4	7.00%
61	Disintegrin and metalloproteinase domain-containing protein 9 OS=Homo sapiens GN=ADAM9 PE=1 SV=1	ADAM9_HUMAN	91 kDa	2	3.50%
62	ADAM DEC1 OS=Homo sapiens GN=ADAMDEC1 PE=1 SV=2	ADEC1_HUMAN	53 kDa	3	7.40%
63	Alcohol dehydrogenase class-3 OS=Homo sapiens GN=ADH5 PE=1 SV=4	ADHX_HUMAN	40 kDa	7	20%
64	Afadin OS=Homo sapiens GN=MLLT4 PE=1 SV=3	AFAD_HUMAN	207 kDa	2	0.82%
65	Afamin OS=Homo sapiens GN=AFM PE=1 SV=1	AFAM_HUMAN	69 kDa	13	22%
66	Phosphoacetylglucosamine mutase OS=Homo sapiens GN=PGM3 PE=1 SV=1	AGM1_HUMAN	60 kDa	8	13%
67	Anterior gradient protein 2 homolog OS=Homo sapiens GN=AGR2 PE=1 SV=1	AGR2_HUMAN	20 kDa	2	13%
68	Agrin OS=Homo sapiens GN=AGRN PE=1 SV=4	AGRIN_HUMAN	215 kDa	3	1.80%
69	Activator of 90 kDa heat shock protein ATPase homolog 1 OS=Homo sapiens GN=AHSA1 PE=1 SV=1	AHSA1_HUMAN	38 kDa	3	12%
70	Allograft inflammatory factor 1-like OS=Homo sapiens GN=AIF1L PE=1 SV=1	AIF1L_HUMAN	17 kDa	7	43%
71	Aminoacyl tRNA synthase complex-interacting multifunctional protein 1 OS=Homo sapiens GN=AIMP1 PE=1 SV=2	AIMP1_HUMAN	34 kDa	4	18%
72	Aminoacyl tRNA synthase complex-interacting multifunctional protein 2 OS=Homo sapiens GN=AIMP2 PE=1 SV=2	AIMP2_HUMAN	35 kDa	2	5.30%
73	Alcohol dehydrogenase [NADP+] OS=Homo sapiens GN=AKR1A1 PE=1 SV=3	AK1A1_HUMAN	37 kDa	22	79%
74	Aldo-keto reductase family 1 member C1 OS=Homo sapiens GN=AKR1C1 PE=1 SV=1	AK1C1_HUMAN	37 kDa	3	37%
75	Aldo-keto reductase family 1 member C3 OS=Homo sapiens GN=AKR1C3 PE=1 SV=4	AK1C3_HUMAN	37 kDa	11	42%
76	Retinal dehydrogenase 1 OS=Homo sapiens GN=ALDH1A1 PE=1 SV=2	AL1A1_HUMAN	55 kDa	24	49%
77	Aldehyde dehydrogenase family 1 member A3 OS=Homo sapiens GN=ALDH1A3 PE=1 SV=2	AL1A3_HUMAN	56 kDa	20	41%

#	Proteins	Uniprot ID	Molecular Weight	Number of unique peptides	Percent of coverage
78	Aldehyde dehydrogenase family 1 member L1 OS=Homo sapiens GN=ALDH1L1 PE=1 SV=2	AL1L1_HUMAN	99 kDa	44	55%
79	Alpha-aminoadipic semialdehyde dehydrogenase OS=Homo sapiens GN=ALDH7A1 PE=1 SV=5	AL7A1_HUMAN	58 kDa	17	39%
80	4-trimethylaminobutyraldehyde dehydrogenase OS=Homo sapiens GN=ALDH9A1 PE=1 SV=3	AL9A1_HUMAN	54 kDa	21	51%
81	Alanine aminotransferase 1 OS=Homo sapiens GN=GPT PE=1 SV=3	ALAT1_HUMAN	55 kDa	11	31%
82	Serum albumin OS=Homo sapiens GN=ALB PE=1 SV=2	ALBU_HUMAN	69 kDa	66	84%
83	Fructose-bisphosphate aldolase A OS=Homo sapiens GN=ALDOA PE=1 SV=2	ALDOA_HUMAN	39 kDa	31	80%
84	Fructose-bisphosphate aldolase C OS=Homo sapiens GN=ALDOC PE=1 SV=2	ALDOC_HUMAN	39 kDa	13	51%
85	Insulin-like growth factor-binding protein complex acid labile subunit OS=Homo sapiens GN=IGFALS PE=1 SV=1	ALS_HUMAN	66 kDa	5	12%
86	Protein AMBP OS=Homo sapiens GN=AMBP PE=1 SV=1	AMBP_HUMAN	39 kDa	11	41%
87	Peptidyl-glycine alpha-amidating monooxygenase OS=Homo sapiens GN=PAM PE=1 SV=2	AMD_HUMAN	108 kDa	4	4.20%
88	Aminopeptidase B OS=Homo sapiens GN=RNPEP PE=1 SV=2	AMPB_HUMAN	73 kDa	19	33%
89	Cytosol aminopeptidase OS=Homo sapiens GN=LAP3 PE=1 SV=3	AMPL_HUMAN	56 kDa	31	72%
90	Aminopeptidase N OS=Homo sapiens GN=ANPEP PE=1 SV=4	AMPN_HUMAN	110 kDa	5	5.90%
91	Alpha-2-macroglobulin receptor-associated protein OS=Homo sapiens GN=LRPAP1 PE=1 SV=1	AMRP_HUMAN	41 kDa	5	12%
92	Alpha-amylase 1 OS=Homo sapiens GN=AMY1A PE=1 SV=2	AMY1_HUMAN	58 kDa	29	74%
93	Alpha-N-acetylglucosaminidase OS=Homo sapiens GN=NAGLU PE=1 SV=2	ANAG_HUMAN	82 kDa	17	34%
94	Angiogenin OS=Homo sapiens GN=ANG PE=1 SV=1	ANGI_HUMAN	17 kDa	3	27%
95	Angiopoietin-related protein 4 OS=Homo sapiens GN=ANGPTL4 PE=1 SV=2	ANGL4_HUMAN	45 kDa	11	39%
96	Angiotensinogen OS=Homo sapiens GN=AGT PE=1 SV=1	ANGT_HUMAN	53 kDa	10	28%
97	Antithrombin-III OS=Homo sapiens GN=SERPINC1 PE=1 SV=1	ANT3_HUMAN	53 kDa	21	44%
98	Annexin A2 OS=Homo sapiens GN=ANXA2 PE=1 SV=2	ANXA2_HUMAN	39 kDa	8	28%
99	Annexin A5 OS=Homo sapiens GN=ANXA5 PE=1 SV=2	ANXA5_HUMAN	36 kDa	7	23%
100	Annexin A6 OS=Homo sapiens GN=ANXA6 PE=1 SV=3	ANXA6_HUMAN	76 kDa	3	5.90%
101	AP-1 complex subunit beta-1 OS=Homo sapiens GN=AP1B1 PE=1 SV=2	AP1B1_HUMAN	105 kDa	7	9.20%
102	AP-1 complex subunit gamma-1 OS=Homo sapiens GN=AP1G1 PE=1 SV=5	AP1G1_HUMAN	91 kDa	3	3.80%
103	AP-2 complex subunit alpha-1 OS=Homo sapiens GN=AP2A1 PE=1 SV=3	AP2A1_HUMAN	108 kDa	2	2.00%
104	Amyloid-like protein 2 OS=Homo sapiens GN=APLP2 PE=1 SV=2	APLP2_HUMAN	87 kDa	3	5.90%
105	Apolipoprotein A-I OS=Homo sapiens GN=APOA1 PE=1 SV=1	APOA1_HUMAN	31 kDa	30	78%
106	Apolipoprotein A-II OS=Homo sapiens GN=APOA2 PE=1 SV=1	APOA2_HUMAN	11 kDa	7	69%
107	Apolipoprotein A-IV OS=Homo sapiens GN=APOA4 PE=1 SV=3	APOA4_HUMAN	45 kDa	28	63%
108	Apolipoprotein B-100 OS=Homo sapiens GN=APOB PE=1 SV=2	APOB_HUMAN	516 kDa	103	27%
109	Apolipoprotein B receptor OS=Homo sapiens GN=APOBR PE=1 SV=1	APOBR_HUMAN	115 kDa	8	8.50%
110	Apolipoprotein C-III OS=Homo sapiens GN=APOC3 PE=1 SV=1	APOC3_HUMAN	11 kDa	2	27%
111	Apolipoprotein D OS=Homo sapiens GN=APOD PE=1 SV=1	APOD_HUMAN	21 kDa	8	38%
112	Apolipoprotein E OS=Homo sapiens GN=APOE PE=1 SV=1	APOE_HUMAN	36 kDa	24	71%
113	Apolipoprotein F OS=Homo sapiens GN=APOF PE=1 SV=2	APOF_HUMAN	35 kDa	2	8.30%
114	Beta-2-glycoprotein 1 OS=Homo sapiens GN=APOH PE=1 SV=3	APOH_HUMAN	38 kDa	12	46%
115	Apolipoprotein M OS=Homo sapiens GN=APOM PE=1 SV=2	APOM_HUMAN	21 kDa	2	8.50%
116	Adenine phosphoribosyltransferase OS=Homo sapiens GN=APRT PE=1 SV=2	APT_HUMAN	20 kDa	12	86%
117	Actin-related protein 2/3 complex subunit 1B OS=Homo sapiens GN=ARPC1B PE=1 SV=3	ARC1B_HUMAN	41 kDa	10	38%
118	ADP-ribosylation factor 1 OS=Homo sapiens GN=ARF1 PE=1 SV=2	ARF1_HUMAN	21 kDa	11	69%

#	Proteins	Uniprot ID	Molecular Weight	Number of unique peptides	Percent of coverage
119	ADP-ribosylation factor 4 OS=Homo sapiens GN=ARF4 PE=1 SV=3	ARF4_HUMAN	21 kDa	2	42%
120	ADP-ribosylation factor 6 OS=Homo sapiens GN=ARF6 PE=1 SV=2	ARF6_HUMAN	20 kDa	2	15%
121	Poly(ADP-ribose) glycohydrolase ARH3 OS=Homo sapiens GN=ADPRHL2 PE=1 SV=1	ARHL2_HUMAN	39 kDa	2	5.50%
122	Aflatoxin B1 aldehyde reductase member 2 OS=Homo sapiens GN=AKR7A2 PE=1 SV=3	ARK72_HUMAN	40 kDa	9	28%
123	ADP-ribosylation factor-like protein 3 OS=Homo sapiens GN=ARL3 PE=1 SV=2	ARL3_HUMAN	20 kDa	3	17%
124	Argininosuccinate lyase OS=Homo sapiens GN=ASL PE=1 SV=4	ARLY_HUMAN	52 kDa	13	30%
125	Actin-related protein 2 OS=Homo sapiens GN=ACTR2 PE=1 SV=1	ARP2_HUMAN	45 kDa	6	16%
126	Actin-related protein 3 OS=Homo sapiens GN=ACTR3 PE=1 SV=3	ARP3_HUMAN	47 kDa	13	36%
127	Actin-related protein 2/3 complex subunit 5-like protein OS=Homo sapiens GN=ARPC5L PE=1 SV=1	ARP5L_HUMAN	17 kDa	3	29%
128	Actin-related protein 2/3 complex subunit 2 OS=Homo sapiens GN=ARPC2 PE=1 SV=1	ARPC2_HUMAN	34 kDa	6	20%
129	Actin-related protein 2/3 complex subunit 3 OS=Homo sapiens GN=ARPC3 PE=1 SV=3	ARPC3_HUMAN	21 kDa	3	17%
130	Actin-related protein 2/3 complex subunit 4 OS=Homo sapiens GN=ARPC4 PE=1 SV=3	ARPC4_HUMAN	20 kDa	6	46%
131	Beta-arrestin-1 OS=Homo sapiens GN=ARRB1 PE=1 SV=2	ARRB1_HUMAN	47 kDa	5	15%
132	Arylsulfatase A OS=Homo sapiens GN=ARSA PE=1 SV=3	ARSA_HUMAN	54 kDa	10	25%
133	Acid ceramidase OS=Homo sapiens GN=ASAH1 PE=1 SV=5	ASAH1_HUMAN	45 kDa	4	12%
134	Apoptosis-associated speck-like protein containing a CARD OS=Homo sapiens GN=PYCARD PE=1 SV=2	ASC_HUMAN	22 kDa	4	24%
135	L-asparaginase OS=Homo sapiens GN=ASRGL1 PE=1 SV=2	ASGL1_HUMAN	32 kDa	2	9.10%
136	Sphingomyelin phosphodiesterase OS=Homo sapiens GN=SMPD1 PE=1 SV=4	ASM_HUMAN	70 kDa	2	3.30%
137	Acid sphingomyelinase-like phosphodiesterase 3a OS=Homo sapiens GN=SMPDL3A PE=1 SV=2	ASM3A_HUMAN	51 kDa	3	7.30%
138	Acid sphingomyelinase-like phosphodiesterase 3b OS=Homo sapiens GN=SMPDL3B PE=2 SV=2	ASM3B_HUMAN	51 kDa	7	26%
139	N(4)-(beta-N-acetylglucosaminyl)-L-asparaginase OS=Homo sapiens GN=AGA PE=1 SV=2	ASPG_HUMAN	37 kDa	2	5.20%
140	Probable cation-transporting ATPase 13A4 OS=Homo sapiens GN=ATP13A4 PE=2 SV=3	AT134_HUMAN	134 kDa	3	4.30%
141	Ubiquitin-like modifier-activating enzyme ATG7 OS=Homo sapiens GN=ATG7 PE=1 SV=1	ATG7_HUMAN	78 kDa	3	5.00%
142	Copper transport protein ATOX1 OS=Homo sapiens GN=ATOX1 PE=1 SV=1	ATOX1_HUMAN	7 kDa	3	38%
143	Attractin OS=Homo sapiens GN=ATRN PE=1 SV=2	ATRN_HUMAN	159 kDa	11	9.40%
144	Beta-2-microglobulin OS=Homo sapiens GN=B2M PE=1 SV=1	B2MG_HUMAN	14 kDa	6	47%
145	UDP-GlcNAc:betaGal beta-1,3-N-acetylglucosaminyltransferase 3 OS=Homo sapiens GN=B3GNT3 PE=1 SV=2	B3GN3_HUMAN	43 kDa	2	7.30%
146	UDP-GlcNAc:betaGal beta-1,3-N-acetylglucosaminyltransferase 7 OS=Homo sapiens GN=B3GNT7 PE=2 SV=1	B3GN7_HUMAN	46 kDa	4	9.70%
147	UDP-GlcNAc:betaGal beta-1,3-N-acetylglucosaminyltransferase 9 OS=Homo sapiens GN=B3GNT9 PE=2 SV=1	B3GN9_HUMAN	44 kDa	2	5.70%
148	Beta-1,4-galactosyltransferase 1 OS=Homo sapiens GN=B4GALT1 PE=1 SV=5	B4GT1_HUMAN	44 kDa	13	43%
149	B7 homolog 6 OS=Homo sapiens GN=B7H6 PE=1 SV=1	B7H6_HUMAN	51 kDa	6	19%
150	Brain-specific angiogenesis inhibitor 1-associated protein 2 OS=Homo sapiens GN=BAIAP2 PE=1 SV=1	BAIP2_HUMAN	61 kDa	4	8.20%
151	Basigin OS=Homo sapiens GN=BSG PE=1 SV=2	BASI_HUMAN	42 kDa	4	15%
152	3-hydroxybutyrate dehydrogenase type 2 OS=Homo sapiens GN=BDH2 PE=1 SV=2	BDH2_HUMAN	27 kDa	5	25%
153	Beta-galactosidase OS=Homo sapiens GN=GLB1 PE=1 SV=2	BGAL_HUMAN	76 kDa	13	23%
154	Transforming growth factor-beta-induced protein ig-h3 OS=Homo sapiens GN=TGFBI PE=1 SV=1	BGH3_HUMAN	75 kDa	2	3.40%
155	Brefeldin A-inhibited guanine nucleotide-exchange protein 2 OS=Homo sapiens GN=ARFGEF2 PE=1 SV=3	BIG2_HUMAN	202 kDa	2	1.60%
156	Myc box-dependent-interacting protein 1 OS=Homo sapiens GN=BIN1 PE=1 SV=1	BIN1_HUMAN	65 kDa	5	11%

#	Proteins	Uniprot ID	Molecular Weight	Number of unique peptides	Percent of coverage
157	Flavin reductase OS=Homo sapiens GN=BLVRB PE=1 SV=3	BLVRB_HUMAN	22 kDa	4	26%
158	Bone morphogenetic protein 1 OS=Homo sapiens GN=BMP1 PE=1 SV=2	BMP1_HUMAN	111 kDa	3	3.00%
159	3'(2'),5'-bisphosphate nucleotidase 1 OS=Homo sapiens GN=BPNT1 PE=1 SV=1	BPNT1_HUMAN	33 kDa	11	35%
160	BRO1 domain-containing protein BROX OS=Homo sapiens GN=BROX PE=1 SV=1	BROX_HUMAN	46 kDa	4	15%
161	Brain-specific serine protease 4 OS=Homo sapiens GN=PRSS22 PE=1 SV=1	BSSP4_HUMAN	34 kDa	5	19%
162	Butyrophilin subfamily 1 member A1 OS=Homo sapiens GN=BTN1A1 PE=1 SV=3	BT1A1_HUMAN	59 kDa	30	47%
163	Transcription factor BTF3 homolog 4 OS=Homo sapiens GN=BTF3L4 PE=1 SV=1	BT3L4_HUMAN	17 kDa	2	12%
164	Biotinidase OS=Homo sapiens GN=BTD PE=1 SV=2	BTD_HUMAN	61 kDa	10	22%
165	Complement C1q tumor necrosis factor-related protein 1 OS=Homo sapiens GN=C1QTNF1 PE=1 SV=1	C1QT1_HUMAN	32 kDa	3	10%
166	Complement C1r subcomponent OS=Homo sapiens GN=C1R PE=1 SV=2	C1R_HUMAN	80 kDa	6	12%
167	Complement C1r subcomponent-like protein OS=Homo sapiens GN=C1RL PE=1 SV=2	C1RL_HUMAN	53 kDa	10	24%
168	Complement C1s subcomponent OS=Homo sapiens GN=C1S PE=1 SV=1	C1S_HUMAN	77 kDa	5	8.40%
169	C-1-tetrahydrofolate synthase, cytoplasmic OS=Homo sapiens GN=MTHFD1 PE=1 SV=3	C1TC_HUMAN	102 kDa	14	15%
170	C4b-binding protein alpha chain OS=Homo sapiens GN=C4BPA PE=1 SV=2	C4BPA_HUMAN	67 kDa	12	23%
171	Voltage-dependent calcium channel subunit alpha-2/delta-1 OS=Homo sapiens GN=CACNA2D1 PE=1 SV=3	CA2D1_HUMAN	125 kDa	5	6.60%
172	Calcium-binding protein 39 OS=Homo sapiens GN=CAB39 PE=1 SV=1	CAB39_HUMAN	40 kDa	9	25%
173	45 kDa calcium-binding protein OS=Homo sapiens GN=SDF4 PE=1 SV=1	CAB45_HUMAN	42 kDa	19	56%
174	Cadherin-1 OS=Homo sapiens GN=CDH1 PE=1 SV=3	CADH1_HUMAN	97 kDa	6	7.30%
175	Carbonic anhydrase 2 OS=Homo sapiens GN=CA2 PE=1 SV=2	CAH2_HUMAN	29 kDa	6	27%
176	Carbonic anhydrase 6 OS=Homo sapiens GN=CA6 PE=1 SV=3	CAH6_HUMAN	35 kDa	12	42%
177	Calretinin OS=Homo sapiens GN=CALB2 PE=1 SV=2	CALB2_HUMAN	32 kDa	14	53%
178	Calmodulin-like protein 5 OS=Homo sapiens GN=CALML5 PE=1 SV=2	CALL5_HUMAN	16 kDa	13	78%
179	Calmodulin OS=Homo sapiens GN=CALM1 PE=1 SV=2	CALM_HUMAN	17 kDa	8	56%
180	Calreticulin OS=Homo sapiens GN=CALR PE=1 SV=1	CALR_HUMAN	48 kDa	8	28%
181	Calumenin OS=Homo sapiens GN=CALU PE=1 SV=2	CALU_HUMAN	37 kDa	7	20%
182	Calnexin OS=Homo sapiens GN=CANX PE=1 SV=2	CALX_HUMAN	68 kDa	2	4.60%
183	Calpain-1 catalytic subunit OS=Homo sapiens GN=CAPN1 PE=1 SV=1	CAN1_HUMAN	82 kDa	9	15%
184	Calcineurin subunit B type 1 OS=Homo sapiens GN=PPP3R1 PE=1 SV=2	CANB1_HUMAN	19 kDa	3	16%
185	Cullin-associated NEDD8-dissociated protein 1 OS=Homo sapiens GN=CAND1 PE=1 SV=2	CAND1_HUMAN	136 kDa	22	21%
186	Soluble calcium-activated nucleotidase 1 OS=Homo sapiens GN=CANT1 PE=1 SV=1	CANT1_HUMAN	45 kDa	8	27%
187	Adenylyl cyclase-associated protein 1 OS=Homo sapiens GN=CAP1 PE=1 SV=5	CAP1_HUMAN	52 kDa	14	35%
188	Macrophage-capping protein OS=Homo sapiens GN=CAPG PE=1 SV=2	CAPG_HUMAN	38 kDa	14	56%
189	Caprin-1 OS=Homo sapiens GN=CAPRIN1 PE=1 SV=2	CAPR1_HUMAN	78 kDa	4	5.20%
190	F-actin-capping protein subunit beta OS=Homo sapiens GN=CAPZB PE=1 SV=4	CAPZB_HUMAN	31 kDa	10	48%
191	Alpha-S1-casein OS=Homo sapiens GN=CSN1S1 PE=1 SV=1	CASA1_HUMAN	22 kDa	16	56%
192	Beta-casein OS=Homo sapiens GN=CSN2 PE=1 SV=4	CASB_HUMAN	25 kDa	12	90%
193	Protein CASC4 OS=Homo sapiens GN=CASC4 PE=2 SV=1	CASC4_HUMAN	49 kDa	2	4.40%
194	Kappa-casein OS=Homo sapiens GN=CSN3 PE=1 SV=3	CASK_HUMAN	20 kDa	10	68%
195	Caspase-3 OS=Homo sapiens GN=CASP3 PE=1 SV=2	CASP3_HUMAN	32 kDa	4	18%
196	Catalase OS=Homo sapiens GN=CAT PE=1 SV=3	CATA_HUMAN	60 kDa	11	25%

#	Proteins	Uniprot ID	Molecular Weight	Number of unique peptides	Percent of coverage
197	Cathepsin B OS=Homo sapiens GN=CTSB PE=1 SV=3	CATB_HUMAN	38 kDa	8	28%
198	Dipeptidyl peptidase 1 OS=Homo sapiens GN=CTSC PE=1 SV=2	CATC_HUMAN	52 kDa	12	31%
199	Cathepsin D OS=Homo sapiens GN=CTSD PE=1 SV=1	CATD_HUMAN	45 kDa	16	44%
200	Pro-cathepsin H OS=Homo sapiens GN=CTSH PE=1 SV=4	CATH_HUMAN	37 kDa	4	11%
201	Cathepsin S OS=Homo sapiens GN=CTSS PE=1 SV=3	CATS_HUMAN	37 kDa	12	43%
202	Cathepsin Z OS=Homo sapiens GN=CTSZ PE=1 SV=1	CATZ_HUMAN	34 kDa	5	18%
203	F-actin-capping protein subunit alpha-1 OS=Homo sapiens GN=CAPZA1 PE=1 SV=3	CAZA1_HUMAN	33 kDa	9	44%
204	F-actin-capping protein subunit alpha-2 OS=Homo sapiens GN=CAPZA2 PE=1 SV=3	CAZA2_HUMAN	33 kDa	5	36%
205	Calcium-binding protein 39-like OS=Homo sapiens GN=CAB39L PE=1 SV=3	CB39L_HUMAN	39 kDa	4	15%
206	Corticosteroid-binding globulin OS=Homo sapiens GN=SERPINA6 PE=1 SV=1	CBG_HUMAN	45 kDa	8	28%
207	Carboxypeptidase B2 OS=Homo sapiens GN=CPB2 PE=1 SV=2	CBPB2_HUMAN	48 kDa	5	14%
208	Carboxypeptidase Z OS=Homo sapiens GN=CPZ PE=1 SV=2	CBPZ_HUMAN	74 kDa	4	8.10%
209	Carbonyl reductase [NADPH] 1 OS=Homo sapiens GN=CBR1 PE=1 SV=3	CBR1_HUMAN	30 kDa	13	56%
210	Cystathionine beta-synthase OS=Homo sapiens GN=CBS PE=1 SV=2	CBS_HUMAN	61 kDa	2	3.80%
211	Monocyte differentiation antigen CD14 OS=Homo sapiens GN=CD14 PE=1 SV=2	CD14_HUMAN	40 kDa	21	69%
212	CD2-associated protein OS=Homo sapiens GN=CD2AP PE=1 SV=1	CD2AP_HUMAN	71 kDa	2	3.10%
213	Platelet glycoprotein 4 OS=Homo sapiens GN=CD36 PE=1 SV=2	CD36_HUMAN	53 kDa	12	29%
214	Leukocyte surface antigen CD47 OS=Homo sapiens GN=CD47 PE=1 SV=1	CD47_HUMAN	35 kDa	3	8.70%
215	CD59 glycoprotein OS=Homo sapiens GN=CD59 PE=1 SV=1	CD59_HUMAN	14 kDa	5	27%
216	CD5 antigen-like OS=Homo sapiens GN=CD5L PE=1 SV=1	CD5L_HUMAN	38 kDa	3	8.90%
217	CD81 antigen OS=Homo sapiens GN=CD81 PE=1 SV=1	CD81_HUMAN	26 kDa	4	28%
218	CD9 antigen OS=Homo sapiens GN=CD9 PE=1 SV=4	CD9_HUMAN	25 kDa	4	18%
219	Hsp90 co-chaperone Cdc37 OS=Homo sapiens GN=CDC37 PE=1 SV=1	CDC37_HUMAN	44 kDa	7	20%
220	Cell division control protein 42 homolog OS=Homo sapiens GN=CDC42 PE=1 SV=2	CDC42_HUMAN	21 kDa	6	44%
221	Protein CDV3 homolog OS=Homo sapiens GN=CDV3 PE=1 SV=1	CDV3_HUMAN	27 kDa	4	34%
222	Bile salt-activated lipase OS=Homo sapiens GN=CEL PE=1 SV=3	CEL_HUMAN	79 kDa	37	56%
223	Ceruloplasmin OS=Homo sapiens GN=CP PE=1 SV=1	CERU_HUMAN	122 kDa	38	44%
224	Uncharacterized protein C6orf15 OS=Homo sapiens GN=C6orf15 PE=2 SV=3	CF015_HUMAN	34 kDa	6	40%
225	Costars family protein C6orf115 OS=Homo sapiens GN=C6orf115 PE=1 SV=1	CF115_HUMAN	9 kDa	4	47%
226	UPF0364 protein C6orf211 OS=Homo sapiens GN=C6orf211 PE=1 SV=1	CF211_HUMAN	51 kDa	5	12%
227	Complement factor B OS=Homo sapiens GN=CFB PE=1 SV=2	CFAB_HUMAN	86 kDa	29	42%
228	Complement factor H OS=Homo sapiens GN=CFH PE=1 SV=4	CFAH_HUMAN	139 kDa	25	27%
229	Complement factor I OS=Homo sapiens GN=CFI PE=1 SV=2	CFAI_HUMAN	66 kDa	15	33%
230	Cystathionine gamma-lyase OS=Homo sapiens GN=CTH PE=1 SV=3	CGL_HUMAN	45 kDa	4	15%
231	Cell growth regulator with EF hand domain protein 1 OS=Homo sapiens GN=CGREF1 PE=2 SV=2	CGRE1_HUMAN	32 kDa	4	16%
232	10 kDa heat shock protein, mitochondrial OS=Homo sapiens GN=HSPE1 PE=1 SV=2	CH10_HUMAN	11 kDa	3	31%
233	Chitinase-3-like protein 1 OS=Homo sapiens GN=CHI3L1 PE=1 SV=2	CH3L1_HUMAN	43 kDa	14	44%
234	Chitinase-3-like protein 2 OS=Homo sapiens GN=CHI3L2 PE=1 SV=1	CH3L2_HUMAN	44 kDa	6	22%
235	60 kDa heat shock protein, mitochondrial OS=Homo sapiens GN=HSPD1 PE=1 SV=2	CH60_HUMAN	61 kDa	3	6.80%
236	Chitinase domain-containing protein 1 OS=Homo sapiens GN=CHID1 PE=1 SV=1	CHID1_HUMAN	45 kDa	4	13%
237	Charged multivesicular body protein 4b OS=Homo sapiens GN=CHMP4B PE=1 SV=1	CHM4B_HUMAN	25 kDa	6	29%

#	Proteins	Uniprot ID	Molecular Weight	Number of unique peptides	Percent of coverage
238	Charged multivesicular body protein 3 OS=Homo sapiens GN=VPS24 PE=1 SV=3	CHMP3_HUMAN	25 kDa	2	9.00%
239	Calcium-regulated heat stable protein 1 OS=Homo sapiens GN=CARHSP1 PE=1 SV=2	CHSP1_HUMAN	16 kDa	4	31%
240	Calcium and integrin-binding protein 1 OS=Homo sapiens GN=CIB1 PE=1 SV=4	CIB1_HUMAN	22 kDa	6	35%
241	Cell death activator CIDE-A OS=Homo sapiens GN=CIDEA PE=1 SV=1	CIDEA_HUMAN	25 kDa	2	10%
242	Cold-inducible RNA-binding protein OS=Homo sapiens GN=CIRBP PE=1 SV=1	CIRBP_HUMAN	19 kDa	2	20%
243	Ester hydrolase C11orf54 OS=Homo sapiens GN=C11orf54 PE=1 SV=1	CK054_HUMAN	35 kDa	2	9.20%
244	CDK5 regulatory subunit-associated protein 2 OS=Homo sapiens GN=CDK5RAP2 PE=1 SV=5	CK5P2_HUMAN	215 kDa	2	0.85%
245	UPF0444 transmembrane protein C12orf23 OS=Homo sapiens GN=C12orf23 PE=1 SV=1	CL023_HUMAN	12 kDa	3	35%
246	Clathrin heavy chain 1 OS=Homo sapiens GN=CLTC PE=1 SV=5	CLH1_HUMAN	192 kDa	3	2.50%
247	Chloride intracellular channel protein 1 OS=Homo sapiens GN=CLIC1 PE=1 SV=4	CLIC1_HUMAN	27 kDa	13	74%
248	Chloride intracellular channel protein 4 OS=Homo sapiens GN=CLIC4 PE=1 SV=4	CLIC4_HUMAN	29 kDa	3	15%
249	Ceroid-lipofuscinosis neuronal protein 5 OS=Homo sapiens GN=CLN5 PE=1 SV=2	CLN5_HUMAN	41 kDa	5	13%
250	Clusterin OS=Homo sapiens GN=CLU PE=1 SV=1	CLUS_HUMAN	52 kDa	24	45%
251	Carboxymethylenebutenolidase homolog OS=Homo sapiens GN=CMBL PE=1 SV=1	CMBL_HUMAN	28 kDa	3	10%
252	UPF0568 protein C14orf166 OS=Homo sapiens GN=C14orf166 PE=1 SV=1	CN166_HUMAN	28 kDa	2	12%
253	2',3'-cyclic-nucleotide 3'-phosphodiesterase OS=Homo sapiens GN=CNP PE=1 SV=2	CN37_HUMAN	48 kDa	3	6.20%
254	Cellular nucleic acid-binding protein OS=Homo sapiens GN=CNBP PE=1 SV=1	CNBP_HUMAN	19 kDa	4	22%
255	Cytosolic non-specific dipeptidase OS=Homo sapiens GN=CNDP2 PE=1 SV=2	CNDP2_HUMAN	53 kDa	28	66%
256	Calponin-2 OS=Homo sapiens GN=CNN2 PE=1 SV=4	CNN2_HUMAN	34 kDa	3	11%
257	Protein canopy homolog 2 OS=Homo sapiens GN=CNPY2 PE=1 SV=1	CNPY2_HUMAN	21 kDa	3	19%
258	UPF0552 protein C15orf38 OS=Homo sapiens GN=C15orf38 PE=1 SV=1	CO038_HUMAN	25 kDa	2	9.30%
259	Complement C2 OS=Homo sapiens GN=C2 PE=1 SV=2	CO2_HUMAN	83 kDa	12	19%
260	Complement C3 OS=Homo sapiens GN=C3 PE=1 SV=2	CO3_HUMAN	187 kDa	116	78%
261	Complement C4-A OS=Homo sapiens GN=C4A PE=1 SV=1	CO4A_HUMAN	193 kDa	85	59%
262	Complement C4-B OS=Homo sapiens GN=C4B PE=1 SV=1	CO4B_HUMAN	193 kDa	2	59%
263	Complement C5 OS=Homo sapiens GN=C5 PE=1 SV=4	CO5_HUMAN	188 kDa	14	10%
264	Complement component C6 OS=Homo sapiens GN=C6 PE=1 SV=3	CO6_HUMAN	105 kDa	9	11%
265	Complement component C7 OS=Homo sapiens GN=C7 PE=1 SV=2	CO7_HUMAN	94 kDa	25	41%
266	Complement component C8 alpha chain OS=Homo sapiens GN=C8A PE=1 SV=2	CO8A_HUMAN	65 kDa	6	12%
267	Complement component C8 beta chain OS=Homo sapiens GN=C8B PE=1 SV=3	CO8B_HUMAN	67 kDa	6	13%
268	Complement component C8 gamma chain OS=Homo sapiens GN=C8G PE=1 SV=3	CO8G_HUMAN	22 kDa	3	16%
269	Complement component C9 OS=Homo sapiens GN=C9 PE=1 SV=2	CO9_HUMAN	63 kDa	21	38%
270	Bifunctional coenzyme A synthase OS=Homo sapiens GN=COASY PE=1 SV=4	COASY_HUMAN	62 kDa	5	12%
271	Cochlin OS=Homo sapiens GN=COCH PE=1 SV=1	COCH_HUMAN	59 kDa	10	19%
272	Cofilin-1 OS=Homo sapiens GN=CFL1 PE=1 SV=3	COF1_HUMAN	19 kDa	12	69%
273	Catechol O-methyltransferase OS=Homo sapiens GN=COMT PE=1 SV=2	COMT_HUMAN	30 kDa	9	39%
274	Coatomer subunit alpha OS=Homo sapiens GN=COPA PE=1 SV=2	COPA_HUMAN	138 kDa	4	2.70%
275	Coatomer subunit beta OS=Homo sapiens GN=COPB1 PE=1 SV=3	COPB_HUMAN	107 kDa	2	2.30%
276	Coatomer subunit beta' OS=Homo sapiens GN=COPB2 PE=1 SV=2	COPB2_HUMAN	102 kDa	2	2.30%

#	Proteins	Uniprot ID	Molecular Weight	Number of unique peptides	Percent of coverage
277	Coatomer subunit delta OS=Homo sapiens GN=ARCN1 PE=1 SV=1	COPD_HUMAN	57 kDa	7	15%
278	Coatomer subunit gamma OS=Homo sapiens GN=COPG PE=1 SV=1	COPG_HUMAN	98 kDa	3	4.50%
279	Coronin-1A OS=Homo sapiens GN=CORO1A PE=1 SV=4	COR1A_HUMAN	51 kDa	2	6.70%
280	Coronin-1B OS=Homo sapiens GN=CORO1B PE=1 SV=1	COR1B_HUMAN	54 kDa	8	24%
281	Coactosin-like protein OS=Homo sapiens GN=COTL1 PE=1 SV=3	COTL1_HUMAN	16 kDa	3	25%
282	UPF0764 protein C16orf89 OS=Homo sapiens GN=C16orf89 PE=2 SV=2	CP089_HUMAN	45 kDa	5	16%
283	Carboxypeptidase N subunit 2 OS=Homo sapiens GN=CPN2 PE=1 SV=3	CPN2_HUMAN	61 kDa	2	4.60%
284	Calpain small subunit 1 OS=Homo sapiens GN=CAPNS1 PE=1 SV=1	CPNS1_HUMAN	28 kDa	3	14%
285	Protein C17orf37 OS=Homo sapiens GN=C17orf37 PE=1 SV=1	CQ037_HUMAN	12 kDa	2	16%
286	Chordin-like protein 2 OS=Homo sapiens GN=CHRDL2 PE=1 SV=1	CRDL2_HUMAN	47 kDa	21	60%
287	Cysteine-rich motor neuron 1 protein OS=Homo sapiens GN=CRIM1 PE=1 SV=1	CRIM1_HUMAN	114 kDa	8	12%
288	Crk-like protein OS=Homo sapiens GN=CRKL PE=1 SV=1	CRKL_HUMAN	34 kDa	2	6.90%
289	C-reactive protein OS=Homo sapiens GN=CRP PE=1 SV=1	CRP_HUMAN	25 kDa	2	9.80%
290	Lambda-crystallin homolog OS=Homo sapiens GN=CRYL1 PE=1 SV=3	CRYL1_HUMAN	35 kDa	7	21%
291	UPF0556 protein C19orf10 OS=Homo sapiens GN=C19orf10 PE=1 SV=1	CS010_HUMAN	19 kDa	3	21%
292	Cold shock domain-containing protein E1 OS=Homo sapiens GN=CSDE1 PE=1 SV=2	CSDE1_HUMAN	89 kDa	3	2.60%
293	Macrophage colony-stimulating factor 1 OS=Homo sapiens GN=CSF1 PE=1 SV=2	CSF1_HUMAN	60 kDa	7	16%
294	Granulocyte-macrophage colony-stimulating factor receptor subunit alpha OS=Homo sapiens GN=CSF2RA PE=1 SV=1	CSF2R_HUMAN	46 kDa	2	6.20%
295	COP9 signalosome complex subunit 1 OS=Homo sapiens GN=GPS1 PE=1 SV=4	CSN1_HUMAN	56 kDa	2	4.30%
296	COP9 signalosome complex subunit 2 OS=Homo sapiens GN=COPS2 PE=1 SV=1	CSN2_HUMAN	52 kDa	3	7.70%
297	COP9 signalosome complex subunit 3 OS=Homo sapiens GN=COPS3 PE=1 SV=3	CSN3_HUMAN	48 kDa	2	5.40%
298	COP9 signalosome complex subunit 4 OS=Homo sapiens GN=COPS4 PE=1 SV=1	CSN4_HUMAN	46 kDa	4	15%
299	COP9 signalosome complex subunit 5 OS=Homo sapiens GN=COPS5 PE=1 SV=4	CSN5_HUMAN	38 kDa	3	9.90%
300	Cysteine and glycine-rich protein 1 OS=Homo sapiens GN=CSRP1 PE=1 SV=3	CSRP1_HUMAN	21 kDa	4	33%
301	Calsyntenin-1 OS=Homo sapiens GN=CLSTN1 PE=1 SV=1	CSTN1_HUMAN	110 kDa	4	5.30%
302	Cullin-4A OS=Homo sapiens GN=CUL4A PE=1 SV=3	CUL4A_HUMAN	88 kDa	2	2.50%
303	Protein CutA OS=Homo sapiens GN=CUTA PE=1 SV=2	CUTA_HUMAN	19 kDa	3	31%
304	UPF0368 protein Cxorf26 OS=Homo sapiens GN=CXorf26 PE=1 SV=1	CX026_HUMAN	26 kDa	2	9.00%
305	C-X-C motif chemokine 2 OS=Homo sapiens GN=CXCL2 PE=1 SV=1	CXCL2_HUMAN	11 kDa	5	47%
306	Cytochrome b5 OS=Homo sapiens GN=CYB5A PE=1 SV=2	CYB5_HUMAN	15 kDa	4	34%
307	Cytochrome b reductase 1 OS=Homo sapiens GN=CYBRD1 PE=1 SV=1	CYBR1_HUMAN	32 kDa	3	12%
308	Cytoplasmic FMR1-interacting protein 1 OS=Homo sapiens GN=CYFIP1 PE=1 SV=1	CYFP1_HUMAN	145 kDa	5	4.50%
309	Cystatin-B OS=Homo sapiens GN=CSTB PE=1 SV=2	CYTB_HUMAN	11 kDa	5	78%
310	Cystatin-C OS=Homo sapiens GN=CST3 PE=1 SV=1	CYTC_HUMAN	16 kDa	11	67%
311	Disabled homolog 2 OS=Homo sapiens GN=DAB2 PE=1 SV=3	DAB2_HUMAN	82 kDa	7	11%
312	Dystroglycan OS=Homo sapiens GN=DAG1 PE=1 SV=2	DAG1_HUMAN	97 kDa	13	23%
313	Drebrin-like protein OS=Homo sapiens GN=DBNL PE=1 SV=1	DBNL_HUMAN	48 kDa	5	13%
314	DNA-binding protein A OS=Homo sapiens GN=CSDA PE=1 SV=4	DBPA_HUMAN	40 kDa	3	16%
315	Deoxycytidylate deaminase OS=Homo sapiens GN=DCTD PE=1 SV=2	DCTD_HUMAN	20 kDa	3	21%
316	Dynactin subunit 1 OS=Homo sapiens GN=DCTN1 PE=1 SV=3	DCTN1_HUMAN	142 kDa	2	2.00%
317	Dynactin subunit 2 OS=Homo sapiens GN=DCTN2 PE=1 SV=4	DCTN2_HUMAN	44 kDa	6	16%

#	Proteins	Uniprot ID	Molecular Weight	Number of unique peptides	Percent of coverage
318	L-xylulose reductase OS=Homo sapiens GN=DCXR PE=1 SV=2	DCXR_HUMAN	26 kDa	14	81%
319	N(G),N(G)-dimethylarginine dimethylaminohydrolase 1 OS=Homo sapiens GN=DDAH1 PE=1 SV=3	DDAH1_HUMAN	31 kDa	16	67%
320	N(G),N(G)-dimethylarginine dimethylaminohydrolase 2 OS=Homo sapiens GN=DDAH2 PE=1 SV=1	DDAH2_HUMAN	30 kDa	5	22%
321	DNA damage-binding protein 1 OS=Homo sapiens GN=DDB1 PE=1 SV=1	DDB1_HUMAN	127 kDa	5	4.10%
322	Epithelial discoidin domain-containing receptor 1 OS=Homo sapiens GN=DDR1 PE=1 SV=1	DDR1_HUMAN	101 kDa	14	16%
323	ATP-dependent RNA helicase DDX3X OS=Homo sapiens GN=DDX3X PE=1 SV=3	DDX3X_HUMAN	73 kDa	5	8.20%
324	Probable ATP-dependent RNA helicase DDX6 OS=Homo sapiens GN=DDX6 PE=1 SV=2	DDX6_HUMAN	54 kDa	2	3.70%
325	Neutrophil defensin 1 OS=Homo sapiens GN=DEFA1 PE=1 SV=1	DEF1_HUMAN (+1)	10 kDa	2	16%
326	Density-regulated protein OS=Homo sapiens GN=DENR PE=1 SV=2	DENR_HUMAN	22 kDa	5	30%
327	Destrin OS=Homo sapiens GN=DSTN PE=1 SV=3	DEST_HUMAN	19 kDa	9	58%
328	Bifunctional ATP-dependent dihydroxyacetone kinase/FAD-AMP lyase (cyclizing) OS=Homo sapiens GN=DAK PE=1 SV=2	DHAK_HUMAN	59 kDa	13	32%
329	Peroxisomal multifunctional enzyme type 2 OS=Homo sapiens GN=HSD17B4 PE=1 SV=3	DHB4_HUMAN	80 kDa	3	5.40%
330	Dihydropteridine reductase OS=Homo sapiens GN=QDPR PE=1 SV=2	DHPR_HUMAN	26 kDa	5	28%
331	Di-N-acetylchitobiase OS=Homo sapiens GN=CTBS PE=1 SV=1	DIAC_HUMAN	44 kDa	8	23%
332	Protein diaphanous homolog 1 OS=Homo sapiens GN=DIAPH1 PE=1 SV=2	DIAP1_HUMAN	141 kDa	4	3.00%
333	Disks large-associated protein 4 OS=Homo sapiens GN=DLGAP4 PE=1 SV=3	DLGP4_HUMAN	108 kDa	2	3.10%
334	Delta and Notch-like epidermal growth factor-related receptor OS=Homo sapiens GN=DNER PE=1 SV=1	DNER_HUMAN	78 kDa	2	4.60%
335	DnaJ homolog subfamily B member 2 OS=Homo sapiens GN=DNAJB2 PE=2 SV=3	DNJB2_HUMAN	36 kDa	3	12%
336	DnaJ homolog subfamily C member 3 OS=Homo sapiens GN=DNAJC3 PE=1 SV=1	DNJC3_HUMAN	58 kDa	7	16%
337	DNA ligase 1 OS=Homo sapiens GN=LIG1 PE=1 SV=1	DNLI1_HUMAN	102 kDa	2	2.00%
338	Deoxyribonuclease-2-alpha OS=Homo sapiens GN=DNASE2 PE=1 SV=2	DNS2A_HUMAN	40 kDa	5	15%
339	D-dopachrome decarboxylase OS=Homo sapiens GN=DDT PE=1 SV=3	DOPD_HUMAN	13 kDa	4	36%
340	HLA class II histocompatibility antigen, DP alpha 1 chain OS=Homo sapiens GN=HLA-DPA1 PE=1 SV=1	DPA1_HUMAN	29 kDa	2	11%
341	Dolichol-phosphate mannosyltransferase subunit 3 OS=Homo sapiens GN=DPM3 PE=1 SV=2	DPM3_HUMAN	10 kDa	2	24%
342	Dipeptidyl peptidase 2 OS=Homo sapiens GN=DPP7 PE=1 SV=3	DPP2_HUMAN	54 kDa	11	27%
343	Dipeptidyl peptidase 3 OS=Homo sapiens GN=DPP3 PE=1 SV=2	DPP3_HUMAN	83 kDa	11	20%
344	Dihydropyrimidinase-related protein 2 OS=Homo sapiens GN=DPYSL2 PE=1 SV=1	DPYL2_HUMAN	62 kDa	25	64%
345	Dihydropyrimidinase-related protein 3 OS=Homo sapiens GN=DPYSL3 PE=1 SV=1	DPYL3_HUMAN	62 kDa	18	57%
346	HLA class II histocompatibility antigen, DR alpha chain OS=Homo sapiens GN=HLA-DRA PE=1 SV=1	DRA_HUMAN	29 kDa	4	21%
347	Developmentally-regulated GTP-binding protein 1 OS=Homo sapiens GN=DRG1 PE=1 SV=1	DRG1_HUMAN	41 kDa	2	6.80%
348	Developmentally-regulated GTP-binding protein 2 OS=Homo sapiens GN=DRG2 PE=1 SV=1	DRG2_HUMAN	41 kDa	2	5.80%
349	Desmocollin-2 OS=Homo sapiens GN=DSC2 PE=1 SV=1	DSC2_HUMAN	100 kDa	6	6.40%
350	Cytoplasmic dynein 1 heavy chain 1 OS=Homo sapiens GN=DYNC1H1 PE=1 SV=5	DYHC1_HUMAN	532 kDa	35	9.00%
351	Dynein light chain 2, cytoplasmic OS=Homo sapiens GN=DYNLL2 PE=1 SV=1	DYL2_HUMAN	10 kDa	2	33%
352	Delta(3,5)-Delta(2,4)-dienoyl-CoA isomerase, mitochondrial OS=Homo sapiens GN=ECH1 PE=1 SV=2	ECH1_HUMAN	36 kDa	2	7.30%
353	Enoyl-CoA hydratase domain-containing protein 1 OS=Homo sapiens GN=ECHDC1 PE=1 SV=2	ECHD1_HUMAN	34 kDa	2	10%
354	Extracellular matrix protein 1 OS=Homo sapiens GN=ECM1 PE=1 SV=2	ECM1_HUMAN	61 kDa	4	8.10%
355	Extracellular matrix protein 2 OS=Homo sapiens GN=ECM2 PE=2 SV=1	ECM2_HUMAN	80 kDa	2	3.40%

**#**	**Proteins**	**Uniprot ID**	**Molecular Weight**	**Number of unique peptides**	**Percent of coverage**
356	Proteasome-associated protein ECM29 homolog OS=Homo sapiens GN=ECM29 PE=1 SV=2	ECM29_HUMAN	204 kDa	2	1.30%
357	Early endosome antigen 1 OS=Homo sapiens GN=EEA1 PE=1 SV=2	EEA1_HUMAN	162 kDa	3	1.40%
358	Elongation factor 1-alpha 1 OS=Homo sapiens GN=EEF1A1 PE=1 SV=1	EF1A1_HUMAN (+1)	50 kDa	18	48%
359	Elongation factor 1-beta OS=Homo sapiens GN=EEF1B2 PE=1 SV=3	EF1B_HUMAN	25 kDa	6	33%
360	Elongation factor 1-delta OS=Homo sapiens GN=EEF1D PE=1 SV=5	EF1D_HUMAN	31 kDa	10	47%
361	Elongation factor 1-gamma OS=Homo sapiens GN=EEF1G PE=1 SV=3	EF1G_HUMAN	50 kDa	12	24%
362	Elongation factor 2 OS=Homo sapiens GN=EEF2 PE=1 SV=4	EF2_HUMAN	95 kDa	39	54%
363	EF-hand domain-containing protein D1 OS=Homo sapiens GN=EFHD1 PE=1 SV=1	EFHD1_HUMAN	27 kDa	13	67%
364	EF-hand domain-containing protein D2 OS=Homo sapiens GN=EFHD2 PE=1 SV=1	EFHD2_HUMAN	27 kDa	2	18%
365	Pro-epidermal growth factor OS=Homo sapiens GN=EGF PE=1 SV=2	EGF_HUMAN	134 kDa	27	25%
366	Endoglin OS=Homo sapiens GN=ENG PE=1 SV=2	EGLN_HUMAN	71 kDa	3	5.00%
367	EH domain-containing protein 4 OS=Homo sapiens GN=EHD4 PE=1 SV=1	EHD4_HUMAN	61 kDa	9	18%
368	Translation initiation factor eIF-2B subunit epsilon OS=Homo sapiens GN=EIF2B5 PE=1 SV=3	EI2BE_HUMAN	80 kDa	2	3.10%
369	Eukaryotic translation initiation factor 1 OS=Homo sapiens GN=EIF1 PE=1 SV=1	EIF1_HUMAN	13 kDa	4	61%
370	Eukaryotic translation initiation factor 2A OS=Homo sapiens GN=EIF2A PE=1 SV=3	EIF2A_HUMAN	65 kDa	2	4.80%
371	Eukaryotic translation initiation factor 3 subunit A OS=Homo sapiens GN=EIF3A PE=1 SV=1	EIF3A_HUMAN	167 kDa	2	1.90%
372	Eukaryotic translation initiation factor 3 subunit C OS=Homo sapiens GN=EIF3C PE=1 SV=1	EIF3C_HUMAN	105 kDa	2	2.80%
373	Eukaryotic translation initiation factor 3 subunit F OS=Homo sapiens GN=EIF3F PE=1 SV=1	EIF3F_HUMAN	38 kDa	3	9.00%
374	Eukaryotic translation initiation factor 3 subunit H OS=Homo sapiens GN=EIF3H PE=1 SV=1	EIF3H_HUMAN	40 kDa	2	8.80%
375	Eukaryotic translation initiation factor 3 subunit I OS=Homo sapiens GN=EIF3I PE=1 SV=1	EIF3I_HUMAN	37 kDa	3	9.20%
376	Eukaryotic translation initiation factor 3 subunit J OS=Homo sapiens GN=EIF3J PE=1 SV=2	EIF3J_HUMAN	29 kDa	3	19%
377	Eukaryotic translation initiation factor 3 subunit L OS=Homo sapiens GN=EIF3L PE=1 SV=1	EIF3L_HUMAN	67 kDa	2	3.00%
378	Transcription elongation factor B polypeptide 1 OS=Homo sapiens GN=TCEB1 PE=1 SV=1	ELOC_HUMAN	12 kDa	2	17%
379	Echinoderm microtubule-associated protein-like 2 OS=Homo sapiens GN=EML2 PE=1 SV=1	EMAL2_HUMAN	71 kDa	7	13%
380	Alpha-enolase OS=Homo sapiens GN=ENO1 PE=1 SV=2	ENOA_HUMAN	47 kDa	30	78%
381	Mitochondrial enolase superfamily member 1 OS=Homo sapiens GN=ENOSF1 PE=1 SV=1	ENOF1_HUMAN	50 kDa	3	11%
382	Enolase-phosphatase E1 OS=Homo sapiens GN=ENOPH1 PE=1 SV=1	ENOPH_HUMAN	29 kDa	4	28%
383	Endoplasmin OS=Homo sapiens GN=HSP90B1 PE=1 SV=1	ENPL_HUMAN	92 kDa	22	26%
384	Alpha-endosulfine OS=Homo sapiens GN=ENSA PE=1 SV=1	ENSA_HUMAN	13 kDa	4	29%
385	Ectonucleoside triphosphate diphosphohydrolase 3 OS=Homo sapiens GN=ENTPD3 PE=1 SV=2	ENTP3_HUMAN	59 kDa	5	11%
386	Ephrin type-A receptor 7 OS=Homo sapiens GN=EPHA7 PE=1 SV=3	EPHA7_HUMAN	112 kDa	10	13%
387	Epiplakin OS=Homo sapiens GN=EPPK1 PE=1 SV=2	EPIPL_HUMAN	556 kDa	2	0.90%
388	Endoplasmic reticulum aminopeptidase 1 OS=Homo sapiens GN=ERAP1 PE=1 SV=3	ERAP1_HUMAN	107 kDa	15	19%
389	Eukaryotic peptide chain release factor GTP-binding subunit ERF3A OS=Homo sapiens GN=GSPT1 PE=1 SV=1	ERF3A_HUMAN	56 kDa	6	14%
390	Lanosterol synthase OS=Homo sapiens GN=LSS PE=1 SV=1	ERG7_HUMAN	83 kDa	8	12%
391	Endoplasmic reticulum resident protein 29 OS=Homo sapiens GN=ERP29 PE=1 SV=4	ERP29_HUMAN	29 kDa	4	16%
392	Endoplasmic reticulum resident protein 44 OS=Homo sapiens GN=ERP44 PE=1 SV=1	ERP44_HUMAN	47 kDa	2	10%
393	Epidermal growth factor receptor kinase substrate 8-like protein 2 OS=Homo sapiens GN=EPS8L2 PE=1 SV=2	ES8L2_HUMAN	81 kDa	4	11%

#	Proteins	Uniprot ID	Molecular Weight	Number of unique peptides	Percent of coverage
394	Carboxylesterase 3 OS=Homo sapiens GN=CES3 PE=1 SV=1	EST3_HUMAN	62 kDa	3	8.20%
395	Carboxylesterase 4A OS=Homo sapiens GN=CES4A PE=2 SV=2	EST4A_HUMAN	64 kDa	2	3.90%
396	S-formylglutathione hydrolase OS=Homo sapiens GN=ESD PE=1 SV=2	ESTD_HUMAN	31 kDa	9	43%
397	Ellis-van Creveld syndrome protein OS=Homo sapiens GN=EVC PE=1 SV=1	EVC_HUMAN	112 kDa	2	2.60%
398	Ezrin OS=Homo sapiens GN=EZR PE=1 SV=4	EZRI_HUMAN	69 kDa	26	54%
399	Protein FAM107B OS=Homo sapiens GN=FAM107B PE=1 SV=1	F107B_HUMAN	16 kDa	8	51%
400	Hsc70-interacting protein OS=Homo sapiens GN=ST13 PE=1 SV=2	F10A1_HUMAN	41 kDa	8	24%
401	Fructose-1,6-bisphosphatase 1 OS=Homo sapiens GN=FBP1 PE=1 SV=5	F16P1_HUMAN	37 kDa	15	54%
402	Coagulation factor XII OS=Homo sapiens GN=F12 PE=1 SV=3	FA12_HUMAN	68 kDa	4	8.60%
403	Protein FAM20A OS=Homo sapiens GN=FAM20A PE=2 SV=4	FA20A_HUMAN	61 kDa	2	6.70%
404	Protein FAM83F OS=Homo sapiens GN=FAM83F PE=2 SV=1	FA83F_HUMAN	55 kDa	2	7.20%
405	Fumarylacetoacetase OS=Homo sapiens GN=FAH PE=1 SV=2	FAAA_HUMAN	46 kDa	8	22%
406	Fatty acid-binding protein, brain OS=Homo sapiens GN=FABP7 PE=1 SV=3	FABP7_HUMAN	15 kDa	7	58%
407	Fatty acid-binding protein, heart OS=Homo sapiens GN=FABP3 PE=1 SV=4	FABPH_HUMAN	15 kDa	16	81%
408	Protein FAM3B OS=Homo sapiens GN=FAM3B PE=1 SV=2	FAM3B_HUMAN	26 kDa	8	39%
409	Protein FAM3C OS=Homo sapiens GN=FAM3C PE=1 SV=1	FAM3C_HUMAN	25 kDa	8	34%
410	Fatty acid synthase OS=Homo sapiens GN=FASN PE=1 SV=3	FAS_HUMAN	273 kDa	133	73%
411	Protocadherin Fat 2 OS=Homo sapiens GN=FAT2 PE=1 SV=2	FAT2_HUMAN	479 kDa	31	9.20%
412	Fibulin-1 OS=Homo sapiens GN=FBLN1 PE=1 SV=4	FBLN1_HUMAN	77 kDa	2	2.80%
413	EGF-containing fibulin-like extracellular matrix protein 1 OS=Homo sapiens GN=EFEMP1 PE=1 SV=2	FBLN3_HUMAN	55 kDa	9	29%
414	Fibulin-5 OS=Homo sapiens GN=FBLN5 PE=1 SV=1	FBLN5_HUMAN	50 kDa	2	4.90%
415	IgGFc-binding protein OS=Homo sapiens GN=FCGBP PE=1 SV=3	FCGBP_HUMAN	572 kDa	9	1.90%
416	GDP-L-fucose synthase OS=Homo sapiens GN=TSTA3 PE=1 SV=1	FCL_HUMAN	36 kDa	7	31%
417	Ficolin-2 OS=Homo sapiens GN=FCN2 PE=1 SV=2	FCN2_HUMAN	34 kDa	4	13%
418	Fermitin family homolog 2 OS=Homo sapiens GN=FERMT2 PE=1 SV=1	FERM2_HUMAN	78 kDa	5	9.10%
419	Alpha-2-HS-glycoprotein OS=Homo sapiens GN=AHSG PE=1 SV=1	FETUA_HUMAN	39 kDa	15	53%
420	Fibroblast growth factor-binding protein 1 OS=Homo sapiens GN=FGFBP1 PE=1 SV=1	FGFP1_HUMAN	26 kDa	4	25%
421	Complement factor H-related protein 1 OS=Homo sapiens GN=CFHR1 PE=1 SV=2	FHR1_HUMAN	38 kDa	2	9.70%
422	Fibrinogen alpha chain OS=Homo sapiens GN=FGA PE=1 SV=2	FIBA_HUMAN	95 kDa	23	30%
423	Fibrinogen beta chain OS=Homo sapiens GN=FGB PE=1 SV=2	FIBB_HUMAN	56 kDa	17	48%
424	Fibrinogen gamma chain OS=Homo sapiens GN=FGG PE=1 SV=3	FIBG_HUMAN	52 kDa	14	38%
425	Filaggrin OS=Homo sapiens GN=FLG PE=1 SV=3	FILA_HUMAN	435 kDa	4	1.50%
426	Fibronectin OS=Homo sapiens GN=FN1 PE=1 SV=4	FINC_HUMAN	263 kDa	49	30%
427	Peptidyl-prolyl cis-trans isomerase FKBP1A OS=Homo sapiens GN=FKBP1A PE=1 SV=2	FKB1A_HUMAN	12 kDa	2	25%
428	Peptidyl-prolyl cis-trans isomerase FKBP3 OS=Homo sapiens GN=FKBP3 PE=1 SV=1	FKBP3_HUMAN	25 kDa	2	10%
429	Peptidyl-prolyl cis-trans isomerase FKBP4 OS=Homo sapiens GN=FKBP4 PE=1 SV=3	FKBP4_HUMAN	52 kDa	16	39%
430	Filamin-B OS=Homo sapiens GN=FLNB PE=1 SV=2	FLNB_HUMAN	278 kDa	36	19%
431	Protein farnesyltransferase/geranylgeranyltransferase type-1 subunit alpha OS=Homo sapiens GN=FNTA PE=1 SV=1	FNTA_HUMAN	44 kDa	3	7.70%
432	Folate receptor alpha OS=Homo sapiens GN=FOLR1 PE=1 SV=3	FOLR1_HUMAN	30 kDa	9	43%
433	Farnesyl pyrophosphate synthase OS=Homo sapiens GN=FDPS PE=1 SV=4	FPPS_HUMAN	48 kDa	6	13%
434	FRAS1-related extracellular matrix protein 3 OS=Homo sapiens GN=FREM3 PE=2 SV=1	FREM3_HUMAN	238 kDa	16	9.20%

#	Proteins	Uniprot ID	Molecular Weight	Number of unique peptides	Percent of coverage
435	Ferritin heavy chain OS=Homo sapiens GN=FTH1 PE=1 SV=2	FRIH_HUMAN	21 kDa	4	23%
436	Fascin OS=Homo sapiens GN=FSCN1 PE=1 SV=3	FSCN1_HUMAN	55 kDa	3	7.10%
437	Follistatin-related protein 1 OS=Homo sapiens GN=FSTL1 PE=1 SV=1	FSTL1_HUMAN	35 kDa	21	65%
438	Tissue alpha-L-fucosidase OS=Homo sapiens GN=FUCA1 PE=1 SV=4	FUCO_HUMAN	54 kDa	8	19%
439	Plasma alpha-L-fucosidase OS=Homo sapiens GN=FUCA2 PE=1 SV=2	FUCO2_HUMAN	54 kDa	7	15%
440	Fumarate hydratase, mitochondrial OS=Homo sapiens GN=FH PE=1 SV=3	FUMH_HUMAN	55 kDa	3	6.90%
441	Alpha-(1,3)-fucosyltransferase OS=Homo sapiens GN=FUT6 PE=2 SV=1	FUT6_HUMAN	42 kDa	8	31%
442	G0/G1 switch regulatory protein 2 OS=Homo sapiens GN=G0S2 PE=1 SV=1	G0S2_HUMAN	11 kDa	2	27%
443	Glyceraldehyde-3-phosphate dehydrogenase OS=Homo sapiens GN=GAPDH PE=1 SV=3	G3P_HUMAN	36 kDa	20	74%
444	Glucose-6-phosphate 1-dehydrogenase OS=Homo sapiens GN=G6PD PE=1 SV=4	G6PD_HUMAN	59 kDa	8	19%
445	Glucose-6-phosphate isomerase OS=Homo sapiens GN=GPI PE=1 SV=4	G6PI_HUMAN	63 kDa	13	31%
446	UDP-glucose 4-epimerase OS=Homo sapiens GN=GALE PE=1 SV=2	GALE_HUMAN	38 kDa	10	52%
447	Polypeptide N-acetylgalactosaminyltransferase 1 OS=Homo sapiens GN=GALNT1 PE=1 SV=1	GALT1_HUMAN	64 kDa	2	4.30%
448	Polypeptide N-acetylgalactosaminyltransferase 2 OS=Homo sapiens GN=GALNT2 PE=1 SV=1	GALT2_HUMAN	65 kDa	15	32%
449	Polypeptide N-acetylgalactosaminyltransferase 3 OS=Homo sapiens GN=GALNT3 PE=2 SV=2	GALT3_HUMAN	73 kDa	3	7.30%
450	Polypeptide N-acetylgalactosaminyltransferase 5 OS=Homo sapiens GN=GALNT5 PE=1 SV=1	GALT5_HUMAN	106 kDa	2	2.80%
451	Neutral alpha-glucosidase AB OS=Homo sapiens GN=GANAB PE=1 SV=3	GANAB_HUMAN	107 kDa	21	29%
452	Golgi-associated plant pathogenesis-related protein 1 OS=Homo sapiens GN=GLIPR2 PE=1 SV=3	GAPR1_HUMAN	17 kDa	6	51%
453	Guanine nucleotide-binding protein G(I)/G(S)/G(T) subunit beta-1 OS=Homo sapiens GN=GNB1 PE=1 SV=3	GBB1_HUMAN	37 kDa	2	25%
454	Guanine nucleotide-binding protein G(I)/G(S)/G(T) subunit beta-2 OS=Homo sapiens GN=GNB2 PE=1 SV=3	GBB2_HUMAN	37 kDa	8	31%
455	Guanine nucleotide-binding protein subunit beta-4 OS=Homo sapiens GN=GNB4 PE=1 SV=3	GBB4_HUMAN	38 kDa	2	21%
456	Guanine nucleotide-binding protein G(I)/G(S)/G(O) subunit gamma-12 OS=Homo sapiens GN=GNG12 PE=1 SV=3	GBG12_HUMAN	8 kDa	4	56%
457	Guanine nucleotide-binding protein subunit beta-2-like 1 OS=Homo sapiens GN=GNB2L1 PE=1 SV=3	GBLP_HUMAN	35 kDa	2	6.30%
458	Interferon-induced guanylate-binding protein 2 OS=Homo sapiens GN=GBP2 PE=2 SV=3	GBP2_HUMAN	67 kDa	2	4.60%
459	Translational activator GCN1 OS=Homo sapiens GN=GCN1L1 PE=1 SV=6	GCN1L_HUMAN	293 kDa	4	1.60%
460	Rab GDP dissociation inhibitor alpha OS=Homo sapiens GN=GDI1 PE=1 SV=2	GDIA_HUMAN	51 kDa	6	38%
461	Rab GDP dissociation inhibitor beta OS=Homo sapiens GN=GDI2 PE=1 SV=2	GDIB_HUMAN	51 kDa	30	72%
462	Rho GDP-dissociation inhibitor 1 OS=Homo sapiens GN=ARHGDIA PE=1 SV=3	GDIR1_HUMAN	23 kDa	6	58%
463	Rho GDP-dissociation inhibitor 2 OS=Homo sapiens GN=ARHGDIB PE=1 SV=3	GDIR2_HUMAN	23 kDa	2	10.00%
464	Gelsolin OS=Homo sapiens GN=GSN PE=1 SV=1	GELS_HUMAN	86 kDa	26	42%
465	Gephyrin OS=Homo sapiens GN=GPHN PE=1 SV=1	GEPH_HUMAN	80 kDa	9	16%
466	Glucosamine--fructose-6-phosphate aminotransferase [isomerizing] 1 OS=Homo sapiens GN=GFPT1 PE=1 SV=3	GFPT1_HUMAN	79 kDa	27	41%
467	GTP cyclohydrolase 1 feedback regulatory protein OS=Homo sapiens GN=GCHFR PE=1 SV=3	GFRP_HUMAN	10 kDa	3	40%
468	Gamma-glutamylcyclotransferase OS=Homo sapiens GN=GGCT PE=1 SV=1	GGCT_HUMAN	21 kDa	5	31%
469	Gamma-glutamyl hydrolase OS=Homo sapiens GN=GGH PE=1 SV=2	GGH_HUMAN	36 kDa	4	14%
470	Gamma-glutamyltranspeptidase 1 OS=Homo sapiens GN=GGT1 PE=1 SV=2	GGT1_HUMAN	61 kDa	14	25%
471	Gamma-glutamyltransferase 5 OS=Homo sapiens GN=GGT5 PE=1 SV=2	GGT5_HUMAN	62 kDa	2	4.60%
472	Gamma-glutamyltransferase 6 OS=Homo sapiens GN=GGT6 PE=2 SV=2	GGT6_HUMAN	51 kDa	3	11%
473	Gamma-interferon-inducible lysosomal thiol reductase OS=Homo sapiens GN=IFI30 PE=1 SV=3	GILT_HUMAN	28 kDa	2	8.80%

#	Proteins	Uniprot ID	Molecular Weight	Number of unique peptides	Percent of coverage
474	PDZ domain-containing protein GIPC1 OS=Homo sapiens GN=GIPC1 PE=1 SV=2	GIPC1_HUMAN	36 kDa	5	17%
475	Glucosylceramidase OS=Homo sapiens GN=GBA PE=1 SV=3	GLCM_HUMAN	60 kDa	8	18%
476	1,4-alpha-glucan-branching enzyme OS=Homo sapiens GN=GBE1 PE=1 SV=2	GLGB_HUMAN	80 kDa	2	2.70%
477	Glutamine synthetase OS=Homo sapiens GN=GLUL PE=1 SV=4	GLNA_HUMAN	42 kDa	4	12%
478	Hydroxyacylglutathione hydrolase, mitochondrial OS=Homo sapiens GN=HAGH PE=1 SV=2	GLO2_HUMAN	34 kDa	4	16%
479	Glyoxalase domain-containing protein 4 OS=Homo sapiens GN=GLOD4 PE=1 SV=1	GLOD4_HUMAN	35 kDa	8	30%
480	Golgi phosphoprotein 3-like OS=Homo sapiens GN=GOLPH3L PE=1 SV=1	GLP3L_HUMAN	33 kDa	3	15%
481	Glycerol kinase OS=Homo sapiens GN=GK PE=1 SV=3	GLPK_HUMAN	61 kDa	5	10%
482	Glutaredoxin-1 OS=Homo sapiens GN=GLRX PE=1 SV=2	GLRX1_HUMAN	12 kDa	8	78%
483	Glutaredoxin-3 OS=Homo sapiens GN=GLRX3 PE=1 SV=2	GLRX3_HUMAN	37 kDa	5	16%
484	Glycolipid transfer protein OS=Homo sapiens GN=GLTP PE=1 SV=3	GLTP_HUMAN	24 kDa	2	18%
485	Glucosidase 2 subunit beta OS=Homo sapiens GN=PRKCSH PE=1 SV=2	GLU2B_HUMAN	59 kDa	11	24%
486	Serine hydroxymethyltransferase, cytosolic OS=Homo sapiens GN=SHMT1 PE=1 SV=1	GLYC_HUMAN	53 kDa	14	35%
487	Glycogenin-1 OS=Homo sapiens GN=GYG1 PE=1 SV=4	GLYG_HUMAN	39 kDa	2	6.90%
488	GDP-mannose 4,6 dehydratase OS=Homo sapiens GN=GMDS PE=1 SV=1	GMDS_HUMAN	42 kDa	16	46%
489	Glia maturation factor beta OS=Homo sapiens GN=GMFB PE=1 SV=2	GMFB_HUMAN	17 kDa	3	25%
490	Mannose-1-phosphate guanyltransferase alpha OS=Homo sapiens GN=GMPPA PE=1 SV=1	GMPPA_HUMAN	46 kDa	8	22%
491	Mannose-1-phosphate guanyltransferase beta OS=Homo sapiens GN=GMPPB PE=1 SV=2	GMPPB_HUMAN	40 kDa	9	34%
492	GMP reductase 2 OS=Homo sapiens GN=GMPR2 PE=1 SV=1	GMPR2_HUMAN	38 kDa	5	21%
493	Glucosamine 6-phosphate N-acetyltransferase OS=Homo sapiens GN=GNPNAT1 PE=1 SV=1	GNA1_HUMAN	21 kDa	2	16%
494	Guanine nucleotide-binding protein G(k) subunit alpha OS=Homo sapiens GN=GNAI3 PE=1 SV=3	GNAI3_HUMAN	41 kDa	2	10%
495	Guanine nucleotide-binding protein G(q) subunit alpha OS=Homo sapiens GN=GNAQ PE=1 SV=4	GNAQ_HUMAN	42 kDa	6	19%
496	Guanine nucleotide-binding protein G(s) subunit alpha isoforms XLas OS=Homo sapiens GN=GNAS PE=1 SV=2	GNAS1_HUMAN (+1)	111 kDa	8	10%
497	Glucosamine-6-phosphate isomerase 1 OS=Homo sapiens GN=GNPDA1 PE=1 SV=1	GNPI1_HUMAN	33 kDa	4	17%
498	N-acetylglucosamine-1-phosphotransferase subunit gamma OS=Homo sapiens GN=GNPTG PE=1 SV=1	GNPTG_HUMAN	34 kDa	4	19%
499	N-acetylglucosamine-6-sulfatase OS=Homo sapiens GN=GNS PE=1 SV=3	GNS_HUMAN	62 kDa	8	15%
500	Golgi integral membrane protein 4 OS=Homo sapiens GN=GOLIM4 PE=1 SV=1	GOLI4_HUMAN	82 kDa	3	4.60%
501	Golgi membrane protein 1 OS=Homo sapiens GN=GOLM1 PE=1 SV=1	GOLM1_HUMAN	45 kDa	17	45%
502	Golgi reassembly-stacking protein 2 OS=Homo sapiens GN=GORASP2 PE=1 SV=3	GORS2_HUMAN	47 kDa	2	4.40%
503	G-protein coupled receptor 126 OS=Homo sapiens GN=GPR126 PE=1 SV=3	GP126_HUMAN	137 kDa	12	10%
504	G-protein coupled receptor family C group 5 member B OS=Homo sapiens GN=GPRC5B PE=2 SV=2	GPC5B_HUMAN	45 kDa	4	16%
505	G-protein coupled receptor family C group 5 member C OS=Homo sapiens GN=GPRC5C PE=1 SV=2	GPC5C_HUMAN	48 kDa	3	7.90%
506	Glycerol-3-phosphate dehydrogenase 1-like protein OS=Homo sapiens GN=GPD1L PE=1 SV=1	GPD1L_HUMAN	38 kDa	4	14%
507	Glycerol-3-phosphate dehydrogenase [NAD+], cytoplasmic OS=Homo sapiens GN=GPD1 PE=1 SV=4	GPDA_HUMAN	38 kDa	10	33%
508	Transmembrane glycoprotein NMB OS=Homo sapiens GN=GPNMB PE=1 SV=2	GPNMB_HUMAN	64 kDa	3	7.90%
509	G-protein coupled receptor 56 OS=Homo sapiens GN=GPR56 PE=1 SV=2	GPR56_HUMAN	78 kDa	4	8.80%
510	Glutathione peroxidase 3 OS=Homo sapiens GN=GPX3 PE=1 SV=2	GPX3_HUMAN	26 kDa	8	38%
511	Phospholipid hydroperoxide glutathione peroxidase, mitochondrial OS=Homo sapiens GN=GPX4 PE=1 SV=3	GPX4_HUMAN	22 kDa	9	46%

#	Proteins	Uniprot ID	Molecular Weight	Number of unique peptides	Percent of coverage
512	Growth factor receptor-bound protein 2 OS=Homo sapiens GN=GRB2 PE=1 SV=1	GRB2_HUMAN	25 kDa	2	8.80%
513	Gremlin-2 OS=Homo sapiens GN=GREM2 PE=1 SV=1	GREM2_HUMAN	19 kDa	4	27%
514	Glyoxylate reductase/hydroxypyruvate reductase OS=Homo sapiens GN=GRHPR PE=1 SV=1	GRHPR_HUMAN	36 kDa	4	18%
515	Granulins OS=Homo sapiens GN=GRN PE=1 SV=2	GRN_HUMAN	64 kDa	19	46%
516	78 kDa glucose-regulated protein OS=Homo sapiens GN=HSPA5 PE=1 SV=2	GRP78_HUMAN	72 kDa	34	49%
517	Glutamate--cysteine ligase catalytic subunit OS=Homo sapiens GN=GCLC PE=1 SV=2	GSH1_HUMAN	73 kDa	7	12%
518	Glutathione synthetase OS=Homo sapiens GN=GSS PE=1 SV=1	GSHB_HUMAN	52 kDa	20	52%
519	Golgi apparatus protein 1 OS=Homo sapiens GN=GLG1 PE=1 SV=2	GSLG1_HUMAN	135 kDa	4	4.70%
520	Glutathione S-transferase theta-2 OS=Homo sapiens GN=GSTT2 PE=1 SV=1	GST2_HUMAN (+1)	28 kDa	4	22%
521	Glutathione S-transferase A1 OS=Homo sapiens GN=GSTA1 PE=1 SV=3	GSTA1_HUMAN (+1)	26 kDa	2	8.60%
522	Glutathione S-transferase kappa 1 OS=Homo sapiens GN=GSTK1 PE=1 SV=3	GSTK1_HUMAN	25 kDa	3	12%
523	Glutathione S-transferase Mu 3 OS=Homo sapiens GN=GSTM3 PE=1 SV=3	GSTM3_HUMAN	27 kDa	4	21%
524	Glutathione S-transferase omega-1 OS=Homo sapiens GN=GSTO1 PE=1 SV=2	GSTO1_HUMAN	28 kDa	5	21%
525	Glutathione S-transferase P OS=Homo sapiens GN=GSTP1 PE=1 SV=2	GSTP1_HUMAN	23 kDa	3	18%
526	Glutathione S-transferase theta-1 OS=Homo sapiens GN=GSTT1 PE=1 SV=4	GSTT1_HUMAN	27 kDa	4	23%
527	GTP-binding protein 5 OS=Homo sapiens GN=GTPBP5 PE=2 SV=1	GTPB5_HUMAN	44 kDa	2	5.70%
528	Histone H2A type 1-B/E OS=Homo sapiens GN=HIST1H2AB PE=1 SV=2	H2A1B_HUMAN (+9)	14 kDa	2	22%
529	Histone H2B type 1-C/E/F/G/I OS=Homo sapiens GN=HIST1H2BC PE=1 SV=4	H2B1C_HUMAN (+6)	14 kDa	5	41%
530	Histone H3.1t OS=Homo sapiens GN=HIST3H3 PE=1 SV=3	H31T_HUMAN (+2)	16 kDa	2	5.90%
531	Histone H4 OS=Homo sapiens GN=HIST1H4A PE=1 SV=2	H4_HUMAN	11 kDa	5	40%
532	28 kDa heat- and acid-stable phosphoprotein OS=Homo sapiens GN=PDAP1 PE=1 SV=1	HAP28_HUMAN	21 kDa	3	14%
533	Hemoglobin subunit alpha OS=Homo sapiens GN=HBA1 PE=1 SV=2	HBA_HUMAN	15 kDa	4	40%
534	Hemoglobin subunit beta OS=Homo sapiens GN=HBB PE=1 SV=2	HBB_HUMAN	16 kDa	6	48%
535	Haloacid dehalogenase-like hydrolase domain-containing protein 2 OS=Homo sapiens GN=HDHD2 PE=1 SV=1	HDHD2_HUMAN	29 kDa	3	18%
536	Heme-binding protein 1 OS=Homo sapiens GN=HEBP1 PE=1 SV=1	HEBP1_HUMAN	21 kDa	6	40%
537	Heme-binding protein 2 OS=Homo sapiens GN=HEBP2 PE=1 SV=1	HEBP2_HUMAN	23 kDa	7	43%
538	Delta-aminolevulinic acid dehydratase OS=Homo sapiens GN=ALAD PE=1 SV=1	HEM2_HUMAN	36 kDa	8	30%
539	Hemopexin OS=Homo sapiens GN=HPX PE=1 SV=2	HEMO_HUMAN	52 kDa	18	51%
540	Heparin cofactor 2 OS=Homo sapiens GN=SERPIND1 PE=1 SV=3	HEP2_HUMAN	57 kDa	10	20%
541	Beta-hexosaminidase subunit alpha OS=Homo sapiens GN=HEXA PE=1 SV=2	HEXA_HUMAN	61 kDa	8	19%
542	Beta-hexosaminidase subunit beta OS=Homo sapiens GN=HEXB PE=1 SV=3	HEXB_HUMAN	63 kDa	17	33%
543	HLA class II histocompatibility antigen gamma chain OS=Homo sapiens GN=CD74 PE=1 SV=3	HG2A_HUMAN	34 kDa	2	8.80%
544	3-hydroxyisobutyryl-CoA hydrolase, mitochondrial OS=Homo sapiens GN=HIBCH PE=1 SV=2	HIBCH_HUMAN	43 kDa	5	16%
545	Histidine triad nucleotide-binding protein 1 OS=Homo sapiens GN=HINT1 PE=1 SV=2	HINT1_HUMAN	14 kDa	3	24%
546	HLA class I histocompatibility antigen, alpha chain E OS=Homo sapiens GN=HLA-E PE=1 SV=3	HLAE_HUMAN	40 kDa	2	11%
547	HLA class I histocompatibility antigen, alpha chain G OS=Homo sapiens GN=HLA-G PE=1 SV=1	HLAG_HUMAN	38 kDa	2	11%
548	Histamine N-methyltransferase OS=Homo sapiens GN=HNMT PE=1 SV=1	HNMT_HUMAN	33 kDa	4	16%
549	Heterogeneous nuclear ribonucleoprotein K OS=Homo sapiens GN=HNRNPK PE=1 SV=1	HNRPK_HUMAN	51 kDa	9	22%
550	Heterogeneous nuclear ribonucleoprotein Q OS=Homo sapiens GN=SYNCRIP PE=1 SV=2	HNRPQ_HUMAN	70 kDa	11	18%

#	Proteins	Uniprot ID	Molecular Weight	Number of unique peptides	Percent of coverage
551	Hyaluronan and proteoglycan link protein 3 OS=Homo sapiens GN=HAPLN3 PE=2 SV=1	HPLN3_HUMAN	41 kDa	17	56%
552	Hypoxanthine-guanine phosphoribosyltransferase OS=Homo sapiens GN=HPRT1 PE=1 SV=2	HPRT_HUMAN	25 kDa	6	29%
553	Haptoglobin OS=Homo sapiens GN=HP PE=1 SV=1	HPT_HUMAN	45 kDa	30	69%
554	Haptoglobin-related protein OS=Homo sapiens GN=HPR PE=1 SV=2	HPTR_HUMAN	39 kDa	2	42%
555	Histidine-rich glycoprotein OS=Homo sapiens GN=HRG PE=1 SV=1	HRG_HUMAN	60 kDa	3	6.70%
556	Heat shock protein 105 kDa OS=Homo sapiens GN=HSPH1 PE=1 SV=1	HS105_HUMAN	97 kDa	3	5.00%
557	Heat shock protein HSP 90-alpha OS=Homo sapiens GN=HSP90AA1 PE=1 SV=5	HS90A_HUMAN	85 kDa	39	53%
558	Heat shock protein HSP 90-beta OS=Homo sapiens GN=HSP90AB1 PE=1 SV=4	HS90B_HUMAN	83 kDa	18	47%
559	Heat shock 70 kDa protein 13 OS=Homo sapiens GN=HSPA13 PE=1 SV=1	HSP13_HUMAN	52 kDa	10	26%
560	Heat shock 70 kDa protein 1A/1B OS=Homo sapiens GN=HSPA1A PE=1 SV=5	HSP71_HUMAN	70 kDa	19	45%
561	Heat shock 70 kDa protein 4 OS=Homo sapiens GN=HSPA4 PE=1 SV=4	HSP74_HUMAN	94 kDa	8	13%
562	Heat shock cognate 71 kDa protein OS=Homo sapiens GN=HSPA8 PE=1 SV=1	HSP7C_HUMAN	71 kDa	28	53%
563	Heat shock protein beta-1 OS=Homo sapiens GN=HSPB1 PE=1 SV=2	HSPB1_HUMAN	23 kDa	13	85%
564	E3 ubiquitin-protein ligase HUWE1 OS=Homo sapiens GN=HUWE1 PE=1 SV=3	HUWE1_HUMAN	482 kDa	4	1.10%
565	Ig heavy chain V-I region HG3 OS=Homo sapiens PE=4 SV=1	HV102_HUMAN	13 kDa	2	28%
566	Ig heavy chain V-I region V35 OS=Homo sapiens PE=1 SV=1	HV103_HUMAN	13 kDa	4	38%
567	Ig heavy chain V-II region WAH OS=Homo sapiens PE=1 SV=1	HV206_HUMAN	14 kDa	2	13%
568	Ig heavy chain V-II region SESS OS=Homo sapiens PE=2 SV=1	HV208_HUMAN	16 kDa	3	14%
569	Ig heavy chain V-II region ARH-77 OS=Homo sapiens PE=4 SV=1	HV209_HUMAN	16 kDa	2	17%
570	Ig heavy chain V-III region TRO OS=Homo sapiens PE=1 SV=1	HV301_HUMAN	13 kDa	3	24%
571	Ig heavy chain V-III region WEA OS=Homo sapiens PE=1 SV=1	HV302_HUMAN	12 kDa	2	36%
572	Ig heavy chain V-III region VH26 OS=Homo sapiens PE=1 SV=1	HV303_HUMAN	13 kDa	3	22%
573	Ig heavy chain V-III region BUT OS=Homo sapiens PE=1 SV=1	HV306_HUMAN	12 kDa	2	36%
574	Ig heavy chain V-III region CAM OS=Homo sapiens PE=1 SV=1	HV307_HUMAN	14 kDa	3	28%
575	Ig heavy chain V-III region GA OS=Homo sapiens PE=1 SV=1	HV308_HUMAN	13 kDa	3	25%
576	Ig heavy chain V-III region NIE OS=Homo sapiens PE=1 SV=1	HV309_HUMAN	13 kDa	3	26%
577	Ig heavy chain V-III region HIL OS=Homo sapiens PE=1 SV=1	HV310_HUMAN	14 kDa	2	16%
578	Ig heavy chain V-III region GAL OS=Homo sapiens PE=1 SV=1	HV320_HUMAN	13 kDa	4	41%
579	Epoxide hydrolase 2 OS=Homo sapiens GN=EPHX2 PE=1 SV=2	HYES_HUMAN	63 kDa	2	7.70%
580	Hypoxia up-regulated protein 1 OS=Homo sapiens GN=HYOU1 PE=1 SV=1	HYOU1_HUMAN	111 kDa	22	28%
581	Huntingtin-interacting protein K OS=Homo sapiens GN=HYPK PE=1 SV=2	HYPK_HUMAN	15 kDa	2	20%
582	Interleukin-18-binding protein OS=Homo sapiens GN=IL18BP PE=1 SV=2	I18BP_HUMAN	21 kDa	5	24%
583	Insulin-like growth factor-binding protein 2 OS=Homo sapiens GN=IGFBP2 PE=1 SV=2	IBP2_HUMAN	35 kDa	18	67%
584	Insulin-like growth factor-binding protein 7 OS=Homo sapiens GN=IGFBP7 PE=1 SV=1	IBP7_HUMAN	29 kDa	2	12%
585	Plasma protease C1 inhibitor OS=Homo sapiens GN=SERPING1 PE=1 SV=2	IC1_HUMAN	55 kDa	16	29%
586	Calpastatin OS=Homo sapiens GN=CAST PE=1 SV=4	ICAL_HUMAN	77 kDa	6	13%
587	Intercellular adhesion molecule 1 OS=Homo sapiens GN=ICAM1 PE=1 SV=2	ICAM1_HUMAN	58 kDa	9	22%
588	Isocitrate dehydrogenase [NADP] cytoplasmic OS=Homo sapiens GN=IDH1 PE=1 SV=2	IDHC_HUMAN	47 kDa	27	64%
589	Isocitrate dehydrogenase [NADP], mitochondrial OS=Homo sapiens GN=IDH2 PE=1 SV=2	IDHP_HUMAN	51 kDa	3	10.00%
590	Isopentenyl-diphosphate Delta-isomerase 1 OS=Homo sapiens GN=IDI1 PE=1 SV=2	IDI1_HUMAN	26 kDa	6	28%

#	Proteins	Uniprot ID	Molecular Weight	Number of unique peptides	Percent of coverage
591	Alpha-L-iduronidase OS=Homo sapiens GN=IDUA PE=1 SV=2	IDUA_HUMAN	73 kDa	4	9.60%
592	Eukaryotic translation initiation factor 1A, X-chromosomal OS=Homo sapiens GN=EIF1AX PE=1 SV=2	IF1AX_HUMAN (+1)	16 kDa	2	15%
593	Eukaryotic translation initiation factor 2 subunit 1 OS=Homo sapiens GN=EIF2S1 PE=1 SV=3	IF2A_HUMAN	36 kDa	3	9.50%
594	Eukaryotic translation initiation factor 2 subunit 3 OS=Homo sapiens GN=EIF2S3 PE=1 SV=3	IF2G_HUMAN	51 kDa	6	18%
595	Eukaryotic initiation factor 4A-I OS=Homo sapiens GN=EIF4A1 PE=1 SV=1	IF4A1_HUMAN	46 kDa	11	31%
596	Eukaryotic initiation factor 4A-II OS=Homo sapiens GN=EIF4A2 PE=1 SV=2	IF4A2_HUMAN	46 kDa	5	27%
597	Eukaryotic translation initiation factor 4B OS=Homo sapiens GN=EIF4B PE=1 SV=2	IF4B_HUMAN	69 kDa	5	10%
598	Eukaryotic translation initiation factor 4 gamma 1 OS=Homo sapiens GN=EIF4G1 PE=1 SV=4	IF4G1_HUMAN	175 kDa	9	6.80%
599	Eukaryotic translation initiation factor 4 gamma 2 OS=Homo sapiens GN=EIF4G2 PE=1 SV=1	IF4G2_HUMAN	102 kDa	2	1.80%
600	Eukaryotic translation initiation factor 4H OS=Homo sapiens GN=EIF4H PE=1 SV=5	IF4H_HUMAN	27 kDa	3	11%
601	Eukaryotic translation initiation factor 5 OS=Homo sapiens GN=EIF5 PE=1 SV=2	IF5_HUMAN	49 kDa	2	5.60%
602	Eukaryotic translation initiation factor 5A-1 OS=Homo sapiens GN=EIF5A PE=1 SV=2	IF5A1_HUMAN	17 kDa	8	51%
603	Ig alpha-1 chain C region OS=Homo sapiens GN=IGHA1 PE=1 SV=2	IGHA1_HUMAN	38 kDa	21	83%
604	Ig alpha-2 chain C region OS=Homo sapiens GN=IGHA2 PE=1 SV=3	IGHA2_HUMAN	37 kDa	5	74%
605	Ig delta chain C region OS=Homo sapiens GN=IGHD PE=1 SV=2	IGHD_HUMAN	42 kDa	7	28%
606	Ig gamma-1 chain C region OS=Homo sapiens GN=IGHG1 PE=1 SV=1	IGHG1_HUMAN	36 kDa	14	56%
607	Ig gamma-2 chain C region OS=Homo sapiens GN=IGHG2 PE=1 SV=2	IGHG2_HUMAN	36 kDa	8	42%
608	Ig gamma-3 chain C region OS=Homo sapiens GN=IGHG3 PE=1 SV=2	IGHG3_HUMAN	41 kDa	2	29%
609	Ig gamma-4 chain C region OS=Homo sapiens GN=IGHG4 PE=1 SV=1	IGHG4_HUMAN	36 kDa	4	43%
610	Ig mu chain C region OS=Homo sapiens GN=IGHM PE=1 SV=3	IGHM_HUMAN	49 kDa	21	53%
611	Immunoglobulin J chain OS=Homo sapiens GN=IGJ PE=1 SV=4	IGJ_HUMAN	18 kDa	11	73%
612	Ig kappa chain C region OS=Homo sapiens GN=IGKC PE=1 SV=1	IGKC_HUMAN	12 kDa	8	86%
613	Immunoglobulin lambda-like polypeptide 1 OS=Homo sapiens GN=IGLL1 PE=1 SV=1	IGLL1_HUMAN	23 kDa	2	18%
614	Immunoglobulin lambda-like polypeptide 5 OS=Homo sapiens GN=IGLL5 PE=2 SV=2	IGLL5_HUMAN	23 kDa	4	50%
615	Immunoglobulin superfamily member 8 OS=Homo sapiens GN=IGSF8 PE=1 SV=1	IGSF8_HUMAN	65 kDa	4	9.00%
616	Interleukin-19 OS=Homo sapiens GN=IL19 PE=1 SV=2	IL19_HUMAN	20 kDa	5	31%
617	Interleukin-6 receptor subunit beta OS=Homo sapiens GN=IL6ST PE=1 SV=2	IL6RB_HUMAN	104 kDa	2	1.70%
618	Leukocyte elastase inhibitor OS=Homo sapiens GN=SERPINB1 PE=1 SV=1	ILEU_HUMAN	43 kDa	6	17%
619	Importin subunit beta-1 OS=Homo sapiens GN=KPNB1 PE=1 SV=2	IMB1_HUMAN	97 kDa	13	16%
620	Inosine-5'-monophosphate dehydrogenase 2 OS=Homo sapiens GN=IMPDH2 PE=1 SV=2	IMDH2_HUMAN	56 kDa	5	15%
621	Inositol monophosphatase 3 OS=Homo sapiens GN=IMPAD1 PE=1 SV=1	IMPA3_HUMAN	39 kDa	3	7.80%
622	Interferon-induced 35 kDa protein OS=Homo sapiens GN=IFI35 PE=1 SV=5	IN35_HUMAN	32 kDa	2	7.00%
623	InaD-like protein OS=Homo sapiens GN=INADL PE=1 SV=3	INADL_HUMAN	196 kDa	2	1.20%
624	Integrator complex subunit 3 OS=Homo sapiens GN=INTS3 PE=1 SV=1	INT3_HUMAN	118 kDa	2	3.70%
625	Importin-4 OS=Homo sapiens GN=IPO4 PE=1 SV=2	IPO4_HUMAN	119 kDa	2	3.00%
626	Importin-5 OS=Homo sapiens GN=IPO5 PE=1 SV=4	IPO5_HUMAN	124 kDa	11	15%
627	Importin-7 OS=Homo sapiens GN=IPO7 PE=1 SV=1	IPO7_HUMAN	120 kDa	2	2.10%
628	Protein phosphatase inhibitor 2 OS=Homo sapiens GN=PPP1R2 PE=1 SV=2	IPP2_HUMAN	23 kDa	6	38%
629	Plasma serine protease inhibitor OS=Homo sapiens GN=SERPINA5 PE=1 SV=3	IPSP_HUMAN	46 kDa	8	23%
630	Inorganic pyrophosphatase OS=Homo sapiens GN=PPA1 PE=1 SV=2	IPYR_HUMAN	33 kDa	15	64%

#	Proteins	Uniprot ID	Molecular Weight	Number of unique peptides	Percent of coverage
631	Ras GTPase-activating-like protein IQGAP1 OS=Homo sapiens GN=IQGAP1 PE=1 SV=1	IQGA1_HUMAN	189 kDa	33	26%
632	Ras GTPase-activating-like protein IQGAP2 OS=Homo sapiens GN=IQGAP2 PE=1 SV=4	IQGA2_HUMAN	181 kDa	3	2.20%
633	Immunoglobulin superfamily containing leucine-rich repeat protein OS=Homo sapiens GN=ISLR PE=1 SV=1	ISLR_HUMAN	46 kDa	5	15%
634	Isochorismatase domain-containing protein 1 OS=Homo sapiens GN=ISOC1 PE=1 SV=3	ISOC1_HUMAN	32 kDa	5	22%
635	IST1 homolog OS=Homo sapiens GN=KIAA0174 PE=1 SV=1	IST1_HUMAN	40 kDa	4	9.90%
636	Integrin beta-like protein 1 OS=Homo sapiens GN=ITGBL1 PE=2 SV=1	ITGBL_HUMAN	54 kDa	2	5.90%
637	Inter-alpha-trypsin inhibitor heavy chain H1 OS=Homo sapiens GN=ITIH1 PE=1 SV=3	ITIH1_HUMAN	101 kDa	17	22%
638	Inter-alpha-trypsin inhibitor heavy chain H2 OS=Homo sapiens GN=ITIH2 PE=1 SV=2	ITIH2_HUMAN	106 kDa	24	26%
639	Inter-alpha-trypsin inhibitor heavy chain H3 OS=Homo sapiens GN=ITIH3 PE=1 SV=2	ITIH3_HUMAN	100 kDa	4	5.70%
640	Inter-alpha-trypsin inhibitor heavy chain H4 OS=Homo sapiens GN=ITIH4 PE=1 SV=4	ITIH4_HUMAN	103 kDa	33	42%
641	Keratin, type I cytoskeletal 10 OS=Homo sapiens GN=KRT10 PE=1 SV=6	K1C10_HUMAN	59 kDa	10	18%
642	Keratin, type I cytoskeletal 19 OS=Homo sapiens GN=KRT19 PE=1 SV=4	K1C19_HUMAN	44 kDa	6	17%
643	Keratin, type I cytoskeletal 9 OS=Homo sapiens GN=KRT9 PE=1 SV=3	K1C9_HUMAN	62 kDa	11	28%
644	Keratin, type II cytoskeletal 2 epidermal OS=Homo sapiens GN=KRT2 PE=1 SV=2	K22E_HUMAN	65 kDa	12	27%
645	Keratin, type II cytoskeletal 1 OS=Homo sapiens GN=KRT1 PE=1 SV=6	K2C1_HUMAN	66 kDa	24	41%
646	Keratin, type II cytoskeletal 7 OS=Homo sapiens GN=KRT7 PE=1 SV=5	K2C7_HUMAN	51 kDa	4	12%
647	Keratin, type II cytoskeletal 8 OS=Homo sapiens GN=KRT8 PE=1 SV=7	K2C8_HUMAN	54 kDa	6	15%
648	6-phosphofructokinase, liver type OS=Homo sapiens GN=PFKL PE=1 SV=6	K6PL_HUMAN	85 kDa	13	21%
649	Adenylate kinase isoenzyme 1 OS=Homo sapiens GN=AK1 PE=1 SV=3	KAD1_HUMAN	22 kDa	11	66%
650	Kallistatin OS=Homo sapiens GN=SERPINA4 PE=1 SV=3	KAIN_HUMAN	49 kDa	8	24%
651	cAMP-dependent protein kinase type II-alpha regulatory subunit OS=Homo sapiens GN=PRKAR2A PE=1 SV=2	KAP2_HUMAN	46 kDa	9	25%
652	cAMP-dependent protein kinase type II-beta regulatory subunit OS=Homo sapiens GN=PRKAR2B PE=1 SV=3	KAP3_HUMAN	46 kDa	2	11%
653	cAMP-dependent protein kinase catalytic subunit alpha OS=Homo sapiens GN=PRKACA PE=1 SV=2	KAPCA_HUMAN	41 kDa	4	13%
654	Kynurenine--oxoglutarate transaminase 3 OS=Homo sapiens GN=CCBL2 PE=1 SV=1	KAT3_HUMAN	51 kDa	4	9.90%
655	Casein kinase I isoform alpha OS=Homo sapiens GN=CSNK1A1 PE=1 SV=2	KC1A_HUMAN	39 kDa	3	8.90%
656	BTB/POZ domain-containing protein KCTD14 OS=Homo sapiens GN=KCTD14 PE=2 SV=2	KCD14_HUMAN	30 kDa	6	31%
657	UMP-CMP kinase OS=Homo sapiens GN=CMPK1 PE=1 SV=3	KCY_HUMAN	22 kDa	15	64%
658	Kinesin-like protein KIF27 OS=Homo sapiens GN=KIF27 PE=2 SV=1	KIF27_HUMAN	160 kDa	2	1.20%
659	Mevalonate kinase OS=Homo sapiens GN=MVK PE=1 SV=1	KIME_HUMAN	42 kDa	2	5.80%
660	Kinesin-1 heavy chain OS=Homo sapiens GN=KIF5B PE=1 SV=1	KINH_HUMAN	110 kDa	6	6.60%
661	Kallikrein-6 OS=Homo sapiens GN=KLK6 PE=1 SV=1	KLK6_HUMAN	27 kDa	12	60%
662	Kallikrein-8 OS=Homo sapiens GN=KLK8 PE=1 SV=1	KLK8_HUMAN	28 kDa	2	11%
663	Plasma kallikrein OS=Homo sapiens GN=KLKB1 PE=1 SV=1	KLKB1_HUMAN	71 kDa	2	3.80%
664	Kininogen-1 OS=Homo sapiens GN=KNG1 PE=1 SV=2	KNG1_HUMAN	72 kDa	24	40%
665	Phosphoribosyl pyrophosphate synthase-associated protein 1 OS=Homo sapiens GN=PRPSAP1 PE=1 SV=2	KPRA_HUMAN	39 kDa	2	6.20%
666	Pyruvate kinase isozymes M1/M2 OS=Homo sapiens GN=PKM2 PE=1 SV=4	KPYM_HUMAN	58 kDa	26	66%
667	Kinectin OS=Homo sapiens GN=KTN1 PE=1 SV=1	KTN1_HUMAN	156 kDa	9	8.40%
668	Ig kappa chain V-I region DEE OS=Homo sapiens PE=1 SV=1	KV105_HUMAN	12 kDa	3	22%
669	Ig kappa chain V-I region EU OS=Homo sapiens PE=1 SV=1	KV106_HUMAN	12 kDa	3	32%
670	Ig kappa chain V-I region Kue OS=Homo sapiens PE=1 SV=1	KV112_HUMAN	12 kDa	2	19%

**#**	**Proteins**	**Uniprot ID**	**Molecular Weight**	**Number of unique peptides**	**Percent of coverage**
671	Ig kappa chain V-I region OU OS=Homo sapiens PE=1 SV=1	KV114_HUMAN	12 kDa	3	22%
672	Ig kappa chain V-I region Rei OS=Homo sapiens PE=1 SV=1	KV115_HUMAN	12 kDa	2	44%
673	Ig kappa chain V-I region Roy OS=Homo sapiens PE=1 SV=1	KV116_HUMAN	12 kDa	2	31%
674	Ig kappa chain V-I region WEA OS=Homo sapiens PE=1 SV=1	KV118_HUMAN	12 kDa	3	36%
675	Ig kappa chain V-I region Wes OS=Homo sapiens PE=1 SV=1	KV119_HUMAN	12 kDa	2	22%
676	Ig kappa chain V-I region Mev OS=Homo sapiens PE=1 SV=1	KV120_HUMAN	12 kDa	2	31%
677	Ig kappa chain V-II region MIL OS=Homo sapiens PE=1 SV=1	KV203_HUMAN	12 kDa	4	39%
678	Ig kappa chain V-II region TEW OS=Homo sapiens PE=1 SV=1	KV204_HUMAN	12 kDa	2	39%
679	Ig kappa chain V-II region RPMI 6410 OS=Homo sapiens PE=4 SV=1	KV206_HUMAN	15 kDa	3	18%
680	Ig kappa chain V-III region B6 OS=Homo sapiens PE=1 SV=1	KV301_HUMAN	12 kDa	4	38%
681	Ig kappa chain V-III region NG9 (Fragment) OS=Homo sapiens PE=1 SV=1	KV303_HUMAN	11 kDa	3	32%
682	Ig kappa chain V-III region WOL OS=Homo sapiens PE=1 SV=1	KV305_HUMAN	12 kDa	6	52%
683	Ig kappa chain V-III region CLL OS=Homo sapiens PE=1 SV=2	KV308_HUMAN	14 kDa	2	19%
684	Ig kappa chain V-IV region Len OS=Homo sapiens PE=1 SV=2	KV402_HUMAN	13 kDa	3	30%
685	Kyphoscoliosis peptidase OS=Homo sapiens GN=KY PE=1 SV=2	KY_HUMAN	64 kDa	2	1.60%
686	Ig lambda-2 chain C regions OS=Homo sapiens GN=IGLC2 PE=1 SV=1	LAC2_HUMAN	11 kDa	10	93%
687	Ig lambda-7 chain C region OS=Homo sapiens GN=IGLC7 PE=1 SV=2	LAC7_HUMAN	11 kDa	3	79%
688	Alpha-lactalbumin OS=Homo sapiens GN=LALBA PE=1 SV=1	LALBA_HUMAN	16 kDa	16	80%
689	Laminin subunit beta-2 OS=Homo sapiens GN=LAMB2 PE=1 SV=2	LAMB2_HUMAN	196 kDa	2	1.30%
690	Laminin subunit gamma-1 OS=Homo sapiens GN=LAMC1 PE=1 SV=3	LAMC1_HUMAN	178 kDa	4	4.40%
691	Lysosome-associated membrane glycoprotein 1 OS=Homo sapiens GN=LAMP1 PE=1 SV=3	LAMP1_HUMAN	45 kDa	4	8.60%
692	Lysosome-associated membrane glycoprotein 2 OS=Homo sapiens GN=LAMP2 PE=1 SV=2	LAMP2_HUMAN	45 kDa	2	4.10%
693	LanC-like protein 1 OS=Homo sapiens GN=LANCL1 PE=1 SV=1	LANC1_HUMAN	45 kDa	4	12%
694	LIM and SH3 domain protein 1 OS=Homo sapiens GN=LASP1 PE=1 SV=2	LASP1_HUMAN	30 kDa	11	34%
695	L-lactate dehydrogenase A chain OS=Homo sapiens GN=LDHA PE=1 SV=2	LDHA_HUMAN	37 kDa	8	30%
696	L-lactate dehydrogenase B chain OS=Homo sapiens GN=LDHB PE=1 SV=2	LDHB_HUMAN	37 kDa	20	64%
697	Galectin-1 OS=Homo sapiens GN=LGALS1 PE=1 SV=2	LEG1_HUMAN	15 kDa	5	41%
698	Galectin-3 OS=Homo sapiens GN=LGALS3 PE=1 SV=5	LEG3_HUMAN	26 kDa	5	23%
699	Galectin-3-binding protein OS=Homo sapiens GN=LGALS3BP PE=1 SV=1	LG3BP_HUMAN	65 kDa	21	41%
700	Legumain OS=Homo sapiens GN=LGMN PE=1 SV=1	LGMN_HUMAN	49 kDa	11	30%
701	Lactoylglutathione lyase OS=Homo sapiens GN=GLO1 PE=1 SV=4	LGUL_HUMAN	21 kDa	5	28%
702	Phospholysine phosphohistidine inorganic pyrophosphate phosphatase OS=Homo sapiens GN=LHPP PE=1 SV=2	LHPP_HUMAN	29 kDa	2	11%
703	Lysosomal acid lipase/cholesteryl ester hydrolase OS=Homo sapiens GN=LIPA PE=1 SV=2	LICH_HUMAN	45 kDa	2	6.80%
704	Lipoprotein lipase OS=Homo sapiens GN=LPL PE=1 SV=1	LIPL_HUMAN	53 kDa	16	37%
705	Platelet-activating factor acetylhydrolase IB subunit alpha OS=Homo sapiens GN=PAFAH1B1 PE=1 SV=2	LIS1_HUMAN	47 kDa	7	21%
706	Leukotriene A-4 hydrolase OS=Homo sapiens GN=LTA4H PE=1 SV=2	LKHA4_HUMAN	69 kDa	19	35%
707	Protein ERGIC-53 OS=Homo sapiens GN=LMAN1 PE=1 SV=2	LMAN1_HUMAN	58 kDa	6	17%
708	Vesicular integral-membrane protein VIP36 OS=Homo sapiens GN=LMAN2 PE=1 SV=1	LMAN2_HUMAN	40 kDa	2	5.30%
709	Prelamin-A/C OS=Homo sapiens GN=LMNA PE=1 SV=1	LMNA_HUMAN	74 kDa	2	3.00%
710	Leucine-rich repeat-containing protein 26 OS=Homo sapiens GN=LRRC26 PE=1 SV=2	LRC26_HUMAN	35 kDa	4	15%
711	Leucine-rich repeat-containing protein 47 OS=Homo sapiens GN=LRRC47 PE=1 SV=1	LRC47_HUMAN	63 kDa	2	4.60%

#	Proteins	Uniprot ID	Molecular Weight	Number of unique peptides	Percent of coverage
712	Leucine-rich repeat flightless-interacting protein 1 OS=Homo sapiens GN=LRRFIP1 PE=1 SV=2	LRRF1_HUMAN	89 kDa	4	5.10%
713	Lipolysis-stimulated lipoprotein receptor OS=Homo sapiens GN=LSR PE=1 SV=4	LSR_HUMAN	71 kDa	11	20%
714	Latent-transforming growth factor beta-binding protein 1 OS=Homo sapiens GN=LTBP1 PE=1 SV=4	LTBP1_HUMAN	187 kDa	8	6.00%
715	Latent-transforming growth factor beta-binding protein 4 OS=Homo sapiens GN=LTBP4 PE=1 SV=2	LTBP4_HUMAN	173 kDa	3	3.00%
716	Lumican OS=Homo sapiens GN=LUM PE=1 SV=2	LUM_HUMAN	38 kDa	9	34%
717	Ig lambda chain V-I region HA OS=Homo sapiens PE=1 SV=1	LV102_HUMAN	12 kDa	2	23%
718	Ig lambda chain V-I region NEW OS=Homo sapiens PE=1 SV=1	LV103_HUMAN	11 kDa	2	26%
719	Ig lambda chain V-I region NIG-64 OS=Homo sapiens PE=1 SV=1	LV104_HUMAN (+1)	11 kDa	2	19%
720	Ig lambda chain V-I region NEWM OS=Homo sapiens PE=1 SV=1	LV105_HUMAN	11 kDa	3	22%
721	Ig lambda chain V-II region TRO OS=Homo sapiens PE=1 SV=1	LV204_HUMAN	12 kDa	3	28%
722	Ig lambda chain V-III region SH OS=Homo sapiens PE=1 SV=1	LV301_HUMAN	11 kDa	3	34%
723	Ig lambda chain V-III region LOI OS=Homo sapiens PE=1 SV=1	LV302_HUMAN	12 kDa	4	46%
724	Ig lambda chain V-VI region SUT OS=Homo sapiens PE=1 SV=1	LV603_HUMAN	12 kDa	4	37%
725	Lymphocyte antigen 96 OS=Homo sapiens GN=LY96 PE=1 SV=2	LY96_HUMAN	19 kDa	3	22%
726	Lysosomal alpha-glucosidase OS=Homo sapiens GN=GAA PE=1 SV=4	LYAG_HUMAN	105 kDa	4	7.00%
727	Tyrosine-protein kinase Lyn OS=Homo sapiens GN=LYN PE=1 SV=3	LYN_HUMAN	59 kDa	3	5.10%
728	Acyl-protein thioesterase 1 OS=Homo sapiens GN=LYPLA1 PE=1 SV=1	LYPA1_HUMAN	25 kDa	6	34%
729	Acyl-protein thioesterase 2 OS=Homo sapiens GN=LYPLA2 PE=1 SV=1	LYPA2_HUMAN	25 kDa	4	19%
730	Ly6/PLAUR domain-containing protein 3 OS=Homo sapiens GN=LYPD3 PE=1 SV=2	LYPD3_HUMAN	36 kDa	6	23%
731	Lysozyme C OS=Homo sapiens GN=LYZ PE=1 SV=1	LYSC_HUMAN	17 kDa	14	71%
732	Protein LZIC OS=Homo sapiens GN=LZIC PE=1 SV=1	LZIC_HUMAN	21 kDa	2	8.90%
733	Mannosyl-oligosaccharide 1,2-alpha-mannosidase IA OS=Homo sapiens GN=MAN1A1 PE=1 SV=3	MA1A1_HUMAN	73 kDa	13	21%
734	Alpha-mannosidase 2 OS=Homo sapiens GN=MAN2A1 PE=1 SV=2	MA2A1_HUMAN	131 kDa	7	6.60%
735	Lysosomal alpha-mannosidase OS=Homo sapiens GN=MAN2B1 PE=1 SV=3	MA2B1_HUMAN	114 kDa	10	11%
736	Epididymis-specific alpha-mannosidase OS=Homo sapiens GN=MAN2B2 PE=1 SV=4	MA2B2_HUMAN	114 kDa	3	4.10%
737	Maleylacetoacetate isomerase OS=Homo sapiens GN=GSTZ1 PE=1 SV=3	MAAI_HUMAN	24 kDa	2	11%
738	Microtubule-actin cross-linking factor 1, isoforms 1/2/3/5 OS=Homo sapiens GN=MACF1 PE=1 SV=4	MACF1_HUMAN	838 kDa	2	0.26%
739	Beta-mannosidase OS=Homo sapiens GN=MANBA PE=1 SV=3	MANBA_HUMAN	101 kDa	16	22%
740	NADP-dependent malic enzyme OS=Homo sapiens GN=ME1 PE=1 SV=1	MAOX_HUMAN	64 kDa	8	17%
741	Myristoylated alanine-rich C-kinase substrate OS=Homo sapiens GN=MARCKS PE=1 SV=4	MARCS_HUMAN	32 kDa	9	39%
742	Matrilin-2 OS=Homo sapiens GN=MATN2 PE=1 SV=4	MATN2_HUMAN	107 kDa	14	17%
743	Matrilin-3 OS=Homo sapiens GN=MATN3 PE=1 SV=2	MATN3_HUMAN	53 kDa	17	50%
744	Myelin basic protein OS=Homo sapiens GN=MBP PE=1 SV=3	MBP_HUMAN	33 kDa	2	15%
745	Malignant T cell-amplified sequence 1 OS=Homo sapiens GN=MCTS1 PE=1 SV=1	MCTS1_HUMAN	21 kDa	2	20%
746	Malate dehydrogenase, cytoplasmic OS=Homo sapiens GN=MDH1 PE=1 SV=4	MDHC_HUMAN	36 kDa	16	45%
747	Malate dehydrogenase, mitochondrial OS=Homo sapiens GN=MDH2 PE=1 SV=3	MDHM_HUMAN	36 kDa	3	15%
748	LDLR chaperone MESD OS=Homo sapiens GN=MESDC2 PE=1 SV=2	MESD_HUMAN	26 kDa	3	11%
749	Methyltransferase-like protein 7A OS=Homo sapiens GN=METTL7A PE=1 SV=1	MET7A_HUMAN	28 kDa	2	11%
750	S-adenosylmethionine synthase isoform type-2 OS=Homo sapiens GN=MAT2A PE=1 SV=1	METK2_HUMAN	44 kDa	2	6.10%
751	Lactadherin OS=Homo sapiens GN=MFGE8 PE=1 SV=2	MFGM_HUMAN	43 kDa	27	79%

#	Proteins	Uniprot ID	Molecular Weight	Number of unique peptides	Percent of coverage
752	Alpha-1,3-mannosyl-glycoprotein 2-beta-N-acetylglucosaminyltransferase OS=Homo sapiens GN=MGAT1 PE=2 SV=2	MGAT1_HUMAN	51 kDa	2	5.60%
753	Melanoma inhibitory activity protein 3 OS=Homo sapiens GN=MIA3 PE=1 SV=1	MIA3_HUMAN	214 kDa	3	2.30%
754	Multiple inositol polyphosphate phosphatase 1 OS=Homo sapiens GN=MINPP1 PE=1 SV=1	MINP1_HUMAN	55 kDa	5	11%
755	Midkine OS=Homo sapiens GN=MDK PE=1 SV=1	MK_HUMAN	16 kDa	3	25%
756	Mitogen-activated protein kinase 1 OS=Homo sapiens GN=MAPK1 PE=1 SV=3	MK01_HUMAN	41 kDa	3	9.40%
757	Mitogen-activated protein kinase 13 OS=Homo sapiens GN=MAPK13 PE=1 SV=1	MK13_HUMAN	42 kDa	4	12%
758	Myosin regulatory light chain 12A OS=Homo sapiens GN=MYL12A PE=1 SV=2	ML12A_HUMAN (+1)	20 kDa	6	40%
759	Matrilysin OS=Homo sapiens GN=MMP7 PE=1 SV=1	MMP7_HUMAN	30 kDa	2	8.20%
760	Moesin OS=Homo sapiens GN=MSN PE=1 SV=3	MOES_HUMAN	68 kDa	31	52%
761	Dual specificity mitogen-activated protein kinase kinase 1 OS=Homo sapiens GN=MAP2K1 PE=1 SV=2	MP2K1_HUMAN	43 kDa	5	13%
762	Mannose-6-phosphate isomerase OS=Homo sapiens GN=MPI PE=1 SV=2	MPI_HUMAN	47 kDa	3	7.10%
763	Cation-independent mannose-6-phosphate receptor OS=Homo sapiens GN=IGF2R PE=1 SV=3	MPRI_HUMAN	274 kDa	11	5.70%
764	Macrophage mannose receptor 1 OS=Homo sapiens GN=MRC1 PE=1 SV=1	MRC1_HUMAN	166 kDa	85	56%
765	MARCKS-related protein OS=Homo sapiens GN=MARCKSL1 PE=1 SV=2	MRP_HUMAN	20 kDa	5	60%
766	Multidrug resistance-associated protein 7 OS=Homo sapiens GN=ABCC10 PE=1 SV=1	MRP7_HUMAN	162 kDa	2	1.90%
767	Mitochondrial peptide methionine sulfoxide reductase OS=Homo sapiens GN=MSRA PE=1 SV=1	MSRA_HUMAN	26 kDa	4	20%
768	S-methyl-5'-thioadenosine phosphorylase OS=Homo sapiens GN=MTAP PE=1 SV=2	MTAP_HUMAN	31 kDa	8	37%
769	Myotrophin OS=Homo sapiens GN=MTPN PE=1 SV=2	MTPN_HUMAN	13 kDa	2	21%
770	Mucin-1 OS=Homo sapiens GN=MUC1 PE=1 SV=3	MUC1_HUMAN	122 kDa	9	8.50%
771	Cell surface glycoprotein MUC18 OS=Homo sapiens GN=MCAM PE=1 SV=2	MUC18_HUMAN	72 kDa	2	4.00%
772	Mucin-20 OS=Homo sapiens GN=MUC20 PE=1 SV=3	MUC20_HUMAN	72 kDa	4	7.90%
773	Mucin-4 OS=Homo sapiens GN=MUC4 PE=1 SV=4	MUC4_HUMAN	232 kDa	23	16%
774	Ig mu heavy chain disease protein OS=Homo sapiens PE=1 SV=1	MUCB_HUMAN	43 kDa	2	50%
775	Mucin-like protein 1 OS=Homo sapiens GN=MUCL1 PE=1 SV=1	MUCL1_HUMAN	9 kDa	2	18%
776	Diphosphomevalonate decarboxylase OS=Homo sapiens GN=MVD PE=1 SV=1	MVD1_HUMAN	43 kDa	6	19%
777	Myosin-9 OS=Homo sapiens GN=MYH9 PE=1 SV=4	MYH9_HUMAN	227 kDa	31	19%
778	Myosin light polypeptide 6 OS=Homo sapiens GN=MYL6 PE=1 SV=2	MYL6_HUMAN	17 kDa	9	66%
779	Myosin-Ic OS=Homo sapiens GN=MYO1C PE=1 SV=4	MYO1C_HUMAN	122 kDa	5	5.10%
780	Myoferlin OS=Homo sapiens GN=MYOF PE=1 SV=1	MYOF_HUMAN	235 kDa	5	2.60%
781	N-alpha-acetyltransferase 15, NatA auxiliary subunit OS=Homo sapiens GN=NAA15 PE=1 SV=1	NAA15_HUMAN	101 kDa	2	2.90%
782	Nascent polypeptide-associated complex subunit alpha OS=Homo sapiens GN=NACA PE=1 SV=1	NACA_HUMAN	23 kDa	5	29%
783	Nicotinate-nucleotide pyrophosphorylase [carboxylating] OS=Homo sapiens GN=QPRT PE=1 SV=3	NADC_HUMAN	31 kDa	3	11%
784	N-acetyl-D-glucosamine kinase OS=Homo sapiens GN=NAGK PE=1 SV=4	NAGK_HUMAN	37 kDa	6	24%
785	Nicotinamide phosphoribosyltransferase OS=Homo sapiens GN=NAMPT PE=1 SV=1	NAMPT_HUMAN	56 kDa	26	67%
786	N-acetyltransferase 10 OS=Homo sapiens GN=NAT10 PE=1 SV=2	NAT10_HUMAN	116 kDa	2	2.40%
787	Non-specific cytotoxic cell receptor protein 1 homolog OS=Homo sapiens GN=NCCRP1 PE=1 SV=1	NCRP1_HUMAN	31 kDa	2	18%
788	Nucleoside diphosphate kinase A OS=Homo sapiens GN=NME1 PE=1 SV=1	NDKA_HUMAN	17 kDa	6	70%
789	Nucleoside diphosphate kinase B OS=Homo sapiens GN=NME2 PE=1 SV=1	NDKB_HUMAN	17 kDa	10	65%
790	Protein NDRG1 OS=Homo sapiens GN=NDRG1 PE=1 SV=1	NDRG1_HUMAN	43 kDa	6	24%

#	Proteins	Uniprot ID	Molecular Weight	Number of unique peptides	Percent of coverage
791	Protein NDRG2 OS=Homo sapiens GN=NDRG2 PE=1 SV=2	NDRG2_HUMAN	41 kDa	6	20%
792	Protein NDRG3 OS=Homo sapiens GN=NDRG3 PE=1 SV=2	NDRG3_HUMAN	41 kDa	2	8.50%
793	Bifunctional heparan sulfate N-deacetylase/N-sulfotransferase 1 OS=Homo sapiens GN=NDST1 PE=1 SV=1	NDST1_HUMAN	101 kDa	5	6.50%
794	Nebulin OS=Homo sapiens GN=NEB PE=1 SV=4	NEBU_HUMAN	773 kDa	2	0.39%
795	Neudesin OS=Homo sapiens GN=NENF PE=1 SV=1	NENF_HUMAN	19 kDa	2	11%
796	Neogenin OS=Homo sapiens GN=NEO1 PE=1 SV=2	NEO1_HUMAN	160 kDa	3	2.70%
797	Sialidase-1 OS=Homo sapiens GN=NEU1 PE=1 SV=1	NEUR1_HUMAN	45 kDa	12	33%
798	Neuroserpin OS=Homo sapiens GN=SERPINI1 PE=1 SV=1	NEUS_HUMAN	46 kDa	6	19%
799	Neutrophil gelatinase-associated lipocalin OS=Homo sapiens GN=LCN2 PE=1 SV=2	NGAL_HUMAN	23 kDa	2	14%
800	NHL repeat-containing protein 2 OS=Homo sapiens GN=NHLRC2 PE=1 SV=1	NHLC2_HUMAN	79 kDa	5	8.00%
801	Na(+)/H(+) exchange regulatory cofactor NHE-RF1 OS=Homo sapiens GN=SLC9A3R1 PE=1 SV=4	NHRF1_HUMAN	39 kDa	19	60%
802	Na(+)/H(+) exchange regulatory cofactor NHE-RF2 OS=Homo sapiens GN=SLC9A3R2 PE=1 SV=2	NHRF2_HUMAN	37 kDa	4	15%
803	Niban-like protein 1 OS=Homo sapiens GN=FAM129B PE=1 SV=2	NIBL1_HUMAN	83 kDa	13	22%
804	Nidogen-1 OS=Homo sapiens GN=NID1 PE=1 SV=3	NID1_HUMAN	136 kDa	8	7.40%
805	Omega-amidase NIT2 OS=Homo sapiens GN=NIT2 PE=1 SV=1	NIT2_HUMAN	31 kDa	5	23%
806	Non-specific lipid-transfer protein OS=Homo sapiens GN=SCP2 PE=1 SV=2	NLTP_HUMAN	59 kDa	10	17%
807	Glycylpeptide N-tetradecanoyltransferase 1 OS=Homo sapiens GN=NMT1 PE=1 SV=2	NMT1_HUMAN	57 kDa	4	12%
808	Nucleolar protein 3 OS=Homo sapiens GN=NOL3 PE=1 SV=1	NOL3_HUMAN	24 kDa	2	8.20%
809	Nodal modulator 1 OS=Homo sapiens GN=NOMO1 PE=1 SV=5	NOMO1_HUMAN (+2)	134 kDa	5	4.80%
810	Nucleosome assembly protein 1-like 1 OS=Homo sapiens GN=NAP1L1 PE=1 SV=1	NP1L1_HUMAN	45 kDa	5	22%
811	Nucleosome assembly protein 1-like 4 OS=Homo sapiens GN=NAP1L4 PE=1 SV=1	NP1L4_HUMAN	43 kDa	5	15%
812	Epididymal secretory protein E1 OS=Homo sapiens GN=NPC2 PE=1 SV=1	NPC2_HUMAN	17 kDa	7	62%
813	Neural proliferation differentiation and control protein 1 OS=Homo sapiens GN=NPDC1 PE=1 SV=2	NPDC1_HUMAN	35 kDa	2	12%
814	Nephronectin OS=Homo sapiens GN=NPNT PE=2 SV=3	NPNT_HUMAN	62 kDa	8	14%
815	Sodium-dependent phosphate transport protein 2B OS=Homo sapiens GN=SLC34A2 PE=1 SV=3	NPT2B_HUMAN	76 kDa	6	8.10%
816	Ribosyldihydronicotinamide dehydrogenase [quinone] OS=Homo sapiens GN=NQO2 PE=1 SV=5	NQO2_HUMAN	26 kDa	4	17%
817	Neuropilin-1 OS=Homo sapiens GN=NRP1 PE=1 SV=3	NRP1_HUMAN	103 kDa	10	15%
818	Vesicle-fusing ATPase OS=Homo sapiens GN=NSF PE=1 SV=3	NSF_HUMAN	83 kDa	2	3.10%
819	NSFL1 cofactor p47 OS=Homo sapiens GN=NSFL1C PE=1 SV=2	NSF1C_HUMAN	41 kDa	7	26%
820	5'-nucleotidase domain-containing protein 1 OS=Homo sapiens GN=NT5DC1 PE=1 SV=1	NT5D1_HUMAN	52 kDa	2	6.20%
821	Nuclear transport factor 2 OS=Homo sapiens GN=NUTF2 PE=1 SV=1	NTF2_HUMAN	14 kDa	3	24%
822	Cytosolic Fe-S cluster assembly factor NUBP1 OS=Homo sapiens GN=NUBP1 PE=1 SV=2	NUBP1_HUMAN	35 kDa	2	8.10%
823	Nucleobindin-1 OS=Homo sapiens GN=NUCB1 PE=1 SV=4	NUCB1_HUMAN	54 kDa	35	70%
824	Nucleobindin-2 OS=Homo sapiens GN=NUCB2 PE=1 SV=2	NUCB2_HUMAN	50 kDa	27	60%
825	Nuclear migration protein nudC OS=Homo sapiens GN=NUDC PE=1 SV=1	NUDC_HUMAN	38 kDa	3	11%
826	ADP-sugar pyrophosphatase OS=Homo sapiens GN=NUDT5 PE=1 SV=1	NUDT5_HUMAN	24 kDa	4	22%
827	Protein NOXP20 OS=Homo sapiens GN=FAM114A1 PE=1 SV=2	NXP20_HUMAN	61 kDa	3	5.90%
828	Occludin OS=Homo sapiens GN=OCLN PE=1 SV=1	OCLN_HUMAN	59 kDa	4	9.20%
829	Obg-like ATPase 1 OS=Homo sapiens GN=OLA1 PE=1 SV=2	OLA1_HUMAN	45 kDa	3	8.80%
830	Olfactomedin-4 OS=Homo sapiens GN=OLFM4 PE=1 SV=1	OLFM4_HUMAN	57 kDa	11	22%

#	Proteins	Uniprot ID	Molecular Weight	Number of unique peptides	Percent of coverage
831	Protein OS-9 OS=Homo sapiens GN=OS9 PE=1 SV=1	OS9_HUMAN	76 kDa	14	25%
832	Oxysterol-binding protein 1 OS=Homo sapiens GN=OSBP PE=1 SV=1	OSBP1_HUMAN	89 kDa	5	6.40%
833	Oncostatin-M-specific receptor subunit beta OS=Homo sapiens GN=OSMR PE=1 SV=1	OSMR_HUMAN	111 kDa	7	7.50%
834	Osteoclast-stimulating factor 1 OS=Homo sapiens GN=OSTF1 PE=1 SV=2	OSTF1_HUMAN	24 kDa	2	14%
835	Osteopontin OS=Homo sapiens GN=SPP1 PE=1 SV=1	OSTP_HUMAN	35 kDa	16	59%
836	Ubiquitin thioesterase OTUB1 OS=Homo sapiens GN=OTUB1 PE=1 SV=2	OTUB1_HUMAN	31 kDa	4	19%
837	Serine/threonine-protein kinase OSR1 OS=Homo sapiens GN=OXSR1 PE=1 SV=1	OXSR1_HUMAN	58 kDa	3	7.20%
838	Phosphoinositide-3-kinase-interacting protein 1 OS=Homo sapiens GN=PIK3IP1 PE=1 SV=2	P3IP1_HUMAN	28 kDa	5	21%
839	Platelet-activating factor acetylhydrolase IB subunit beta OS=Homo sapiens GN=PAFAH1B2 PE=1 SV=1	PA1B2_HUMAN	26 kDa	3	22%
840	Platelet-activating factor acetylhydrolase IB subunit gamma OS=Homo sapiens GN=PAFAH1B3 PE=1 SV=1	PA1B3_HUMAN	26 kDa	2	7.80%
841	Group 3 secretory phospholipase A2 OS=Homo sapiens GN=PLA2G3 PE=1 SV=2	PA2G3_HUMAN	57 kDa	3	7.70%
842	Proliferation-associated protein 2G4 OS=Homo sapiens GN=PA2G4 PE=1 SV=3	PA2G4_HUMAN	44 kDa	10	31%
843	Polyadenylate-binding protein 1 OS=Homo sapiens GN=PABPC1 PE=1 SV=2	PABP1_HUMAN	71 kDa	19	34%
844	Polyadenylate-binding protein 4 OS=Homo sapiens GN=PABPC4 PE=1 SV=1	PABP4_HUMAN	71 kDa	2	15%
845	Protein kinase C and casein kinase substrate in neurons protein 2 OS=Homo sapiens GN=PACSIN2 PE=1 SV=2	PACN2_HUMAN	56 kDa	3	5.80%
846	Protein kinase C and casein kinase substrate in neurons protein 3 OS=Homo sapiens GN=PACSIN3 PE=1 SV=2	PACN3_HUMAN	48 kDa	2	5.40%
847	Phosphofurin acidic cluster sorting protein 1 OS=Homo sapiens GN=PACS1 PE=1 SV=2	PACS1_HUMAN	105 kDa	2	3.10%
848	Protein-arginine deiminase type-2 OS=Homo sapiens GN=PADI2 PE=1 SV=2	PADI2_HUMAN	76 kDa	2	3.90%
849	Group XV phospholipase A2 OS=Homo sapiens GN=PLA2G15 PE=1 SV=2	PAG15_HUMAN	47 kDa	2	5.60%
850	Polyadenylate-binding protein-interacting protein 1 OS=Homo sapiens GN=PAIP1 PE=1 SV=1	PAIP1_HUMAN	54 kDa	7	14%
851	Plasminogen activator inhibitor 1 RNA-binding protein OS=Homo sapiens GN=SERBP1 PE=1 SV=2	PAIRB_HUMAN	45 kDa	5	15%
852	Serine/threonine-protein kinase PAK 2 OS=Homo sapiens GN=PAK2 PE=1 SV=3	PAK2_HUMAN	58 kDa	5	11%
853	Serine/threonine-protein kinase PAK 4 OS=Homo sapiens GN=PAK4 PE=1 SV=1	PAK4_HUMAN	64 kDa	2	4.20%
854	Paralemmin-3 OS=Homo sapiens GN=PALM3 PE=1 SV=2	PALM3_HUMAN	72 kDa	2	3.70%
855	Protein DJ-1 OS=Homo sapiens GN=PARK7 PE=1 SV=2	PARK7_HUMAN	20 kDa	16	83%
856	Purkinje cell protein 4-like protein 1 OS=Homo sapiens GN=PCP4L1 PE=2 SV=3	PC4L1_HUMAN	7 kDa	2	32%
857	Poly(rC)-binding protein 1 OS=Homo sapiens GN=PCBP1 PE=1 SV=2	PCBP1_HUMAN	37 kDa	11	42%
858	Poly(rC)-binding protein 2 OS=Homo sapiens GN=PCBP2 PE=1 SV=1	PCBP2_HUMAN	39 kDa	3	23%
859	Procollagen C-endopeptidase enhancer 1 OS=Homo sapiens GN=PCOLCE PE=1 SV=2	PCOC1_HUMAN	48 kDa	2	5.80%
860	Lysosomal Pro-X carboxypeptidase OS=Homo sapiens GN=PRCP PE=1 SV=1	PCP_HUMAN	56 kDa	2	6.20%
861	Proprotein convertase subtilisin/kexin type 6 OS=Homo sapiens GN=PCSK6 PE=1 SV=1	PCSK6_HUMAN	106 kDa	2	3.00%
862	Ethanolamine-phosphate cytidylyltransferase OS=Homo sapiens GN=PCYT2 PE=1 SV=1	PCY2_HUMAN	44 kDa	6	19%
863	Programmed cell death 6-interacting protein OS=Homo sapiens GN=PDCD6IP PE=1 SV=1	PDC6I_HUMAN	96 kDa	24	27%
864	Programmed cell death protein 4 OS=Homo sapiens GN=PDCD4 PE=1 SV=2	PDCD4_HUMAN	52 kDa	6	15%
865	Programmed cell death protein 5 OS=Homo sapiens GN=PDCD5 PE=1 SV=3	PDCD5_HUMAN	14 kDa	4	38%
866	Protein disulfide-isomerase OS=Homo sapiens GN=P4HB PE=1 SV=3	PDIA1_HUMAN	57 kDa	34	66%
867	Protein disulfide-isomerase A3 OS=Homo sapiens GN=PDIA3 PE=1 SV=4	PDIA3_HUMAN	57 kDa	25	47%
868	Protein disulfide-isomerase A4 OS=Homo sapiens GN=PDIA4 PE=1 SV=2	PDIA4_HUMAN	73 kDa	13	21%
869	Protein disulfide-isomerase A6 OS=Homo sapiens GN=PDIA6 PE=1 SV=1	PDIA6_HUMAN	48 kDa	4	12%

#	Proteins	Uniprot ID	Molecular Weight	Number of unique peptides	Percent of coverage
870	PDZ and LIM domain protein 5 OS=Homo sapiens GN=PDLIM5 PE=1 SV=5	PDLI5_HUMAN	64 kDa	4	11%
871	Pyridoxal kinase OS=Homo sapiens GN=PDXK PE=1 SV=1	PDXK_HUMAN	35 kDa	10	31%
872	Podocalyxin-like protein 2 OS=Homo sapiens GN=PODXL2 PE=1 SV=1	PDXL2_HUMAN	65 kDa	2	3.60%
873	PDZK1-interacting protein 1 OS=Homo sapiens GN=PDZK1IP1 PE=1 SV=1	PDZ1I_HUMAN	12 kDa	2	26%
874	Astrocytic phosphoprotein PEA-15 OS=Homo sapiens GN=PEA15 PE=1 SV=2	PEA15_HUMAN	15 kDa	4	45%
875	Phosphatidylethanolamine-binding protein 1 OS=Homo sapiens GN=PEBP1 PE=1 SV=3	PEBP1_HUMAN	21 kDa	12	83%
876	Phosphatidylethanolamine-binding protein 4 OS=Homo sapiens GN=PEBP4 PE=1 SV=3	PEBP4_HUMAN	26 kDa	2	7.90%
877	Pigment epithelium-derived factor OS=Homo sapiens GN=SERPINF1 PE=1 SV=4	PEDF_HUMAN	46 kDa	16	46%
878	Xaa-Pro dipeptidase OS=Homo sapiens GN=PEPD PE=1 SV=3	PEPD_HUMAN	55 kDa	2	3.70%
879	Periplakin OS=Homo sapiens GN=PPL PE=1 SV=4	PEPL_HUMAN	205 kDa	2	1.30%
880	Lactoperoxidase OS=Homo sapiens GN=LPO PE=1 SV=2	PERL_HUMAN	80 kDa	31	50%
881	Prefoldin subunit 2 OS=Homo sapiens GN=PFDN2 PE=1 SV=1	PFD2_HUMAN	17 kDa	3	23%
882	Prefoldin subunit 3 OS=Homo sapiens GN=VBP1 PE=1 SV=3	PFD3_HUMAN	23 kDa	2	13%
883	Prefoldin subunit 5 OS=Homo sapiens GN=PFDN5 PE=1 SV=2	PFD5_HUMAN	17 kDa	3	33%
884	Prefoldin subunit 6 OS=Homo sapiens GN=PFDN6 PE=1 SV=1	PFD6_HUMAN	15 kDa	3	14%
885	Phosphoglycerate mutase 1 OS=Homo sapiens GN=PGAM1 PE=1 SV=2	PGAM1_HUMAN	29 kDa	12	60%
886	Basement membrane-specific heparan sulfate proteoglycan core protein OS=Homo sapiens GN=HSPG2 PE=1 SV=4	PGBM_HUMAN	469 kDa	3	0.91%
887	Plasma glutamate carboxypeptidase OS=Homo sapiens GN=PGCP PE=1 SV=1	PGCP_HUMAN	52 kDa	9	25%
888	Phosphoglycerate kinase 1 OS=Homo sapiens GN=PGK1 PE=1 SV=3	PGK1_HUMAN	45 kDa	28	73%
889	Phosphoglucomutase-1 OS=Homo sapiens GN=PGM1 PE=1 SV=3	PGM1_HUMAN	61 kDa	33	70%
890	Phosphoglucomutase-2 OS=Homo sapiens GN=PGM2 PE=1 SV=4	PGM2_HUMAN	68 kDa	13	25%
891	Membrane-associated progesterone receptor component 1 OS=Homo sapiens GN=PGRMC1 PE=1 SV=3	PGRC1_HUMAN	22 kDa	3	17%
892	N-acetylmuramoyl-L-alanine amidase OS=Homo sapiens GN=PGLYRP2 PE=1 SV=1	PGRP2_HUMAN	62 kDa	5	13%
893	14 kDa phosphohistidine phosphatase OS=Homo sapiens GN=PHPT1 PE=1 SV=1	PHP14_HUMAN	14 kDa	3	26%
894	Pterin-4-alpha-carbinolamine dehydratase OS=Homo sapiens GN=PCBD1 PE=1 SV=2	PHS_HUMAN	12 kDa	7	64%
895	Phosphatidylinositol-5-phosphate 4-kinase type-2 gamma OS=Homo sapiens GN=PIP4K2C PE=1 SV=3	PI42C_HUMAN	47 kDa	4	12%
896	Phosphatidylinositol 4-kinase alpha OS=Homo sapiens GN=PI4KA PE=1 SV=3	PI4KA_HUMAN	231 kDa	5	3.90%
897	Polymeric immunoglobulin receptor OS=Homo sapiens GN=PIGR PE=1 SV=4	PIGR_HUMAN	83 kDa	55	71%
898	Protein-L-isoaspartate(D-aspartate) O-methyltransferase OS=Homo sapiens GN=PCMT1 PE=1 SV=4	PIMT_HUMAN	25 kDa	7	35%
899	Prolactin-inducible protein OS=Homo sapiens GN=PIP PE=1 SV=1	PIP_HUMAN	17 kDa	7	57%
900	Phosphatidylinositol transfer protein alpha isoform OS=Homo sapiens GN=PITPNA PE=1 SV=2	PIPNA_HUMAN	32 kDa	6	25%
901	Phosphatidylinositol transfer protein beta isoform OS=Homo sapiens GN=PITPNB PE=1 SV=2	PIPNB_HUMAN	32 kDa	3	13%
902	Pirin OS=Homo sapiens GN=PIR PE=1 SV=1	PIR_HUMAN	32 kDa	4	16%
903	PITH domain-containing protein 1 OS=Homo sapiens GN=PITHD1 PE=1 SV=1	PITH1_HUMAN	24 kDa	2	13%
904	Phospholipase A-2-activating protein OS=Homo sapiens GN=PLAA PE=1 SV=2	PLAP_HUMAN	87 kDa	2	4.30%
905	Putative phospholipase B-like 2 OS=Homo sapiens GN=PLBD2 PE=1 SV=2	PLBL2_HUMAN	65 kDa	6	11%
906	1-phosphatidylinositol-4,5-bisphosphate phosphodiesterase gamma-2 OS=Homo sapiens GN=PLCG2 PE=1 SV=4	PLCG2_HUMAN	148 kDa	2	1.20%
907	Plectin OS=Homo sapiens GN=PLEC PE=1 SV=3	PLEC_HUMAN	532 kDa	3	0.90%
908	Perilipin-2 OS=Homo sapiens GN=PLIN2 PE=1 SV=2	PLIN2_HUMAN	48 kDa	29	75%

#	Proteins	Uniprot ID	Molecular Weight	Number of unique peptides	Percent of coverage
909	Perilipin-3 OS=Homo sapiens GN=PLIN3 PE=1 SV=3	PLIN3_HUMAN	47 kDa	27	86%
910	Plasminogen OS=Homo sapiens GN=PLG PE=1 SV=2	PLMN_HUMAN	91 kDa	23	33%
911	Procollagen-lysine,2-oxoglutarate 5-dioxygenase 1 OS=Homo sapiens GN=PLOD1 PE=1 SV=2	PLOD1_HUMAN	84 kDa	16	27%
912	Procollagen-lysine,2-oxoglutarate 5-dioxygenase 2 OS=Homo sapiens GN=PLOD2 PE=1 SV=2	PLOD2_HUMAN	85 kDa	5	7.30%
913	Procollagen-lysine,2-oxoglutarate 5-dioxygenase 3 OS=Homo sapiens GN=PLOD3 PE=1 SV=1	PLOD3_HUMAN	85 kDa	5	9.30%
914	Plastin-1 OS=Homo sapiens GN=PLS1 PE=1 SV=2	PLSI_HUMAN	70 kDa	4	10%
915	Plastin-2 OS=Homo sapiens GN=LCP1 PE=1 SV=6	PLSL_HUMAN	70 kDa	21	42%
916	Phospholipid transfer protein OS=Homo sapiens GN=PLTP PE=1 SV=1	PLTP_HUMAN	55 kDa	11	25%
917	Phosphomannomutase 2 OS=Homo sapiens GN=PMM2 PE=1 SV=1	PMM2_HUMAN	28 kDa	6	26%
918	Nicotinate phosphoribosyltransferase OS=Homo sapiens GN=NAPRT1 PE=1 SV=2	PNCB_HUMAN	58 kDa	9	23%
919	Purine nucleoside phosphorylase OS=Homo sapiens GN=PNP PE=1 SV=2	PNPH_HUMAN	32 kDa	18	74%
920	Podocalyxin OS=Homo sapiens GN=PODXL PE=1 SV=2	PODXL_HUMAN	59 kDa	8	14%
921	Serum paraoxonase/arylesterase 1 OS=Homo sapiens GN=PON1 PE=1 SV=3	PON1_HUMAN	40 kDa	9	29%
922	Periostin OS=Homo sapiens GN=POSTN PE=1 SV=2	POSTN_HUMAN	93 kDa	4	7.30%
923	Serine/threonine-protein phosphatase PP1-beta catalytic subunit OS=Homo sapiens GN=PPP1CB PE=1 SV=3	PP1B_HUMAN	37 kDa	7	32%
924	Protein phosphatase 1 regulatory subunit 7 OS=Homo sapiens GN=PPP1R7 PE=1 SV=1	PP1R7_HUMAN	42 kDa	9	29%
925	Serine/threonine-protein phosphatase 2A catalytic subunit alpha isoform OS=Homo sapiens GN=PPP2CA PE=1 SV=1	PP2AA_HUMAN	36 kDa	2	6.10%
926	Serine/threonine-protein phosphatase 2B catalytic subunit beta isoform OS=Homo sapiens GN=PPP3CB PE=1 SV=2	PP2BB_HUMAN	59 kDa	2	3.40%
927	Low molecular weight phosphotyrosine protein phosphatase OS=Homo sapiens GN=ACP1 PE=1 SV=3	PPAC_HUMAN	18 kDa	3	22%
928	Prolyl endopeptidase OS=Homo sapiens GN=PREP PE=1 SV=2	PPCE_HUMAN	81 kDa	3	6.20%
929	Phosphopantothenate--cysteine ligase OS=Homo sapiens GN=PPCS PE=1 SV=2	PPCS_HUMAN	34 kDa	3	9.00%
930	Lysosomal protective protein OS=Homo sapiens GN=CTSA PE=1 SV=2	PPGB_HUMAN	54 kDa	4	11%
931	Peptidyl-prolyl cis-trans isomerase A OS=Homo sapiens GN=PPIA PE=1 SV=2	PPIA_HUMAN	18 kDa	15	82%
932	Peptidyl-prolyl cis-trans isomerase B OS=Homo sapiens GN=PPIB PE=1 SV=2	PPIB_HUMAN	24 kDa	14	55%
933	Peptidyl-prolyl cis-trans isomerase C OS=Homo sapiens GN=PPIC PE=1 SV=1	PPIC_HUMAN	23 kDa	5	24%
934	Protein phosphatase 1A OS=Homo sapiens GN=PPM1A PE=1 SV=1	PPM1A_HUMAN	42 kDa	4	11%
935	Serine/threonine-protein phosphatase 5 OS=Homo sapiens GN=PPP5C PE=1 SV=1	PPP5_HUMAN	57 kDa	3	6.80%
936	Palmitoyl-protein thioesterase 1 OS=Homo sapiens GN=PPT1 PE=1 SV=1	PPT1_HUMAN	34 kDa	4	14%
937	Peroxiredoxin-1 OS=Homo sapiens GN=PRDX1 PE=1 SV=1	PRDX1_HUMAN	22 kDa	13	61%
938	Peroxiredoxin-2 OS=Homo sapiens GN=PRDX2 PE=1 SV=5	PRDX2_HUMAN	22 kDa	9	48%
939	Peroxiredoxin-4 OS=Homo sapiens GN=PRDX4 PE=1 SV=1	PRDX4_HUMAN	31 kDa	5	31%
940	Peroxiredoxin-5, mitochondrial OS=Homo sapiens GN=PRDX5 PE=1 SV=4	PRDX5_HUMAN	22 kDa	8	48%
941	Peroxiredoxin-6 OS=Homo sapiens GN=PRDX6 PE=1 SV=3	PRDX6_HUMAN	25 kDa	17	76%
942	Major prion protein OS=Homo sapiens GN=PRNP PE=1 SV=1	PRIO_HUMAN	28 kDa	2	8.30%
943	Prolactin OS=Homo sapiens GN=PRL PE=1 SV=1	PRL_HUMAN	26 kDa	2	11%
944	Vitamin K-dependent protein C OS=Homo sapiens GN=PROC PE=1 SV=1	PROC_HUMAN	52 kDa	3	7.60%
945	Profilin-1 OS=Homo sapiens GN=PFN1 PE=1 SV=2	PROF1_HUMAN	15 kDa	12	91%
946	Prominin-1 OS=Homo sapiens GN=PROM1 PE=1 SV=1	PROM1_HUMAN	97 kDa	13	19%
947	Prominin-2 OS=Homo sapiens GN=PROM2 PE=1 SV=1	PROM2_HUMAN	92 kDa	11	18%
948	Vitamin K-dependent protein S OS=Homo sapiens GN=PROS1 PE=1 SV=1	PROS_HUMAN	75 kDa	3	3.40%

#	Proteins	Uniprot ID	Molecular Weight	Number of unique peptides	Percent of coverage
949	Proline synthase co-transcribed bacterial homolog protein OS=Homo sapiens GN=PROSC PE=1 SV=1	PROSC_HUMAN	30 kDa	4	17%
950	Ribose-phosphate pyrophosphokinase 2 OS=Homo sapiens GN=PRPS2 PE=1 SV=2	PRPS2_HUMAN	35 kDa	14	50%
951	Protein PRRC1 OS=Homo sapiens GN=PRRC1 PE=1 SV=1	PRRC1_HUMAN	47 kDa	4	9.70%
952	26S protease regulatory subunit 10B OS=Homo sapiens GN=PSMC6 PE=1 SV=1	PRS10_HUMAN	44 kDa	3	8.00%
953	Serine protease 23 OS=Homo sapiens GN=PRSS23 PE=1 SV=1	PRS23_HUMAN	43 kDa	2	6.30%
954	26S protease regulatory subunit 6A OS=Homo sapiens GN=PSMC3 PE=1 SV=3	PRS6A_HUMAN	49 kDa	8	21%
955	26S protease regulatory subunit 6B OS=Homo sapiens GN=PSMC4 PE=1 SV=2	PRS6B_HUMAN	47 kDa	6	14%
956	26S protease regulatory subunit 7 OS=Homo sapiens GN=PSMC2 PE=1 SV=3	PRS7_HUMAN	49 kDa	8	22%
957	26S protease regulatory subunit 8 OS=Homo sapiens GN=PSMC5 PE=1 SV=1	PRS8_HUMAN	46 kDa	8	26%
958	Prostasin OS=Homo sapiens GN=PRSS8 PE=1 SV=1	PRSS8_HUMAN	36 kDa	2	6.10%
959	Puromycin-sensitive aminopeptidase OS=Homo sapiens GN=NPEPPS PE=1 SV=2	PSA_HUMAN	103 kDa	15	19%
960	Proteasome subunit alpha type-1 OS=Homo sapiens GN=PSMA1 PE=1 SV=1	PSA1_HUMAN	30 kDa	12	46%
961	Proteasome subunit alpha type-2 OS=Homo sapiens GN=PSMA2 PE=1 SV=2	PSA2_HUMAN	26 kDa	9	50%
962	Proteasome subunit alpha type-3 OS=Homo sapiens GN=PSMA3 PE=1 SV=2	PSA3_HUMAN	28 kDa	11	35%
963	Proteasome subunit alpha type-4 OS=Homo sapiens GN=PSMA4 PE=1 SV=1	PSA4_HUMAN	29 kDa	10	49%
964	Proteasome subunit alpha type-5 OS=Homo sapiens GN=PSMA5 PE=1 SV=3	PSA5_HUMAN	26 kDa	8	42%
965	Proteasome subunit alpha type-6 OS=Homo sapiens GN=PSMA6 PE=1 SV=1	PSA6_HUMAN	27 kDa	8	36%
966	Proteasome subunit alpha type-7 OS=Homo sapiens GN=PSMA7 PE=1 SV=1	PSA7_HUMAN	28 kDa	12	56%
967	Proteasome subunit beta type-1 OS=Homo sapiens GN=PSMB1 PE=1 SV=2	PSB1_HUMAN	26 kDa	10	51%
968	Proteasome subunit beta type-2 OS=Homo sapiens GN=PSMB2 PE=1 SV=1	PSB2_HUMAN	23 kDa	7	39%
969	Proteasome subunit beta type-3 OS=Homo sapiens GN=PSMB3 PE=1 SV=2	PSB3_HUMAN	23 kDa	9	47%
970	Proteasome subunit beta type-4 OS=Homo sapiens GN=PSMB4 PE=1 SV=4	PSB4_HUMAN	29 kDa	7	38%
971	Proteasome subunit beta type-5 OS=Homo sapiens GN=PSMB5 PE=1 SV=3	PSB5_HUMAN	28 kDa	2	7.60%
972	Proteasome subunit beta type-6 OS=Homo sapiens GN=PSMB6 PE=1 SV=4	PSB6_HUMAN	25 kDa	4	17%
973	Proteasome subunit beta type-7 OS=Homo sapiens GN=PSMB7 PE=1 SV=1	PSB7_HUMAN	30 kDa	5	19%
974	Proteasome subunit beta type-8 OS=Homo sapiens GN=PSMB8 PE=1 SV=3	PSB8_HUMAN	30 kDa	5	21%
975	Proteasome subunit beta type-9 OS=Homo sapiens GN=PSMB9 PE=1 SV=2	PSB9_HUMAN	23 kDa	2	8.70%
976	26S proteasome non-ATPase regulatory subunit 11 OS=Homo sapiens GN=PSMD11 PE=1 SV=3	PSD11_HUMAN	47 kDa	7	18%
977	26S proteasome non-ATPase regulatory subunit 12 OS=Homo sapiens GN=PSMD12 PE=1 SV=3	PSD12_HUMAN	53 kDa	5	12%
978	26S proteasome non-ATPase regulatory subunit 13 OS=Homo sapiens GN=PSMD13 PE=1 SV=2	PSD13_HUMAN	43 kDa	6	17%
979	26S proteasome non-ATPase regulatory subunit 7 OS=Homo sapiens GN=PSMD7 PE=1 SV=2	PSD7_HUMAN	37 kDa	5	16%
980	26S proteasome non-ATPase regulatory subunit 14 OS=Homo sapiens GN=PSMD14 PE=1 SV=1	PSDE_HUMAN	35 kDa	2	4.50%
981	26S proteasome non-ATPase regulatory subunit 1 OS=Homo sapiens GN=PSMD1 PE=1 SV=2	PSMD1_HUMAN	106 kDa	14	20%
982	26S proteasome non-ATPase regulatory subunit 2 OS=Homo sapiens GN=PSMD2 PE=1 SV=3	PSMD2_HUMAN	100 kDa	8	12%
983	26S proteasome non-ATPase regulatory subunit 3 OS=Homo sapiens GN=PSMD3 PE=1 SV=2	PSMD3_HUMAN	61 kDa	5	10%
984	26S proteasome non-ATPase regulatory subunit 4 OS=Homo sapiens GN=PSMD4 PE=1 SV=1	PSMD4_HUMAN	41 kDa	3	9.50%
985	26S proteasome non-ATPase regulatory subunit 5 OS=Homo sapiens GN=PSMD5 PE=1 SV=3	PSMD5_HUMAN	56 kDa	5	13%
986	26S proteasome non-ATPase regulatory subunit 6 OS=Homo sapiens GN=PSMD6 PE=1 SV=1	PSMD6_HUMAN	46 kDa	4	12%
987	26S proteasome non-ATPase regulatory subunit 8 OS=Homo sapiens GN=PSMD8 PE=1 SV=2	PSMD8_HUMAN	40 kDa	5	16%

#	Proteins	Uniprot ID	Molecular Weight	Number of unique peptides	Percent of coverage
988	Proteasome activator complex subunit 1 OS=Homo sapiens GN=PSME1 PE=1 SV=1	PSME1_HUMAN	29 kDa	16	55%
989	Proteasome activator complex subunit 2 OS=Homo sapiens GN=PSME2 PE=1 SV=4	PSME2_HUMAN	27 kDa	10	41%
990	Proteasome inhibitor PI31 subunit OS=Homo sapiens GN=PSMF1 PE=1 SV=2	PSMF1_HUMAN	30 kDa	4	18%
991	Proteasome assembly chaperone 1 OS=Homo sapiens GN=PSMG1 PE=1 SV=1	PSMG1_HUMAN	33 kDa	2	10%
992	Prostaglandin reductase 1 OS=Homo sapiens GN=PTGR1 PE=1 SV=2	PTGR1_HUMAN	36 kDa	11	37%
993	Parathyroid hormone-related protein OS=Homo sapiens GN=PTHLH PE=1 SV=1	PTHR_HUMAN	20 kDa	6	37%
994	Prothymosin alpha OS=Homo sapiens GN=PTMA PE=1 SV=2	PTMA_HUMAN	12 kDa	2	22%
995	Serine/threonine-protein phosphatase 2A activator OS=Homo sapiens GN=PPP2R4 PE=1 SV=3	PTPA_HUMAN	41 kDa	4	11%
996	Receptor-type tyrosine-protein phosphatase F OS=Homo sapiens GN=PTPRF PE=1 SV=2	PTPRF_HUMAN	213 kDa	12	9.00%
997	Receptor-type tyrosine-protein phosphatase eta OS=Homo sapiens GN=PTPRJ PE=1 SV=3	PTPRJ_HUMAN	146 kDa	3	2.50%
998	Receptor-type tyrosine-protein phosphatase kappa OS=Homo sapiens GN=PTPRK PE=1 SV=2	PTPRK_HUMAN	162 kDa	5	4.10%
999	Putative peptidyl-tRNA hydrolase PTRHD1 OS=Homo sapiens GN=PTRHD1 PE=1 SV=1	PTRD1_HUMAN	16 kDa	5	49%
1000	Trifunctional purine biosynthetic protein adenosine-3 OS=Homo sapiens GN=GART PE=1 SV=1	PUR2_HUMAN	108 kDa	10	16%
1001	Multifunctional protein ADE2 OS=Homo sapiens GN=PAICS PE=1 SV=3	PUR6_HUMAN	47 kDa	7	16%
1002	Bifunctional purine biosynthesis protein PURH OS=Homo sapiens GN=ATIC PE=1 SV=3	PUR9_HUMAN	65 kDa	6	12%
1003	Transcriptional activator protein Pur-alpha OS=Homo sapiens GN=PURA PE=1 SV=2	PURA_HUMAN	35 kDa	3	13%
1004	Adenylosuccinate synthetase isozyme 2 OS=Homo sapiens GN=ADSS PE=1 SV=3	PURA2_HUMAN	50 kDa	3	8.30%
1005	Peroxidasin homolog OS=Homo sapiens GN=PXDN PE=1 SV=2	PXDN_HUMAN	165 kDa	4	4.30%
1006	Glycogen phosphorylase, brain form OS=Homo sapiens GN=PYGB PE=1 SV=5	PYGB_HUMAN	97 kDa	31	41%
1007	Glycogen phosphorylase, liver form OS=Homo sapiens GN=PYGL PE=1 SV=4	PYGL_HUMAN	97 kDa	20	32%
1008	Pregnancy zone protein OS=Homo sapiens GN=PZP PE=1 SV=4	PZP_HUMAN	164 kDa	14	17%
1009	Quinone oxidoreductase OS=Homo sapiens GN=CRYZ PE=1 SV=1	QOR_HUMAN	35 kDa	4	16%
1010	Quinone oxidoreductase PIG3 OS=Homo sapiens GN=TP53I3 PE=1 SV=2	QORX_HUMAN	36 kDa	3	12%
1011	Glutaminyl-peptide cyclotransferase OS=Homo sapiens GN=QPCT PE=1 SV=1	QPCT_HUMAN	41 kDa	2	8.00%
1012	Sulfhydryl oxidase 1 OS=Homo sapiens GN=QSOX1 PE=1 SV=3	QSOX1_HUMAN	83 kDa	43	56%
1013	Ras-related protein Rab-10 OS=Homo sapiens GN=RAB10 PE=1 SV=1	RAB10_HUMAN	23 kDa	5	34%
1014	Ras-related protein Rab-14 OS=Homo sapiens GN=RAB14 PE=1 SV=4	RAB14_HUMAN	24 kDa	7	53%
1015	Ras-related protein Rab-18 OS=Homo sapiens GN=RAB18 PE=1 SV=1	RAB18_HUMAN	23 kDa	10	59%
1016	Ras-related protein Rab-1A OS=Homo sapiens GN=RAB1A PE=1 SV=3	RAB1A_HUMAN	23 kDa	16	75%
1017	Ras-related protein Rab-1B OS=Homo sapiens GN=RAB1B PE=1 SV=1	RAB1B_HUMAN	22 kDa	6	66%
1018	Ras-related protein Rab-21 OS=Homo sapiens GN=RAB21 PE=1 SV=3	RAB21_HUMAN	24 kDa	8	35%
1019	Ras-related protein Rab-25 OS=Homo sapiens GN=RAB25 PE=1 SV=2	RAB25_HUMAN	23 kDa	4	23%
1020	Ras-related protein Rab-2A OS=Homo sapiens GN=RAB2A PE=1 SV=1	RAB2A_HUMAN	24 kDa	8	45%
1021	Ras-related protein Rab-35 OS=Homo sapiens GN=RAB35 PE=1 SV=1	RAB35_HUMAN	23 kDa	2	26%
1022	Ras-related protein Rab-3D OS=Homo sapiens GN=RAB3D PE=1 SV=1	RAB3D_HUMAN	24 kDa	4	28%
1023	Ras-related protein Rab-5A OS=Homo sapiens GN=RAB5A PE=1 SV=2	RAB5A_HUMAN	24 kDa	4	42%
1024	Ras-related protein Rab-5B OS=Homo sapiens GN=RAB5B PE=1 SV=1	RAB5B_HUMAN	24 kDa	9	54%
1025	Ras-related protein Rab-5C OS=Homo sapiens GN=RAB5C PE=1 SV=2	RAB5C_HUMAN	23 kDa	5	48%
1026	Ras-related protein Rab-6A OS=Homo sapiens GN=RAB6A PE=1 SV=3	RAB6A_HUMAN	24 kDa	4	25%
1027	Ras-related protein Rab-7a OS=Homo sapiens GN=RAB7A PE=1 SV=1	RAB7A_HUMAN	23 kDa	12	61%

#	Proteins	Uniprot ID	Molecular Weight	Number of unique peptides	Percent of coverage
1028	Ras-related protein Rab-8A OS=Homo sapiens GN=RAB8A PE=1 SV=1	RAB8A_HUMAN	24 kDa	5	36%
1029	Cellular retinoic acid-binding protein 1 OS=Homo sapiens GN=CRABP1 PE=1 SV=2	RABP1_HUMAN	16 kDa	5	46%
1030	Cellular retinoic acid-binding protein 2 OS=Homo sapiens GN=CRABP2 PE=1 SV=2	RABP2_HUMAN	16 kDa	7	47%
1031	Ras-related C3 botulinum toxin substrate 1 OS=Homo sapiens GN=RAC1 PE=1 SV=1	RAC1_HUMAN	21 kDa	7	31%
1032	DNA repair protein RAD50 OS=Homo sapiens GN=RAD50 PE=1 SV=1	RAD50_HUMAN	154 kDa	2	1.50%
1033	Radixin OS=Homo sapiens GN=RDX PE=1 SV=1	RADI_HUMAN	69 kDa	16	37%
1034	Ras-related protein Ral-B OS=Homo sapiens GN=RALB PE=1 SV=1	RALB_HUMAN	23 kDa	5	28%
1035	GTP-binding nuclear protein Ran OS=Homo sapiens GN=RAN PE=1 SV=3	RAN_HUMAN	24 kDa	4	21%
1036	Ran-specific GTPase-activating protein OS=Homo sapiens GN=RANBP1 PE=1 SV=1	RANG_HUMAN	23 kDa	2	16%
1037	Ras-related protein Rap-1b OS=Homo sapiens GN=RAP1B PE=1 SV=1	RAP1B_HUMAN	21 kDa	9	51%
1038	Ras-related protein Rap-2c OS=Homo sapiens GN=RAP2C PE=1 SV=1	RAP2C_HUMAN	21 kDa	2	11%
1039	GTPase KRas OS=Homo sapiens GN=KRAS PE=1 SV=1	RASK_HUMAN	22 kDa	2	12%
1040	Ras-related protein Rab-11B OS=Homo sapiens GN=RAB11B PE=1 SV=4	RB11B_HUMAN	24 kDa	10	46%
1041	Reticulocalbin-1 OS=Homo sapiens GN=RCN1 PE=1 SV=1	RCN1_HUMAN	39 kDa	3	12%
1042	UV excision repair protein RAD23 homolog A OS=Homo sapiens GN=RAD23A PE=1 SV=1	RD23A_HUMAN	40 kDa	3	18%
1043	UV excision repair protein RAD23 homolog B OS=Homo sapiens GN=RAD23B PE=1 SV=1	RD23B_HUMAN	43 kDa	11	33%
1044	Reversion-inducing cysteine-rich protein with Kazal motifs OS=Homo sapiens GN=RECK PE=1 SV=1	RECK_HUMAN	106 kDa	3	3.40%
1045	Receptor expression-enhancing protein 5 OS=Homo sapiens GN=REEP5 PE=1 SV=3	REEP5_HUMAN	21 kDa	3	10%
1046	Regulator of nonsense transcripts 1 OS=Homo sapiens GN=UPF1 PE=1 SV=2	RENT1_HUMAN	124 kDa	3	2.60%
1047	Retinol-binding protein 1 OS=Homo sapiens GN=RBP1 PE=1 SV=2	RET1_HUMAN	16 kDa	6	58%
1048	Retinol-binding protein 4 OS=Homo sapiens GN=RBP4 PE=1 SV=3	RET4_HUMAN	23 kDa	3	15%
1049	GTP-binding protein Rheb OS=Homo sapiens GN=RHEB PE=1 SV=1	RHEB_HUMAN	20 kDa	2	11%
1050	Rho GTPase-activating protein 1 OS=Homo sapiens GN=ARHGAP1 PE=1 SV=1	RHG01_HUMAN	50 kDa	2	7.50%
1051	Rho GTPase-activating protein 18 OS=Homo sapiens GN=ARHGAP18 PE=1 SV=3	RHG18_HUMAN	75 kDa	21	37%
1052	Transforming protein RhoA OS=Homo sapiens GN=RHOA PE=1 SV=1	RHOA_HUMAN	22 kDa	9	50%
1053	Ribonuclease inhibitor OS=Homo sapiens GN=RNH1 PE=1 SV=2	RINI_HUMAN	50 kDa	16	50%
1054	Retinoid-inducible serine carboxypeptidase OS=Homo sapiens GN=SCPEP1 PE=1 SV=1	RISC_HUMAN	51 kDa	16	40%
1055	60S ribosomal protein L10a OS=Homo sapiens GN=RPL10A PE=1 SV=2	RL10A_HUMAN	25 kDa	4	23%
1056	60S ribosomal protein L23 OS=Homo sapiens GN=RPL23 PE=1 SV=1	RL23_HUMAN	15 kDa	2	20%
1057	60S ribosomal protein L3 OS=Homo sapiens GN=RPL3 PE=1 SV=2	RL3_HUMAN	46 kDa	2	3.70%
1058	60S ribosomal protein L34 OS=Homo sapiens GN=RPL34 PE=1 SV=3	RL34_HUMAN	13 kDa	2	13%
1059	60S ribosomal protein L4 OS=Homo sapiens GN=RPL4 PE=1 SV=5	RL4_HUMAN	48 kDa	4	11%
1060	Ubiquitin-60S ribosomal protein L40 OS=Homo sapiens GN=UBA52 PE=1 SV=2	RL40_HUMAN (+3)	15 kDa	5	38%
1061	60S ribosomal protein L6 OS=Homo sapiens GN=RPL6 PE=1 SV=3	RL6_HUMAN	33 kDa	3	14%
1062	60S acidic ribosomal protein P0 OS=Homo sapiens GN=RPLP0 PE=1 SV=1	RLA0_HUMAN	34 kDa	5	24%
1063	60S acidic ribosomal protein P2 OS=Homo sapiens GN=RPLP2 PE=1 SV=1	RLA2_HUMAN	12 kDa	3	38%
1064	Ribonuclease 4 OS=Homo sapiens GN=RNASE4 PE=1 SV=3	RNAS4_HUMAN	17 kDa	6	44%
1065	Ribonuclease T2 OS=Homo sapiens GN=RNASET2 PE=1 SV=2	RNT2_HUMAN	29 kDa	7	29%
1066	Tyrosine-protein kinase transmembrane receptor ROR2 OS=Homo sapiens GN=ROR2 PE=1 SV=2	ROR2_HUMAN	105 kDa	2	2.00%
1067	Ras-related protein R-Ras OS=Homo sapiens GN=RRAS PE=1 SV=1	RRAS_HUMAN	23 kDa	4	21%

#	Proteins	Uniprot ID	Molecular Weight	Number of unique peptides	Percent of coverage
1068	Ras-related protein R-Ras2 OS=Homo sapiens GN=RRAS2 PE=1 SV=1	RRAS2_HUMAN	23 kDa	2	17%
1069	Ribosome-binding protein 1 OS=Homo sapiens GN=RRBP1 PE=1 SV=4	RRBP1_HUMAN	152 kDa	32	22%
1070	40S ribosomal protein S12 OS=Homo sapiens GN=RPS12 PE=1 SV=3	RS12_HUMAN	15 kDa	4	39%
1071	40S ribosomal protein S13 OS=Homo sapiens GN=RPS13 PE=1 SV=2	RS13_HUMAN	17 kDa	2	16%
1072	40S ribosomal protein S16 OS=Homo sapiens GN=RPS16 PE=1 SV=2	RS16_HUMAN	16 kDa	2	12%
1073	40S ribosomal protein S18 OS=Homo sapiens GN=RPS18 PE=1 SV=3	RS18_HUMAN	18 kDa	3	19%
1074	40S ribosomal protein S2 OS=Homo sapiens GN=RPS2 PE=1 SV=2	RS2_HUMAN	31 kDa	4	12%
1075	40S ribosomal protein S28 OS=Homo sapiens GN=RPS28 PE=1 SV=1	RS28_HUMAN	8 kDa	2	33%
1076	40S ribosomal protein S3 OS=Homo sapiens GN=RPS3 PE=1 SV=2	RS3_HUMAN	27 kDa	2	7.00%
1077	40S ribosomal protein S3a OS=Homo sapiens GN=RPS3A PE=1 SV=2	RS3A_HUMAN	30 kDa	2	8.30%
1078	40S ribosomal protein S5 OS=Homo sapiens GN=RPS5 PE=1 SV=4	RS5_HUMAN	23 kDa	2	14%
1079	40S ribosomal protein S7 OS=Homo sapiens GN=RPS7 PE=1 SV=1	RS7_HUMAN	22 kDa	2	10%
1080	40S ribosomal protein SA OS=Homo sapiens GN=RPSA PE=1 SV=4	RSSA_HUMAN	33 kDa	3	9.20%
1081	Protein S100-B OS=Homo sapiens GN=S100B PE=1 SV=2	S100B_HUMAN	11 kDa	2	33%
1082	Protein S100-A1 OS=Homo sapiens GN=S100A1 PE=1 SV=2	S10A1_HUMAN	11 kDa	4	49%
1083	Protein S100-A6 OS=Homo sapiens GN=S100A6 PE=1 SV=1	S10A6_HUMAN	10 kDa	2	17%
1084	Protein S100-A8 OS=Homo sapiens GN=S100A8 PE=1 SV=1	S10A8_HUMAN	11 kDa	2	25%
1085	Protein S100-A9 OS=Homo sapiens GN=S100A9 PE=1 SV=1	S10A9_HUMAN	13 kDa	3	32%
1086	Protein S100-A11 OS=Homo sapiens GN=S100A11 PE=1 SV=2	S10AB_HUMAN	12 kDa	7	58%
1087	Protein S100-A13 OS=Homo sapiens GN=S100A13 PE=1 SV=1	S10AD_HUMAN	11 kDa	6	49%
1088	Protein S100-A14 OS=Homo sapiens GN=S100A14 PE=1 SV=1	S10AE_HUMAN	12 kDa	3	34%
1089	Protein S100-A16 OS=Homo sapiens GN=S100A16 PE=1 SV=1	S10AG_HUMAN	12 kDa	4	42%
1090	Putative sodium-coupled neutral amino acid transporter 10 OS=Homo sapiens GN=SLC38A10 PE=1 SV=2	S38AA_HUMAN	120 kDa	11	14%
1091	Serum amyloid A protein OS=Homo sapiens GN=SAA1 PE=1 SV=2	SAA_HUMAN	14 kDa	3	41%
1092	Serum amyloid A-4 protein OS=Homo sapiens GN=SAA4 PE=1 SV=2	SAA4_HUMAN	15 kDa	3	22%
1093	Adenosylhomocysteinase OS=Homo sapiens GN=AHCY PE=1 SV=4	SAHH_HUMAN	48 kDa	13	35%
1094	Putative adenosylhomocysteinase 2 OS=Homo sapiens GN=AHCYL1 PE=1 SV=2	SAHH2_HUMAN	59 kDa	3	7.40%
1095	Serum amyloid P-component OS=Homo sapiens GN=APCS PE=1 SV=2	SAMP_HUMAN	25 kDa	2	11%
1096	Proactivator polypeptide OS=Homo sapiens GN=PSAP PE=1 SV=2	SAP_HUMAN	58 kDa	26	56%
1097	Ganglioside GM2 activator OS=Homo sapiens GN=GM2A PE=1 SV=4	SAP3_HUMAN	21 kDa	5	29%
1098	GTP-binding protein SAR1a OS=Homo sapiens GN=SAR1A PE=1 SV=1	SAR1A_HUMAN	22 kDa	7	47%
1099	GTP-binding protein SAR1b OS=Homo sapiens GN=SAR1B PE=1 SV=1	SAR1B_HUMAN	22 kDa	2	29%
1100	S-acyl fatty acid synthase thioesterase, medium chain OS=Homo sapiens GN=OLAH PE=2 SV=1	SAST_HUMAN	30 kDa	12	55%
1101	Ribosome maturation protein SBDS OS=Homo sapiens GN=SBDS PE=1 SV=4	SBDS_HUMAN	29 kDa	3	10.00%
1102	Selenium-binding protein 1 OS=Homo sapiens GN=SELENBP1 PE=1 SV=2	SBP1_HUMAN	52 kDa	26	62%
1103	Vesicle-trafficking protein SEC22b OS=Homo sapiens GN=SEC22B PE=1 SV=4	SC22B_HUMAN	25 kDa	3	13%
1104	Protein transport protein Sec23B OS=Homo sapiens GN=SEC23B PE=1 SV=2	SC23B_HUMAN	86 kDa	3	4.60%
1105	Protein transport protein Sec31A OS=Homo sapiens GN=SEC31A PE=1 SV=3	SC31A_HUMAN	133 kDa	3	3.60%
1106	Sec1 family domain-containing protein 1 OS=Homo sapiens GN=SCFD1 PE=1 SV=4	SCFD1_HUMAN	72 kDa	3	6.90%
1107	Secernin-3 OS=Homo sapiens GN=SCRN3 PE=1 SV=1	SCRN3_HUMAN	49 kDa	2	4.20%
1108	Syndecan-2 OS=Homo sapiens GN=SDC2 PE=1 SV=2	SDC2_HUMAN	22 kDa	3	15%
1109	Syndecan-4 OS=Homo sapiens GN=SDC4 PE=1 SV=2	SDC4_HUMAN	22 kDa	5	33%

#	Proteins	Uniprot ID	Molecular Weight	Number of unique peptides	Percent of coverage
1110	Syntenin-1 OS=Homo sapiens GN=SDCBP PE=1 SV=1	SDCB1_HUMAN	32 kDa	8	40%
1111	Protein SEC13 homolog OS=Homo sapiens GN=SEC13 PE=1 SV=3	SEC13_HUMAN	36 kDa	2	7.50%
1112	Selenoprotein M OS=Homo sapiens GN=SELM PE=1 SV=3	SELM_HUMAN	16 kDa	3	30%
1113	Semaphorin-4B OS=Homo sapiens GN=SEMA4B PE=1 SV=3	SEM4B_HUMAN	92 kDa	4	4.20%
1114	Selenoprotein P OS=Homo sapiens GN=SEPP1 PE=1 SV=3	SEPP1_HUMAN	43 kDa	6	16%
1115	Septin-2 OS=Homo sapiens GN=SEPT2 PE=1 SV=1	SEPT2_HUMAN	41 kDa	2	5.80%
1116	Septin-9 OS=Homo sapiens GN=SEPT9 PE=1 SV=2	SEPT9_HUMAN	65 kDa	2	3.20%
1117	D-3-phosphoglycerate dehydrogenase OS=Homo sapiens GN=PHGDH PE=1 SV=4	SERA_HUMAN	57 kDa	14	30%
1118	Secreted frizzled-related protein 1 OS=Homo sapiens GN=SFRP1 PE=1 SV=1	SFRP1_HUMAN	35 kDa	2	7.00%
1119	Small glutamine-rich tetratricopeptide repeat-containing protein alpha OS=Homo sapiens GN=SGTA PE=1 SV=1	SGTA_HUMAN	34 kDa	2	7.70%
1120	SH3 domain-binding protein 4 OS=Homo sapiens GN=SH3BP4 PE=1 SV=1	SH3B4_HUMAN	107 kDa	3	3.70%
1121	SH3 domain-binding glutamic acid-rich-like protein OS=Homo sapiens GN=SH3BGRL PE=1 SV=1	SH3L1_HUMAN	13 kDa	2	19%
1122	SH3 domain-binding glutamic acid-rich-like protein 3 OS=Homo sapiens GN=SH3BGRL3 PE=1 SV=1	SH3L3_HUMAN	10 kDa	3	40%
1123	Sex hormone-binding globulin OS=Homo sapiens GN=SHBG PE=1 SV=2	SHBG_HUMAN	44 kDa	5	16%
1124	Shootin-1 OS=Homo sapiens GN=KIAA1598 PE=1 SV=4	SHOT1_HUMAN	72 kDa	10	13%
1125	Tyrosine-protein phosphatase non-receptor type substrate 1 OS=Homo sapiens GN=SIRPA PE=1 SV=2	SHPS1_HUMAN	55 kDa	9	21%
1126	Sialate O-acetylesterase OS=Homo sapiens GN=SIAE PE=1 SV=1	SIAE_HUMAN	58 kDa	9	18%
1127	Sialic acid synthase OS=Homo sapiens GN=NANS PE=1 SV=2	SIAS_HUMAN	40 kDa	12	36%
1128	Nucleotide exchange factor SIL1 OS=Homo sapiens GN=SIL1 PE=1 SV=1	SIL1_HUMAN	52 kDa	16	38%
1129	S-phase kinase-associated protein 1 OS=Homo sapiens GN=SKP1 PE=1 SV=2	SKP1_HUMAN	19 kDa	4	25%
1130	STE20-like serine/threonine-protein kinase OS=Homo sapiens GN=SLK PE=1 SV=1	SLK_HUMAN	143 kDa	3	2.30%
1131	Alpha-soluble NSF attachment protein OS=Homo sapiens GN=NAPA PE=1 SV=3	SNAA_HUMAN	33 kDa	3	14%
1132	Gamma-soluble NSF attachment protein OS=Homo sapiens GN=NAPG PE=1 SV=1	SNAG_HUMAN	35 kDa	2	7.40%
1133	Staphylococcal nuclease domain-containing protein 1 OS=Homo sapiens GN=SND1 PE=1 SV=1	SND1_HUMAN	102 kDa	7	9.20%
1134	Synaptosomal-associated protein 23 OS=Homo sapiens GN=SNAP23 PE=1 SV=1	SNP23_HUMAN	23 kDa	12	69%
1135	Synaptosomal-associated protein 29 OS=Homo sapiens GN=SNAP29 PE=1 SV=1	SNP29_HUMAN	29 kDa	3	13%
1136	Sorting nexin-12 OS=Homo sapiens GN=SNX12 PE=1 SV=3	SNX12_HUMAN	20 kDa	3	30%
1137	Sorting nexin-9 OS=Homo sapiens GN=SNX9 PE=1 SV=1	SNX9_HUMAN	67 kDa	2	4.40%
1138	Superoxide dismutase [Cu-Zn] OS=Homo sapiens GN=SOD1 PE=1 SV=2	SODC_HUMAN	16 kDa	7	53%
1139	Extracellular superoxide dismutase [Cu-Zn] OS=Homo sapiens GN=SOD3 PE=1 SV=2	SODE_HUMAN	26 kDa	6	30%
1140	Sortilin OS=Homo sapiens GN=SORT1 PE=1 SV=3	SORT_HUMAN	92 kDa	6	7.80%
1141	Sclerostin domain-containing protein 1 OS=Homo sapiens GN=SOSTDC1 PE=1 SV=2	SOSD1_HUMAN	23 kDa	5	33%
1142	Serpin B6 OS=Homo sapiens GN=SERPINB6 PE=1 SV=3	SPB6_HUMAN	43 kDa	5	16%
1143	Spermidine synthase OS=Homo sapiens GN=SRM PE=1 SV=1	SPEE_HUMAN	34 kDa	2	4.60%
1144	Kunitz-type protease inhibitor 1 OS=Homo sapiens GN=SPINT1 PE=1 SV=2	SPIT1_HUMAN	58 kDa	17	32%
1145	Kunitz-type protease inhibitor 2 OS=Homo sapiens GN=SPINT2 PE=1 SV=2	SPIT2_HUMAN	28 kDa	3	15%
1146	Sepiapterin reductase OS=Homo sapiens GN=SPR PE=1 SV=1	SPRE_HUMAN	28 kDa	3	19%
1147	SPARC-like protein 1 OS=Homo sapiens GN=SPARCL1 PE=1 SV=2	SPRL1_HUMAN	75 kDa	2	3.60%
1148	Spermine synthase OS=Homo sapiens GN=SMS PE=1 SV=2	SPSY_HUMAN	41 kDa	7	20%
1149	Spectrin alpha chain, brain OS=Homo sapiens GN=SPTAN1 PE=1 SV=3	SPTA2_HUMAN	285 kDa	4	2.40%

#	Proteins	Uniprot ID	Molecular Weight	Number of unique peptides	Percent of coverage
1150	Src substrate cortactin OS=Homo sapiens GN=CTTN PE=1 SV=2	SRC8_HUMAN	62 kDa	9	16%
1151	Signal recognition particle 9 kDa protein OS=Homo sapiens GN=SRP9 PE=1 SV=2	SRP09_HUMAN	10 kDa	2	22%
1152	Signal recognition particle 14 kDa protein OS=Homo sapiens GN=SRP14 PE=1 SV=2	SRP14_HUMAN	15 kDa	2	20%
1153	Suppressor of tumorigenicity 14 protein OS=Homo sapiens GN=ST14 PE=1 SV=2	ST14_HUMAN	95 kDa	9	14%
1154	Signal transducer and activator of transcription 5A OS=Homo sapiens GN=STAT5A PE=1 SV=1	STA5A_HUMAN	91 kDa	11	15%
1155	Signal transducer and activator of transcription 3 OS=Homo sapiens GN=STAT3 PE=1 SV=2	STAT3_HUMAN	88 kDa	3	6.40%
1156	Double-stranded RNA-binding protein Staufen homolog 1 OS=Homo sapiens GN=STAU1 PE=1 SV=2	STAU1_HUMAN	63 kDa	4	8.10%
1157	Stanniocalcin-1 OS=Homo sapiens GN=STC1 PE=1 SV=1	STC1_HUMAN	28 kDa	3	11%
1158	Stress-induced-phosphoprotein 1 OS=Homo sapiens GN=STIP1 PE=1 SV=1	STIP1_HUMAN	63 kDa	21	40%
1159	Serine/threonine-protein kinase 24 OS=Homo sapiens GN=STK24 PE=1 SV=1	STK24_HUMAN	49 kDa	3	8.80%
1160	Serine/threonine-protein kinase 3 OS=Homo sapiens GN=STK3 PE=1 SV=2	STK3_HUMAN	56 kDa	8	18%
1161	Erythrocyte band 7 integral membrane protein OS=Homo sapiens GN=STOM PE=1 SV=3	STOM_HUMAN	32 kDa	9	45%
1162	Serine-threonine kinase receptor-associated protein OS=Homo sapiens GN=STRAP PE=1 SV=1	STRAP_HUMAN	38 kDa	3	9.40%
1163	Syntaxin-12 OS=Homo sapiens GN=STX12 PE=1 SV=1	STX12_HUMAN	32 kDa	2	8.70%
1164	Syntaxin-3 OS=Homo sapiens GN=STX3 PE=1 SV=3	STX3_HUMAN	33 kDa	7	21%
1165	Syntaxin-binding protein 1 OS=Homo sapiens GN=STXBP1 PE=1 SV=1	STXB1_HUMAN	68 kDa	9	18%
1166	Syntaxin-binding protein 2 OS=Homo sapiens GN=STXBP2 PE=1 SV=2	STXB2_HUMAN	66 kDa	26	51%
1167	Suppressor of G2 allele of SKP1 homolog OS=Homo sapiens GN=SUGT1 PE=1 SV=3	SUGT1_HUMAN	41 kDa	3	7.40%
1168	Extracellular sulfatase Sulf-2 OS=Homo sapiens GN=SULF2 PE=1 SV=1	SULF2_HUMAN	100 kDa	5	5.90%
1169	Sulfatase-modifying factor 2 OS=Homo sapiens GN=SUMF2 PE=1 SV=2	SUMF2_HUMAN	34 kDa	4	18%
1170	Small ubiquitin-related modifier 2 OS=Homo sapiens GN=SUMO2 PE=1 SV=2	SUMO2_HUMAN	11 kDa	2	23%
1171	Alanyl-tRNA synthetase, cytoplasmic OS=Homo sapiens GN=AARS PE=1 SV=2	SYAC_HUMAN	107 kDa	8	10%
1172	Aspartyl-tRNA synthetase, cytoplasmic OS=Homo sapiens GN=DARS PE=1 SV=2	SYDC_HUMAN	57 kDa	8	17%
1173	Bifunctional aminoacyl-tRNA synthetase OS=Homo sapiens GN=EPRS PE=1 SV=5	SYEP_HUMAN	171 kDa	18	15%
1174	Phenylalanyl-tRNA synthetase alpha chain OS=Homo sapiens GN=FARSA PE=1 SV=3	SYFA_HUMAN	58 kDa	2	4.70%
1175	Phenylalanyl-tRNA synthetase beta chain OS=Homo sapiens GN=FARSB PE=1 SV=3	SYFB_HUMAN	66 kDa	6	9.20%
1176	Glycyl-tRNA synthetase OS=Homo sapiens GN=GARS PE=1 SV=3	SYG_HUMAN	83 kDa	8	9.50%
1177	Histidyl-tRNA synthetase, cytoplasmic OS=Homo sapiens GN=HARS PE=1 SV=2	SYHC_HUMAN	57 kDa	7	15%
1178	Isoleucyl-tRNA synthetase, cytoplasmic OS=Homo sapiens GN=IARS PE=1 SV=2	SYIC_HUMAN	145 kDa	3	2.90%
1179	Lysyl-tRNA synthetase OS=Homo sapiens GN=KARS PE=1 SV=3	SYK_HUMAN	68 kDa	8	15%
1180	Leucyl-tRNA synthetase, cytoplasmic OS=Homo sapiens GN=LARS PE=1 SV=2	SYLC_HUMAN	134 kDa	5	5.30%
1181	Asparaginyl-tRNA synthetase, cytoplasmic OS=Homo sapiens GN=NARS PE=1 SV=1	SYNC_HUMAN	63 kDa	8	17%
1182	Glutaminyl-tRNA synthetase OS=Homo sapiens GN=QARS PE=1 SV=1	SYQ_HUMAN	88 kDa	7	10%
1183	Arginyl-tRNA synthetase, cytoplasmic OS=Homo sapiens GN=RARS PE=1 SV=2	SYRC_HUMAN	75 kDa	9	15%
1184	Seryl-tRNA synthetase, cytoplasmic OS=Homo sapiens GN=SARS PE=1 SV=3	SYSC_HUMAN	59 kDa	7	14%
1185	Threonyl-tRNA synthetase, cytoplasmic OS=Homo sapiens GN=TARS PE=1 SV=3	SYTC_HUMAN	83 kDa	14	19%
1186	Valyl-tRNA synthetase OS=Homo sapiens GN=VARS PE=1 SV=4	SYVC_HUMAN	140 kDa	7	7.00%
1187	Tryptophanyl-tRNA synthetase, cytoplasmic OS=Homo sapiens GN=WARS PE=1 SV=2	SYWC_HUMAN	53 kDa	16	41%

#	Proteins	Uniprot ID	Molecular Weight	Number of unique peptides	Percent of coverage
1188	Tyrosyl-tRNA synthetase, cytoplasmic OS=Homo sapiens GN=YARS PE=1 SV=4	SYYC_HUMAN	59 kDa	7	14%
1189	Transmembrane protein 132A OS=Homo sapiens GN=TMEM132A PE=2 SV=1	T132A_HUMAN	110 kDa	4	5.80%
1190	Tandem C2 domains nuclear protein OS=Homo sapiens GN=TC2N PE=1 SV=2	TAC2N_HUMAN	55 kDa	4	9.60%
1191	Tumor-associated calcium signal transducer 2 OS=Homo sapiens GN=TACSTD2 PE=1 SV=3	TACD2_HUMAN	36 kDa	3	12%
1192	Transgelin-2 OS=Homo sapiens GN=TAGLN2 PE=1 SV=3	TAGL2_HUMAN	22 kDa	10	55%
1193	Transaldolase OS=Homo sapiens GN=TALDO1 PE=1 SV=2	TALDO_HUMAN	38 kDa	9	28%
1194	Serine/threonine-protein kinase TAO3 OS=Homo sapiens GN=TAOK3 PE=1 SV=2	TAOK3_HUMAN	105 kDa	2	1.90%
1195	Tax1-binding protein 1 OS=Homo sapiens GN=TAX1BP1 PE=1 SV=2	TAXB1_HUMAN	91 kDa	2	2.40%
1196	Tubulin alpha-1B chain OS=Homo sapiens GN=TUBA1B PE=1 SV=1	TBA1B_HUMAN	50 kDa	11	34%
1197	Tubulin beta chain OS=Homo sapiens GN=TUBB PE=1 SV=2	TBB5_HUMAN	50 kDa	12	32%
1198	TBC1 domain family member 24 OS=Homo sapiens GN=TBC1D24 PE=1 SV=2	TBC24_HUMAN	63 kDa	3	7.20%
1199	Tubulin-specific chaperone A OS=Homo sapiens GN=TBCA PE=1 SV=3	TBCA_HUMAN	13 kDa	7	38%
1200	Tubulin-specific chaperone D OS=Homo sapiens GN=TBCD PE=1 SV=2	TBCD_HUMAN	133 kDa	2	1.80%
1201	TBC1 domain family member 4 OS=Homo sapiens GN=TBC1D4 PE=1 SV=2	TBCD4_HUMAN	147 kDa	2	2.10%
1202	Transcobalamin-1 OS=Homo sapiens GN=TCN1 PE=1 SV=2	TCO1_HUMAN	48 kDa	16	42%
1203	T-complex protein 1 subunit alpha OS=Homo sapiens GN=TCP1 PE=1 SV=1	TCPA_HUMAN	60 kDa	20	42%
1204	T-complex protein 1 subunit beta OS=Homo sapiens GN=CCT2 PE=1 SV=4	TCPB_HUMAN	57 kDa	17	41%
1205	T-complex protein 1 subunit delta OS=Homo sapiens GN=CCT4 PE=1 SV=4	TCPD_HUMAN	58 kDa	10	28%
1206	T-complex protein 1 subunit epsilon OS=Homo sapiens GN=CCT5 PE=1 SV=1	TCPE_HUMAN	60 kDa	14	33%
1207	T-complex protein 1 subunit gamma OS=Homo sapiens GN=CCT3 PE=1 SV=4	TCPG_HUMAN	61 kDa	17	37%
1208	T-complex protein 1 subunit eta OS=Homo sapiens GN=CCT7 PE=1 SV=2	TCPH_HUMAN	59 kDa	15	36%
1209	T-complex protein 1 subunit theta OS=Homo sapiens GN=CCT8 PE=1 SV=4	TCPQ_HUMAN	60 kDa	25	47%
1210	T-complex protein 1 subunit zeta OS=Homo sapiens GN=CCT6A PE=1 SV=3	TCPZ_HUMAN	58 kDa	14	33%
1211	Translationally-controlled tumor protein OS=Homo sapiens GN=TPT1 PE=1 SV=1	TCTP_HUMAN	20 kDa	5	32%
1212	Tudor domain-containing protein 6 OS=Homo sapiens GN=TDRD6 PE=2 SV=2	TDRD6_HUMAN	237 kDa	2	0.62%
1213	Prostaglandin E synthase 3 OS=Homo sapiens GN=PTGES3 PE=1 SV=1	TEBP_HUMAN	19 kDa	3	23%
1214	Tenascin OS=Homo sapiens GN=TNC PE=1 SV=3	TENA_HUMAN	241 kDa	95	56%
1215	Transitional endoplasmic reticulum ATPase OS=Homo sapiens GN=VCP PE=1 SV=4	TERA_HUMAN	89 kDa	37	47%
1216	Tetranectin OS=Homo sapiens GN=CLEC3B PE=1 SV=3	TETN_HUMAN	23 kDa	5	34%
1217	Protein TFG OS=Homo sapiens GN=TFG PE=1 SV=2	TFG_HUMAN	43 kDa	5	13%
1218	Transforming growth factor beta receptor type 3 OS=Homo sapiens GN=TGFBR3 PE=1 SV=3	TGBR3_HUMAN	94 kDa	7	10%
1219	Transforming growth factor beta-2 OS=Homo sapiens GN=TGFB2 PE=1 SV=1	TGFB2_HUMAN	48 kDa	2	4.30%
1220	Trans-Golgi network integral membrane protein 2 OS=Homo sapiens GN=TGOLN2 PE=1 SV=2	TGON2_HUMAN	51 kDa	12	28%
1221	Thyroxine-binding globulin OS=Homo sapiens GN=SERPINA7 PE=1 SV=2	THBG_HUMAN	46 kDa	9	22%
1222	Acetyl-CoA acetyltransferase, cytosolic OS=Homo sapiens GN=ACAT2 PE=1 SV=2	THIC_HUMAN	41 kDa	13	55%
1223	3-ketoacyl-CoA thiolase, peroxisomal OS=Homo sapiens GN=ACAA1 PE=1 SV=2	THIK_HUMAN	44 kDa	2	6.40%
1224	Thioredoxin OS=Homo sapiens GN=TXN PE=1 SV=3	THIO_HUMAN	12 kDa	6	54%
1225	Prothrombin OS=Homo sapiens GN=F2 PE=1 SV=2	THRB_HUMAN	70 kDa	19	42%
1226	3-mercaptopyruvate sulfurtransferase OS=Homo sapiens GN=MPST PE=1 SV=3	THTM_HUMAN	33 kDa	2	7.40%

#	Proteins	Uniprot ID	Molecular Weight	Number of unique peptides	Percent of coverage
1227	Tyrosine-protein kinase receptor Tie-1 OS=Homo sapiens GN=TIE1 PE=1 SV=1	TIE1_HUMAN	125 kDa	2	1.40%
1228	Retinoic acid receptor responder protein 1 OS=Homo sapiens GN=RARRES1 PE=2 SV=2	TIG1_HUMAN	33 kDa	2	7.50%
1229	Metalloproteinase inhibitor 1 OS=Homo sapiens GN=TIMP1 PE=1 SV=1	TIMP1_HUMAN	23 kDa	6	42%
1230	Metalloproteinase inhibitor 4 OS=Homo sapiens GN=TIMP4 PE=1 SV=1	TIMP4_HUMAN	26 kDa	4	22%
1231	T-cell immunomodulatory protein OS=Homo sapiens GN=ITFG1 PE=1 SV=1	TIP_HUMAN	68 kDa	4	9.20%
1232	Titin OS=Homo sapiens GN=TTN PE=1 SV=2	TITIN_HUMAN	3816 kDa	2	0.04%
1233	Transketolase OS=Homo sapiens GN=TKT PE=1 SV=3	TKT_HUMAN	68 kDa	21	47%
1234	Talin-1 OS=Homo sapiens GN=TLN1 PE=1 SV=3	TLN1_HUMAN	270 kDa	18	9.50%
1235	Toll-like receptor 2 OS=Homo sapiens GN=TLR2 PE=1 SV=1	TLR2_HUMAN	90 kDa	15	23%
1236	TOM1-like protein 1 OS=Homo sapiens GN=TOM1L1 PE=1 SV=2	TM1L1_HUMAN	53 kDa	2	4.60%
1237	Transmembrane channel-like protein 5 OS=Homo sapiens GN=TMC5 PE=2 SV=3	TMC5_HUMAN	115 kDa	6	8.30%
1238	Thymosin beta-4-like protein 3 OS=Homo sapiens GN=TMSL3 PE=2 SV=1	TMSL3_HUMAN (+1)	5 kDa	2	41%
1239	Tumor necrosis factor ligand superfamily member 10 OS=Homo sapiens GN=TNFSF10 PE=1 SV=1	TNF10_HUMAN	33 kDa	6	21%
1240	Tumor necrosis factor ligand superfamily member 13 OS=Homo sapiens GN=TNFSF13 PE=1 SV=1	TNF13_HUMAN	27 kDa	5	16%
1241	Transportin-1 OS=Homo sapiens GN=TNPO1 PE=1 SV=2	TNPO1_HUMAN	102 kDa	3	4.50%
1242	Transportin-2 OS=Homo sapiens GN=TNPO2 PE=1 SV=3	TNPO2_HUMAN	101 kDa	2	3.60%
1243	Toll-interacting protein OS=Homo sapiens GN=TOLLIP PE=1 SV=1	TOLIP_HUMAN	30 kDa	3	11%
1244	Target of Myb protein 1 OS=Homo sapiens GN=TOM1 PE=1 SV=2	TOM1_HUMAN	54 kDa	5	12%
1245	Tumor protein D52 OS=Homo sapiens GN=TPD52 PE=1 SV=2	TPD52_HUMAN	24 kDa	7	38%
1246	Tumor protein D53 OS=Homo sapiens GN=TPD52L1 PE=1 SV=1	TPD53_HUMAN	22 kDa	3	15%
1247	Triosephosphate isomerase OS=Homo sapiens GN=TPI1 PE=1 SV=2	TPIS_HUMAN	27 kDa	17	84%
1248	Tropomyosin alpha-1 chain OS=Homo sapiens GN=TPM1 PE=1 SV=2	TPM1_HUMAN	33 kDa	3	18%
1249	Tropomyosin alpha-3 chain OS=Homo sapiens GN=TPM3 PE=1 SV=1	TPM3_HUMAN	33 kDa	4	25%
1250	Tropomyosin alpha-4 chain OS=Homo sapiens GN=TPM4 PE=1 SV=3	TPM4_HUMAN	29 kDa	18	49%
1251	Tripeptidyl-peptidase 1 OS=Homo sapiens GN=TPP1 PE=1 SV=2	TPP1_HUMAN	61 kDa	4	15%
1252	Tumor necrosis factor receptor superfamily member 11B OS=Homo sapiens GN=TNFRSF11B PE=1 SV=3	TR11B_HUMAN	46 kDa	18	51%
1253	Serotransferrin OS=Homo sapiens GN=TF PE=1 SV=3	TRFE_HUMAN	77 kDa	47	62%
1254	Lactotransferrin OS=Homo sapiens GN=LTF PE=1 SV=6	TRFL_HUMAN	78 kDa	84	88%
1255	Thioredoxin reductase 1, cytoplasmic OS=Homo sapiens GN=TXNRD1 PE=1 SV=3	TRXR1_HUMAN	71 kDa	9	13%
1256	Trypsin-1 OS=Homo sapiens GN=PRSS1 PE=1 SV=1	TRY1_HUMAN	27 kDa	3	15%
1257	Tumor susceptibility gene 101 protein OS=Homo sapiens GN=TSG101 PE=1 SV=2	TS101_HUMAN	44 kDa	2	4.40%
1258	Translin OS=Homo sapiens GN=TSN PE=1 SV=1	TSN_HUMAN	26 kDa	7	30%
1259	Tetraspanin-6 OS=Homo sapiens GN=TSPAN6 PE=1 SV=1	TSN6_HUMAN	28 kDa	2	10%
1260	Tetraspanin-9 OS=Homo sapiens GN=TSPAN9 PE=1 SV=1	TSN9_HUMAN	27 kDa	3	15%
1261	Translin-associated protein X OS=Homo sapiens GN=TSNAX PE=1 SV=1	TSNAX_HUMAN	33 kDa	3	20%
1262	Thrombospondin-1 OS=Homo sapiens GN=THBS1 PE=1 SV=2	TSP1_HUMAN	129 kDa	36	35%
1263	Thrombospondin-3 OS=Homo sapiens GN=THBS3 PE=1 SV=1	TSP3_HUMAN	104 kDa	3	3.20%
1264	Thrombospondin-4 OS=Homo sapiens GN=THBS4 PE=1 SV=2	TSP4_HUMAN	106 kDa	3	4.30%
1265	Thiosulfate sulfurtransferase/rhodanese-like domain-containing protein 1 OS=Homo sapiens GN=TSTD1 PE=1 SV=3	TSTD1_HUMAN	13 kDa	2	19%
1266	Transthyretin OS=Homo sapiens GN=TTR PE=1 SV=1	TTHY_HUMAN	16 kDa	11	73%
1267	Protein tweety homolog 3 OS=Homo sapiens GN=TTYH3 PE=1 SV=3	TTYH3_HUMAN	58 kDa	2	4.20%

#	Proteins	Uniprot ID	Molecular Weight	Number of unique peptides	Percent of coverage
1268	Terminal uridylyltransferase 4 OS=Homo sapiens GN=ZCCHC11 PE=1 SV=3	TUT4_HUMAN	185 kDa	3	2.70%
1269	Twinfilin-1 OS=Homo sapiens GN=TWF1 PE=1 SV=3	TWF1_HUMAN	40 kDa	6	23%
1270	Twinfilin-2 OS=Homo sapiens GN=TWF2 PE=1 SV=2	TWF2_HUMAN	40 kDa	10	38%
1271	Thioredoxin domain-containing protein 12 OS=Homo sapiens GN=TXNDC12 PE=1 SV=1	TXD12_HUMAN	19 kDa	2	14%
1272	Thioredoxin domain-containing protein 16 OS=Homo sapiens GN=TXNDC16 PE=2 SV=4	TXD16_HUMAN	94 kDa	4	5.70%
1273	Thioredoxin domain-containing protein 17 OS=Homo sapiens GN=TXNDC17 PE=1 SV=1	TXD17_HUMAN	14 kDa	5	45%
1274	Thioredoxin domain-containing protein 5 OS=Homo sapiens GN=TXNDC5 PE=1 SV=2	TXND5_HUMAN	48 kDa	6	22%
1275	Thioredoxin-like protein 1 OS=Homo sapiens GN=TXNL1 PE=1 SV=3	TXNL1_HUMAN	32 kDa	4	24%
1276	Thymosin beta-10 OS=Homo sapiens GN=TMSB10 PE=1 SV=2	TYB10_HUMAN	5 kDa	2	50%
1277	Thymidine phosphorylase OS=Homo sapiens GN=TYMP PE=1 SV=2	TYPH_HUMAN	50 kDa	3	9.30%
1278	UDP-N-acetylhexosamine pyrophosphorylase OS=Homo sapiens GN=UAP1 PE=1 SV=3	UAP1_HUMAN	59 kDa	4	9.40%
1279	Ubiquitin-conjugating enzyme E2 D2 OS=Homo sapiens GN=UBE2D2 PE=1 SV=1	UB2D2_HUMAN (+1)	17 kDa	2	20%
1280	Ubiquitin-conjugating enzyme E2 L3 OS=Homo sapiens GN=UBE2L3 PE=1 SV=1	UB2L3_HUMAN	18 kDa	6	42%
1281	Ubiquitin-conjugating enzyme E2 variant 1 OS=Homo sapiens GN=UBE2V1 PE=1 SV=2	UB2V1_HUMAN	16 kDa	5	34%
1282	Ubiquitin-like modifier-activating enzyme 1 OS=Homo sapiens GN=UBA1 PE=1 SV=3	UBA1_HUMAN	118 kDa	26	33%
1283	NEDD8-activating enzyme E1 catalytic subunit OS=Homo sapiens GN=UBA3 PE=1 SV=2	UBA3_HUMAN	52 kDa	2	8.00%
1284	Ubiquitin-like modifier-activating enzyme 5 OS=Homo sapiens GN=UBA5 PE=1 SV=1	UBA5_HUMAN	45 kDa	5	22%
1285	Ubiquitin-conjugating enzyme E2 K OS=Homo sapiens GN=UBE2K PE=1 SV=3	UBE2K_HUMAN	22 kDa	2	13%
1286	Ubiquitin-conjugating enzyme E2 N OS=Homo sapiens GN=UBE2N PE=1 SV=1	UBE2N_HUMAN	17 kDa	8	53%
1287	Ubiquitin carboxyl-terminal hydrolase 14 OS=Homo sapiens GN=USP14 PE=1 SV=3	UBP14_HUMAN	56 kDa	5	14%
1288	Ubiquitin carboxyl-terminal hydrolase 15 OS=Homo sapiens GN=USP15 PE=1 SV=3	UBP15_HUMAN	112 kDa	3	3.00%
1289	Ubiquitin carboxyl-terminal hydrolase 47 OS=Homo sapiens GN=USP47 PE=1 SV=3	UBP47_HUMAN	157 kDa	4	2.60%
1290	Ubiquitin carboxyl-terminal hydrolase 5 OS=Homo sapiens GN=USP5 PE=1 SV=2	UBP5_HUMAN	96 kDa	7	11%
1291	Ubiquilin-1 OS=Homo sapiens GN=UBQLN1 PE=1 SV=2	UBQL1_HUMAN	63 kDa	3	10%
1292	E3 ubiquitin-protein ligase UBR4 OS=Homo sapiens GN=UBR4 PE=1 SV=1	UBR4_HUMAN	574 kDa	5	1.10%
1293	Ubiquitin carboxyl-terminal hydrolase isozyme L3 OS=Homo sapiens GN=UCHL3 PE=1 SV=1	UCHL3_HUMAN	26 kDa	5	27%
1294	Ubiquitin-fold modifier-conjugating enzyme 1 OS=Homo sapiens GN=UFC1 PE=1 SV=3	UFC1_HUMAN	19 kDa	2	10%
1295	Ubiquitin-fold modifier 1 OS=Homo sapiens GN=UFM1 PE=1 SV=1	UFM1_HUMAN	9 kDa	4	68%
1296	UDP-glucose:glycoprotein glucosyltransferase 1 OS=Homo sapiens GN=UGGT1 PE=1 SV=3	UGGG1_HUMAN	177 kDa	5	4.10%
1297	UTP--glucose-1-phosphate uridylyltransferase OS=Homo sapiens GN=UGP2 PE=1 SV=5	UGPA_HUMAN	57 kDa	40	72%
1298	Urokinase plasminogen activator surface receptor OS=Homo sapiens GN=PLAUR PE=1 SV=1	UPAR_HUMAN	37 kDa	3	17%
1299	General vesicular transport factor p115 OS=Homo sapiens GN=USO1 PE=1 SV=2	USO1_HUMAN	108 kDa	8	9.90%
1300	Vesicle-associated membrane protein-associated protein A OS=Homo sapiens GN=VAPA PE=1 SV=3	VAPA_HUMAN	28 kDa	5	23%
1301	V-type proton ATPase subunit S1 OS=Homo sapiens GN=ATP6AP1 PE=1 SV=2	VAS1_HUMAN	52 kDa	7	21%
1302	Vasorin OS=Homo sapiens GN=VASN PE=1 SV=1	VASN_HUMAN	72 kDa	2	4.50%
1303	Vasodilator-stimulated phosphoprotein OS=Homo sapiens GN=VASP PE=1 SV=3	VASP_HUMAN	40 kDa	2	6.80%
1304	Synaptic vesicle membrane protein VAT-1 homolog OS=Homo sapiens GN=VAT1 PE=1 SV=2	VAT1_HUMAN	42 kDa	7	26%

#	Proteins	Uniprot ID	Molecular Weight	Number of unique peptides	Percent of coverage
1305	V-type proton ATPase catalytic subunit A OS=Homo sapiens GN=ATP6V1A PE=1 SV=2	VATA_HUMAN	68 kDa	3	5.50%
1306	V-type proton ATPase subunit B, brain isoform OS=Homo sapiens GN=ATP6V1B2 PE=1 SV=3	VATB2_HUMAN	57 kDa	4	14%
1307	Vascular cell adhesion protein 1 OS=Homo sapiens GN=VCAM1 PE=1 SV=1	VCAM1_HUMAN	81 kDa	4	6.60%
1308	Vimentin OS=Homo sapiens GN=VIM PE=1 SV=4	VIME_HUMAN	54 kDa	2	4.90%
1309	Vinculin OS=Homo sapiens GN=VCL PE=1 SV=4	VINC_HUMAN	124 kDa	11	12%
1310	Vacuolar protein sorting-associated protein 29 OS=Homo sapiens GN=VPS29 PE=1 SV=1	VPS29_HUMAN	21 kDa	3	18%
1311	Vacuolar protein sorting-associated protein 35 OS=Homo sapiens GN=VPS35 PE=1 SV=2	VPS35_HUMAN	92 kDa	7	10%
1312	Vacuolar protein sorting-associated protein 4B OS=Homo sapiens GN=VPS4B PE=1 SV=2	VPS4B_HUMAN	49 kDa	3	7.90%
1313	Putative V-set and immunoglobulin domain-containing protein 6 OS=Homo sapiens GN=VSIG6 PE=5 SV=2	VSIG6_HUMAN	14 kDa	3	18%
1314	V-set domain-containing T-cell activation inhibitor 1 OS=Homo sapiens GN=VTCN1 PE=1 SV=1	VTCN1_HUMAN	31 kDa	4	14%
1315	Vitamin D-binding protein OS=Homo sapiens GN=GC PE=1 SV=1	VTDB_HUMAN	53 kDa	30	69%
1316	Vitronectin OS=Homo sapiens GN=VTN PE=1 SV=1	VTNC_HUMAN	54 kDa	10	32%
1317	von Willebrand factor A domain-containing protein 1 OS=Homo sapiens GN=VWA1 PE=2 SV=1	VWA1_HUMAN	47 kDa	14	56%
1318	WW domain-binding protein 2 OS=Homo sapiens GN=WBP2 PE=1 SV=1	WBP2_HUMAN	28 kDa	4	17%
1319	WD repeat-containing protein 1 OS=Homo sapiens GN=WDR1 PE=1 SV=4	WDR1_HUMAN	66 kDa	20	40%
1320	WD repeat-containing protein 65 OS=Homo sapiens GN=WDR65 PE=2 SV=3	WDR65_HUMAN	145 kDa	2	1.40%
1321	WAP four-disulfide core domain protein 2 OS=Homo sapiens GN=WFDC2 PE=1 SV=2	WFDC2_HUMAN	13 kDa	4	45%
1322	Xanthine dehydrogenase/oxidase OS=Homo sapiens GN=XDH PE=1 SV=4	XDH_HUMAN	146 kDa	85	75%
1323	Exportin-1 OS=Homo sapiens GN=XPO1 PE=1 SV=1	XPO1_HUMAN	123 kDa	2	1.70%
1324	Exportin-T OS=Homo sapiens GN=XPOT PE=1 SV=2	XPOT_HUMAN	110 kDa	2	2.20%
1325	Xaa-Pro aminopeptidase 1 OS=Homo sapiens GN=XPNPEP1 PE=1 SV=3	XPP1_HUMAN	70 kDa	10	19%
1326	Protein XRP2 OS=Homo sapiens GN=RP2 PE=1 SV=4	XRP2_HUMAN	40 kDa	3	5.70%
1327	Xylosyltransferase 1 OS=Homo sapiens GN=XYLT1 PE=1 SV=1	XYLT1_HUMAN	108 kDa	3	3.00%
1328	Nuclease-sensitive element-binding protein 1 OS=Homo sapiens GN=YBX1 PE=1 SV=3	YBOX1_HUMAN	36 kDa	4	20%
1329	Synaptobrevin homolog YKT6 OS=Homo sapiens GN=YKT6 PE=1 SV=1	YKT6_HUMAN	22 kDa	9	52%
1330	Zinc finger protein 804B OS=Homo sapiens GN=ZNF804B PE=1 SV=2	Z804B_HUMAN	153 kDa	2	1.10%
1331	Zinc-alpha-2-glycoprotein OS=Homo sapiens GN=AZGP1 PE=1 SV=2	ZA2G_HUMAN	34 kDa	23	61%
1332	Zymogen granule protein 16 homolog B OS=Homo sapiens GN=ZG16B PE=1 SV=3	ZG16B_HUMAN	23 kDa	7	49%
1333	Protein Z-dependent protease inhibitor OS=Homo sapiens GN=SERPINA10 PE=1 SV=1	ZPI_HUMAN	51 kDa	2	5.90%
