# Supplementary material for: Quantitative Analysis of the Human Milk Whey Proteome Reveals Developing Milk and Mammary-Gland Functions across the First Year of Lactation
Source: Proteomes. 2013 Sep 3;1(2):128–58. doi: 10.3390/proteomes1020128 (PMC5302745; doi:10.3390/proteomes1020128)
Supplement: Supplementary File 1 [file proteomes-01-00128-s001.zip › Supplementary dataset 2.pdf]

Supplementary Dataset 2. List of 615 quantified human milk whey proteins during the first 12 months of lactation.

No.	Uniprot ID	Gene symbol	*p*-value (Subject) [a)]	*p*-value (Time) [b)]	*p*-value (1 mo *vs.* 0 mo) [c)]	Ratio (1 mo *vs.* 0 mo) [d)]	*p*-value (3 mo *vs.* 0 mo) [c)]	Ratio (3 mo *vs.* 0 mo) [d)]	*p*-value (6 mo *vs.* 0 mo) [c)]	Ratio (6 mo *vs.* 0 mo) [d)]	*p*-value (9 mo *vs.* 0 mo) [c)]	Ratio (9 mo *vs.* 0 mo) [d)]	*p*-value (12 mo *vs.* 0 mo) [c)]	Ratio (12 mo *vs.* 0 mo) [d)]
1	1433B_HUMAN	YWHAB	6.6E-10	2.0E-08	7.0E-03	1.4	1.2E-05	1.8	4.8E-08	2.2	4.4E-08	2.2	3.5E-07	2.0
2	1433E_HUMAN	YWHAE	4.1E-10	2.9E-08	2.6E-02	1.3	9.2E-05	1.7	2.3E-07	2.1	4.2E-08	2.2	7.3E-07	2.0
3	1433F_HUMAN	YWHAH	1.8E-10	1.4E-09	3.6E-03	1.5	2.4E-06	2.0	8.6E-09	2.3	1.8E-09	2.4	2.0E-07	2.1
4	1433G_HUMAN	YWHAG	1.2E-10	4.1E-08	3.8E-03	1.5	1.8E-05	1.8	5.6E-08	2.2	4.6E-08	2.2	6.5E-07	2.0
5	1433S_HUMAN	SFN	1.9E-06	5.2E-09	7.1E-04	1.6	1.1E-05	1.9	2.0E-08	2.3	3.3E-09	2.5	1.1E-07	2.2
6	1433T_HUMAN	YWHAQ	1.0E-10	1.2E-08	6.2E-03	1.4	6.0E-06	1.9	9.0E-09	2.3	1.4E-07	2.1	3.7E-07	2.0
7	1433Z_HUMAN	YWHAZ	1.9E-11	5.6E-08	2.0E-03	1.5	1.7E-05	1.8	5.3E-08	2.1	4.6E-08	2.2	7.8E-07	2.0
8	2AAA_HUMAN	PPP2R1A	7.1E-02	1.4E-01	6.1E-02	1.2	1.4E-02	1.3	3.9E-02	1.2	2.2E-02	1.3	6.6E-02	1.2
9	6PGD_HUMAN	PGD	1.0E-09	3.2E-13	5.5E-06	1.9	1.2E-08	2.4	2.8E-11	2.8	6.4E-13	3.1	1.0E-11	2.9
10	6PGL_HUMAN	PGLS	3.0E-03	6.2E-02	1.4E-01	1.2	2.2E-01	1.1	4.4E-03	1.4	3.7E-02	1.3	1.7E-02	1.3
11	A1AG1_HUMAN	ORM1	6.0E-10	1.6E-24	7.0E-03	0.8	7.3E-15	0.6	1.1E-14	0.6	1.2E-18	0.5	5.8E-21	0.5
12	A1AG2_HUMAN	ORM2	4.2E-08	4.8E-06	5.5E-02	0.9	1.6E-05	0.7	4.5E-05	0.7	6.5E-06	0.7	2.4E-05	0.7
13	A1AT_HUMAN	SERPINA1	1.7E-15	2.5E-19	1.7E-07	0.7	6.3E-15	0.6	2.4E-17	0.6	1.3E-14	0.6	4.7E-17	0.6
14	A1BG_HUMAN	A1BG	1.1E-12	1.0E-15	4.8E-03	0.8	9.4E-10	0.6	4.0E-13	0.5	1.1E-12	0.5	3.7E-12	0.5
15	A2AP_HUMAN	SERPINF2	1.7E-10	7.4E-04	7.6E-04	1.2	6.3E-04	1.2	1.1E-03	1.2	3.0E-05	1.3	1.1E-02	1.2
16	A2GL_HUMAN	LRG1	1.5E-17	2.7E-33	1.3E-18	0.5	2.3E-27	0.4	4.4E-31	0.3	4.7E-28	0.4	5.9E-27	0.4
17	A2MG_HUMAN	A2M	9.7E-20	1.2E-18	5.0E-01	1.0	3.5E-05	0.8	7.8E-08	0.7	8.6E-12	0.7	2.2E-16	0.6
18	A4_HUMAN	APP	5.5E-12	2.8E-21	1.1E-03	0.7	3.1E-07	0.6	1.2E-15	0.4	7.1E-18	0.3	1.5E-17	0.3
19	AACT_HUMAN	SERPINA3	3.9E-14	3.6E-06	1.0E-04	0.5	6.7E-05	0.4	4.1E-07	0.3	1.4E-03	0.5	1.1E-01	0.7
20	AATC_HUMAN	GOT1	1.5E-06	5.8E-07	3.3E-02	1.3	9.4E-04	1.5	3.6E-07	2.0	3.2E-05	1.7	5.8E-07	1.9
21	ABCG2_HUMAN	ABCG2	1.8E-09	1.9E-06	3.2E-01	1.2	7.3E-03	1.5	3.5E-07	2.2	1.2E-04	1.8	1.9E-04	1.8
22	ABHEB_HUMAN	ABHD14B	3.2E-06	1.8E-09	2.6E-02	1.4	2.0E-05	1.9	9.9E-08	2.2	6.6E-10	2.6	2.1E-06	2.0
23	ACBP_HUMAN	DBI	6.4E-07	3.6E-06	1.2E-03	1.4	6.9E-01	1.0	7.5E-01	1.0	2.0E-02	0.8	6.7E-02	0.8
24	ACE_HUMAN	ACE	3.2E-15	1.6E-11	3.8E-03	0.8	1.1E-07	0.7	3.4E-07	0.7	1.6E-10	0.6	4.2E-11	0.6
25	ACLY_HUMAN	ACLY	5.7E-11	2.8E-04	6.1E-03	1.7	2.2E-03	1.8	1.2E-04	2.1	4.4E-05	2.3	7.3E-05	2.2
26	ACOC_HUMAN	ACO1	3.9E-10	5.1E-07	3.4E-07	2.2	3.9E-07	2.1	3.0E-05	1.8	2.7E-03	1.5	2.4E-02	1.4
27	ACSA_HUMAN	ACSS2	7.2E-04	2.0E-01	1.3E-01	1.3	2.8E-02	1.5	4.5E-02	1.4	2.9E-02	1.4	1.1E-01	1.3
28	ACTB_HUMAN	ACTB	2.2E-06	1.9E-04	9.2E-03	1.3	2.4E-03	1.4	1.5E-04	1.5	3.4E-05	1.6	9.6E-05	1.6
29	ACTG_HUMAN	ACTG1	2.0E-05	8.2E-04	2.4E-04	1.7	5.9E-04	1.6	2.1E-04	1.7	6.8E-02	1.3	2.0E-02	1.4
30	ACTN1_HUMAN	ACTN1	1.0E-10	9.5E-03	2.5E-01	1.2	2.0E-01	1.2	3.7E-03	1.5	7.8E-04	1.6	8.3E-02	1.2
31	ACTN4_HUMAN	ACTN4	5.5E-11	4.4E-03	2.0E-01	1.2	8.4E-02	1.3	2.0E-03	1.5	2.6E-04	1.6	5.1E-02	1.3
32	ADA10_HUMAN	ADAM10	1.6E-15	3.0E-09	2.9E-02	1.2	1.1E-04	1.4	1.3E-05	1.4	1.3E-06	1.5	1.4E-10	1.7
33	ADA15_HUMAN	ADAM15	1.0E-01	2.2E-01	1.6E-01	0.9	8.8E-01	1.0	3.5E-01	1.0	8.5E-01	1.0	2.6E-01	1.0
34	ADEC1_HUMAN	ADAMDEC1	3.8E-01	5.1E-03	6.2E-03	1.1	5.6E-04	1.1	6.8E-03	1.1	2.2E-03	1.1	2.2E-03	1.1
35	AFAM_HUMAN	AFM	2.6E-07	4.2E-13	2.0E-03	0.8	3.9E-06	0.7	1.8E-09	0.6	4.3E-09	0.6	7.0E-14	0.5
36	AGM1_HUMAN	PGM3	1.1E-02	3.3E-01	8.5E-02	1.2	4.5E-02	1.3	8.4E-02	1.2	1.3E-01	1.2	3.6E-01	1.1

No.	Uniprot ID	Gene symbol	*p*-value (Subject) a)	*p*-value (Time) b)	*p*-value (1 mo *vs.* 0 mo) c)	Ratio (1 mo *vs.* 0 mo) d)	*p*-value (3 mo *vs.* 0 mo) c)	Ratio (3 mo *vs.* 0 mo) d)	*p*-value (6 mo *vs.* 0 mo) c)	Ratio (6 mo *vs.* 0 mo) d)	*p*-value (9 mo *vs.* 0 mo) c)	Ratio (9 mo *vs.* 0 mo) d)	*p*-value (12 mo *vs.* 0 mo) c)	Ratio (12 mo *vs.* 0 mo) d)
37	AK1A1_HUMAN	AKR1A1	7.3E-12	5.5E-12	2.2E-06	2.0	1.2E-09	2.5	6.5E-12	3.0	1.1E-10	2.7	7.7E-10	2.6
38	AK1C3_HUMAN	AKR1C3	2.3E-06	1.2E-05	1.6E-03	1.8	2.1E-04	2.0	1.7E-05	2.3	1.4E-05	2.3	4.4E-06	2.5
39	AL1A1_HUMAN	ALDH1A1	1.5E-07	1.3E-21	1.9E-08	2.6	8.5E-16	4.4	3.2E-19	5.7	1.6E-19	5.8	2.3E-16	4.6
40	AL1A3_HUMAN	ALDH1A3	1.5E-10	5.7E-12	2.5E-06	2.1	2.3E-10	2.9	1.2E-11	3.1	1.6E-10	2.9	2.4E-09	2.7
41	AL1L1_HUMAN		2.1E-10	1.1E-17	1.3E-06	2.1	2.5E-12	3.3	1.2E-15	4.1	6.4E-16	4.1	2.8E-14	3.7
42	AL7A1_HUMAN	ALDH7A1	5.5E-10	1.6E-05	1.0E-02	1.3	1.1E-04	1.5	1.9E-06	1.7	1.3E-05	1.6	1.5E-04	1.5
43	AL9A1_HUMAN	ALDH9A1	3.7E-11	4.1E-09	4.9E-05	1.8	1.8E-07	2.2	8.0E-10	2.7	6.1E-08	2.3	9.0E-08	2.3
44	ALAT1_HUMAN	GPT	2.3E-07	3.6E-07	1.8E-03	1.7	7.3E-07	2.5	5.9E-08	2.8	9.7E-06	2.2	2.6E-03	1.7
45	ALBU_HUMAN	ALB	9.4E-15	1.1E-27	5.9E-20	1.6	1.3E-26	1.7	4.6E-25	1.7	2.1E-20	1.6	8.6E-18	1.5
46	ALDOA_HUMAN	ALDOA	5.7E-09	2.3E-12	1.1E-04	1.7	4.0E-08	2.2	3.3E-11	2.7	5.6E-12	2.9	6.8E-10	2.5
47	ALDOC_HUMAN	ALDOC	1.7E-04	1.8E-10	8.9E-04	1.7	2.3E-07	2.2	3.9E-09	2.5	2.4E-10	2.8	1.3E-08	2.5
48	AMBP_HUMAN	AMBP	4.0E-02	3.5E-01	1.8E-01	1.1	8.6E-01	1.0	5.3E-01	1.0	9.7E-01	1.0	2.6E-01	0.9
49	AMPB_HUMAN	RNPEP	2.7E-04	1.5E-10	3.0E-04	1.6	2.2E-07	2.1	4.0E-09	2.3	2.6E-09	2.4	2.8E-10	2.5
50	AMPL_HUMAN	LAP3	7.2E-10	2.3E-24	3.1E-05	1.9	1.4E-14	3.7	5.7E-19	4.9	1.2E-20	5.5	6.6E-20	5.2
51	AMRP_HUMAN	LRPAP1	1.2E-04	1.4E-04	2.1E-01	1.1	2.3E-04	1.5	4.1E-04	1.5	2.4E-04	1.5	1.2E-04	1.6
52	AMY1_HUMAN	AMY1B AMY1C AMY1A	1.7E-06	4.3E-22	4.7E-01	1.0	5.9E-06	0.8	4.9E-10	0.7	6.7E-15	0.7	4.5E-15	0.7
53	ANAG_HUMAN	NAGLU	1.4E-03	1.9E-05	5.5E-04	1.3	8.1E-07	1.5	7.4E-05	1.4	1.3E-03	1.3	4.7E-02	1.2
54	ANGI_HUMAN	ANG	7.0E-01	2.3E-04	1.2E-04	1.3	4.8E-05	1.3	1.1E-03	1.2	6.7E-04	1.2	5.1E-02	1.1
55	ANGL4_HUMAN	ANGPTL4	1.9E-07	7.2E-11	1.6E-03	0.6	5.8E-06	0.5	1.1E-08	0.4	1.5E-11	0.3	7.7E-09	0.3
56	ANGT_HUMAN	AGT	3.7E-10	2.8E-28	6.2E-14	0.6	4.1E-19	0.5	5.1E-21	0.5	7.7E-24	0.5	4.3E-28	0.4
57	ANT3_HUMAN	SERPINC1	3.4E-11	1.6E-01	1.6E-02	1.1	4.8E-02	1.1	3.7E-02	1.1	2.6E-02	1.1	8.3E-02	1.1
58	AP1B1_HUMAN	AP1B1	2.0E-02	3.1E-02	6.6E-01	1.0	1.2E-01	1.1	2.5E-02	1.2	5.7E-02	1.2	3.3E-02	1.2
59	APOA1_HUMAN	APOA1	1.2E-11	1.3E-08	1.6E-01	1.1	5.9E-01	1.0	8.8E-02	0.9	1.4E-03	0.8	1.5E-06	0.7
60	APOA2_HUMAN	APOA2	4.9E-10	8.5E-06	5.4E-06	1.5	3.0E-04	1.4	2.7E-04	1.4	1.7E-03	1.3	4.7E-01	1.1
61	APOA4_HUMAN	APOA4	1.6E-16	8.8E-24	2.2E-04	1.2	6.3E-01	1.0	4.0E-03	0.9	9.4E-11	0.7	2.2E-12	0.7
62	APOB_HUMAN	APOB	1.4E-11	1.3E-13	5.9E-04	0.7	8.9E-08	0.6	6.3E-08	0.5	7.4E-12	0.5	7.6E-14	0.4
63	APOD_HUMAN	APOD	1.4E-11	1.1E-03	1.6E-01	0.9	3.7E-04	0.8	1.9E-03	0.8	2.7E-01	0.9	6.7E-01	1.0
64	APOE_HUMAN	APOE	1.8E-12	1.9E-10	9.7E-01	1.0	2.9E-04	0.8	1.0E-04	0.8	1.1E-06	0.7	6.4E-09	0.7
65	APOH_HUMAN	APOH	5.9E-08	3.5E-11	8.3E-01	1.0	1.1E-03	0.7	1.9E-06	0.6	1.3E-09	0.5	3.1E-07	0.6
66	APT_HUMAN	APRT	3.1E-06	2.9E-03	1.6E-02	1.5	3.2E-03	1.6	3.3E-04	1.8	2.8E-03	1.6	2.0E-03	1.7
67	ARC1B_HUMAN	ARPC1B	3.5E-03	5.3E-04	2.6E-01	1.2	4.6E-03	1.7	6.8E-03	1.6	2.8E-05	2.2	1.4E-03	1.8
68	ARF1_HUMAN	ARF1	1.1E-08	5.8E-12	8.6E-07	1.6	5.0E-09	1.8	6.6E-14	2.3	2.1E-08	1.8	6.9E-06	1.6
69	ARF4_HUMAN	ARF4	1.2E-11	1.0E-08	3.4E-04	1.6	6.5E-07	2.0	1.1E-10	2.6	1.3E-05	1.8	6.8E-05	1.7
70	ARK72_HUMAN	AKR7A2	6.2E-10	4.2E-10	5.1E-07	2.2	7.9E-09	2.6	5.6E-10	2.8	1.6E-08	2.5	7.5E-09	2.6
71	ARLY_HUMAN	ASL	5.3E-03	2.5E-06	1.9E-02	1.4	5.3E-05	1.9	1.6E-06	2.3	2.1E-06	2.2	2.7E-05	2.0

No.	Uniprot ID	Gene symbol	*p*-value (Subject) [a)]	*p*-value (Time) [b)]	*p*-value (1 mo *vs.* 0 mo) [c)]	Ratio (1 mo *vs.* 0 mo) [d)]	*p*-value (3 mo *vs.* 0 mo) [c)]	Ratio (3 mo *vs.* 0 mo) [d)]	*p*-value (6 mo *vs.* 0 mo) [c)]	Ratio (6 mo *vs.* 0 mo) [d)]	*p*-value (9 mo *vs.* 0 mo) [c)]	Ratio (9 mo *vs.* 0 mo) [d)]	*p*-value (12 mo *vs.* 0 mo) [c)]	Ratio (12 mo *vs.* 0 mo) [d)]
72	ARP2_HUMAN	ACTR2	9.5E-05	6.8E-04	7.5E-03	1.6	2.0E-04	1.9	1.6E-04	1.9	4.8E-04	1.8	1.9E-03	1.7
73	ARP3_HUMAN	ACTR3	1.1E-02	1.7E-01	1.1E-01	1.2	3.9E-02	1.3	2.9E-02	1.3	1.6E-02	1.3	8.6E-02	1.2
74	ARPC2_HUMAN	ARPC2	3.7E-02	9.0E-02	6.3E-01	1.0	9.0E-02	1.2	3.7E-02	1.2	9.5E-03	1.3	1.3E-01	1.2
75	ARPC3_HUMAN	ARPC3	8.7E-01	4.0E-03	4.2E-04	1.3	1.1E-02	1.2	1.3E-02	1.2	7.0E-04	1.3	6.2E-03	1.3
76	ARPC4_HUMAN	ARPC4 TTLL3	8.6E-06	9.5E-04	1.2E-01	1.3	1.3E-03	1.7	1.2E-03	1.7	7.9E-05	2.0	2.3E-02	1.5
77	ARSA_HUMAN	ARSA	8.1E-01	7.7E-06	9.1E-03	1.2	4.7E-05	1.3	1.1E-06	1.4	1.3E-05	1.3	2.8E-04	1.3
78	ASM3B_HUMAN	SMPDL3B	3.8E-16	1.6E-16	1.2E-02	0.8	1.4E-11	0.6	1.2E-08	0.7	6.1E-14	0.6	9.1E-14	0.6
79	ATRN_HUMAN	ATRN	2.0E-01	9.3E-03	9.1E-01	1.0	5.6E-02	0.9	1.5E-02	0.9	9.5E-03	0.9	5.3E-03	0.9
80	B2MG_HUMAN	B2M	6.4E-09	3.8E-02	4.8E-01	1.0	5.8E-01	1.0	8.0E-03	0.9	2.7E-02	0.9	7.6E-01	1.0
81	B3GN7_HUMAN	B3GNT7	4.2E-04	8.2E-26	3.4E-03	0.7	6.0E-14	0.4	3.9E-18	0.3	1.4E-22	0.2	1.1E-18	0.3
82	B4GT1_HUMAN	B4GALT1	4.3E-13	5.9E-23	2.2E-04	0.8	1.0E-14	0.6	8.2E-17	0.6	2.3E-18	0.6	1.7E-19	0.5
83	B7H6_HUMAN		5.3E-09	8.3E-11	1.4E-07	1.7	6.2E-09	1.9	3.0E-11	2.1	1.3E-08	1.8	1.5E-04	1.5
84	BGAL_HUMAN	GLB1	6.0E-04	3.0E-02	8.0E-02	1.2	3.8E-02	1.2	6.9E-04	1.4	1.1E-01	1.2	1.1E-01	1.2
85	BLVRB_HUMAN	BLVRB	5.4E-03	7.4E-09	1.7E-03	1.6	9.7E-06	1.9	8.8E-09	2.5	7.0E-09	2.5	2.7E-07	2.2
86	BPNT1_HUMAN	BPNT1	1.3E-03	7.4E-03	2.0E-01	1.2	2.8E-02	1.3	3.0E-02	1.3	1.4E-03	1.5	7.9E-04	1.6
87	BT1A1_HUMAN	BTN1A1	6.9E-17	7.5E-22	2.6E-03	1.3	1.6E-07	1.5	2.9E-17	2.1	4.1E-17	2.1	4.1E-17	2.1
88	BTD_HUMAN	BTD	8.0E-06	1.1E-04	1.5E-02	0.8	4.7E-04	0.8	1.2E-05	0.7	4.9E-05	0.7	4.6E-03	0.8
89	C1RL_HUMAN	C1RL	2.8E-05	6.3E-16	2.4E-04	0.8	5.5E-08	0.7	1.5E-14	0.6	3.4E-13	0.6	5.7E-13	0.6
90	C1TC_HUMAN	MTHFD1	8.3E-01	2.8E-01	9.2E-01	1.0	2.1E-01	1.1	1.8E-01	1.1	4.6E-02	1.2	3.9E-01	1.1
91	C4BPA_HUMAN	C4BPA	1.6E-13	3.1E-18	8.5E-07	0.5	4.2E-13	0.3	3.1E-17	0.2	3.9E-15	0.2	1.6E-15	0.2
92	CAB39_HUMAN	CAB39	8.2E-09	4.1E-06	2.6E-02	1.2	1.8E-05	1.5	5.6E-07	1.7	4.2E-04	1.4	3.2E-05	1.5
93	CAB45_HUMAN	SDF4	1.7E-11	5.3E-03	1.4E-01	0.9	2.2E-02	0.8	9.5E-03	0.8	6.3E-03	0.8	1.6E-04	0.7
94	CADH1_HUMAN	CDH1	9.6E-04	5.5E-06	1.4E-01	0.8	4.9E-05	0.5	7.5E-05	0.6	2.5E-06	0.5	1.4E-04	0.6
95	CAH2_HUMAN	CA2	2.0E-15	8.3E-09	8.1E-08	2.1	1.8E-08	2.2	6.3E-06	1.9	1.1E-02	1.4	6.5E-02	1.3
96	CAH6_HUMAN	CA6	6.3E-13	1.8E-34	1.8E-02	0.8	1.1E-07	0.7	2.4E-17	0.5	3.1E-26	0.4	2.7E-29	0.3
97	CALB2_HUMAN	CALB2	3.7E-11	5.1E-28	5.2E-14	3.4	8.4E-21	5.3	2.4E-24	6.7	3.3E-25	7.1	6.3E-24	6.5
98	CALL5_HUMAN	CALML5	3.2E-04	9.8E-20	2.2E-09	2.5	6.0E-15	3.8	9.4E-17	4.3	1.5E-17	4.5	1.1E-16	4.3
99	CALM_HUMAN	CALM1 CALM2 CALM3	3.2E-08	1.3E-08	8.7E-03	1.4	3.1E-05	1.6	8.0E-07	1.8	6.3E-08	1.9	1.1E-08	2.0
100	CALR_HUMAN	CALR	4.6E-10	2.3E-01	6.2E-01	1.0	5.3E-01	1.1	1.5E-01	1.1	9.7E-01	1.0	3.7E-01	0.9
101	CALU_HUMAN	CALU	5.6E-04	3.9E-10	2.0E-02	0.9	6.4E-02	0.9	2.7E-01	1.1	1.1E-03	1.2	1.1E-07	1.5
102	CAND1_HUMAN	CAND1	1.8E-05	4.4E-06	3.4E-03	1.5	1.4E-04	1.8	2.6E-06	2.1	5.0E-06	2.0	6.3E-06	2.0
103	CANT1_HUMAN	CANT1	6.7E-08	3.4E-04	9.3E-04	0.7	5.3E-04	0.7	7.6E-03	0.8	4.8E-03	0.8	8.4E-06	0.6
104	CAP1_HUMAN	CAP1	3.8E-11	1.2E-05	1.2E-03	1.6	1.5E-04	1.7	4.7E-06	1.9	7.6E-06	1.9	6.3E-06	1.9
105	CAPG_HUMAN	CAPG	2.0E-06	6.4E-34	2.4E-04	1.5	7.2E-17	3.1	4.5E-23	4.1	5.5E-28	5.7	4.6E-29	6.1

No.	Uniprot ID	Gene symbol	*p*-value (Subject) [a)]	*p*-value (Time) [b)]	*p*-value (1 mo *vs.* 0 mo) [c)]	Ratio (1 mo *vs.* 0 mo) [d)]	*p*-value (3 mo *vs.* 0 mo) [c)]	Ratio (3 mo *vs.* 0 mo) [d)]	*p*-value (6 mo *vs.* 0 mo) [c)]	Ratio (6 mo *vs.* 0 mo) [d)]	*p*-value (9 mo *vs.* 0 mo) [c)]	Ratio (9 mo *vs.* 0 mo) [d)]	*p*-value (12 mo *vs.* 0 mo) [c)]	Ratio (12 mo *vs.* 0 mo) [d)]
106	CAPZB_HUMAN	CAPZB	8.0E-05	1.3E-04	1.6E-01	1.2	2.2E-03	1.6	1.7E-05	2.0	3.5E-03	1.5	1.7E-04	1.8
107	CASA1_HUMAN	CSN1S1	3.6E-06	7.4E-27	3.1E-18	0.5	3.8E-22	0.4	7.7E-26	0.4	4.7E-23	0.4	2.8E-15	0.5
108	CASB_HUMAN	CSN2	6.9E-12	5.6E-01	8.0E-01	1.0	3.9E-01	1.1	1.4E-01	1.1	7.0E-01	1.0	7.8E-01	1.0
109	CASK_HUMAN	CSN3	1.8E-05	7.9E-21	3.6E-12	0.5	2.7E-16	0.4	1.3E-20	0.3	9.6E-17	0.4	6.4E-08	0.6
110	CATA_HUMAN	CAT	5.6E-01	2.9E-04	9.2E-02	1.2	4.4E-02	1.2	2.8E-04	1.4	2.5E-05	1.5	1.7E-03	1.4
111	CATB_HUMAN	CTSB	4.3E-14	3.8E-02	2.4E-01	0.9	7.5E-01	1.0	4.6E-01	1.1	3.8E-02	1.2	1.2E-01	1.2
112	CATC_HUMAN	CTSC	1.3E-12	1.3E-22	3.3E-05	0.7	3.9E-12	0.5	5.4E-17	0.4	7.5E-20	0.4	7.1E-20	0.4
113	CATD_HUMAN	CTSD	2.5E-18	1.2E-09	7.5E-01	1.0	1.1E-02	1.3	9.3E-06	1.5	7.8E-08	1.7	8.9E-07	1.6
114	CATS_HUMAN	CTSS	1.6E-10	9.6E-27	9.5E-05	1.2	5.3E-15	1.6	3.5E-20	1.9	1.6E-20	1.9	2.6E-23	2.0
115	CATZ_HUMAN	CTSZ	4.3E-06	1.8E-01	8.1E-01	1.0	6.1E-01	1.0	3.2E-01	1.1	1.4E-01	0.9	4.2E-01	0.9
116	CAZA1_HUMAN	CAPZA1	1.0E-06	1.1E-07	6.0E-02	1.3	2.8E-05	1.7	3.8E-05	1.7	4.0E-08	2.1	7.4E-07	1.9
117	CAZA2_HUMAN	CAPZA2	2.6E-04	1.6E-06	7.7E-02	1.3	1.7E-04	1.7	2.5E-05	1.8	1.1E-06	2.0	3.7E-06	1.9
118	CBG_HUMAN	SERPINA6	1.7E-08	3.4E-05	4.1E-05	0.8	4.2E-04	0.8	1.1E-04	0.8	4.5E-03	0.8	2.1E-06	0.7
119	CBR1_HUMAN	CBR1	9.9E-05	4.3E-03	2.4E-02	1.5	6.1E-04	2.0	4.2E-03	1.7	2.0E-03	1.9	2.7E-01	1.2
120	CD14_HUMAN	CD14	5.7E-11	1.0E-02	1.8E-01	0.9	1.9E-01	0.9	8.8E-01	1.0	8.9E-01	1.0	3.7E-02	1.1
121	CD36_HUMAN	CD36	1.6E-21	8.3E-10	9.2E-05	0.7	1.2E-10	0.6	2.9E-08	0.6	2.2E-08	0.6	1.1E-04	0.7
122	CD59_HUMAN	CD59	1.2E-07	5.7E-01	3.9E-01	0.9	7.4E-02	0.9	3.5E-01	0.9	6.3E-01	1.0	7.1E-01	1.0
123	CD81_HUMAN	CD81	3.6E-06	7.5E-05	1.8E-01	1.1	9.9E-03	0.8	8.3E-03	0.8	2.1E-03	0.8	2.4E-01	0.9
124	CD9_HUMAN	CD9	4.1E-10	1.9E-08	2.3E-03	0.8	2.0E-07	0.7	2.2E-04	0.8	4.4E-02	0.9	5.2E-01	1.0
125	CDC42_HUMAN	CDC42P2<br>CDC42	6.1E-09	2.4E-02	1.1E-01	1.1	2.5E-02	1.2	9.7E-04	1.3	3.1E-02	1.2	7.7E-03	1.2
126	CEL_HUMAN	CEL	6.8E-08	6.8E-14	4.5E-02	1.1	6.2E-01	1.0	6.7E-02	0.9	6.8E-06	0.8	3.5E-09	0.7
127	CERU_HUMAN	CP	9.6E-10	2.2E-31	1.2E-04	0.8	5.5E-21	0.6	1.1E-20	0.6	2.7E-25	0.5	3.3E-26	0.5
128	CF015_HUMAN	C6ORF15	3.7E-09	4.2E-06	3.8E-01	1.1	2.9E-05	1.9	5.6E-06	2.0	4.3E-03	1.5	8.0E-05	1.8
129	CFAB_HUMAN	CFB	2.9E-07	1.6E-09	7.2E-03	0.8	1.8E-04	0.7	5.1E-10	0.6	3.3E-08	0.6	6.2E-07	0.7
130	CFAH_HUMAN	CFH	5.3E-06	5.0E-14	1.8E-01	1.1	1.4E-01	0.9	2.4E-04	0.8	1.1E-08	0.7	6.6E-09	0.7
131	CFAI_HUMAN	CFI	1.5E-11	5.6E-14	5.6E-02	0.9	9.8E-05	0.7	2.2E-09	0.6	1.6E-12	0.5	1.8E-10	0.6
132	CH3L1_HUMAN	CHI3L1	9.2E-17	3.0E-18	3.1E-08	0.4	3.5E-14	0.2	5.5E-18	0.2	4.8E-15	0.2	3.6E-13	0.3
133	CH3L2_HUMAN	CHI3L2	7.7E-02	5.2E-03	5.8E-02	0.8	6.1E-03	0.8	9.3E-04	0.7	3.4E-03	0.7	1.0E-03	0.7
134	CIB1_HUMAN	CIB1	8.6E-01	3.4E-02	6.0E-03	0.9	5.4E-03	0.9	1.2E-02	0.9	1.6E-02	0.9	8.6E-02	0.9
135	CLIC1_HUMAN	CLIC1	5.1E-13	1.2E-03	2.1E-02	1.3	2.5E-03	1.5	5.4E-04	1.5	2.7E-04	1.6	1.5E-04	1.6
136	CLUS_HUMAN	CLU	1.4E-13	3.8E-06	4.9E-02	0.8	7.2E-04	0.7	1.8E-07	0.5	2.5E-05	0.6	1.0E-02	0.7
137	CMBL_HUMAN	CMBL	3.8E-02	1.0E-03	2.8E-02	1.3	5.5E-04	1.6	8.1E-05	1.7	2.6E-03	1.5	1.3E-03	1.5
138	CNDP2_HUMAN	CNDP2	1.1E-12	1.2E-08	3.6E-08	2.1	1.3E-08	2.2	8.8E-08	2.0	1.1E-04	1.7	1.6E-03	1.5
139	CO2_HUMAN	C2	2.2E-01	2.7E-10	4.0E-01	0.9	7.5E-03	0.8	1.9E-05	0.8	5.3E-09	0.6	4.1E-09	0.6
140	CO3_HUMAN	C3	4.0E-10	8.3E-11	2.9E-10	0.5	5.2E-09	0.6	6.0E-11	0.5	1.8E-07	0.6	9.3E-06	0.7
141	CO4A_HUMAN	C4A	6.2E-04	6.3E-01	7.7E-01	1.0	3.9E-01	0.9	1.0E-01	0.8	4.0E-01	0.9	8.1E-01	1.0

No.	Uniprot ID	Gene symbol	*p*-value (Subject) [a)]	*p*-value (Time) [b)]	*p*-value (1 mo *vs.* 0 mo) [c)]	Ratio (1 mo *vs.* 0 mo) [d)]	*p*-value (3 mo *vs.* 0 mo) [c)]	Ratio (3 mo *vs.* 0 mo) [d)]	*p*-value (6 mo *vs.* 0 mo) [c)]	Ratio (6 mo *vs.* 0 mo) [d)]	*p*-value (9 mo *vs.* 0 mo) [c)]	Ratio (9 mo *vs.* 0 mo) [d)]	*p*-value (12 mo *vs.* 0 mo) [c)]	Ratio (12 mo *vs.* 0 mo) [d)]
142	CO4B_HUMAN	C4B	3.6E-12	2.1E-03	8.1E-02	0.7	3.2E-02	0.6	4.1E-05	0.4	2.7E-02	0.6	3.0E-01	0.8
143	CO5_HUMAN	C5	7.9E-04	1.9E-08	4.9E-02	0.9	9.8E-03	0.8	9.5E-06	0.7	1.0E-08	0.7	2.6E-07	0.7
144	CO6_HUMAN	C6	2.5E-01	7.0E-02	1.0E+00	1.0	6.1E-01	1.0	9.2E-01	1.0	1.6E-01	0.9	2.4E-02	0.9
145	CO7_HUMAN	C7	6.8E-15	6.7E-03	9.0E-01	1.0	5.7E-01	1.0	5.0E-02	1.2	9.5E-03	1.2	7.7E-03	1.2
146	CO9_HUMAN	C9	4.2E-05	1.3E-32	2.5E-10	0.7	2.6E-20	0.5	1.6E-24	0.4	4.4E-30	0.4	9.9E-28	0.4
147	COF1_HUMAN	CFL1	4.1E-11	1.1E-04	1.8E-02	1.3	1.3E-03	1.5	3.3E-05	1.7	2.6E-05	1.7	1.0E-04	1.6
148	COMT_HUMAN	COMT	8.0E-03	7.4E-02	4.3E-02	1.2	1.7E-01	1.1	4.7E-03	1.3	5.5E-01	1.1	3.5E-01	1.1
149	COPD_HUMAN	ARCN1	5.2E-01	2.0E-04	2.3E-02	1.2	2.8E-05	1.5	4.0E-04	1.4	1.6E-04	1.4	6.3E-04	1.4
150	CRDL2_HUMAN	CHRDL2	4.2E-13	7.0E-45	1.7E-08	0.7	3.5E-20	0.5	5.2E-32	0.4	6.4E-35	0.3	1.0E-41	0.3
151	CSF1_HUMAN	CSF1	9.3E-02	8.9E-08	2.1E-01	1.1	4.0E-01	1.1	8.0E-03	0.8	5.3E-05	0.7	4.6E-05	0.6
152	CXCL2_HUMAN	CXCL2	1.2E-07	7.2E-05	9.7E-01	1.0	2.9E-01	0.9	1.1E-01	0.8	4.8E-02	0.8	8.6E-06	0.6
153	CYTB_HUMAN	CSTB	1.6E-11	1.1E-04	4.6E-04	3.2	9.5E-05	3.7	5.0E-06	4.9	9.7E-04	3.0	4.8E-04	3.2
154	CYTC_HUMAN	CST3	3.1E-10	1.7E-07	1.7E-01	1.1	7.9E-01	1.0	3.1E-01	0.9	4.2E-04	0.8	6.1E-05	0.8
155	DAG1_HUMAN	DAG1	3.2E-05	7.1E-03	2.3E-01	1.1	2.3E-01	1.1	4.4E-02	1.1	3.0E-03	1.2	6.5E-04	1.2
156	DCXR_HUMAN	DCXR	4.3E-11	3.5E-13	2.4E-05	2.0	1.1E-10	3.0	6.5E-13	3.5	3.8E-11	3.1	1.2E-09	2.8
157	DDAH1_HUMAN	DDAH1	9.1E-10	1.6E-15	9.7E-08	1.8	4.4E-13	2.4	3.8E-14	2.5	1.4E-13	2.5	1.3E-12	2.3
158	DDR1_HUMAN	DDR1	8.9E-08	2.3E-03	5.0E-02	1.1	4.4E-01	1.0	8.5E-01	1.0	5.5E-01	1.0	2.6E-02	0.9
159	DEST_HUMAN	DSTN	8.3E-04	3.4E-05	1.3E-01	1.2	5.6E-04	1.6	5.8E-05	1.7	9.1E-05	1.7	5.9E-05	1.7
160	DHAK_HUMAN	DAK	9.1E-02	2.0E-06	1.9E-02	1.5	3.5E-05	2.0	5.0E-06	2.2	9.8E-06	2.1	9.9E-07	2.4
161	DHPR_HUMAN	QDPR	2.7E-02	5.1E-02	3.7E-02	1.2	3.6E-03	1.2	1.0E-02	1.2	7.8E-02	1.1	2.9E-02	1.2
162	DIAC_HUMAN	CTBS	7.1E-09	7.1E-01	2.2E-01	1.3	1.4E-01	1.3	2.2E-01	1.3	3.1E-01	1.2	1.8E-01	1.3
163	DPP2_HUMAN	DPP7	2.8E-06	7.5E-03	3.4E-02	1.6	1.6E-03	2.0	1.9E-03	2.0	1.9E-03	2.0	1.6E-03	2.0
164	DPP3_HUMAN	DPP3	4.0E-05	2.5E-04	1.6E-02	1.4	7.9E-04	1.6	3.3E-05	1.9	2.1E-03	1.6	9.5E-05	1.8
165	DPYL2_HUMAN	DPYSL2	1.8E-09	1.1E-06	1.1E-04	1.7	4.0E-06	1.9	6.2E-08	2.1	4.8E-06	1.9	2.3E-05	1.8
166	DPYL3_HUMAN	DPYSL3	2.3E-10	4.3E-01	7.6E-01	1.0	9.5E-01	1.0	6.2E-01	1.1	4.9E-01	0.9	1.7E-01	0.8
167	DSC2_HUMAN	DSC2	3.1E-04	5.8E-13	2.7E-04	0.8	7.6E-07	0.7	1.6E-09	0.6	2.1E-13	0.5	1.4E-10	0.6
168	DYHC1_HUMAN	DYNC1H1	2.4E-01	2.9E-01	8.4E-02	1.2	9.7E-02	1.2	3.5E-01	1.1	3.0E-02	1.2	1.1E-01	1.2
169	EF1A1_HUMAN	EEF1A1<br>EEF1A1P24<br>EEF1A1P5<br>EEF1A1P9	1.1E-11	6.5E-05	2.4E-02	1.4	9.4E-04	1.7	2.7E-05	2.0	5.4E-05	2.0	2.6E-05	2.0
170	EF1B_HUMAN	EEF1B2	1.7E-04	5.8E-06	2.5E-02	1.5	2.2E-04	2.1	1.3E-05	2.4	8.7E-06	2.4	2.9E-06	2.6
171	EF1D_HUMAN	EEF1D	6.8E-10	2.7E-08	3.6E-02	1.4	1.2E-04	1.8	1.3E-05	2.0	4.3E-08	2.5	4.9E-08	2.5
172	EF1G_HUMAN	EEF1G	3.4E-05	3.0E-04	1.4E-01	1.3	7.9E-02	1.3	7.7E-04	1.7	1.5E-03	1.7	2.4E-05	2.0
173	EF2_HUMAN	EEF2	8.8E-11	2.0E-04	1.6E-02	1.5	3.5E-04	1.8	3.3E-05	2.0	7.7E-05	1.9	2.7E-04	1.8
174	EFHD1_HUMAN	EFHD1	3.9E-07	3.0E-04	6.3E-03	1.5	3.8E-03	1.6	2.7E-04	1.8	6.6E-06	2.0	2.4E-03	1.6
175	EGF_HUMAN	EGF	4.2E-05	5.9E-06	4.4E-01	1.0	5.4E-03	0.9	3.4E-03	0.9	1.7E-06	0.8	1.2E-04	0.8

**No.**	**Uniprot ID**	**Gene symbol**	***p*-value (Subject)** [a)]	***p*-value (Time)** [b)]	***p*-value (1 mo *vs.* 0 mo)** [c)]	**Ratio (1 mo *vs.* 0 mo)** [d)]	***p*-value (3 mo *vs.* 0 mo)** [c)]	**Ratio (3 mo *vs.* 0 mo)** [d)]	***p*-value (6 mo *vs.* 0 mo)** [c)]	**Ratio (6 mo *vs.* 0 mo)** [d)]	***p*-value (9 mo *vs.* 0 mo)** [c)]	**Ratio (9 mo *vs.* 0 mo)** [d)]	***p*-value (12 mo *vs.* 0 mo)** [c)]	**Ratio (12 mo *vs.* 0 mo)** [d)]
176	EHD4_HUMAN	EHD4	6.5E-02	7.2E-03	8.7E-02	1.3	3.3E-03	1.5	3.5E-03	1.5	2.4E-03	1.6	1.9E-03	1.6
177	EMAL2_HUMAN	EML2	3.0E-01	1.3E-02	2.3E-02	1.3	1.9E-02	1.3	2.0E-03	1.4	9.3E-04	1.5	2.5E-02	1.3
178	ENOA_HUMAN	ENO1	3.0E-10	2.1E-03	6.1E-04	1.5	5.5E-04	1.5	1.4E-03	1.4	3.1E-02	1.3	1.6E-01	1.2
179	ENPL_HUMAN	HSP90B1	1.6E-12	5.0E-02	3.2E-01	1.1	3.5E-02	1.2	1.2E-02	1.2	5.3E-01	1.1	9.8E-01	1.0
180	EPHA7_HUMAN	EPHA7	2.8E-04	1.8E-11	2.9E-01	0.9	4.2E-04	0.7	2.9E-06	0.7	7.0E-10	0.6	3.0E-10	0.6
181	ERAP1_HUMAN	ERAP1	1.3E-14	2.9E-04	1.9E-01	0.9	8.5E-02	0.9	4.2E-04	0.8	3.3E-05	0.7	3.1E-03	0.8
182	ERP29_HUMAN	ERP29	2.5E-10	1.4E-08	7.4E-04	1.7	1.8E-07	2.5	1.5E-07	2.5	1.3E-08	2.7	6.0E-07	2.3
183	ESTD_HUMAN	ESD	2.2E-01	4.0E-02	5.8E-01	1.1	3.7E-02	1.2	3.3E-02	1.2	3.5E-03	1.3	1.2E-01	1.2
184	EZRI_HUMAN	EZR	3.5E-12	4.6E-03	1.3E-03	1.4	9.0E-04	1.5	4.2E-04	1.5	6.1E-03	1.4	7.0E-03	1.4
185	F10A1_HUMAN	ST13	2.7E-11	2.3E-05	3.0E-02	1.4	8.0E-04	1.7	6.2E-05	1.9	1.6E-06	2.2	1.2E-04	1.9
186	F16P1_HUMAN	FBP1	1.2E-08	9.0E-06	4.9E-04	1.8	8.6E-06	2.1	9.4E-07	2.3	1.6E-05	2.1	1.6E-04	1.9
187	F16P2_HUMAN	FBP2	7.6E-06	6.1E-05	6.4E-03	1.5	1.6E-03	1.6	4.2E-06	2.1	5.1E-05	1.9	4.8E-02	1.3
188	FA12_HUMAN	F12	7.1E-03	9.2E-07	4.0E-01	0.9	6.3E-01	1.0	4.2E-02	0.9	3.6E-05	0.7	6.6E-07	0.7
189	FAAA_HUMAN	FAH	1.4E-02	5.9E-08	4.3E-02	1.4	1.1E-03	1.6	1.4E-05	2.1	3.3E-08	2.7	2.4E-07	2.6
190	FABP7_HUMAN	FABP7	1.9E-05	1.2E-08	1.4E-04	1.9	1.4E-09	3.0	1.0E-07	2.5	3.7E-07	2.4	1.4E-05	2.1
191	FABPH_HUMAN	FABP3	1.2E-13	5.4E-17	2.0E-08	2.1	3.3E-14	2.8	1.1E-15	3.1	5.7E-15	2.9	4.7E-13	2.7
192	FAM3B_HUMAN	FAM3B	9.9E-04	1.8E-02	3.4E-01	1.1	8.7E-03	1.2	1.7E-03	1.2	3.1E-02	1.1	2.2E-01	1.1
193	FAM3C_HUMAN	FAM3C	1.8E-11	1.7E-02	2.5E-02	1.2	1.1E-02	1.2	3.5E-02	1.2	5.7E-02	1.2	8.6E-01	1.0
194	FAS_HUMAN	FASN	3.1E-10	3.7E-11	1.7E-05	2.2	6.0E-08	2.7	9.7E-11	3.4	1.8E-10	3.3	8.5E-10	3.2
195	FAT2_HUMAN	FAT2	1.1E-08	1.5E-06	5.7E-01	1.0	4.6E-01	1.1	3.8E-02	1.2	4.4E-05	1.3	3.0E-06	1.4
196	FCGBP_HUMAN	FCGBP	1.5E-01	1.4E-02	5.9E-02	0.9	3.8E-03	0.8	5.2E-03	0.8	6.4E-03	0.8	2.3E-03	0.8
197	FCL_HUMAN	TSTA3	7.5E-10	1.3E-07	2.8E-05	1.9	5.7E-07	2.1	3.7E-08	2.3	1.6E-06	2.1	5.3E-07	2.2
198	FETUA_HUMAN	AHSG	2.3E-11	4.4E-01	8.3E-01	1.0	8.7E-01	1.0	5.1E-01	1.0	6.6E-01	1.0	1.9E-01	0.9
199	FIBA_HUMAN	FGA	4.9E-19	6.5E-03	2.6E-03	0.7	2.8E-01	0.9	3.8E-01	0.9	2.9E-03	0.7	8.5E-03	0.7
200	FIBB_HUMAN	FGB	9.9E-12	8.4E-05	1.5E-04	0.5	4.3E-02	0.7	1.4E-02	0.7	2.7E-06	0.4	3.0E-03	0.6
201	FIBG_HUMAN	FGG	3.1E-14	1.5E-04	4.5E-05	0.5	1.3E-01	0.8	8.3E-02	0.7	6.7E-05	0.5	3.5E-03	0.6
202	FINC_HUMAN	FN1	5.0E-20	2.6E-10	2.6E-01	0.9	3.1E-03	0.8	1.9E-05	0.7	3.6E-07	0.7	4.1E-10	0.6
203	FKBP4_HUMAN	FKBP4	2.1E-11	1.0E-03	2.7E-03	1.6	4.0E-04	1.8	9.2E-05	1.9	5.0E-04	1.8	1.6E-03	1.7
204	FLNB_HUMAN	FLNB	6.8E-14	2.7E-01	8.6E-01	1.0	1.5E-01	1.2	3.9E-02	1.3	1.9E-01	1.2	5.3E-01	1.1
205	FOLR1_HUMAN	FOLR1	1.1E-17	7.9E-13	8.3E-06	1.3	6.6E-12	1.5	5.7E-13	1.6	8.2E-10	1.5	4.5E-07	1.3
206	FPPS_HUMAN	FDPS	4.2E-09	5.7E-06	1.1E-04	1.9	5.3E-07	2.4	1.6E-06	2.3	4.3E-04	1.8	6.7E-04	1.8
207	FSTL1_HUMAN	FSTL1	1.8E-19	1.3E-14	3.7E-05	1.5	3.7E-08	1.8	8.0E-06	1.6	5.1E-02	1.2	8.5E-03	0.8
208	FUCO_HUMAN	FUCA1	6.7E-03	5.3E-38	2.8E-04	1.4	9.0E-16	2.4	8.7E-27	3.8	4.0E-31	4.7	8.8E-32	4.8
209	FUT6_HUMAN	FUT6	3.4E-01	6.2E-04	2.6E-02	0.9	1.3E-04	0.8	3.9E-04	0.8	6.5E-04	0.8	8.3E-04	0.8
210	G3P_HUMAN	GAPDH GAPDHL6	5.9E-10	8.4E-06	6.5E-03	1.4	1.0E-03	1.5	8.2E-06	1.8	7.9E-06	1.8	2.2E-06	1.8
211	G6PI_HUMAN	GPI	6.0E-09	3.0E-02	2.4E-01	1.2	2.8E-02	1.3	1.2E-01	1.2	2.2E-02	1.3	1.3E-03	1.5

**No.**	**Uniprot ID**	**Gene symbol**	***p*-value (Subject)** [a)]	***p*-value (Time)** [b)]	***p*-value (1 mo *vs.* 0 mo)** [c)]	**Ratio (1 mo *vs.* 0 mo)** [d)]	***p*-value (3 mo *vs.* 0 mo)** [c)]	**Ratio (3 mo *vs.* 0 mo)** [d)]	***p*-value (6 mo *vs.* 0 mo)** [c)]	**Ratio (6 mo *vs.* 0 mo)** [d)]	***p*-value (9 mo *vs.* 0 mo)** [c)]	**Ratio (9 mo *vs.* 0 mo)** [d)]	***p*-value (12 mo *vs.* 0 mo)** [c)]	**Ratio (12 mo *vs.* 0 mo)** [d)]
212	GALE_HUMAN	GALE	6.4E-05	5.0E-14	9.8E-03	1.6	4.0E-08	2.9	7.8E-11	3.7	2.3E-12	4.3	2.1E-11	4.0
213	GALT2_HUMAN	GALNT2	2.6E-08	2.5E-14	5.2E-06	0.8	4.5E-09	0.7	3.8E-11	0.7	1.1E-13	0.6	2.5E-13	0.6
214	GANAB_HUMAN	GANAB	5.8E-13	1.3E-10	2.5E-05	0.8	3.4E-09	0.7	3.1E-12	0.6	1.5E-05	0.8	4.0E-06	0.7
215	GAPR1_HUMAN	GLIPR2	1.3E-05	8.3E-04	1.7E-05	1.5	3.7E-03	1.3	8.3E-02	1.2	7.8E-02	1.2	1.3E-01	1.1
216	GBB1_HUMAN	GNB1	3.0E-06	3.5E-02	6.4E-01	1.0	1.4E-01	0.9	1.5E-01	0.9	2.7E-03	0.8	2.3E-02	0.8
217	GBB2_HUMAN	GNB2	2.8E-07	2.5E-07	8.5E-01	1.0	1.5E-01	0.9	2.7E-01	0.9	1.3E-05	0.7	2.1E-06	0.7
218	GBB4_HUMAN	GNB4	5.3E-06	1.0E-01	9.6E-01	1.0	8.1E-01	1.0	7.4E-01	1.0	1.5E-01	0.9	2.0E-02	0.8
219	GBG12_HUMAN	GNG12	7.4E-02	4.1E-01	2.9E-01	1.1	7.1E-01	1.0	5.7E-01	1.0	4.0E-02	1.2	7.0E-01	1.0
220	GDIA_HUMAN	GDI1	3.8E-06	1.3E-05	4.1E-02	1.2	1.1E-05	1.5	7.5E-06	1.6	5.5E-03	1.3	2.3E-01	1.1
221	GDIB_HUMAN	GDI2	1.3E-09	1.6E-04	7.5E-02	1.2	1.2E-04	1.4	1.6E-05	1.5	4.2E-02	1.2	8.2E-02	1.2
222	GDIR1_HUMAN	ARHGDIA	7.8E-13	5.2E-11	4.3E-04	1.5	7.9E-09	2.0	7.0E-09	2.0	5.7E-10	2.1	6.3E-10	2.1
223	GELS_HUMAN	GSN	2.2E-15	4.8E-44	3.6E-09	1.3	6.9E-24	1.8	2.0E-32	2.2	3.1E-36	2.4	6.9E-40	2.6
224	GFPT1_HUMAN	GFPT1	1.1E-09	1.1E-09	1.8E-04	1.9	5.0E-08	2.7	5.5E-09	2.9	3.0E-09	3.0	4.3E-08	2.7
225	GGT1_HUMAN	GGT4P GGTLC3 GGTLC5P GGT1 GGT2	1.1E-20	0.0E+00	1.1E-12	0.6	7.2E-34	0.3	4.6E-40	0.2	2.7E-40	0.2	3.4E-40	0.2
226	GLCM_HUMAN	GBA	6.6E-03	9.4E-12	4.3E-02	0.9	5.8E-04	0.8	1.5E-04	0.8	3.8E-09	0.6	2.5E-12	0.6
227	GLOD4_HUMAN	GLOD4	2.5E-03	4.0E-06	2.0E-01	1.2	4.5E-04	1.6	6.3E-05	1.8	4.3E-06	1.9	1.4E-05	1.9
228	GLRX1_HUMAN	GLRX	5.0E-07	7.5E-12	2.3E-04	1.6	9.7E-09	2.3	4.3E-10	2.5	6.7E-12	2.8	5.0E-09	2.3
229	GLRX3_HUMAN	GLRX3	1.8E-01	4.2E-02	7.7E-02	1.2	1.6E-02	1.3	8.3E-03	1.4	2.1E-02	1.3	4.5E-03	1.4
230	GLU2B_HUMAN	PRKCSH	4.3E-07	3.9E-05	5.0E-01	1.0	1.1E-01	1.1	5.5E-02	1.1	2.4E-04	1.3	5.3E-04	1.2
231	GLYC_HUMAN	SHMT1	9.2E-06	7.0E-12	3.4E-05	2.0	5.7E-09	2.9	4.2E-12	3.7	3.7E-10	3.2	3.1E-09	3.0
232	GMDS_HUMAN	GMDS	3.2E-08	3.0E-09	3.8E-05	2.0	4.8E-08	2.6	8.0E-10	3.0	1.2E-08	2.8	3.6E-06	2.2
233	GMPPA_HUMAN	GMPPA	2.3E-06	8.9E-04	3.2E-02	1.6	1.0E-04	2.4	2.1E-04	2.3	8.0E-03	1.8	3.5E-03	1.9
234	GMPPB_HUMAN	GMPPB	5.4E-04	1.7E-04	3.2E-02	1.5	3.4E-04	2.1	1.1E-05	2.5	1.5E-03	1.9	5.0E-04	2.1
235	GNAQ_HUMAN	GNAQ	8.7E-07	2.3E-01	6.9E-01	1.1	2.9E-01	1.1	2.0E-01	1.2	1.8E-02	1.4	6.0E-01	1.1
236	GNAS1_HUMAN	GNAS	4.7E-04	4.6E-01	6.0E-01	1.1	6.7E-01	1.1	8.6E-01	1.0	8.8E-01	1.0	1.3E-01	0.8
237	GNPTG_HUMAN	GNPTG	9.3E-04	2.2E-01	5.9E-01	1.0	2.5E-01	0.9	3.2E-02	0.9	5.2E-02	0.9	9.7E-02	0.9
238	GNS_HUMAN	GNS	3.2E-01	1.8E-05	6.5E-02	1.3	1.1E-04	1.7	2.2E-05	1.8	3.1E-05	1.7	5.5E-05	1.7
239	GOLM1_HUMAN	GOLM1	6.6E-07	3.0E-25	1.1E-01	0.9	1.0E-07	0.7	2.5E-15	0.6	6.4E-18	0.5	7.5E-21	0.5
240	GP126_HUMAN	GPR126	5.4E-08	1.1E-11	2.1E-04	0.8	2.0E-11	0.7	1.4E-11	0.7	7.8E-08	0.7	4.0E-06	0.8
241	GPC5B_HUMAN	GPRC5B	2.3E-02	2.0E-01	6.7E-02	0.9	1.2E-01	0.9	4.6E-02	0.9	7.1E-01	1.0	7.6E-01	1.0
242	GPDA_HUMAN	GPD1	3.2E-02	1.6E-03	2.5E-02	1.4	1.9E-03	1.6	1.7E-04	1.7	4.2E-04	1.7	3.4E-03	1.5
243	GPR56_HUMAN	GPR56	7.9E-02	8.3E-02	1.8E-01	0.9	1.4E-02	0.9	1.8E-02	0.9	1.8E-02	0.9	2.3E-01	0.9
244	GPX3_HUMAN	GPX3	3.6E-08	9.8E-17	1.2E-07	1.5	1.2E-09	1.6	1.4E-04	1.3	5.3E-01	1.0	1.7E-02	0.8
245	GPX4_HUMAN	GPX4	2.1E-08	1.1E-04	6.4E-03	1.4	4.0E-06	1.8	1.8E-04	1.6	8.6E-03	1.4	7.1E-02	1.2

No.	Uniprot ID	Gene symbol	*p*-value (Subject) [a)]	*p*-value (Time) [b)]	*p*-value (1 mo *vs.* 0 mo) [c)]	Ratio (1 mo *vs.* 0 mo) [d)]	*p*-value (3 mo *vs.* 0 mo) [c)]	Ratio (3 mo *vs.* 0 mo) [d)]	*p*-value (6 mo *vs.* 0 mo) [c)]	Ratio (6 mo *vs.* 0 mo) [d)]	*p*-value (9 mo *vs.* 0 mo) [c)]	Ratio (9 mo *vs.* 0 mo) [d)]	*p*-value (12 mo *vs.* 0 mo) [c)]	Ratio (12 mo *vs.* 0 mo) [d)]
246	GRN_HUMAN	GRN	2.0E-14	9.8E-28	6.5E-10	1.9	5.8E-19	2.7	2.2E-24	3.5	9.7E-24	3.4	5.4E-23	3.3
247	GRP78_HUMAN	HSPA5	2.9E-13	2.9E-02	5.5E-01	1.0	9.6E-02	1.1	2.2E-02	1.1	1.0E-01	1.1	1.1E-01	1.1
248	GSHB_HUMAN	GSS	1.8E-08	4.9E-07	2.7E-03	1.6	6.8E-06	2.0	2.0E-07	2.3	7.9E-07	2.2	3.2E-06	2.1
249	H2B1B_HUMAN	HIST1H2BB	1.6E-03	5.8E-02	2.2E-01	0.8	1.6E-02	0.7	3.0E-02	0.7	3.1E-02	0.7	6.1E-03	0.7
250	H90B4_HUMAN	HSP90AB4P	6.7E-11	8.4E-05	6.8E-03	1.6	1.5E-04	2.0	1.2E-05	2.3	2.4E-05	2.2	7.6E-04	1.9
251	HDHD2_HUMAN	HDHD2	4.8E-01	9.6E-02	2.5E-01	1.1	1.8E-02	1.2	2.9E-02	1.2	1.2E-02	1.3	1.1E-01	1.2
252	HEBP1_HUMAN	HEBP1	1.6E-05	4.1E-11	1.1E-06	1.9	1.4E-09	2.3	1.3E-11	2.6	5.0E-09	2.2	1.7E-07	2.0
253	HEBP2_HUMAN	HEBP2	2.8E-04	1.7E-04	9.5E-02	1.3	2.3E-04	1.7	3.0E-04	1.7	5.4E-04	1.7	5.2E-05	1.8
254	HEM2_HUMAN	ALAD	4.1E-03	7.7E-03	1.7E-02	1.4	1.9E-03	1.5	3.0E-03	1.5	1.6E-03	1.5	1.8E-03	1.5
255	HEMO_HUMAN	HPX	9.8E-18	2.2E-04	4.7E-02	1.1	1.6E-01	0.9	3.1E-02	0.9	1.5E-01	1.1	4.8E-01	1.0
256	HEP2_HUMAN	SERPIND1	8.7E-05	1.1E-08	2.1E-02	1.1	5.6E-01	1.0	1.1E-01	0.9	1.6E-02	0.9	9.0E-06	0.8
257	HEXA_HUMAN	HEXA	8.7E-06	5.1E-01	2.6E-01	1.1	2.7E-01	1.1	6.3E-02	1.1	1.3E-01	1.1	1.6E-01	1.1
258	HEXB_HUMAN	HEXB	2.2E-21	4.3E-13	1.5E-02	1.2	1.3E-09	1.5	4.4E-11	1.6	3.1E-10	1.6	1.1E-09	1.5
259	HNRPK_HUMAN	HNRNPK	5.0E-02	7.2E-02	6.1E-01	1.0	1.4E-01	1.1	1.1E-01	1.2	4.0E-02	1.2	5.4E-03	1.3
260	HNRPQ_HUMAN	SYNCRIP	3.4E-08	6.5E-05	5.4E-01	1.1	4.7E-03	1.6	1.4E-02	1.5	1.3E-05	2.2	2.8E-04	1.9
261	HPLN3_HUMAN	HAPLN3	1.5E-09	2.8E-22	1.4E-08	0.5	5.1E-14	0.4	3.4E-19	0.3	7.1E-22	0.2	1.3E-16	0.3
262	HPT_HUMAN	HP HPR	8.3E-17	6.9E-37	8.4E-09	0.7	5.8E-20	0.5	5.6E-27	0.4	1.3E-31	0.3	8.2E-33	0.3
263	HS71L_HUMAN	HSPA1L	1.9E-04	8.7E-05	1.7E-01	1.2	4.2E-02	1.2	1.1E-04	1.6	5.1E-04	1.5	2.6E-05	1.6
264	HS90A_HUMAN	HSP90AA1 HSP90AA2	2.1E-11	5.7E-06	2.0E-03	1.7	7.4E-05	1.9	4.1E-06	2.2	7.4E-07	2.3	2.2E-05	2.0
265	HS90B_HUMAN	HSP90AB1	1.3E-12	1.1E-05	2.1E-03	1.7	1.3E-04	2.0	6.8E-06	2.3	1.6E-06	2.4	2.0E-05	2.2
266	HSP13_HUMAN	HSPA13	2.7E-02	7.4E-02	9.8E-01	1.0	2.3E-01	1.1	5.2E-02	1.2	1.6E-02	1.2	5.6E-02	1.2
267	HSP71_HUMAN	HSPA1A HSPA1B	9.4E-12	1.3E-06	1.9E-01	1.2	6.7E-03	1.3	1.9E-05	1.6	1.5E-05	1.6	1.5E-06	1.7
268	HSP74_HUMAN	HSPA4	4.1E-03	2.1E-01	6.9E-02	1.4	1.6E-02	1.6	1.2E-01	1.3	1.6E-01	1.3	4.0E-01	1.2
269	HSP7C_HUMAN	HSPA8	3.4E-11	1.3E-05	9.3E-03	1.3	1.7E-04	1.5	6.5E-06	1.7	6.2E-06	1.7	1.6E-05	1.6
270	HSPB1_HUMAN	HSPBL2 HSPB1	7.2E-05	3.6E-23	3.5E-06	2.5	1.1E-14	5.5	2.5E-18	7.5	1.9E-20	9.0	5.6E-19	7.9
271	HV101_HUMAN		2.2E-02	3.4E-04	1.7E-03	0.7	5.7E-04	0.7	2.1E-04	0.7	1.4E-02	0.8	5.1E-01	0.9
272	HV206_HUMAN		3.3E-06	3.7E-03	1.9E-02	0.8	1.2E-01	0.9	9.0E-03	0.8	8.4E-01	1.0	3.8E-01	1.1
273	HV208_HUMAN		8.6E-02	2.8E-02	2.7E-02	0.8	1.5E-01	0.9	6.5E-02	0.8	5.8E-01	1.1	9.4E-01	1.0
274	HV209_HUMAN		5.5E-06	3.0E-04	4.8E-03	0.8	1.5E-02	0.8	1.0E-03	0.8	9.0E-02	0.9	5.2E-01	1.0
275	HV301_HUMAN		2.5E-09	3.7E-06	1.3E-03	0.8	8.8E-03	0.8	2.7E-03	0.8	1.9E-01	0.9	1.1E-01	1.1
276	HV302_HUMAN		1.0E-01	1.5E-01	1.6E-01	0.9	2.1E-01	0.9	1.0E-01	0.9	2.2E-01	0.9	4.5E-01	1.0
277	HV304_HUMAN		1.2E-06	3.1E-04	2.0E-02	0.8	1.5E-03	0.7	3.2E-04	0.7	7.8E-02	0.8	9.2E-01	1.0
278	HV305_HUMAN		1.0E-08	4.7E-03	1.4E-02	0.8	1.7E-01	0.9	1.1E-01	0.9	4.1E-01	0.9	1.6E-01	1.1
279	HV306_HUMAN		1.9E-04	1.1E-04	1.0E-02	0.8	1.1E-02	0.8	5.3E-04	0.7	1.3E-01	0.9	3.7E-01	1.1

No.	Uniprot ID	Gene symbol	*p*-value (Subject) [a)]	*p*-value (Time) [b)]	*p*-value (1 mo *vs.* 0 mo) [c)]	Ratio (1 mo *vs.* 0 mo) [d)]	*p*-value (3 mo *vs.* 0 mo) [c)]	Ratio (3 mo *vs.* 0 mo) [d)]	*p*-value (6 mo *vs.* 0 mo) [c)]	Ratio (6 mo *vs.* 0 mo) [d)]	*p*-value (9 mo *vs.* 0 mo) [c)]	Ratio (9 mo *vs.* 0 mo) [d)]	*p*-value (12 mo *vs.* 0 mo) [c)]	Ratio (12 mo *vs.* 0 mo) [d)]
280	HV307_HUMAN		1.1E-08	2.2E-05	4.8E-05	0.7	1.4E-04	0.7	5.2E-06	0.7	1.3E-03	0.8	9.5E-02	0.9
281	HV319_HUMAN		7.3E-02	1.5E-02	2.0E-01	0.9	6.2E-01	1.0	7.2E-01	1.0	4.8E-01	1.1	2.2E-02	1.2
282	HV320_HUMAN		1.1E-08	6.1E-07	1.9E-04	0.8	5.1E-04	0.8	7.4E-05	0.8	2.4E-01	0.9	5.7E-01	1.0
283	HYOU1_HUMAN	HYOU1	1.2E-03	2.0E-01	5.8E-01	1.0	8.1E-01	1.0	6.7E-01	1.0	1.5E-01	0.9	3.8E-02	0.9
284	I18BP_HUMAN	IL18BP	1.5E-05	5.6E-06	7.9E-01	1.0	5.3E-01	0.9	8.9E-03	0.7	1.3E-04	0.6	2.3E-06	0.5
285	IBP2_HUMAN	IGFBP2	2.2E-21	1.1E-11	1.5E-07	1.5	2.2E-03	1.2	1.3E-01	1.1	3.9E-01	0.9	3.9E-02	0.9
286	IC1_HUMAN	SERPING1	1.4E-10	4.3E-29	1.1E-01	0.9	9.5E-09	0.8	2.9E-18	0.7	8.2E-22	0.6	1.2E-22	0.6
287	ICAM1_HUMAN	ICAM1	1.9E-03	7.7E-05	2.6E-01	1.2	1.0E-01	1.2	3.5E-01	1.1	8.4E-04	1.6	6.4E-06	1.8
288	IDHC_HUMAN	IDH1	2.1E-12	8.9E-11	8.3E-08	2.1	2.9E-10	2.5	7.4E-11	2.6	1.2E-08	2.2	2.2E-08	2.2
289	IF4A1_HUMAN	EIF4A1<br>EIF4A1P4<br>SNORA67	5.6E-04	6.3E-01	5.3E-01	1.1	1.2E-01	1.3	3.9E-01	1.2	3.3E-01	1.2	9.6E-01	1.0
290	IF5A1_HUMAN	EIF5AL1<br>EIF5A	8.2E-11	8.4E-06	1.2E-03	1.6	8.8E-05	1.8	1.1E-06	2.1	3.6E-06	2.0	7.2E-05	1.8
291	IGHA1_HUMAN	IGHA1	5.0E-07	1.5E-06	2.8E-03	0.8	8.3E-03	0.8	3.1E-03	0.8	4.7E-01	0.9	5.7E-02	1.2
292	IGHA2_HUMAN	IGHA2	3.5E-07	1.2E-06	1.9E-03	0.8	2.8E-03	0.8	8.8E-04	0.8	3.4E-01	0.9	1.3E-01	1.1
293	IGHG1_HUMAN	IGH@<br>IGHG3<br>IGHM<br>IGHV3-11<br>IGHV3-7<br>IGHG1<br>IGHV4-31	7.4E-15	7.2E-39	6.5E-09	1.3	5.5E-23	1.7	3.6E-30	2.0	1.5E-32	2.1	6.2E-34	2.2
294	IGHG2_HUMAN	IGHG2	7.5E-13	4.7E-38	1.4E-11	1.4	7.7E-26	1.8	9.8E-31	2.0	9.6E-32	2.0	6.7E-34	2.1
295	IGHG3_HUMAN	IGH@<br>IGHG3<br>IGHM<br>IGHV3-11<br>IGHV3-7<br>IGHG1<br>IGHV4-31	1.8E-10	1.1E-36	2.8E-09	1.3	8.4E-23	1.7	6.0E-29	2.0	1.8E-30	2.1	7.1E-33	2.2
296	IGHG4_HUMAN	IGHG4	6.4E-05	1.8E-18	1.7E-05	1.3	3.3E-13	1.8	1.5E-15	2.0	9.2E-16	2.0	2.5E-17	2.1
297	IGHM_HUMAN	IGH@<br>IGHG3<br>IGHM<br>IGHV3-11<br>IGHV3-7<br>IGHG1<br>IGHV4-31	3.8E-10	1.7E-27	4.0E-15	0.4	1.0E-20	0.3	3.5E-25	0.3	1.5E-25	0.3	1.5E-20	0.3
298	IGJ_HUMAN	IGJ	1.7E-04	1.6E-06	3.2E-02	0.9	5.2E-01	1.0	4.9E-01	1.0	3.5E-02	1.2	1.4E-03	1.3
299	IGKC_HUMAN	IGKC	2.4E-06	1.6E-06	1.9E-03	0.8	3.0E-02	0.8	4.4E-03	0.8	4.9E-01	0.9	3.9E-02	1.2
300	IGLL5_HUMAN		3.7E-07	1.1E-07	1.2E-03	0.8	2.0E-02	0.8	1.5E-02	0.8	1.0E+00	1.0	1.0E-02	1.2

**No.**	**Uniprot ID**	**Gene symbol**	***p*-value (Subject)** [a)]	***p*-value (Time)** [b)]	***p*-value (1 mo *vs.* 0 mo)** [c)]	**Ratio (1 mo *vs.* 0 mo)** [d)]	***p*-value (3 mo *vs.* 0 mo)** [c)]	**Ratio (3 mo *vs.* 0 mo)** [d)]	***p*-value (6 mo *vs.* 0 mo)** [c)]	**Ratio (6 mo *vs.* 0 mo)** [d)]	***p*-value (9 mo *vs.* 0 mo)** [c)]	**Ratio (9 mo *vs.* 0 mo)** [d)]	***p*-value (12 mo *vs.* 0 mo)** [c)]	**Ratio (12 mo *vs.* 0 mo)** [d)]
301	IL19_HUMAN	IL19	5.5E-05	4.7E-19	2.3E-02	1.2	1.2E-06	1.6	2.5E-12	2.0	1.8E-15	2.3	3.4E-16	2.4
302	ILEU_HUMAN	SERPINB1	8.1E-03	1.8E-02	2.4E-01	1.1	3.8E-03	1.4	2.3E-02	1.3	5.7E-03	1.4	6.1E-03	1.4
303	IMB1_HUMAN	KPNB1	2.7E-03	1.9E-01	3.2E-01	1.2	5.8E-02	1.4	6.0E-02	1.4	6.2E-02	1.4	2.3E-02	1.5
304	INADL_HUMAN	INADL	4.6E-03	6.3E-01	6.0E-01	1.1	4.7E-01	1.1	8.2E-01	1.0	3.9E-01	1.2	8.9E-02	1.4
305	IPYR_HUMAN	PPA1	1.6E-05	5.5E-13	1.5E-03	1.6	2.6E-08	2.3	8.7E-10	2.6	7.5E-12	3.0	2.1E-11	2.9
306	IQGA1_HUMAN	IQGAP1	4.6E-12	4.9E-03	3.6E-01	1.1	3.5E-02	1.3	2.5E-03	1.5	8.6E-03	1.4	8.1E-04	1.6
307	ITIH1_HUMAN	ITIH1	1.1E-08	2.3E-03	2.1E-01	1.1	4.9E-01	1.0	1.3E-01	0.9	1.1E-01	0.9	1.7E-02	0.9
308	ITIH2_HUMAN	ITIH2	5.6E-09	5.1E-15	3.1E-02	1.1	2.8E-01	1.0	5.0E-02	0.9	4.5E-04	0.9	7.2E-10	0.8
309	ITIH4_HUMAN	ITIH4	6.2E-13	1.9E-23	1.2E-06	0.8	1.2E-11	0.7	3.3E-15	0.6	1.2E-18	0.6	2.5E-23	0.5
310	K1C9_HUMAN	KRT9	7.0E-05	1.8E-01	3.3E-02	2.4	8.0E-01	0.9	1.9E-01	1.7	2.4E-01	1.6	6.8E-01	1.2
311	K2C1_HUMAN	KRT1	1.0E-04	3.0E-03	4.8E-02	2.0	6.3E-02	0.5	4.0E-02	0.5	9.8E-01	1.0	6.2E-01	0.9
312	K6PL_HUMAN	PFKL	2.8E-01	3.7E-01	2.6E-01	1.1	1.1E-01	1.1	4.2E-02	1.2	9.0E-02	1.2	2.6E-01	1.1
313	KAD1_HUMAN	AK1	3.8E-11	2.5E-08	1.5E-03	1.7	2.3E-07	2.5	2.8E-08	2.8	2.0E-06	2.3	7.9E-08	2.6
314	KAIN_HUMAN	SERPINA4	8.6E-01	1.2E-02	2.0E-01	1.0	4.9E-03	1.1	4.0E-01	1.0	5.2E-01	1.0	3.1E-01	1.0
315	KCD14_HUMAN	KCTD14	4.0E-01	9.9E-02	8.4E-01	1.0	7.8E-01	1.0	6.5E-01	1.0	3.0E-02	1.3	6.0E-02	1.2
316	KCY_HUMAN	CMPK1	8.1E-13	2.7E-14	1.9E-05	2.1	5.3E-10	3.2	2.4E-11	3.5	5.2E-13	4.0	4.8E-13	4.1
317	KLK6_HUMAN	KLK6	5.3E-16	4.7E-29	6.6E-05	1.3	1.2E-13	1.8	7.6E-20	2.2	8.1E-23	2.4	3.3E-26	2.7
318	KNG1_HUMAN	KNG1	1.5E-07	6.5E-27	3.6E-09	0.8	1.3E-13	0.7	8.8E-20	0.6	1.3E-22	0.6	8.4E-26	0.6
319	KPYM_HUMAN	PKM2	6.1E-12	2.0E-05	9.4E-02	1.3	7.0E-03	1.5	1.1E-03	1.6	6.4E-05	1.8	1.4E-06	2.1
320	KV105_HUMAN		1.1E-04	3.7E-05	1.0E-02	0.8	5.8E-02	0.8	3.7E-01	0.9	6.4E-01	1.0	9.0E-03	1.3
321	KV106_HUMAN		1.4E-04	6.5E-05	3.9E-03	0.8	5.0E-03	0.8	1.6E-03	0.8	8.8E-02	0.9	3.7E-01	1.1
322	KV116_HUMAN		1.1E-02	4.7E-02	6.3E-02	0.8	1.7E-01	0.9	3.8E-02	0.8	3.8E-01	0.9	4.2E-01	1.1
323	KV117_HUMAN		5.0E-05	3.4E-05	4.6E-03	0.8	2.1E-01	0.9	1.3E-01	0.9	3.9E-01	1.1	3.0E-02	1.2
324	KV119_HUMAN		2.3E-03	8.8E-02	5.0E-01	0.9	2.8E-01	0.9	7.5E-02	0.8	4.1E-01	1.1	3.4E-01	1.1
325	KV121_HUMAN		7.4E-02	6.5E-05	8.6E-03	0.8	5.7E-02	0.9	4.5E-02	0.8	5.8E-01	1.0	1.7E-02	1.2
326	KV122_HUMAN		4.3E-04	2.1E-05	3.3E-01	0.9	5.8E-01	1.0	1.7E-01	1.1	9.4E-02	1.2	1.3E-05	1.5
327	KV203_HUMAN		4.0E-07	4.6E-07	1.5E-03	0.8	4.2E-03	0.8	2.8E-05	0.7	3.6E-01	0.9	2.9E-01	1.1
328	KV204_HUMAN		6.6E-07	9.0E-07	3.6E-03	0.8	6.9E-03	0.8	4.7E-06	0.7	1.7E-01	0.9	4.3E-01	1.1
329	KV206_HUMAN		3.8E-01	4.3E-02	1.3E-01	0.9	2.1E-01	0.9	1.7E-01	0.9	9.4E-01	1.0	1.9E-01	1.1
330	KV301_HUMAN		5.5E-08	3.5E-02	7.0E-02	0.8	5.0E-02	0.8	4.3E-03	0.7	2.0E-01	0.9	8.9E-01	1.0
331	KV302_HUMAN		5.1E-01	3.0E-02	1.8E-02	0.9	4.4E-02	0.9	8.2E-03	0.9	7.7E-02	0.9	8.6E-01	1.0
332	KV303_HUMAN		1.2E-02	2.6E-01	1.8E-01	0.9	4.6E-01	1.0	9.3E-02	0.9	5.8E-01	1.0	5.8E-01	1.0
333	KV305_HUMAN		7.7E-03	2.7E-02	2.5E-02	0.9	1.6E-02	0.8	4.3E-03	0.8	9.4E-02	0.9	6.1E-01	1.0
334	KV308_HUMAN		2.7E-04	5.1E-01	2.2E-01	0.9	2.9E-01	0.9	1.8E-01	0.9	8.1E-02	0.8	1.0E-01	0.8
335	KV309_HUMAN	IGKV3D-11	1.6E-06	1.4E-01	1.9E-01	0.8	7.3E-01	0.9	2.2E-01	0.8	5.9E-01	0.9	1.0E-02	0.7
336	KV402_HUMAN		3.0E-05	6.4E-04	6.8E-02	0.8	4.9E-03	0.8	6.3E-04	0.7	2.6E-01	0.9	5.7E-01	1.1

**No.**	**Uniprot ID**	**Gene symbol**	***p*-value (Subject)** [a)]	***p*-value (Time)** [b)]	***p*-value (1 mo *vs.* 0 mo)** [c)]	**Ratio (1 mo *vs.* 0 mo)** [d)]	***p*-value (3 mo *vs.* 0 mo)** [c)]	**Ratio (3 mo *vs.* 0 mo)** [d)]	***p*-value (6 mo *vs.* 0 mo)** [c)]	**Ratio (6 mo *vs.* 0 mo)** [d)]	***p*-value (9 mo *vs.* 0 mo)** [c)]	**Ratio (9 mo *vs.* 0 mo)** [d)]	***p*-value (12 mo *vs.* 0 mo)** [c)]	**Ratio (12 mo *vs.* 0 mo)** [d)]
337	LAC2_HUMAN		2.3E-06	1.2E-07	2.3E-03	0.8	3.1E-02	0.8	2.8E-02	0.8	8.6E-01	1.0	5.2E-03	1.3
338	LAC7_HUMAN		1.2E-06	5.2E-05	2.2E-03	0.8	1.7E-02	0.8	2.9E-02	0.8	5.4E-01	1.0	9.2E-02	1.1
339	LALBA_HUMAN	LALBA	1.5E-11	4.6E-09	5.4E-06	1.3	6.0E-05	1.3	3.6E-03	1.2	1.7E-02	1.1	1.5E-01	0.9
340	LASP1_HUMAN	LASP1	2.9E-15	2.6E-12	7.0E-05	1.9	4.1E-07	2.3	8.6E-09	2.7	1.9E-11	3.2	3.5E-12	3.5
341	LDHA_HUMAN	LDHA	8.3E-12	1.4E-08	2.2E-02	1.4	6.3E-05	1.7	2.1E-06	1.9	2.6E-07	2.1	5.6E-09	2.3
342	LDHB_HUMAN	LDHB	2.1E-12	7.7E-14	5.9E-04	1.5	8.1E-09	2.2	7.9E-12	2.6	1.5E-12	2.7	3.7E-11	2.5
343	LEG1_HUMAN	LGALS1	7.2E-01	9.4E-03	4.3E-03	1.2	1.3E-02	1.2	3.5E-03	1.2	3.1E-03	1.2	1.7E-03	1.3
344	LEG3_HUMAN	LGALS3	3.0E-04	1.2E-05	2.3E-01	1.2	5.4E-01	1.1	1.8E-01	1.2	1.2E-05	1.8	5.4E-05	1.7
345	LG3BP_HUMAN	LGALS3BP	6.2E-05	4.1E-20	1.1E-01	0.9	1.1E-06	0.7	6.0E-16	0.5	3.4E-15	0.6	7.5E-13	0.6
346	LGMN_HUMAN	LGMN	9.3E-02	3.8E-02	4.5E-02	0.8	7.3E-02	0.8	9.1E-01	1.0	9.5E-01	1.0	3.2E-01	1.1
347	LGUL_HUMAN	GLO1	3.8E-05	4.0E-03	1.2E-01	1.4	1.8E-02	1.6	1.7E-02	1.6	2.8E-04	2.1	8.5E-04	2.0
348	LIPL_HUMAN	LPL	2.2E-13	3.2E-17	3.9E-07	0.5	2.3E-11	0.4	9.5E-16	0.3	9.3E-17	0.3	6.8E-12	0.4
349	LKHA4_HUMAN	LTA4H	1.8E-03	1.9E-03	6.9E-02	1.3	3.5E-03	1.5	1.7E-04	1.8	4.1E-04	1.7	1.4E-02	1.4
350	LSR_HUMAN	LSR	2.6E-04	4.0E-01	2.2E-01	1.1	8.4E-01	1.0	6.8E-01	1.0	3.4E-01	0.9	6.5E-01	1.0
351	LUM_HUMAN	LUM	3.1E-09	2.8E-05	2.9E-04	1.3	7.6E-03	1.2	5.6E-01	1.0	4.7E-01	0.9	3.3E-01	0.9
352	LV001_HUMAN	IGLV7-43	8.3E-11	4.5E-05	7.1E-04	0.8	7.1E-04	0.8	1.2E-05	0.7	2.1E-02	0.8	4.5E-01	0.9
353	LV102_HUMAN		1.1E-06	1.1E-07	1.8E-03	0.7	3.2E-03	0.7	4.2E-03	0.7	6.8E-01	1.0	7.6E-03	1.3
354	LV105_HUMAN		8.6E-01	6.2E-05	1.7E-04	0.7	5.1E-02	0.8	1.6E-03	0.7	5.0E-01	0.9	2.9E-01	1.1
355	LV301_HUMAN		8.7E-04	4.3E-02	1.1E-02	0.8	3.7E-02	0.8	3.9E-03	0.7	3.6E-02	0.8	2.3E-01	0.9
356	LV302_HUMAN		4.9E-06	7.6E-06	5.1E-04	0.8	5.3E-04	0.8	9.4E-04	0.8	2.6E-01	0.9	5.9E-01	1.0
357	LV401_HUMAN		2.4E-02	2.9E-01	4.5E-01	0.9	7.1E-01	1.1	2.1E-01	0.8	5.0E-01	1.1	4.0E-01	1.1
358	LV403_HUMAN		6.7E-03	4.8E-01	8.5E-01	1.0	5.1E-01	1.0	6.9E-01	1.0	1.9E-01	1.0	6.0E-01	1.0
359	LYPA1_HUMAN	LYPLA1	1.3E-09	9.7E-11	1.2E-04	2.0	1.2E-07	2.8	3.2E-09	3.3	5.8E-10	3.6	5.5E-10	3.7
360	LYPD3_HUMAN	LYPD3	3.2E-01	2.1E-01	8.3E-01	1.0	3.1E-01	0.9	2.7E-02	0.8	3.5E-01	0.9	3.6E-01	0.9
361	LYSC_HUMAN	LYZ	6.2E-10	2.3E-14	3.1E-02	0.7	6.5E-01	1.1	1.3E-02	1.4	2.3E-04	1.7	3.2E-10	2.7
362	MA1A1_HUMAN	MAN1A1	2.2E-02	1.3E-03	2.8E-02	0.9	7.8E-03	0.8	9.6E-04	0.8	1.5E-04	0.8	5.8E-04	0.8
363	MA2B1_HUMAN	MAN2B1	2.5E-01	4.0E-05	1.9E-02	1.2	1.4E-04	1.4	7.6E-05	1.4	1.4E-05	1.4	5.4E-05	1.4
364	MANBA_HUMAN	MANBA	8.1E-06	1.5E-04	5.5E-01	1.0	9.8E-01	1.0	4.5E-01	0.9	2.1E-03	0.8	3.7E-04	0.8
365	MARCS_HUMAN	MARCKS	4.6E-09	1.0E-02	5.1E-01	1.1	6.4E-02	1.2	6.0E-01	1.0	8.7E-02	0.9	2.1E-01	0.9
366	MATN2_HUMAN	MATN2	6.0E-01	1.5E-03	7.6E-01	1.0	3.0E-01	0.9	1.6E-02	0.9	5.1E-04	0.8	2.8E-03	0.8
367	MATN3_HUMAN	MATN3	3.4E-07	1.8E-42	2.6E-13	0.5	2.0E-24	0.3	1.2E-32	0.1	1.4E-37	0.1	2.4E-39	0.1
368	MDHC_HUMAN	MDH1	2.8E-12	2.1E-10	2.6E-04	1.5	1.4E-07	1.9	7.9E-10	2.1	9.5E-10	2.1	5.2E-09	2.0
369	MFGM_HUMAN	MFGE8	1.6E-23	1.1E-18	4.0E-04	0.8	3.6E-16	0.5	3.1E-15	0.6	7.1E-15	0.6	1.4E-08	0.7
370	MOES_HUMAN	MSN	2.9E-12	3.5E-04	4.0E-02	1.2	9.0E-04	1.4	1.8E-04	1.5	2.5E-04	1.4	7.4E-05	1.5
371	MRC1_HUMAN	MRC1	6.3E-19	5.8E-12	1.1E-03	1.3	7.6E-01	1.0	7.8E-01	1.0	2.3E-03	0.8	6.3E-06	0.7
372	MRP_HUMAN	MARCKSL1	6.3E-06	1.1E-01	1.4E-01	0.8	5.6E-02	0.8	1.0E-02	0.7	3.8E-02	0.8	6.1E-02	0.8

No.	Uniprot ID	Gene symbol	*p*-value (Subject) [a)]	*p*-value (Time) [b)]	*p*-value (1 mo *vs.* 0 mo) [c)]	Ratio (1 mo *vs.* 0 mo) [d)]	*p*-value (3 mo *vs.* 0 mo) [c)]	Ratio (3 mo *vs.* 0 mo) [d)]	*p*-value (6 mo *vs.* 0 mo) [c)]	Ratio (6 mo *vs.* 0 mo) [d)]	*p*-value (9 mo *vs.* 0 mo) [c)]	Ratio (9 mo *vs.* 0 mo) [d)]	*p*-value (12 mo *vs.* 0 mo) [c)]	Ratio (12 mo *vs.* 0 mo) [d)]
373	MUC1_HUMAN	MUC1	1.3E-16	2.3E-03	3.2E-02	0.8	3.9E-05	0.7	5.3E-02	0.8	4.6E-03	0.8	5.9E-03	0.8
374	MUC4_HUMAN	MUC4	6.4E-22	2.5E-03	2.4E-01	0.9	3.3E-04	0.7	6.9E-03	0.8	2.4E-03	0.8	3.0E-03	0.8
375	MUCB_HUMAN		5.9E-06	4.3E-19	7.1E-12	0.4	6.3E-16	0.3	2.2E-18	0.2	6.9E-18	0.3	3.6E-14	0.3
376	MUCL1_HUMAN	MUCL1	1.7E-08	3.0E-03	2.7E-02	0.7	4.2E-04	0.5	6.4E-04	0.5	7.6E-02	0.7	2.3E-01	0.8
377	MVD1_HUMAN		1.3E-04	1.4E-03	5.3E-02	1.4	2.5E-03	1.8	2.7E-04	2.1	2.3E-03	1.8	1.1E-03	1.9
378	MYH9_HUMAN	MYH9	4.1E-06	4.5E-01	8.3E-01	1.0	9.4E-01	1.0	1.0E+00	1.0	2.2E-01	1.2	1.0E-01	1.2
379	MYL6_HUMAN	MYL6	1.6E-07	3.1E-04	2.4E-01	1.2	2.8E-02	1.4	7.5E-03	1.5	5.6E-05	1.8	1.7E-04	1.7
380	NAGK_HUMAN	NAGK	1.5E-01	1.3E-01	8.8E-02	1.2	1.2E-02	1.4	2.0E-02	1.3	4.8E-02	1.3	1.1E-01	1.2
381	NAMPT_HUMAN	NAMPT	8.1E-12	5.0E-11	2.9E-07	1.9	7.2E-09	2.1	1.2E-10	2.3	1.8E-10	2.3	1.8E-09	2.2
382	NDKA_HUMAN	NME1-NME2 NME2 NME1	4.1E-06	2.5E-09	8.3E-02	1.3	4.4E-04	1.7	7.8E-07	2.2	2.0E-09	2.7	4.5E-07	2.3
383	NDKB_HUMAN	NME1-NME2 NME2 NME1	5.1E-06	2.0E-09	3.2E-03	1.5	2.5E-04	1.7	2.0E-08	2.4	3.3E-09	2.5	4.1E-08	2.3
384	NDRG2_HUMAN	NDRG2	1.7E-01	6.0E-03	1.1E-01	1.2	4.0E-02	1.2	2.9E-02	1.2	2.9E-03	1.3	2.2E-04	1.4
385	NEUR1_HUMAN	NEU1	2.6E-06	3.3E-12	5.9E-03	0.8	5.1E-01	1.1	2.7E-04	1.4	9.0E-07	1.6	3.8E-06	1.6
386	NEUS_HUMAN	SERPINI1	8.3E-06	1.3E-18	4.8E-04	0.7	1.0E-08	0.5	1.2E-08	0.5	2.0E-15	0.4	6.5E-19	0.3
387	NHRF1_HUMAN	SLC9A3R1	2.6E-17	3.4E-08	6.3E-03	1.4	5.8E-04	1.5	3.2E-06	1.7	2.2E-08	2.0	4.4E-08	1.9
388	NIBL1_HUMAN	FAM129B	8.9E-04	3.1E-02	2.2E-01	1.3	1.9E-02	1.6	3.9E-03	1.8	1.2E-02	1.6	1.9E-02	1.6
389	NLTP_HUMAN	SCP2	6.7E-08	1.1E-30	3.6E-07	2.1	1.7E-16	4.0	8.7E-26	7.4	3.0E-25	7.1	4.9E-26	7.5
390	NPC2_HUMAN	NPC2	1.7E-12	1.5E-07	3.6E-02	1.1	8.3E-06	1.3	3.0E-06	1.3	5.4E-08	1.4	2.0E-05	1.3
391	NPNT_HUMAN	NPNT	8.3E-01	1.2E-03	7.3E-02	0.8	1.3E-02	0.8	9.8E-04	0.7	9.3E-05	0.6	1.4E-03	0.7
392	NPT2B_HUMAN	SLC34A2	7.8E-03	8.3E-01	9.9E-01	1.0	4.3E-01	0.9	9.6E-01	1.0	5.1E-01	1.0	6.0E-01	1.0
393	NSF1C_HUMAN	NSFL1C	2.0E-02	9.3E-03	1.0E-01	1.8	2.9E-01	1.5	7.4E-03	2.8	8.8E-04	3.6	6.9E-03	2.8
394	NUCB1_HUMAN	NUCB1	4.3E-15	1.4E-12	1.5E-03	1.2	5.5E-08	1.4	1.0E-10	1.5	1.5E-12	1.6	2.7E-08	1.4
395	NUCB2_HUMAN	NUCB2	3.4E-08	2.0E-29	7.7E-10	1.6	2.5E-21	2.3	6.4E-26	2.6	1.1E-25	2.6	3.4E-22	2.3
396	OLA1_HUMAN	OLA1	3.2E-02	6.2E-01	2.8E-01	1.1	1.1E-01	1.2	1.3E-01	1.2	3.0E-01	1.1	4.3E-01	1.1
397	OS9_HUMAN	OS9	8.3E-08	9.6E-23	6.5E-03	0.8	3.6E-09	0.7	2.0E-16	0.5	4.7E-16	0.5	1.5E-20	0.5
398	OSMR_HUMAN	OSMR	3.6E-04	2.6E-01	3.2E-02	1.1	5.1E-02	1.1	1.2E-01	1.1	4.0E-01	1.0	3.3E-01	1.1
399	OSTP_HUMAN	SPP1	4.1E-16	3.4E-06	4.8E-01	0.9	6.8E-01	1.0	8.9E-02	1.2	6.9E-03	1.4	1.4E-05	1.7
400	P3IP1_HUMAN	PIK3IP1	6.5E-12	4.3E-01	9.0E-01	1.0	1.7E-01	0.9	2.7E-01	0.9	7.4E-01	1.0	8.8E-01	1.0
401	PA2G4_HUMAN	PA2G4 PA2G4P4	1.1E-10	7.3E-02	4.9E-02	1.3	7.7E-03	1.4	5.5E-02	1.3	9.8E-02	1.2	7.6E-03	1.4
402	PABP1_HUMAN	PABPC1 PABPC1P4	6.0E-09	1.5E-05	9.0E-02	1.3	3.3E-04	1.8	3.1E-06	2.2	3.7E-05	2.0	2.4E-04	1.9
403	PARK7_HUMAN	PARK7	1.5E-11	2.6E-10	2.4E-05	1.6	1.2E-07	1.9	3.8E-10	2.1	5.2E-10	2.1	1.8E-08	2.0
404	PCBP1_HUMAN	PCBP1	1.4E-09	1.9E-08	1.1E-03	1.7	6.5E-06	2.0	5.2E-08	2.4	2.1E-08	2.5	9.6E-08	2.4
405	PCBP2_HUMAN	PCBP2	5.6E-03	1.6E-04	2.5E-02	1.5	3.6E-03	1.7	2.8E-05	2.2	3.7E-04	1.9	4.5E-05	2.2
406	PDC6I_HUMAN	PDCD6IP	1.8E-13	8.9E-08	4.0E-02	1.3	8.5E-05	1.6	8.6E-06	1.7	5.0E-07	1.8	5.2E-08	1.9

**No.**	**Uniprot ID**	**Gene symbol**	***p*-value (Subject)** [a)]	***p*-value (Time)** [b)]	***p*-value (1 mo *vs.* 0 mo)** [c)]	**Ratio (1 mo *vs.* 0 mo)** [d)]	***p*-value (3 mo *vs.* 0 mo)** [c)]	**Ratio (3 mo *vs.* 0 mo)** [d)]	***p*-value (6 mo *vs.* 0 mo)** [c)]	**Ratio (6 mo *vs.* 0 mo)** [d)]	***p*-value (9 mo *vs.* 0 mo)** [c)]	**Ratio (9 mo *vs.* 0 mo)** [d)]	***p*-value (12 mo *vs.* 0 mo)** [c)]	**Ratio (12 mo *vs.* 0 mo)** [d)]
407	PDCD5_HUMAN	PDCD5	4.0E-02	1.8E-02	1.5E-02	1.3	2.0E-02	1.3	1.6E-02	1.3	2.0E-03	1.4	2.7E-03	1.4
408	PDIA1_HUMAN	P4HB	4.1E-08	1.9E-15	4.8E-02	1.2	1.0E-05	1.4	7.4E-12	1.7	1.1E-12	1.8	2.6E-11	1.7
409	PDIA3_HUMAN	PDIA3	4.3E-12	8.6E-02	1.6E-01	0.9	2.7E-01	1.1	1.9E-01	1.1	7.0E-01	1.0	4.6E-01	1.1
410	PDIA4_HUMAN	PDIA4	1.0E-06	7.5E-02	2.4E-01	1.1	4.2E-03	1.3	5.0E-02	1.2	2.0E-01	1.1	5.2E-01	1.1
411	PDXK_HUMAN	PDXK	6.3E-05	2.0E-04	3.0E-02	1.7	1.2E-04	2.5	6.0E-05	2.7	1.1E-04	2.5	7.2E-03	1.9
412	PEBP1_HUMAN	PEBP1	2.4E-15	9.1E-19	2.6E-08	1.9	7.2E-15	2.6	5.1E-17	2.9	1.2E-16	2.9	1.6E-14	2.6
413	PEDF_HUMAN	SERPINF1	7.9E-06	3.8E-12	4.0E-02	0.9	2.2E-04	0.8	6.1E-08	0.7	2.1E-10	0.7	9.5E-11	0.7
414	PERL_HUMAN	LPO	6.6E-11	1.0E-11	2.1E-04	1.5	7.4E-12	2.3	4.7E-11	2.2	2.1E-06	1.7	4.3E-04	1.5
415	PGAM1_HUMAN	PGAM1	3.8E-07	1.5E-07	1.1E-02	1.4	4.2E-05	1.8	5.6E-06	2.0	3.4E-08	2.3	9.3E-07	2.1
416	PGCP_HUMAN	PGCP	9.2E-15	4.3E-19	7.5E-08	1.5	8.7E-17	1.9	6.0E-17	1.9	8.7E-17	1.9	7.9E-11	1.6
417	PGK1_HUMAN	PGK1	5.4E-10	1.4E-08	6.2E-05	1.6	1.2E-06	1.8	1.7E-08	2.0	6.3E-08	1.9	1.8E-08	2.0
418	PGM1_HUMAN	PGM1	5.4E-13	1.1E-04	8.7E-04	1.5	3.0E-05	1.6	6.6E-06	1.7	5.5E-04	1.5	1.2E-03	1.4
419	PGRP2_HUMAN	PGLYRP2	7.7E-02	1.0E-03	4.2E-05	1.3	2.8E-01	1.1	7.2E-01	1.0	4.9E-01	1.0	6.3E-02	1.1
420	PIGR_HUMAN	PIGR	6.5E-02	3.2E-14	3.3E-09	0.7	7.8E-13	0.7	2.3E-14	0.6	1.5E-08	0.7	4.0E-05	0.8
421	PIPNA_HUMAN	PITPNA	3.4E-05	9.8E-05	3.8E-01	1.1	1.1E-03	1.5	3.2E-04	1.6	2.9E-05	1.7	2.2E-03	1.5
422	PIP_HUMAN	PIP	1.5E-08	3.6E-33	1.3E-24	0.2	3.6E-32	0.1	1.1E-29	0.1	2.0E-22	0.2	8.4E-19	0.3
423	PLIN2_HUMAN	PLIN2	2.4E-14	1.6E-16	6.8E-03	1.3	3.6E-07	1.8	1.0E-13	2.4	6.5E-12	2.2	4.3E-14	2.5
424	PLIN3_HUMAN	PLIN3	3.1E-15	2.7E-06	1.7E-05	1.5	1.3E-06	1.6	1.3E-06	1.6	4.4E-06	1.6	2.5E-04	1.4
425	PLMN_HUMAN	PLG	2.6E-05	2.4E-04	1.5E-04	0.8	7.2E-02	0.9	5.5E-03	0.9	6.8E-05	0.8	1.5E-04	0.8
426	PLOD1_HUMAN	PLOD1	2.4E-03	2.8E-03	3.0E-04	0.7	8.2E-03	0.7	8.0E-03	0.7	3.4E-02	0.8	5.3E-01	0.9
427	PLSL_HUMAN	LCP1	4.7E-12	5.2E-01	7.5E-01	1.0	2.1E-01	0.8	4.8E-01	0.9	5.1E-01	0.9	5.1E-01	1.1
428	PLTP_HUMAN	PLTP	1.0E-02	4.4E-03	1.2E-02	0.8	1.4E-03	0.8	1.5E-03	0.8	7.3E-02	0.9	1.0E-03	0.8
429	PNPH_HUMAN	PNP	3.4E-09	4.6E-11	2.9E-07	2.1	2.0E-09	2.5	2.6E-10	2.6	7.2E-11	2.7	5.4E-09	2.4
430	PODXL_HUMAN	PODXL	2.6E-16	2.3E-16	1.3E-01	0.9	6.1E-02	0.8	7.2E-01	1.0	9.7E-05	1.4	3.6E-10	1.9
431	PON1_HUMAN	PON1	2.3E-06	1.2E-05	1.4E-04	1.4	1.6E-03	1.3	3.6E-02	1.2	3.4E-01	1.1	3.0E-01	0.9
432	PP1R7_HUMAN	PPP1R7	6.4E-09	1.3E-07	1.8E-03	1.6	8.9E-07	2.1	4.7E-07	2.2	2.4E-08	2.4	2.4E-04	1.7
433	PPIA_HUMAN	PPIAL3 PPIA	2.2E-14	2.9E-07	1.9E-05	1.7	1.1E-06	1.8	2.4E-07	1.9	1.8E-07	1.9	2.5E-06	1.7
434	PPIB_HUMAN	PPIB	3.6E-12	1.5E-06	7.9E-04	0.8	9.8E-02	0.9	4.5E-01	1.0	3.5E-02	1.1	2.0E-01	1.1
435	PPIC_HUMAN	PPIC	3.3E-03	1.8E-12	2.6E-02	0.8	9.1E-08	0.6	3.1E-11	0.5	5.4E-10	0.6	1.6E-09	0.6
436	PRDX1_HUMAN	PRDX1	5.0E-12	5.5E-09	3.8E-02	1.3	3.8E-05	1.8	5.3E-07	2.1	7.6E-09	2.3	1.9E-07	2.1
437	PRDX2_HUMAN	PRDX2	2.0E-10	3.7E-08	6.4E-03	1.5	1.8E-05	1.9	1.6E-07	2.2	1.9E-08	2.4	9.8E-07	2.1
438	PRDX4_HUMAN	PRDX4	2.5E-12	7.5E-03	2.4E-01	1.2	2.4E-03	1.6	3.9E-02	1.4	1.6E-02	1.4	6.9E-04	1.7
439	PRDX5_HUMAN	PRDX5	5.0E-11	7.9E-11	3.1E-06	2.0	2.8E-08	2.3	1.5E-10	2.6	2.4E-10	2.6	4.2E-09	2.4
440	PRDX6_HUMAN	PRDX6	1.0E-10	5.3E-19	3.3E-04	1.6	5.4E-11	2.4	1.2E-14	3.0	3.0E-16	3.2	7.5E-16	3.2
441	PROF1_HUMAN	PFN1	8.5E-10	1.7E-04	3.2E-03	1.5	6.1E-04	1.6	1.9E-05	1.8	3.3E-05	1.8	2.7E-04	1.6
442	PROM1_HUMAN	PROM1	2.2E-05	3.1E-08	1.0E-03	0.5	3.4E-07	0.4	2.7E-06	0.4	3.5E-02	0.7	4.6E-01	1.1

No.	Uniprot ID	Gene symbol	*p*-value (Subject) [a)]	*p*-value (Time) [b)]	*p*-value (1 mo *vs.* 0 mo) [c)]	Ratio (1 mo *vs.* 0 mo) [d)]	*p*-value (3 mo *vs.* 0 mo) [c)]	Ratio (3 mo *vs.* 0 mo) [d)]	*p*-value (6 mo *vs.* 0 mo) [c)]	Ratio (6 mo *vs.* 0 mo) [d)]	*p*-value (9 mo *vs.* 0 mo) [c)]	Ratio (9 mo *vs.* 0 mo) [d)]	*p*-value (12 mo *vs.* 0 mo) [c)]	Ratio (12 mo *vs.* 0 mo) [d)]
443	PROM2_HUMAN	PROM2	1.0E-04	3.0E-06	2.8E-04	0.6	2.5E-06	0.5	2.9E-06	0.5	4.8E-04	0.6	7.8E-05	0.6
444	PRPS2_HUMAN	PRPS2	3.8E-07	6.2E-08	2.4E-07	2.4	5.6E-07	2.3	3.2E-08	2.6	8.4E-07	2.3	2.5E-04	1.8
445	PRSS8_HUMAN	PRSS8	1.9E-01	1.1E-07	2.9E-05	0.8	1.1E-07	0.7	3.3E-08	0.7	3.3E-04	0.8	8.6E-03	0.9
446	PSA1_HUMAN	PSMA1	6.5E-13	3.5E-06	7.0E-02	1.4	2.4E-03	1.7	2.2E-05	2.1	1.3E-06	2.4	1.5E-05	2.2
447	PSA2_HUMAN	PSMA2	9.4E-07	7.3E-07	4.1E-01	1.1	2.6E-02	1.4	7.2E-06	2.1	1.0E-05	2.0	5.6E-06	2.1
448	PSA3_HUMAN	PSMA3	2.0E-08	2.2E-05	6.5E-02	1.4	8.4E-04	1.8	2.5E-05	2.1	5.3E-06	2.3	2.1E-04	1.9
449	PSA4_HUMAN	PSMA4	3.4E-11	2.2E-06	1.0E-03	1.7	3.0E-05	2.0	1.7E-06	2.2	3.6E-07	2.3	1.8E-05	2.0
450	PSA5_HUMAN	PSMA5	4.8E-07	3.6E-05	7.5E-02	1.4	5.3E-03	1.7	1.8E-04	2.0	6.2E-06	2.4	6.7E-05	2.1
451	PSA6_HUMAN	PSMA6	9.7E-11	1.2E-06	2.4E-02	1.5	1.5E-03	1.7	6.0E-06	2.2	3.4E-07	2.5	4.5E-06	2.2
452	PSA7_HUMAN	PSMA7	1.4E-09	1.8E-07	9.8E-02	1.3	2.4E-03	1.6	1.6E-05	2.0	2.0E-07	2.4	1.2E-06	2.3
453	PSA_HUMAN	NPEPPS	6.1E-10	6.1E-04	3.6E-03	1.7	8.9E-05	2.0	5.7E-05	2.1	1.0E-03	1.8	7.8E-03	1.6
454	PSB1_HUMAN	PSMB1	3.0E-09	2.3E-06	3.1E-01	1.2	3.1E-03	1.6	1.8E-04	1.8	1.8E-05	2.0	1.8E-06	2.3
455	PSB2_HUMAN	PSMB2	1.5E-07	1.8E-03	3.7E-01	1.2	7.7E-03	1.8	2.6E-03	1.9	6.6E-04	2.1	1.5E-03	2.0
456	PSB4_HUMAN	PSMB4	3.6E-03	9.6E-03	5.1E-01	1.2	4.0E-01	1.2	3.3E-02	1.6	1.7E-03	2.1	5.9E-03	1.9
457	PSB6_HUMAN	PSMB6	2.4E-11	5.9E-07	1.4E-01	1.3	7.3E-04	1.8	3.6E-05	2.1	5.3E-07	2.5	3.3E-06	2.3
458	PSB8_HUMAN	PSMB8	7.9E-01	5.8E-04	7.5E-01	1.1	2.6E-02	1.4	1.3E-02	1.5	1.0E-04	2.0	2.4E-03	1.7
459	PSCA_HUMAN	PSCA	2.4E-03	6.8E-20	1.0E-07	0.6	5.2E-16	0.4	1.6E-19	0.3	8.5E-17	0.3	1.2E-15	0.3
460	PSMD1_HUMAN	PSMD1	7.2E-03	2.8E-03	2.5E-01	1.2	1.9E-02	1.6	4.8E-03	1.7	9.0E-04	1.9	5.8E-04	2.0
461	PSME1_HUMAN	PSME1	9.9E-06	1.6E-04	2.4E-02	1.5	8.2E-04	1.8	1.2E-04	2.0	7.2E-06	2.2	1.8E-03	1.7
462	PSME2_HUMAN	PSME2	2.4E-01	9.7E-03	7.5E-01	0.9	9.7E-02	1.4	4.0E-02	1.5	7.9E-03	1.7	1.7E-02	1.6
463	PTGR1_HUMAN	PTGR1	4.1E-05	4.0E-09	1.8E-05	1.7	4.9E-06	1.8	9.4E-09	2.1	9.1E-08	2.0	3.2E-09	2.2
464	PTHR_HUMAN	PTHLH	3.8E-03	2.8E-04	9.1E-02	1.4	8.2E-03	1.7	5.8E-05	2.3	6.4E-05	2.3	2.3E-03	1.8
465	PTPRF_HUMAN	PTPRF	1.1E-02	3.9E-04	8.6E-04	0.7	1.0E-04	0.6	3.5E-04	0.7	2.6E-02	0.8	2.1E-01	0.9
466	PUR6_HUMAN	PAICS	5.4E-06	1.3E-03	2.8E-01	1.2	2.5E-02	1.5	1.1E-01	1.4	1.9E-03	1.8	7.5E-05	2.4
467	PYGB_HUMAN	PYGB	8.2E-10	9.3E-13	2.1E-01	1.2	5.4E-04	1.6	3.1E-07	2.0	1.6E-10	2.5	6.8E-11	2.6
468	PYGL_HUMAN	PYGL	2.2E-08	7.7E-12	5.2E-03	1.5	1.4E-07	2.2	2.1E-09	2.4	3.5E-10	2.6	5.9E-11	2.7
469	PZP_HUMAN	PZP	1.5E-05	4.4E-09	7.6E-05	0.6	5.0E-07	0.5	2.8E-06	0.5	5.0E-09	0.5	4.4E-09	0.5
470	QSOX1_HUMAN	QSOX1	3.0E-16	2.0E-14	1.0E-06	0.8	4.9E-13	0.7	4.2E-13	0.7	1.5E-06	0.8	1.2E-02	0.9
471	RAB10_HUMAN	RAB10	1.1E-08	2.3E-04	1.3E-03	1.4	5.2E-04	1.4	1.1E-05	1.6	1.0E-02	1.3	7.6E-02	1.2
472	RAB14_HUMAN	RAB14	7.9E-06	4.5E-07	4.4E-03	1.3	7.7E-06	1.6	2.1E-07	1.7	1.3E-06	1.7	6.9E-06	1.6
473	RAB18_HUMAN	RAB18	6.7E-14	2.5E-02	3.4E-03	0.8	1.0E-01	0.9	9.6E-01	1.0	2.1E-01	0.9	4.9E-02	0.9
474	RAB1A_HUMAN	RAB1A	5.8E-14	8.8E-08	2.0E-05	1.4	4.0E-07	1.5	2.3E-09	1.7	1.4E-05	1.4	4.8E-05	1.4
475	RAB1B_HUMAN	RAB1B	1.1E-02	2.0E-03	9.2E-03	1.2	8.0E-04	1.3	7.6E-05	1.3	2.6E-02	1.2	3.5E-02	1.2
476	RAB2A_HUMAN	RAB2A	1.1E-09	1.1E-05	2.9E-02	1.2	2.2E-04	1.3	3.3E-07	1.5	8.3E-03	1.2	1.2E-04	1.4
477	RAB2B_HUMAN	RAB2B	1.7E-03	1.9E-01	1.7E-01	1.2	1.4E-01	1.2	2.3E-02	1.3	2.9E-02	1.3	2.8E-01	1.1
478	RAB35_HUMAN	RAB35	9.4E-03	6.5E-01	2.3E-01	1.1	2.1E-01	1.1	1.6E-01	1.1	3.1E-01	1.1	7.6E-01	1.0

No.	Uniprot ID	Gene symbol	*p*-value (Subject) [a)]	*p*-value (Time) [b)]	*p*-value (1 mo *vs.* 0 mo) [c)]	Ratio (1 mo *vs.* 0 mo) [d)]	*p*-value (3 mo *vs.* 0 mo) [c)]	Ratio (3 mo *vs.* 0 mo) [d)]	*p*-value (6 mo *vs.* 0 mo) [c)]	Ratio (6 mo *vs.* 0 mo) [d)]	*p*-value (9 mo *vs.* 0 mo) [c)]	Ratio (9 mo *vs.* 0 mo) [d)]	*p*-value (12 mo *vs.* 0 mo) [c)]	Ratio (12 mo *vs.* 0 mo) [d)]
479	RAB5A_HUMAN	RAB5A	5.8E-08	2.3E-03	1.9E-01	1.1	2.0E-03	1.3	1.2E-03	1.3	5.5E-01	1.0	7.0E-01	1.0
480	RAB5B_HUMAN	RAB5B	8.4E-07	1.1E-04	1.5E-01	1.1	2.7E-04	1.3	7.3E-06	1.4	3.1E-02	1.2	8.7E-02	1.1
481	RAB5C_HUMAN	RAB5C	8.5E-09	4.4E-03	8.5E-02	1.1	6.1E-03	1.2	2.2E-03	1.3	9.9E-01	1.0	5.0E-01	1.1
482	RAB6A_HUMAN	RAB6A RAB6C	3.5E-02	1.6E-03	8.2E-02	1.1	2.3E-03	1.2	2.2E-04	1.3	7.7E-04	1.3	2.4E-03	1.2
483	RAB7A_HUMAN	RAB7A	1.7E-09	1.7E-03	3.7E-01	0.9	3.6E-01	0.9	3.4E-01	0.9	2.2E-02	0.8	9.7E-05	0.7
484	RAB8A_HUMAN	RAB8A	8.5E-01	1.9E-02	6.8E-02	1.1	5.0E-03	1.2	9.7E-04	1.3	4.7E-02	1.1	1.4E-01	1.1
485	RABP1_HUMAN	CRABP1	3.9E-07	1.4E-05	4.4E-05	3.4	3.0E-03	2.3	6.4E-02	1.7	6.7E-01	0.9	7.9E-01	0.9
486	RABP2_HUMAN	CRABP2	1.1E-18	1.7E-18	2.5E-07	1.9	1.8E-12	2.5	8.5E-16	3.0	4.2E-16	3.1	1.6E-16	3.1
487	RAC1_HUMAN	RAC1	1.4E-10	1.5E-02	1.3E-01	1.1	2.7E-02	1.2	6.8E-04	1.3	2.2E-02	1.2	4.8E-03	1.3
488	RADI_HUMAN	RDX	1.4E-10	5.4E-02	6.4E-02	1.2	1.7E-02	1.3	5.7E-03	1.3	9.6E-03	1.3	1.1E-02	1.3
489	RALA_HUMAN	RALA	5.6E-03	4.4E-01	6.6E-01	1.0	2.8E-01	0.9	3.3E-01	0.9	6.2E-02	0.9	1.1E-01	0.9
490	RAN_HUMAN	RAN	4.5E-03	1.6E-02	2.1E-01	1.2	2.8E-02	1.4	2.6E-03	1.7	4.3E-03	1.6	6.8E-03	1.6
491	RAP1A_HUMAN	RAP1A	5.6E-05	2.0E-02	1.9E-02	1.2	5.7E-02	1.2	3.7E-02	1.2	8.4E-01	1.0	6.3E-01	1.0
492	RAP1B_HUMAN	RAP1B	5.1E-02	1.2E-01	7.7E-02	1.1	3.3E-02	1.1	1.3E-02	1.1	1.4E-01	1.1	4.8E-01	1.0
493	RB11A_HUMAN	RAB11A	2.9E-02	5.2E-10	1.1E-04	1.4	2.0E-08	1.8	1.2E-11	2.1	7.2E-06	1.5	2.3E-05	1.5
494	RB11B_HUMAN	RAB11B	7.9E-02	7.0E-04	5.3E-02	1.1	5.0E-03	1.2	1.0E-03	1.2	7.3E-05	1.3	3.4E-04	1.2
495	RD23A_HUMAN	RAD23A	6.2E-07	1.5E-10	9.3E-03	1.5	1.1E-04	1.8	9.6E-10	2.8	2.2E-10	3.0	2.2E-07	2.3
496	RD23B_HUMAN	RAD23B	1.2E-09	2.5E-12	2.1E-03	1.5	1.8E-06	1.9	1.1E-09	2.4	8.3E-12	2.7	1.8E-10	2.5
497	RET1_HUMAN	RBP1	1.0E-07	1.5E-03	7.9E-02	1.3	2.2E-02	1.4	1.8E-05	2.0	2.7E-02	1.4	9.6E-02	1.3
498	RET4_HUMAN	RBP4	3.2E-02	3.8E-01	6.0E-01	1.0	2.0E-01	1.1	9.1E-01	1.0	3.4E-01	0.9	8.5E-01	1.0
499	RHG18_HUMAN	ARHGAP18	6.8E-09	2.9E-17	4.7E-06	2.2	5.0E-13	4.2	3.6E-15	5.1	2.3E-15	5.2	4.3E-14	4.6
500	RHOA_HUMAN	RHOA	6.2E-08	5.2E-06	7.8E-05	1.4	1.1E-06	1.5	2.4E-06	1.5	1.1E-04	1.4	6.9E-03	1.2
501	RHOC_HUMAN	RHOC	1.5E-02	1.0E-03	1.3E-03	1.4	2.0E-04	1.5	1.2E-03	1.4	4.7E-04	1.4	6.4E-03	1.3
502	RINI_HUMAN	RNH1	3.3E-02	1.5E-03	9.4E-02	1.5	2.7E-03	2.0	1.5E-04	2.5	1.3E-03	2.1	1.7E-03	2.1
503	RISC_HUMAN	SCPEP1	9.5E-20	1.4E-05	5.5E-01	0.9	2.6E-01	1.1	6.6E-02	1.2	1.1E-03	1.4	7.2E-05	1.5
504	RNAS4_HUMAN	RNASE4	6.2E-09	4.4E-07	4.1E-05	1.5	1.3E-06	1.7	4.6E-08	1.9	9.6E-06	1.6	2.0E-03	1.4
505	RNT2_HUMAN	RNASET2	1.8E-10	3.5E-06	1.5E-07	0.6	8.3E-05	0.7	5.5E-03	0.7	1.3E-01	0.9	6.2E-02	0.8
506	RRAS_HUMAN	RRAS	2.3E-01	2.7E-03	6.3E-02	1.2	2.6E-03	1.3	7.7E-04	1.4	7.1E-03	1.3	3.4E-04	1.4
507	RRBP1_HUMAN	RRBP1	3.3E-11	4.1E-20	3.1E-03	1.3	8.3E-10	2.0	1.7E-13	2.3	7.8E-15	2.5	4.7E-19	3.0
508	RS12_HUMAN	RPS12P11 RPS12P4 RPS12P9 RPS12	9.0E-04	6.9E-01	4.9E-01	1.1	2.1E-01	1.1	6.7E-01	1.0	2.0E-01	1.1	1.8E-01	1.1
509	RS27A_HUMAN	RPS27A RPS27AP11 RPS27AP12 RPS27AP16	3.4E-09	6.3E-08	2.3E-01	1.1	2.6E-02	1.2	7.3E-06	1.4	4.4E-06	1.5	1.3E-07	1.6

No.	Uniprot ID	Gene symbol	*p*-value (Subject) [a)]	*p*-value (Time) [b)]	*p*-value (1 mo *vs.* 0 mo) [c)]	Ratio (1 mo *vs.* 0 mo) [d)]	*p*-value (3 mo *vs.* 0 mo) [c)]	Ratio (3 mo *vs.* 0 mo) [d)]	*p*-value (6 mo *vs.* 0 mo) [c)]	Ratio (6 mo *vs.* 0 mo) [d)]	*p*-value (9 mo *vs.* 0 mo) [c)]	Ratio (9 mo *vs.* 0 mo) [d)]	*p*-value (12 mo *vs.* 0 mo) [c)]	Ratio (12 mo *vs.* 0 mo) [d)]
		UBB UBC UBA52												
510	S10A1_HUMAN	S100A1	1.2E-10	2.5E-13	4.9E-04	1.7	1.3E-07	2.2	1.5E-11	3.0	3.1E-12	3.1	2.9E-11	2.9
511	S10AB_HUMAN	S100A11 S100A11P1	4.2E-10	7.2E-15	1.1E-02	1.4	3.2E-06	2.0	3.2E-10	2.6	6.7E-12	2.9	8.0E-14	3.3
512	S10AD_HUMAN	S100A13	9.4E-07	1.2E-07	4.2E-04	1.4	1.4E-06	1.7	6.9E-07	1.7	2.2E-06	1.6	7.7E-08	1.8
513	S10AG_HUMAN	S100A16	3.0E-01	4.3E-03	6.0E-02	1.3	3.1E-03	1.4	3.0E-03	1.4	1.8E-02	1.4	6.6E-04	1.6
514	S38AA_HUMAN	SLC38A10	1.9E-08	4.4E-01	1.7E-01	1.1	8.2E-02	1.1	7.7E-02	1.1	4.9E-01	1.0	3.9E-01	1.1
515	SAHH_HUMAN	AHCY	3.2E-09	2.9E-01	5.0E-01	1.1	4.5E-02	1.4	8.5E-02	1.3	6.8E-02	1.3	1.5E-01	1.3
516	SAP3_HUMAN	GM2A	1.6E-11	1.4E-09	1.1E-01	1.1	3.6E-03	0.8	1.4E-05	0.8	9.3E-06	0.8	8.0E-05	0.8
517	SAP_HUMAN	PSAP	2.5E-19	1.1E-13	4.6E-01	1.1	6.7E-05	1.4	9.2E-09	1.6	2.8E-11	1.8	6.2E-09	1.6
518	SAR1A_HUMAN	SAR1A	7.6E-11	6.9E-13	1.0E-04	1.5	3.4E-12	2.3	7.4E-13	2.4	1.8E-08	1.9	9.5E-07	1.7
519	SAR1B_HUMAN	SAR1B	2.7E-01	8.1E-06	2.6E-03	1.3	4.9E-06	1.5	2.5E-06	1.5	2.1E-05	1.5	1.2E-04	1.4
520	SAST_HUMAN	OLAH	1.9E-10	1.9E-08	3.2E-05	1.9	5.3E-07	2.2	1.6E-09	2.7	1.6E-07	2.3	1.8E-06	2.1
521	SBP1_HUMAN	SELENBP1	1.5E-10	8.3E-01	9.9E-01	1.0	2.8E-01	0.9	5.3E-01	0.9	9.4E-01	1.0	8.5E-01	1.0
522	SDC2_HUMAN	SDC2	4.9E-01	1.1E-01	7.2E-02	1.1	5.9E-03	1.1	4.6E-02	1.1	9.3E-02	1.1	2.8E-01	1.1
523	SDC4_HUMAN	SDC4	2.7E-09	3.7E-02	7.0E-01	1.0	4.7E-01	0.9	2.4E-03	0.7	7.7E-02	0.8	2.5E-01	0.9
524	SDCB1_HUMAN	SDCBP	1.4E-02	1.4E-06	2.0E-04	0.6	1.2E-04	0.6	2.4E-07	0.5	1.0E-04	0.6	3.0E-01	0.9
525	SEPP1_HUMAN	SEPP1	8.9E-08	4.7E-08	3.5E-05	0.6	3.4E-07	0.6	1.1E-05	0.6	2.5E-03	0.7	8.8E-01	1.0
526	SERA_HUMAN	PHGDH	8.4E-04	1.3E-10	1.9E-01	1.3	2.9E-04	2.0	5.8E-06	2.5	6.4E-10	3.8	3.8E-09	3.5
527	SFRP1_HUMAN	SFRP1	3.9E-01	8.1E-05	2.3E-02	0.7	1.1E-03	0.6	1.8E-04	0.6	3.7E-05	0.6	2.7E-05	0.6
528	SH3L3_HUMAN	SH3BGRL3	1.0E-06	1.9E-03	1.5E-03	1.6	1.4E-03	1.6	1.6E-04	1.7	2.9E-03	1.5	3.9E-03	1.5
529	SHOT1_HUMAN	KIAA1598	3.1E-01	4.9E-03	1.7E-01	1.2	2.7E-02	1.3	1.5E-03	1.4	1.6E-02	1.3	4.0E-04	1.5
530	SHPS1_HUMAN	SIRPA	1.9E-04	1.5E-20	2.1E-01	1.1	7.9E-05	1.3	1.6E-08	1.4	1.3E-14	1.7	1.1E-18	1.9
531	SIAE_HUMAN	SIAE	9.4E-08	1.1E-07	1.0E-06	0.7	2.1E-08	0.6	6.6E-07	0.7	3.5E-06	0.7	2.3E-05	0.7
532	SIAS_HUMAN	NANS	2.5E-04	4.3E-03	4.1E-02	2.0	3.3E-03	2.9	3.2E-03	2.9	3.7E-04	3.7	3.1E-03	3.0
533	SIL1_HUMAN	SIL1	1.7E-13	4.8E-25	1.1E-08	0.7	3.0E-15	0.6	6.1E-20	0.6	2.3E-22	0.6	4.9E-22	0.6
534	SND1_HUMAN	SND1	1.7E-02	2.2E-01	1.3E-01	1.2	3.4E-02	1.2	5.8E-02	1.2	1.1E-01	1.2	2.6E-02	1.2
535	SNP23_HUMAN	SNAP23	4.1E-05	5.2E-08	9.6E-02	1.3	2.1E-05	2.3	3.8E-07	2.8	7.7E-05	2.1	4.8E-08	3.1
536	SODC_HUMAN	SOD1	9.2E-12	8.9E-08	8.6E-06	1.8	7.2E-07	2.0	5.6E-07	2.0	2.6E-08	2.2	2.2E-06	1.9
537	SOSD1_HUMAN	SOSTDC1	1.2E-06	2.8E-03	1.4E-01	0.8	2.0E-03	0.6	6.6E-02	0.7	1.0E-04	0.5	4.7E-02	0.7
538	SP2_HUMAN	SP2	7.6E-01	1.9E-02	1.0E-02	0.9	3.0E-02	0.9	7.7E-03	0.9	9.3E-02	0.9	9.1E-01	1.0
539	SPIT1_HUMAN	SPINT1	2.1E-03	2.6E-03	5.6E-03	1.2	3.5E-04	1.2	2.9E-04	1.2	2.1E-02	1.1	1.8E-03	1.2
540	SPIT2_HUMAN	SPINT2	1.9E-01	1.0E-02	3.9E-02	1.1	9.0E-04	1.2	4.1E-03	1.1	5.3E-03	1.1	6.4E-03	1.1
541	SRC8_HUMAN	CTTN	5.1E-05	8.7E-08	9.2E-02	1.4	1.3E-03	1.9	2.4E-05	2.3	5.8E-08	3.2	2.9E-07	2.9
542	ST14_HUMAN	ST14	1.8E-03	3.8E-01	5.7E-01	1.0	5.7E-01	1.0	7.3E-01	1.0	2.3E-01	0.9	2.9E-01	0.9
543	STIP1_HUMAN	STIP1	2.3E-09	1.3E-03	2.6E-02	1.5	2.2E-02	1.5	1.8E-03	1.7	4.6E-05	2.0	1.7E-03	1.7

No.	Uniprot ID	Gene symbol	*p*-value (Subject) [a)]	*p*-value (Time) [b)]	*p*-value (1 mo *vs.* 0 mo) [c)]	Ratio (1 mo *vs.* 0 mo) [d)]	*p*-value (3 mo *vs.* 0 mo) [c)]	Ratio (3 mo *vs.* 0 mo) [d)]	*p*-value (6 mo *vs.* 0 mo) [c)]	Ratio (6 mo *vs.* 0 mo) [d)]	*p*-value (9 mo *vs.* 0 mo) [c)]	Ratio (9 mo *vs.* 0 mo) [d)]	*p*-value (12 mo *vs.* 0 mo) [c)]	Ratio (12 mo *vs.* 0 mo) [d)]
544	STX3_HUMAN	STX3	1.5E-08	8.6E-05	1.2E-01	1.4	4.2E-03	1.9	3.0E-05	2.6	9.3E-05	2.4	1.8E-04	2.3
545	STXB2_HUMAN	STXBP2	2.6E-13	8.0E-06	2.1E-02	1.2	9.7E-05	1.5	1.7E-07	1.7	2.4E-04	1.4	5.4E-04	1.4
546	SUMO2_HUMAN	SUMO2P SUMO3 SUMO2	2.7E-01	2.3E-04	2.5E-03	1.4	1.3E-04	1.6	2.3E-04	1.5	2.3E-04	1.5	8.1E-05	1.6
547	SYDC_HUMAN	DARS	1.1E-01	3.5E-01	7.8E-01	1.0	4.2E-01	1.1	9.4E-02	1.1	1.7E-01	1.1	3.0E-01	1.1
548	SYEP_HUMAN	EPRS	5.1E-04	3.4E-03	5.2E-01	1.1	1.4E-02	1.7	5.5E-02	1.5	2.3E-04	2.3	1.3E-02	1.7
549	SYSC_HUMAN	SARS	5.9E-04	2.7E-02	3.9E-02	1.6	3.0E-03	2.0	6.6E-03	1.8	1.2E-02	1.8	1.3E-01	1.4
550	SYTC_HUMAN	TARS	5.2E-06	5.2E-05	1.6E-02	1.5	2.9E-05	2.1	2.3E-05	2.1	8.3E-05	2.0	3.2E-04	1.8
551	SYWC_HUMAN	WARS	6.6E-07	1.8E-03	1.8E-04	1.9	3.3E-03	1.6	2.6E-04	1.9	1.1E-02	1.5	3.2E-02	1.4
552	SZT2_HUMAN		1.4E-16	6.7E-06	7.9E-07	0.6	3.9E-06	0.7	4.7E-05	0.7	5.3E-03	0.8	1.9E-03	0.8
553	TAGL2_HUMAN	TAGLN2	2.1E-07	3.5E-15	1.9E-04	1.9	1.9E-08	2.9	4.1E-12	4.0	4.2E-14	4.7	4.2E-13	4.3
554	TALDO_HUMAN	TALDO1	5.6E-01	1.6E-03	7.8E-01	1.0	1.3E-01	1.1	4.5E-04	1.3	1.7E-03	1.3	3.9E-02	1.2
555	TBA1B_HUMAN	TUBA1B	1.4E-05	3.3E-01	7.1E-01	1.1	2.1E-01	1.3	3.5E-02	1.5	8.7E-01	1.0	4.2E-01	1.2
556	TBB5_HUMAN	TUBBP1 TUBBP2 TUBB	3.4E-04	3.4E-02	9.2E-02	0.7	5.2E-01	1.2	6.5E-01	1.1	8.8E-02	1.6	5.9E-01	0.9
557	TBCA_HUMAN	TBCA	4.2E-05	5.0E-08	2.0E-03	1.6	4.7E-06	2.1	6.3E-07	2.2	6.1E-08	2.4	3.6E-07	2.3
558	TCO1_HUMAN	TCN1	4.1E-14	1.2E-40	8.3E-03	0.8	2.0E-01	1.1	5.0E-10	2.0	1.8E-24	4.0	1.8E-29	5.1
559	TCPA_HUMAN	TCP1 TCP1P3	6.9E-10	8.4E-02	8.1E-02	1.4	8.0E-03	1.6	2.0E-02	1.5	1.2E-02	1.6	4.7E-02	1.4
560	TCPB_HUMAN	CCT2	7.4E-07	1.2E-01	4.3E-02	1.5	1.1E-02	1.7	2.7E-02	1.5	4.6E-02	1.5	1.7E-01	1.3
561	TCPD_HUMAN	CCT4	9.6E-05	1.6E-01	5.0E-01	1.2	6.7E-02	1.5	5.7E-02	1.5	2.5E-02	1.6	5.5E-02	1.5
562	TCPE_HUMAN	CCT5	5.6E-03	5.8E-01	1.9E-01	1.2	2.9E-01	1.1	4.2E-01	1.1	2.7E-01	1.1	6.8E-02	1.2
563	TCPG_HUMAN	CCT3	6.2E-07	1.7E-01	2.5E-01	1.3	2.2E-02	1.6	3.4E-02	1.5	4.2E-02	1.5	5.4E-02	1.5
564	TCPH_HUMAN	CCT7	9.3E-05	1.1E-01	2.9E-01	1.2	1.3E-02	1.7	5.2E-02	1.5	1.9E-02	1.7	1.2E-01	1.4
565	TCPQ_HUMAN	CCT8 CCT8P1	7.3E-11	3.7E-02	2.9E-02	1.5	1.7E-02	1.5	5.2E-03	1.6	2.6E-03	1.7	1.5E-02	1.5
566	TCPZ_HUMAN	CCT6A	2.2E-09	1.5E-01	3.1E-01	1.2	1.3E-02	1.6	2.9E-02	1.5	8.1E-02	1.4	1.5E-01	1.3
567	TCTP_HUMAN	TPT1	7.5E-09	3.6E-07	9.0E-04	1.8	1.3E-06	2.4	9.1E-08	2.6	1.0E-06	2.4	2.6E-05	2.1
568	TENA_HUMAN	TNC	2.4E-06	0.0E+00	2.8E-15	0.3	1.2E-36	0.1	0.0E+00	0.0	0.0E+00	0.0	0.0E+00	0.0
569	TERA_HUMAN	VCP	4.1E-10	6.6E-07	4.2E-02	1.4	9.8E-04	1.8	2.1E-06	2.4	1.5E-07	2.7	4.3E-05	2.1
570	TETN_HUMAN	CLEC3B	2.2E-03	2.0E-13	1.5E-09	1.5	2.9E-13	1.7	8.0E-11	1.6	1.1E-09	1.5	4.0E-10	1.6
571	TGBR3_HUMAN	TGFBR3	4.5E-03	2.5E-01	3.8E-01	0.9	5.7E-01	1.0	8.7E-01	1.0	4.8E-01	1.1	1.6E-01	1.1
572	TGON2_HUMAN	TGOLN2	5.9E-03	1.5E-21	9.3E-06	1.4	9.0E-10	1.7	2.2E-15	2.1	2.8E-19	2.3	3.0E-19	2.3
573	THBG_HUMAN	SERPINA7	1.8E-02	3.1E-17	4.3E-06	0.7	9.0E-11	0.5	8.2E-12	0.5	2.2E-14	0.4	5.8E-18	0.3
574	THIC_HUMAN	ACAT2	2.1E-01	2.3E-09	1.4E-06	2.2	3.4E-09	2.8	1.7E-08	2.6	1.7E-08	2.5	6.0E-07	2.2
575	THIO_HUMAN	TXN	2.9E-06	6.5E-05	1.3E-03	1.6	3.2E-04	1.6	1.4E-05	1.8	9.6E-05	1.7	2.6E-05	1.8

No.	Uniprot ID	Gene symbol	*p*-value (Subject) [a)]	*p*-value (Time) [b)]	*p*-value (1 mo *vs.* 0 mo) [c)]	Ratio (1 mo *vs.* 0 mo) [d)]	*p*-value (3 mo *vs.* 0 mo) [c)]	Ratio (3 mo *vs.* 0 mo) [d)]	*p*-value (6 mo *vs.* 0 mo) [c)]	Ratio (6 mo *vs.* 0 mo) [d)]	*p*-value (9 mo *vs.* 0 mo) [c)]	Ratio (9 mo *vs.* 0 mo) [d)]	*p*-value (12 mo *vs.* 0 mo) [c)]	Ratio (12 mo *vs.* 0 mo) [d)]
576	THRB_HUMAN	F2	5.1E-10	4.7E-10	2.6E-03	0.8	7.1E-07	0.7	4.7E-06	0.7	5.7E-09	0.7	1.3E-10	0.6
577	TIMP1_HUMAN	TIMP1	2.1E-16	1.4E-02	2.0E-02	1.1	6.1E-03	1.2	1.5E-02	1.1	5.4E-01	1.0	6.6E-01	1.0
578	TKT_HUMAN	TKT	6.3E-10	4.6E-06	1.4E-03	1.5	1.9E-04	1.6	4.0E-06	1.8	7.6E-07	1.9	6.6E-06	1.8
579	TLN1_HUMAN	TLN1	1.8E-01	1.6E-02	3.0E-01	1.1	3.4E-02	1.2	1.6E-02	1.3	2.6E-03	1.4	3.2E-03	1.4
580	TLR2_HUMAN	TLR2	1.3E-11	5.6E-10	2.0E-02	0.8	1.6E-02	0.8	3.5E-01	1.1	8.3E-02	1.1	3.5E-06	1.5
581	TM87B_HUMAN	TMEM87B	8.9E-01	8.3E-04	5.1E-01	1.0	8.2E-02	0.9	1.4E-03	0.8	4.5E-03	0.8	4.5E-02	0.9
582	TNF10_HUMAN	TNFSF10	7.6E-03	3.4E-05	5.8E-04	0.4	1.7E-04	0.4	1.3E-05	0.3	6.0E-04	0.5	2.7E-01	0.8
583	TPIS_HUMAN	TPI1 TPI1P1	4.1E-11	3.6E-10	7.2E-04	1.5	1.9E-06	1.7	6.5E-08	1.9	7.0E-10	2.1	5.9E-10	2.1
584	TPM1_HUMAN	TPM1	1.9E-09	2.7E-04	2.0E-01	1.2	1.1E-03	1.8	3.2E-05	2.1	5.4E-04	1.9	1.0E-02	1.6
585	TPM3_HUMAN	TPM3	1.9E-07	1.6E-03	1.1E-01	1.4	1.3E-02	1.6	4.5E-04	2.0	5.2E-03	1.7	1.9E-04	2.1
586	TPM4_HUMAN	TPM4	1.3E-10	2.7E-02	1.2E-01	1.3	6.3E-02	1.4	2.6E-02	1.5	5.5E-04	1.8	9.2E-02	1.3
587	TR11B_HUMAN	TNFRSF11B	1.2E-19	3.2E-09	7.0E-05	0.7	2.9E-06	0.6	1.2E-07	0.6	7.5E-09	0.5	9.1E-10	0.5
588	TRFE_HUMAN	TF	1.6E-15	2.4E-18	4.8E-07	0.8	6.0E-14	0.6	6.4E-12	0.7	4.0E-18	0.6	3.1E-15	0.6
589	TRFL_HUMAN	LTF	9.1E-04	2.4E-20	8.5E-06	0.8	3.0E-14	0.6	7.9E-19	0.5	1.0E-17	0.6	6.4E-12	0.7
590	TRY1_HUMAN	PRSS1 TRY6	2.6E-17	2.9E-01	1.1E-01	0.9	8.1E-01	1.0	8.5E-01	1.0	8.7E-01	1.0	4.5E-01	1.1
591	TSN_HUMAN	TSN	1.1E-07	2.6E-09	2.6E-04	1.8	2.1E-06	2.3	1.6E-08	2.7	2.6E-09	2.9	1.4E-08	2.7
592	TSP1_HUMAN	THBS1	1.0E-09	7.3E-27	1.5E-06	0.4	4.1E-15	0.2	6.8E-22	0.1	1.6E-23	0.1	2.4E-21	0.1
593	TSTD1_HUMAN	TSTD1	6.2E-02	2.7E-09	5.2E-04	1.9	8.7E-06	2.4	5.4E-07	2.8	5.4E-08	3.1	1.6E-10	4.2
594	TTHY_HUMAN	TTR	3.1E-09	6.3E-03	1.8E-01	0.9	1.1E-01	0.9	6.1E-01	1.0	2.5E-01	1.1	1.2E-02	0.8
595	TWF2_HUMAN	TWF2	3.7E-01	1.8E-04	5.6E-03	1.5	1.7E-04	1.7	8.3E-05	1.7	3.4E-05	1.8	1.8E-03	1.5
596	TXD17_HUMAN	TXNDC17	3.7E-09	1.4E-11	3.8E-06	1.9	1.8E-08	2.3	1.4E-10	2.6	2.3E-11	2.7	6.6E-10	2.4
597	TXND5_HUMAN	MUTED TXNDC5	8.8E-03	2.9E-04	1.1E-01	1.1	2.0E-03	1.3	3.9E-01	1.1	8.5E-04	1.3	4.4E-05	1.5
598	TYB10_HUMAN	TMSB10	3.7E-01	1.5E-02	5.5E-03	1.4	1.6E-03	1.4	4.9E-03	1.4	2.9E-02	1.3	6.6E-03	1.4
599	UB2L3_HUMAN	UBE2L3	1.3E-04	1.8E-08	3.3E-04	1.6	3.6E-06	1.9	1.1E-06	2.1	6.8E-08	2.2	1.4E-08	2.4
600	UBA1_HUMAN	UBA1	4.0E-10	3.6E-06	2.3E-03	1.6	6.1E-06	2.0	2.1E-06	2.1	2.1E-06	2.1	2.1E-05	1.9
601	UBE2N_HUMAN	UBE2N	7.7E-03	1.6E-03	2.5E-01	1.1	1.6E-02	1.3	5.2E-03	1.4	7.8E-04	1.5	2.0E-04	1.5
602	UFM1_HUMAN	UFM1	6.4E-02	3.4E-02	4.9E-02	1.3	4.9E-02	1.3	1.1E-01	1.3	5.1E-03	1.5	2.5E-03	1.6
603	UGPA_HUMAN	UGP2	6.1E-12	2.0E-05	9.1E-06	2.0	9.2E-06	2.0	1.8E-05	2.0	2.0E-03	1.6	1.4E-02	1.5
604	VAS1_HUMAN	ATP6AP1	5.3E-11	2.3E-19	9.3E-01	1.0	1.2E-06	1.4	8.7E-10	1.5	5.3E-14	1.7	2.0E-15	1.7
605	VINC_HUMAN	VCL	1.3E-01	1.4E-01	3.3E-01	1.1	7.1E-01	1.0	4.4E-01	1.1	1.0E-02	1.3	9.6E-02	1.2
606	VTDB_HUMAN	GC	2.4E-10	3.1E-03	1.3E-01	0.9	4.9E-01	1.0	1.5E-02	1.1	1.4E-01	1.1	1.7E-01	1.1
607	VTNC_HUMAN	VTN	3.6E-01	2.0E-09	9.8E-01	1.0	2.0E-03	0.9	4.2E-04	0.9	1.3E-05	0.8	1.1E-08	0.8
608	VWA1_HUMAN	VWA1	1.4E-09	2.3E-06	1.9E-05	0.8	2.5E-05	0.8	8.5E-08	0.7	2.1E-05	0.8	7.3E-05	0.8
609	WDR1_HUMAN	WDR1	1.9E-12	1.1E-05	3.1E-03	1.5	1.6E-04	1.7	1.4E-05	1.9	3.7E-06	2.0	4.2E-06	2.0
610	XDH_HUMAN	XDH	2.7E-12	3.0E-10	2.7E-04	1.4	6.7E-10	1.7	5.6E-11	1.8	3.9E-06	1.5	1.9E-05	1.4
611	XPP1_HUMAN	XPNPEP1	4.3E-03	1.9E-03	7.9E-02	1.4	5.3E-04	2.0	1.4E-04	2.2	1.8E-02	1.6	2.4E-02	1.5

**No.**	**Uniprot ID**	**Gene symbol**	***p*-value (Subject)** [a)]	***p*-value (Time)** [b)]	***p*-value (1 mo *vs.* 0 mo)** [c)]	**Ratio (1 mo *vs.* 0 mo)** [d)]	***p*-value (3 mo *vs.* 0 mo)** [c)]	**Ratio (3 mo *vs.* 0 mo)** [d)]	***p*-value (6 mo *vs.* 0 mo)** [c)]	**Ratio (6 mo *vs.* 0 mo)** [d)]	***p*-value (9 mo *vs.* 0 mo)** [c)]	**Ratio (9 mo *vs.* 0 mo)** [d)]	***p*-value (12 mo *vs.* 0 mo)** [c)]	**Ratio (12 mo *vs.* 0 mo)** [d)]
612	YIPF3_HUMAN	YIPF3	2.0E-03	8.0E-01	3.3E-01	1.0	7.2E-01	1.0	4.2E-01	1.0	9.3E-01	1.0	3.1E-01	1.0
613	YKT6_HUMAN	YKT6	2.3E-09	1.2E-01	4.0E-01	1.1	1.6E-02	1.3	1.6E-02	1.3	2.8E-01	1.1	2.1E-01	1.1
614	ZA2G_HUMAN	AZGP1<br>AZGP1P1	8.1E-12	1.0E-23	7.5E-01	1.0	1.8E-07	1.4	1.1E-10	1.5	5.9E-18	1.8	2.2E-17	1.7
615	ZG16B_HUMAN	ZG16B	4.4E-07	2.1E-14	7.5E-12	0.3	8.9E-13	0.3	4.0E-11	0.3	9.6E-13	0.3	5.7E-09	0.4

(a) Significance *p*-value for the changes of relative protein abundance with respect to biological subjects (n = 10); (b) Significance *p*-value for the changes of relative protein abundance with respect to lactation time (x mo / 0 mo, x = 1, 3, 6, 9 and 12); (c) Significance *p*-value for the changes of protein ratios at x mo (x = 1, 3, 6, 9 and 12) with respect to 0 mo; (d) Relative protein abundance ratios (x mo / 0 mo, x = 1, 3, 6, 9 and 12).
